# Refined single-cell profiling captures a CCR5^high^ CD4^+^ cytotoxic T-cell precursor in multiple sclerosis

**DOI:** 10.1016/j.ebiom.2026.106324

**Published:** 2026-06-06

**Authors:** Fabiënne van Puijfelik, Jasper Rip, Yifan van Hasselt, Annet F. Wierenga-Wolf, Sophie Shyfrin, Anna C. Pelser, Marie-Jose Melief, Romy A.M. Klein Kranenbarg, Eric M. Bindels, Harmen J.G. van de Werken, Chaja M.J. van Ansenwoude, Helga E. de Vries, Willem A. Dik, Janet de Beukelaar, Ide Smets, Beatrijs H. Wokke, Joost Smolders, Marvin M. van Luijn

**Affiliations:** a Department of Immunology, Erasmus MC, University Medical Center, Rotterdam, the Netherlands; b MS Center ErasMS, Erasmus MC, University Medical Center, Rotterdam, the Netherlands; c Department of Neurology, Erasmus MC, University Medical Center, Rotterdam, the Netherlands; d Department of Neurology, Albert Schweitzer Hospital, Dordrecht, the Netherlands; e Department of Hematology, Erasmus MC, University Medical Center, Rotterdam, the Netherlands; f Department of Molecular Cell Biology and Immunology, Amsterdam Neuroscience, Amsterdam Institute for Infection and Immunity, Amsterdam UMC Location Vrije Universiteit, Amsterdam, the Netherlands; g MS Center Amsterdam, Amsterdam UMC Location Vrije Universiteit, Amsterdam, the Netherlands; h Laboratory Medical Immunology, Erasmus MC, University Medical Center, Rotterdam, the Netherlands; i Neuroimmunology Research Group, Netherlands Institute for Neuroscience, Amsterdam, the Netherlands

**Keywords:** Multiple sclerosis (MS), CD4^+^ T cell, Cytotoxic T lymphocytes (CTL), Th17.1 cells, CCR5, Single-cell RNA sequencing (scRNA-seq)

## Abstract

**Background:**

Decades of immunological and genetic studies highlight CD4^+^ T cells as initiators of central nervous system (CNS) pathology in individuals who develop multiple sclerosis (MS). A low-frequency CD4^+^ T-cell population termed T helper 17.1 (Th17.1) has been argued to play a central role during MS onset due to differences in abundance, yet little is known about their functional heterogeneity and distinctive mode of action in MS.

**Methods:**

Features that distinguished Th17.1 from Th17 and Th1 cells in the blood were explored using spectral flow cytometry. We focused on the brain-homing properties of Th17.1 in MS by analysing paired blood and cerebrospinal fluid (CSF) as well as blood before and after natalizumab treatment. Identified Th17.1 effector signatures were further analysed using purified subsets for single-cell transcriptomics and corresponding *in vitro* stimulation assays.

**Findings:**

Our screens reveal an CCR5^high^ Th17.1 cluster that is enriched in CSF, but also selectively reduced in post-versus pre-natalizumab blood samples from people with MS. The phenotypical and transcriptional signature of this Th17.1 cluster matched and pointed to a highly receptive and pre-cytotoxic state. Indeed, particularly CCR5^high^ Th17.1 cells upregulated cytolytic proteins by strongly responding to IL-12, while secretion of these proteins was only found when adding both IL-12 and IL-18 *in vitro*.

**Interpretation:**

We show that low-frequency, circulating Th17.1 cells harbour a unique MS-associated CCR5^high^ cluster with enhanced cytotoxic and CNS-infiltrating potential. This CD4^+^ cytotoxic T-cell precursor could be a promising target for future research aimed at monitoring disease-relevant immune perturbations for precision medicine in MS.

**Funding:**

10.13039/501100001826ZonMw (09150171910036), 10.13039/501100003000Stichting MS Research (19–1075 MS, 23–490 g MS), European Union’s Horizon Europe Research and Innovation Actions (101137235; BEHIND-MS) and the 10.13039/501100007352Swiss State Secretariat for Education, Research and Innovation (SERI), Erasmus MC Foundation (10502/02).

**Research in context**

**Evidence before this study**

Although the cause of multiple sclerosis (MS) remains unclear, CD4^+^ T cells are thought to be the first immune cells to breach the blood–brain barrier and initiate local inflammation. Previously, we and others have found indications that circulating, low-frequency T helper 17.1 (Th17.1) cells are prime suspects for such a role. Within the CD4+ memory T-cell pool, Th17.1 is the only subset capable of migrating through a non-inflamed blood–brain barrier *in vitro*. Furthermore, Th17.1 is selectively abundant in the cerebrospinal fluid and targeted by the high-efficacy drug natalizumab (anti-VLA-4 antibody) in people with MS. However, in-depth insight into the functional heterogeneity of Th17.1 cells and the effector programs underlying their distinct mode of action in MS is lacking.

**Added value of this study**

In this study, we performed subset-specific *ex vivo* single-cell phenotypic and transcriptomic analyses as well as short- and long-term *in vitro* stimulations to unveil that human Th17.1 cells harbour an exclusive and functionally distinct CCR5^high^ cluster. This Th17.1 cluster is enriched in the cerebrospinal fluid and specifically reduced in the blood after natalizumab treatment in people with MS. Single cell spectral flow cytometry and RNA sequencing revealed that this cluster expresses a unique set of molecules reflecting both brain-homing and cytotoxic potential. CCR5+ and not CCR5- Th17.1 cells strongly responded to IL-12 to upregulate IL-18 receptor alpha and induce intracellular expression of cytotoxic proteins such as granzyme B and perforin. This was hardly seen for Th1 and Th17 cells. Th17.1 cells were only able to secrete cytotoxic molecules when stimulated with both IL-12 and IL-18, which impaired blood–brain barrier integrity *in vitro*. These findings expand on current knowledge about the way through which human CD4^+^ T cells contribute to MS by putting forward a highly responsive and distinctive Th17.1 cluster with unique cytotoxic features.

**Implications of all the available evidence**

The identification and features of this circulating precursor of CD4^+^ cytotoxic T cells provide a rationale for studying its role within the CNS and as a cellular biomarker and therapeutic target for earlier control of chronic inflammatory diseases like MS.

## Introduction

Multiple sclerosis (MS) is one of the most frequently occurring causes of neurological disability among young adults and is characterised by the entry of different pathogenic lymphocytes into the central nervous system (CNS). Probably years before the diagnosis of MS, CD4^+^ T cells are considered to be the first lymphocytes that breach the blood-CNS barrier, making it also possible for other subsets, such as B and CD8^+^ T cells, to gradually accumulate and eventually initiate MS pathology.^1^

Th17.1, a discrete and low-frequency population of CD4^+^ T cells that has been proposed as a key player in chronic inflammation, is defined by its unique surface expression pattern of chemokine receptors, i.e. high levels of CCR6 and CXCR3 together with low levels of CCR4.^2^, ^3^, ^4^ In contrast to the Th1-like Th17 cells as described in mice, human Th17.1 cells stand out based on strong co-expression of IFN-γ and GM-CSF rather than IL-17 and an enhanced resistance to glucocorticoid treatment.^5^ Despite the fact that this type of CD4^+^ T cells contributes to chronic inflammatory diseases including MS,^6^^,^^7^ these cells are also present in the circulation and CSF of healthy individuals.^4^^,^^5^^,^^8^ Currently, little is known about the heterogeneity of this CD4^+^ T-cell population and which subsets drive pathogenic or protective immunity.

In earlier work, we found several indications that Th17.1 cells are the main CD4^+^ T cells corresponding to early disease activity in MS. In individuals who rapidly develop MS after first clinical symptoms, Th17.1 cells are less present in the blood and enriched in the cerebrospinal fluid (CSF) as compared to controls.^4^^,^^5^ After treatment with natalizumab, an anti-VLA-4 antibody that blocks immune cell migration into tissues such as the CNS, Th17.1 cells selectively accumulated in the blood.^5^ Additionally, of all effector CD4^+^ T cells analysed, Th17.1 is the only population capable of migrating through a non-inflamed blood–brain barrier *in vitro*, a breach that may be induced in a CCR5-and granzyme K (GZMK)-dependent manner.^8^, ^9^, ^10^

This study aimed to uncover features that make Th17.1 cells unique in their mode of action as putative contributors to MS. To reach this, we first used spectral flow cytometry-based deep phenotyping to distinguish features of circulating Th17.1 from Th17 and Th1 cells in healthy blood. Subsequently, these features were associated with their CNS-homing potential by assessing paired blood and CSF samples of untreated MS patients as well as paired blood samples from MS patients before and after natalizumab treatment. Discriminative effector profiles of Th17.1 cells were further elucidated by using purified subsets for single-cell transcriptomics and corresponding short- and long-term *in vitro* stimulation as well as blood–brain barrier integrity assays.

## Methods

### Study samples

Fresh paired peripheral blood (PB) and CSF samples were collected from peoples with MS (pwMS) diagnosed with either progressive or non-progressive MS using the 2017 McDonald criteria.^11^ Blood samples were also collected from healthy controls. Lumbar punctures and blood draws were conducted at the ErasMS tertiary referral center at the Erasmus Medical Center (Rotterdam, The Netherlands). At the time of sampling, pwMS were either treatment-naïve or treated with natalizumab for 6 months. Cells used for *in vitro* stimulation experiments were obtained from healthy donor buffy coats (Sanquin, Amsterdam, The Netherlands). The demographic characteristics of the pwMS and healthy blood donors included in this study are shown in Table 1. Information about gender, ancestry, race and ethnicity of participants was not available.

**Table 1:** Donor characteristics.

	HD blood	HD buffy	MS blood pre- versus post-Tx	MS blood versus CSF
Number	21	14	8	15
Diagnosis RRMS/PPMS	n.a.	n.a.	8/0	3/12
Sex M/F	7/21	6/14	2/8	9/6
Average age (range)	35 (24–61)	48 (25–71)	39 (30–49)	53 (23–71)
Treatment	None	None	6 months with NTZ	None

### Ethics

The study received approval from the medical ethics committee of the Erasmus Medical Center (2019-0845; 2021-0251; 2021-0946; 2023-0691) and all participants provided informed consent.

### Processing of primary material

Buffy coat samples were flushed with PBS containing 0.1% BSA. Peripheral blood mononuclear cells (PBMCs) were isolated using density gradient centrifugation with Ficoll–Paque (GE Healthcare). Memory CD4^+^ T cells were subsequently purified from PBMCs through magnetic-activated cell sorting (MACS) utilising the Memory CD4^+^ T Cell Isolation Kit (Miltenyi Biotec, Cat#130-091-893) on the autoMACS Pro Separator (Miltenyi Biotec), following the manufacturer's protocol. MACS isolated CD4^+^ memory cells were cryopreserved in RPMI 1640 medium (Gibco, Cat# 11875093) supplemented with 20% calf serum (FCS; Gibco, Cat# 10270-106) and 10% dimethyl sulfoxide (Sigma–Aldrich), and stored at −80 °C until further use in experiments. Blood samples from pwMS were collected in Vacutainer CPT tubes (BD Biosciences, Cat# 362753) containing sodium heparin. Peripheral blood mononuclear cells (PBMCs) were isolated from the blood following the manufacturer's protocol. The average frequency of Th17.1 cells among total CD4^+^ memory T cells was 12.6% in healthy donors, 12.0% in untreated pwMS, and 17.8% in NTZ-treated pwMS. Cells from cerebrospinal fluid (CSF)-containing tubes were freshly isolated by centrifugation at 500*g* for 10 min. Isolated cells were re-suspended in 1× phosphate-buffered saline (PBS) at pH 7.4 (Gibco, Cat# 10010023), supplemented with 0.1% bovine serum albumin (BSA; Sigma, Cat# A9647-50G) (PBS–0.1% BSA), and subsequently stained for flow cytometry as detailed below.

### Single cell RNA-sequencing

PBMCs from pwMS prior to and 6 months after NTZ treatment were thawed in FCS and washed with RPMI 1640 medium supplemented with Glutamax (Gibco, Cat# 35050061). Th17.1 and total lymphocytes were isolated by using Fluorescence-Activated Cell Sorting (FACS) using the BD FACSAria™ III Cell Sorter or BD FACSAria™ Fusion Flow Cytometer with a 100 μm nozzle. Cells were collected in 15 ml tubes containing pure FCS, and subsequently resuspended to a concentration of 1000 cells per μl in RPMI supplemented with 10% FCS. Total lymphocytes and purified Th17.1 cells were then loaded onto a 10× Genomics Next GEM K chip kit (10× Genomics) for single-cell partitioning on the Chromium Controller (10× Genomics).

#### Library preparation and sequencing

Final 5′ gene expression (5′ GEX) libraries were prepared following the Chromium Next GEM Single Cell 5′ Kit (v2 chemistry, dual index) User Guide (CG000330, 10× Genomics). Sequencing was performed on an Illumina NovaSeq 6000 platform in paired-end mode (28-10-10-90 cycles), targeting a depth of approximately 25,000 reads per cell. Raw sequencing data were processed using Cell Ranger software (v7.1.0, 10× Genomics), with reads aligned to the GRCh38-1.2.0 reference genome and intronic reads included. This pipeline yielded the final gene expression datasets.

#### Data processing and quality control

All samples were processed in R (v.4.5.0; R Core Team (2025) with Seurat package (v.5.2.1).^12^ After inspection of raw data quality and complexity, the UMI count matrices were filtered with the following criteria: genes expressed in at least 1 cell, cells expressing more than 300 features, more than 500 UMIs, less than 25% mitochondrial reads and more than 10% ribosomal reads. For each sample, we FACS purified Th17.1 cells as well as total lymphocytes from PBMCs, which were separately labelled with hash-tag oligonucleotides (HTOs) TotalSeq™-C hashtags (Biolegend) prior to sorting according to the manufacturer instructions. After sequencing, Th17.1 cells were separated from total lymphocytes using the demuxmix package (v.1.10.0)^13^ for further use in this study. For this, we performed the default demultiplexing steps with the demuxmix algorithm (including k-means clustering with k = 2). As such, we could identify cells that were single positive for each HTO for Th17.1 cells and total lymphocytes, and exclude HTO-negative and HTO-doublet cells from further analysis. The result of the demultiplexing algorithm was checked with cell scatter and ridge plots. This resulted in a total of 21,631 cells in the Th17.1 dataset with an average of 1442 cells per sample.

#### Filtering, data integration, clustering and DEG analysis

This Th17.1 dataset including all samples was further processed with an integrative clustering workflow as described in the Seurat v5 standard pipeline.^12^ First, the RNA data was log-normalised where the highly variable features were identified then used to scale the data to exclude dominant effect from those genes. Linear dimensional reduction was then performed with principal component analysis (PCA), and the dimensionality was determined based on ElbowPlot and gene features in each PC. The samples were split and integrated with the anchor-based reciprocal PCA (RPCA) method, and the nearest neighbours were calculated using shared nearest neighbours (SNN) method with pre-determined number of PCs. Leiden algorithm was used next to estimate and define clusters. Cell embedding for visualisation was achieved with the uniform manifold approximation and projection (UMAP) and Azimuth package (v.0.5.0)^12^ was used for cell annotation. Top differentially expressed features of each cell cluster against the rest of the cells were calculated in aid of further quality check and cell annotation. We further filtered out a cluster of non-lymphoid cells (mainly monocytes based on RNA expression profile), contamination of CD8 cells and a cluster of low-quality cells. The above steps of data normalisation and dimensional reduction were repeated after each filtering. The final seven clusters were identified with the first 23 PCs and Leiden algorithm under 0.5 resolutions. The clustree package (v.0.5.1)^14^ was used to determine the optimal resolution for clustering. Using the cluster tree, we compared top influential transcripts and their biological relevance for each cluster at resolutions of 0.5, 0.6 and 0.7, of which 0.5 appeared to be most optimal. Top influential transcripts at the clustering level were determined using the default settings (Wilcoxon Rank Sum test) of the FindAllMarker function in the Seurat pipeline. Transcripts with adjusted P-values less than 0.05 were defined as significant. After all processing steps, we ended up with a final dataset of 20,737 Th17.1 cells with an average of 1383 cells per sample.

For differential expression analysis comparing gene expression changes across conditions and in between each cluster, considering our sample size and data distribution, we performed cell-level analysis using a two-hurdle model from MAST package (v.1.33.0).^15^ Transcripts with adjusted P-values less than 0.05 were defined as significant. For analysing the differences in percentage of each cluster, we used the Wilcoxon signed-rank test for comparing paired treated (‘NTZ’) and untreated (‘UNTR’) MS samples and the Wilcoxon unpaired test for comparing MS and healthy control samples.

### T-cell clonality analysis

To analyse single-cell T-cell receptor sequencing data, filtered_contig_annotations.csv from Cell Ranger pipeline output was used in the scRepertoire package (v.2.6.2)^16^ to load TCR contigs. When combining TCR contigs into clones, in the presence of multiple chains, the 2 corresponding chains with the highest expression for a single barcode were selected. The top 10 clones from each sample were defined based on the following criteria: 1) clones with information on both the alpha and beta chain; 2) clones expressed in at least 2 cells; and 3) the 10 highest frequencies of clones that were grouped based on CDR3 amino acid sequences. The distribution of these clones was visualised in both UMAPs and bar plots. Statistical significance for clonal distribution was calculated with immunarch package (v.0.10.3).^17^

Clonotype annotation towards validated epitopes was performed by querying identified top clones against public databases (downloaded at 16-12-2025) from the VDJdb (HomoSapiens records; paired gene information; CDR3 amino acid length≥6; database confidence score >0). The distances between our TCR sequences with the reference database entries were calculated using stringdist package (v.0.9.17)^18^ with a Levenshtein distance of≤1 for both chains defined as a match.

### Spectral flow cytometry

Prior to antibody staining, cells were washed in FACSFlow buffer (BD Biosciences) and stained with ZombieNIR™ fixable viability dye (Biolegend, Cat# 15-5825-82) at a 1:2500 dilution in FACSFlow for 10 min in the dark. Antibodies were titrated and validated prior (see Supplementary Table S1 for antibody details) and antibodies were added to the cells in an optimised sequence. First, chemokine receptors were incubated sequentially for 5–20 min at room temperature (RT) and after a washing step, the cells were incubated with antibodies conjugated to brilliant ultraviolet (BUV), violet (BV) and blue (BB) fluorochromes one by one in FACS buffer supplemented with Brilliant Stain buffer BSB (BD Biosciences, according to manufacturer instructions) for 20 min at RT. The samples were then washed again and stained with a mix containing the remaining antibodies specific for extracellular markers for 15 min at RT. Next, for staining of intracellular proteins, cells were subsequently fixed and permeabilized using the BD Pharmingen™ Transcription Factor Buffer Set according to the manufacturer's instructions, followed by staining with antibodies specific for intracellular markers for 40 min in Perm/Wash Buffer (BD Biosciences) at 4 °C in the dark (Supplementary Table S1). Finally, cells were washed, re-suspended in PBS–0.1% BSA and measured using a Cytek® Aurora™ spectral analyser (5-laser; 355, 405, 488, 561, and 640 nm). Data were acquired using SpectroFlo® 3.3.0 (Cytek) and analysed using OMIQ (Dotmatics) software.

### *In vitro* assay for IL-12-induced STAT4 phosphorylation

This assay was performed as previously described.^19^ In this short-term assay, PBMCs from healthy donor blood (1 × 10^6^ cells per well) were seeded in 50 μL RPMI supplemented with 2% FCS. For stimulation, plates were transferred to a 37 °C water bath and cells were incubated with and without 400 ng/mL human recombinant interleukin 12 (IL-12; Biotechne, Cat# 219-IL) for 15 min. After incubation, plates were immediately placed back on ice and cold FACS buffer was added to halt stimulation. Cells were washed and stained with fluorochrome-conjugated antibodies (Supplementary Table S1) against extracellular markers in FACS buffer for 30 min and subsequently with ZombieNIR™ fixable viability dye (Biolegend, Cat# 15-5825-82) in PBS for 10 min at 4 °C in the dark. Fixation and permeabilization were performed using the Transcription Factor Phospho Buffer Set (BD Biosciences; Cat# 563239, RRID: AB_2869473) Cells were incubated for 30 min followed by additional permeabilization with Perm III Buffer for 20 min and intracellular staining with anti-p-STAT4 antibody (Y693, PE-CF594, BD Biosciences) for 30 min at 4 °C in the dark.

### *In vitro* cytokine and chemokine stimulation assay

In this long-term assay, we first purified Th1, Th17 and Th17.1 subsets from healthy blood using FACS. For this, cryopreserved CD4^+^ memory cells were thawed, washed and stained for 30 min at 4 °C in the dark using sorting antibodies (Supplementary Table S1) in PBS containing 0.1% BSA (PBS–0.1% BSA). Subsequently, cells were washed again and sorted using the BD FACSAria™ Fusion into live memory CD4^+^ T cells, following the strategy outlined in Supplementary Fig. S1. Th1 (CCR6^–^CXCR3^+^CCR4^–^), Th17 (CCR6^+^CXCR3^–^CCR4^+^), and Th17.1 (CCR6^+^CXCR3^+^CCR4^–^) subsets were collected in 0.5 ml FCS. Next, cells were washed in culture medium (IMDM; Gibco, Cat# 11504556), 5% FCS, 1% Glutamax, 50 U/ml Penicillin-Streptomycin (Gibco, Cat# 15140122)), and plated in a 96-well culture plate at a density of 20,000 cells per well in medium containing 10 U/ml interleukin 2 (IL-2) (Miltenyi, Cat# 130-097-742) and 5 ng/ml interleukin 15 (IL-15) (Peprotech). Cells were cultured with and without 100 ng/ml IL-12 (Bio-Techne, Cat#219-IL) and 100 ng/ml interleukin 18 (IL-18) (Bio-Techne, Cat#219-IL) for 6 days at 37 °C. Th17.1 cells were also cultured for 2 and 6 days at 37 °C in the presence of 1 μg/ml CCL4 (MIP-1; R&D, Cat#271-BME) or 1 μg/ml CCL5 (RANTES; R&D, Cat#278-RN). After stimulation, cells were stained using the previously described intracellular panel and measured on the 5L-Aurora. Fluorochromes used during sorting were omitted from the staining panel as these were still detectable after culture.

### Luminex assay and ELISA

A custom designed Human Magnetic Luminex Assay® KIT (Biotechne, Cat#LXSAHM) was used to simultaneously quantify cytokine concentrations in supernatants according to the manufacturer's protocol. Assay performance was validated by including internal controls and following recommended quality control procedures to ensure reproducibility and reliability of results. Samples were measured using the Bio-plex magpix multiplex reader (Biorad) with the Bio-plex manager software (V6.1). All measured analytes are shown in Supplementary Table S2. IFN-γ and perforin (PRF) levels were measured separately using the Human IFN-gamma DuoSet (R&D Systems, Cat# DY285B) and the Human Perforin ELISA kit (Abcam, Cat# ab46068). The plates were measured using the Versamax microplate reader with the software pro (V7.1) software.

### Electric cell-substrate impedance sensing (ECIS)

The effect of Th17 and Th17.1 cell-conditioned medium on trans endothelial electrical resistance (TEER) was measured using the ECIS Model 1600R (Applied BioPhysics). The immortalised human cerebral microvascular endothelial cell line (hCMEC/D3; RRID: CVCL_U985) was a kind gift of Prof. dr. I. A. Romero (Open University, UK) and Prof. dr. P. O. Couraud (Université Paris Descartes).^20^ hCMEC/D3 cells are a well-established model of the human blood–brain barrier^21^ and were previously validated.^22^ Cells were cultured in endothelial basal medium-2 (EBM-2) supplemented with 2.5% (v/v) heat-inactivated foetal bovine serum (FBS), 1% (v/v) penicillin-streptomycin, and growth supplement kit according to manufacturer's instructions (EGM-2, Lonza). Cultures were grown on plates coated with type I collagen (Invitrogen) at 37 °C and 5% CO2 until confluent, without reaching past passage 35. Next, cells were detached using trypsin/EDTA in PBS (Gibco) and seeded at a density of 100,000 cells per well into type I collagen-coated 8W10+ ECIS arrays (Ibidi). The impedance Z was measured at multiple frequencies over time. Upon reaching confluence, medium was replaced by serum-free EGM-2 medium for 1 h, followed by replacement with conditioned supernatant of Th17 and Th17.1 cells. As a negative control, HCMEC/D3 cells were cultured with serum-free medium and serum-free medium supplemented with IL-12 (Bio-Techne, Cat# 219-IL) and IL-18 (Bio-Techne, Cat# 219-IL) at 100 ng/ml. Serum-free medium supplemented with TNF-α (250 IU/mL, PeproTech) and IFN-γ (250 IU/mL, PeproTech), with and without IL-12 and IL-18, was used as a positive control for TEER decrease. Impedance was measured for 72 h, after which the resistance [ohm] was quantified by normalising the data at 4000 Hz to the resistance at the time of media replacement, followed by a calculation of the area under the curve (AUC).

### MTS cell proliferation assay

To assess the effect of Th17 and Th17.1-cell conditioned medium on hCMEC/D3 cell proliferation, the hCMEC/D3 cells were seeded in a 96-well plate at 20,000 cells per well and cultured until confluent. Next, medium was replaced by serum-free medium for an hour, followed by culture in cell-conditioned medium or positive and negative controls as stated previously. After 24 h, medium was replaced by MTS reagent according to manufacturer's instructions (CellTiter 96 AQueous One Solution Cell Proliferation Assay, Promega), followed by 1 h incubation at 37 °C and 5% CO2. The absorbance at 490 nm was measured using the BioTek 800 TS microplate reader (Agilent). Data were normalised by dividing sample absorbance values by the absorbance of the negative control condition (serum-free medium without cytokines) and expressed as a percentage.

### Statistical analysis

Comparative analyses of differences between groups were performed using the appropriate relevant statistical methods based on nature of data and normality of data distribution. For paired groups we used either Friedman with Dunn's post hoc analysis, Wilcoxon singed-rank analysis or 2-way ANOVA with post hoc Tukey. For non-paired groups we used the Kruskal Wallis with Dunn's post hoc analysis. Correlations were tested for significance using Pearson r test. Statistical analyses were performed using GraphPad Prism (version 10.0.2, San Diego, CA, USA); specific test for each dataset are indicated in the figure legend. Statistical testing of single-cell sequencing data is described above.

### Data availability

Due to patient privacy reasons, only processed demultiplexed Th17.1-purified Seurat objects from the single-cell sequencing data reported in this study are deposited at the publicly accessible data repository platform DataverseNL (https://doi.org/10.34894/GNSVFB). Other experimental data will be made available from the corresponding author upon reasonable request.

### Code availability

The code described in the methods for the single-cell sequencing analysis is available on GitHub (https://github.com/YFWang-YvH/Refined-single-cell-profiling-captures-a-CCR5high-CD4-cytotoxic-T-cell-precursor-in-MS).

### Role of funders

The funders of the study had no role in experimental design, data collection, data analysis, data interpretation or writing of the study.

## Results

### Spectral flow screening reveals a distinct CCR5-associated effector phenotype for Th17.1 cells

**Fig. 1:**
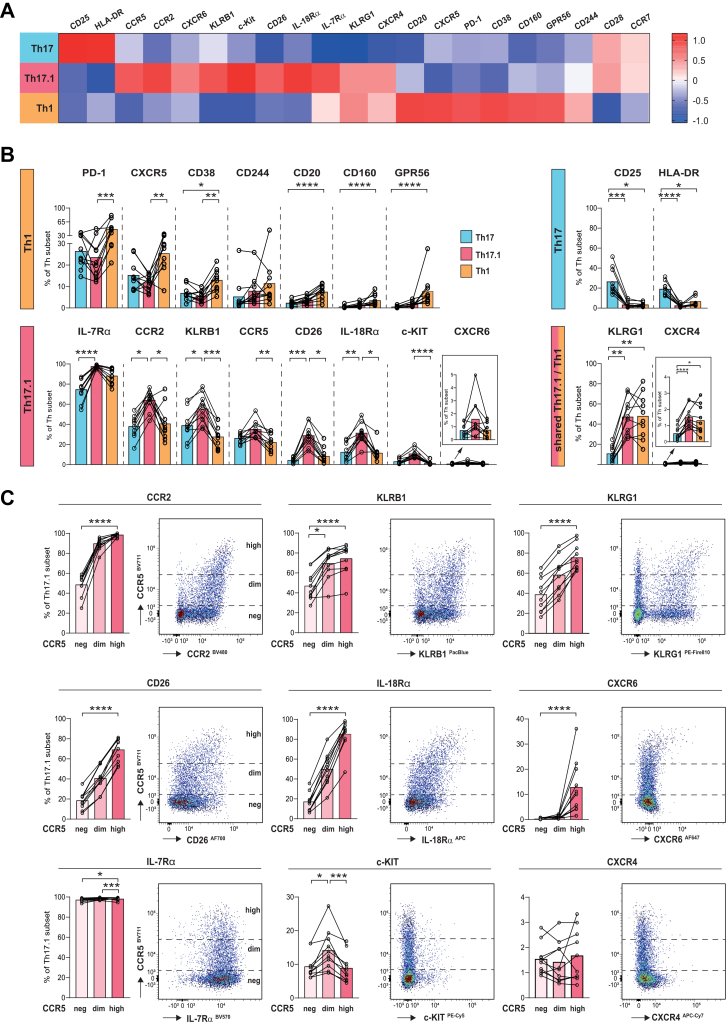
**Comprehensive spectral flow cytometric analysis of Th17, Th17.1 and Th1 cells in healthy blood.** (A) Heatmap showing normalised frequencies (z-scores) of surface markers on circulating Th17 (CCR6^+^CXCR3^−^CCR4^+^), Th17.1 (CCR6^+^CXCR3^+^CCR4^-^) and Th1 (CCR6^−^CXCR3^+^CCR4^-^) cells. (B) Relative frequencies of cells positive for different effector molecules within circulating Th17, Th17.1 and Th1. (C) Relative frequencies and representative dot plots of effector molecules expressed by CCR5^-^, CCR5^dim^ and CCR5^high^ Th17.1 cells. Each dot in the graphs represents a single individual. n = 10 per sample for each subset. Data were analysed using Friedman tests with Dunn's post hoc analysis (B–C). ∗P ≤ 0.05, ∗∗P ≤ 0.01, ∗∗∗P ≤ 0.001, ∗∗∗∗P ≤ 0.0001.

To explore the phenotypic characteristics and diversity of Th17.1 cells, we analysed healthy donor PBMCs using an in-house designed 37-parameter spectral flow cytometry panel including T effector cell-defining surface markers. Comparative analysis of Th17.1 (CCR6^+^CXCR3^+^CCR4^–^, Th17 (CCR6^+^CXCR3^–^CCR4^+^), and Th1 (CCR6^–^CXCR3^+^CCR4^–^) cells revealed different effector profiles (Fig. 1A). Th17 cells expressed relatively higher levels of CD25 and HLA-DR, whereas Th1 cells were more positive for PD-1, CXCR5, CD38, CD244, CD20, CD160 and GPR56 as compared to Th17 and/or Th17.1 cells (Fig. 1B). Th17.1 cells, in turn, consisted of significantly more fractions expressing CCR2, CXCR6, KLRB1, c-KIT, CD26, IL-18Rα, IL-7Rα and CCR5 than both Th17 and Th1 cells (Fig. 1B). KLRG1 and CXCR4 were equally expressed by Th1 and Th17.1, which was higher than for Th17 cells (Fig. 1B). We next examined its expression together with other surface markers upregulated on Th17.1 cells. Notably, within the Th17.1 population, CCR5^high^ cells were enriched for CCR2, KLRB1, KLRG1, CD26, IL-18Rα, and CXCR6 as compared with CCR5^dim^ and CCR5^neg^ fractions, while showing no increase in c-KIT, IL-7Rα, or CXCR4 expression (Fig. 1C). These trends were even more pronounced when assessing the MFI of these markers within the different CCR5 Th17.1 subsets (Supplementary Fig. S2) Taken together, these results not only support the fact that Th17.1 shares features with both Th1 and Th17 cells, but also reveal an exclusive marker profile for Th17.1 cells that is defined by increased CCR5 expression. In the context of MS, this points towards a subset within circulating Th17.1 cells that has its own effector program with a propensity to enter the CNS.

### The effector phenotype of CCR5^high^ Th17.1 cells is more pronounced in the CSF of people with MS

**Fig. 2:**
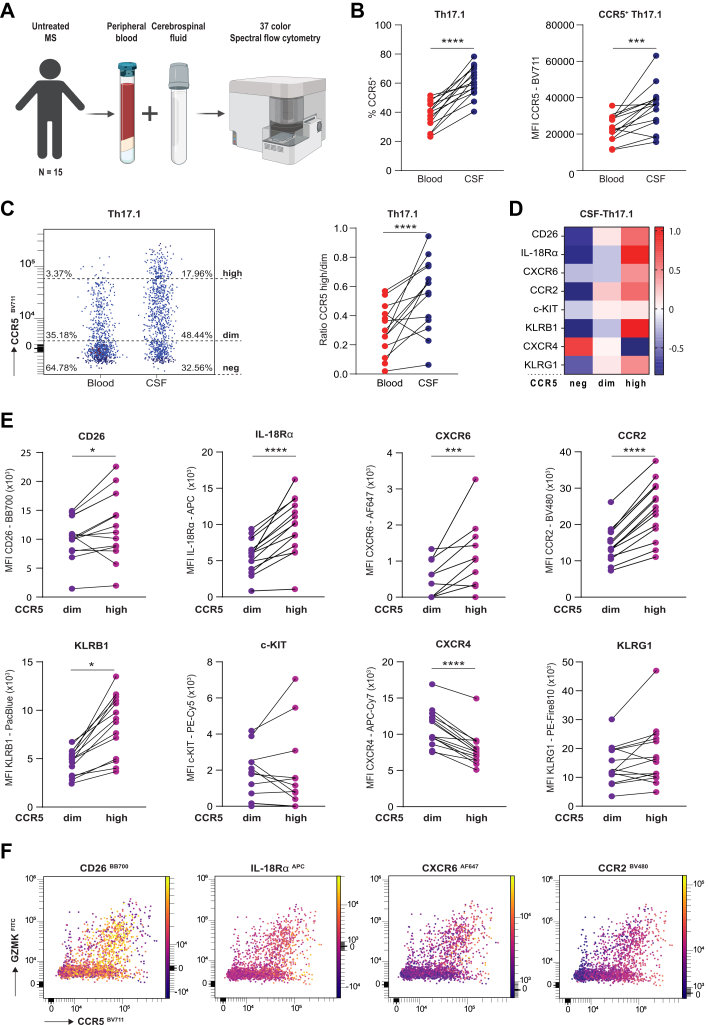
**Differential expression of CCR5 and associated surface markers on Th17.1 cells in peripheral blood and CSF of people with MS.** (A) Experimental setup. (B) Relative frequencies of CCR5^+^ cells within Th17.1 in paired peripheral blood (PB) and cerebrospinal fluid (CSF; *left*); Mean fluorescent Intensity (MFI) of CCR5 within CCR5^+^ Th17.1 cells in paired PB and CSF from pwMS (*right*). (C) Representative example of CCR5 expression within PB and CSF Th17.1 cells (*left*) and ratio of CCR5^high^ and CCR5^dim^ fractions within Th17.1 cells in paired PB and CSF (*right*). (D) Heatmap showing normalised frequencies (Z-scores) of surface markers on CSF Th17.1 cells. (E) MFI of CD26, IL-18Rα, CXCR6, CCR2, KLRB1, c-KIT, CXCR4 and KLRG1 expression on CCR5^dim^ versus CCR5^high^ Th17.1 cells in the CSF. (F) Representative example of CD26, IL-18Rα, CXCR6 and CCR2 in conjunction with CCR5 and granzyme K expression levels in Th17.1 cells. Each dot in the graphs represents a single individual. n = 14–15 samples for paired PB and CSF data. Data were analysed using Wilcoxon singed-rank tests (B–C, E) and obtained from fresh cells of people with MS. ∗P ≤ 0.05, ∗∗∗P ≤ 0.001, ∗∗∗∗P ≤ 0.0001.

To assess if the identified CCR5-associated effector profile of Th17.1 cells supports their preferential CNS-homing capacity in MS, we used our spectral flow cytometry panel to analyse 15 paired CSF and blood samples from people with MS without influence of any immunomodulatory treatment at sampling (Table 1; Fig. 2A; gating strategy in Supplementary Fig. S3). Both the frequency and intensity of CCR5 expression was on average 1.6 and 1.5-fold higher for CSF-derived Th17.1 cells as compared to those from the blood (Fig. 2B). This was underlined by the on average 2-fold elevated ratio of CCR5^high^ versus CCR5^dim^ fractions within CSF Th17.1 cells (Fig. 2C). In line with our findings in healthy blood (Fig. 1C), CCR5^high^ Th17.1 cells that were enriched in the CSF showed high levels of associated surface markers, particularly CCR2, CXCR6, KLRB1, CD26 and IL-18Rα (Fig. 2D and E). This was not the case for c-KIT, IL-7Rα and CXCR4, of which the latter was even less expressed on the CCR5^high^ fraction. Finally, we verified that intracellular protein GZMK is part of the identified CCR5-associated cluster within Th17.1 cells (Fig. 2F), which likely contributes to an effector state that enables it to cross a brain endothelial layer already under non-inflammatory conditions.

### Natalizumab reduces CCR5^high^ Th17.1 cells with a highly receptive and pre-cytotoxic state in MS

**Fig. 3:**
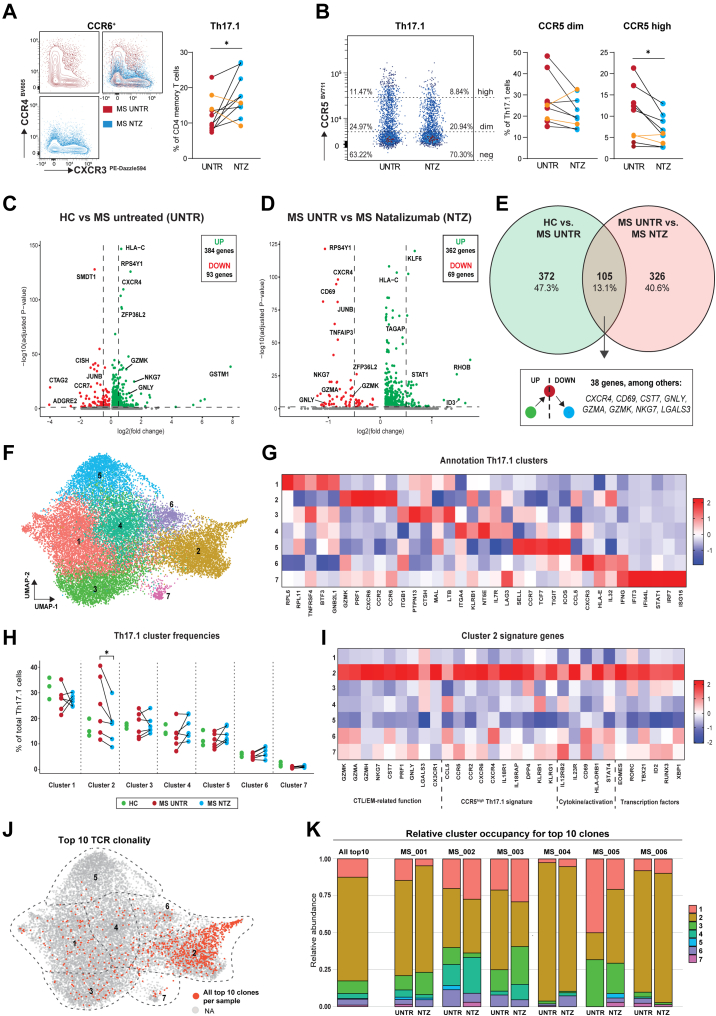
**Single cell analyses of circulating Th17.1 cells in people with MS before and after natalizumab treatment.** (A) Representative contour plots and overlap of CXCR3 versus CCR4 expression in CCR6^+^ CD4+ memory T cells (*left*) highlighting the relatively increased frequency of Th17.1 cells in the MS NTZ (blue) versus MS UNTR (red) group (*right*). (B) Representative example of CCR5 expression on Th17.1 cells of the MS UNTR and MS NTZ group (*left*) and relative frequencies of CCR5^dim^ (*middle*) and CCR5^high^ (*right*) cells within Th17.1. The orange samples showed decreased frequencies of Th17.1 cells after NTZ treatment. (C (C, D) Volcano plots showing upregulated (green) and downregulated (red) genes in healthy controls (HC) versus untreated MS patients (MS UNTR; C) and untreated versus natalizumab-treated MS (MS NTZ) patients (D). (E) Venn diagram showing overlapping counts of genes differentially expressed between the groups. (F) Uniform Manifold Approximation and Projection (UMAP) showing 7 clusters of Th17.1 cells. (G) Heatmap showing relative expression levels (Z-scores) of genes used for the annotation of Th17.1 clusters. (H) Relative frequencies of Th17.1 cells per cluster for HC as well as paired MS UNTR and MS NTZ groups. (I) Heatmap depicting biological relevant and significantly different genes for cluster 2. (J) UMAP showing 7 clusters with in red top 10 clones for all MS samples. (K) Quantification of relative abundance of top 10 clones across 7 clusters, for all MS samples combined and split per sample. n = 6–9 samples for paired UNTR-NTZ samples, n = 3 for healthy controls (HC). Data were analysed using Wilcoxon signed-rank test. ∗P ≤ 0.05.

To further study the effector state of this Th17.1 subset and understand how this is associated with MS, we first analysed paired blood samples of people with MS before (MS UNTR) and after natalizumab (NTZ) treatment (n = 9). This treatment prevents immune cell entrance into the CNS and effectively suppresses MS disease activity. Besides confirming the entrapment of Th17.1 cells within the blood (Fig. 3A), we found that the CCR5^high^ fraction within Th17.1 cells was on average 0.63-fold diminished the most following NTZ treatment (Fig. 3B). This was most pronounced for pre-treatment samples with a relatively large number of CCR5^high^ cells within the Th17.1 and not the case for either Th1 or Th17 cells and CCR5^+^ subsets therein (see Supplementary Fig. S4). Complementary to this, we performed in-depth single-cell transcriptomics for Th17.1 cells that were enriched from the same blood samples in order to have a close look into gene expression profiles and cell states (Supplementary Fig. S5A). First, we performed differential expression (DE) analysis on Th17.1 cells and identified 477 DE genes of which 384 and 93 were up- and downregulated in MS UNTR compared to HC (Fig. 3C and Supplementary Table S3). When comparing MS UNTR to MS NTZ, we observed 431 DE genes of which 362 and 69 were up- and downregulated, respectively (Fig. 3D and Supplementary Table S4). Furthermore, we identified 105 transcripts that were both associated with MS and influenced by NTZ treatment (Fig. 3E and Supplementary Table S5). Thirty eight of these were lower expressed in MS NTZ and HC as compared to MS UNTR, of which many relate to an effector memory and cytotoxic function of T cells (e.g. *CST7*, *GNLY*, *GZMA*, *NKG7*; Fig. 3E). Additionally, we performed clustering and dimension reduction analysis to assess whether these expression differences indeed reflect specific cell identities. This type of analysis yielded 7 distinct Th17.1 subpopulations (Fig. 3F) each defined by unique gene expression profiles (Fig. 3G; Supplementary Table S6). Notably, cluster 2 was enriched for *CCR5* and CCR5-related transcripts such as *CCR2*, *CXCR6* and *GZMK*, which supports our Th17.1 signature effector phenotype identified in blood and CSF using spectral flow cytometry (Fig. 1, Fig. 2). Of all the identified Th17.1 clusters, only cluster 2 was significantly decreased on average 0.75 fold change after NTZ treatment (Fig. 3H). In depth analysis on the identity of cluster 2 revealed an elevated expression of genes involved in cytotoxicity (e.g. *GZMA*, *PRF1*, *CX3CR1*), cytokine signalling (e.g. *IL12RB2*, *IL18R1*) and other markers that we earlier identified on protein level (e.g. *CXCR6*, *DPP4* [CD26], *KLRG1*) (Fig. 3I).

Next to subset analysis, we performed TCR clonality analysis and found that top clonotypes for all individual MS patients predominantly resided in the NTZ-targeted cluster 2 (Fig. 3J and K and Supplementary Fig. S5B). Within cluster 2, we did not find differences in the number of expanded clones when comparing untreated and NTZ-treated samples (Supplementary Fig. S5C and D).Using VDJdb as a reference database and stringdist package for distance calculation, none of these clones contained CDR3 regions that matched with sequences known to recognise a specific epitope. Taken together, these independent findings highlight a CCR5^high^ subset of Th17.1 cells that is relevant for MS and has increased ability to gain cytotoxic properties.

### Th17.1 cells gain a CCR5-associated effector profile via preferential IL-12 and IL-18 response

**Fig. 4:**
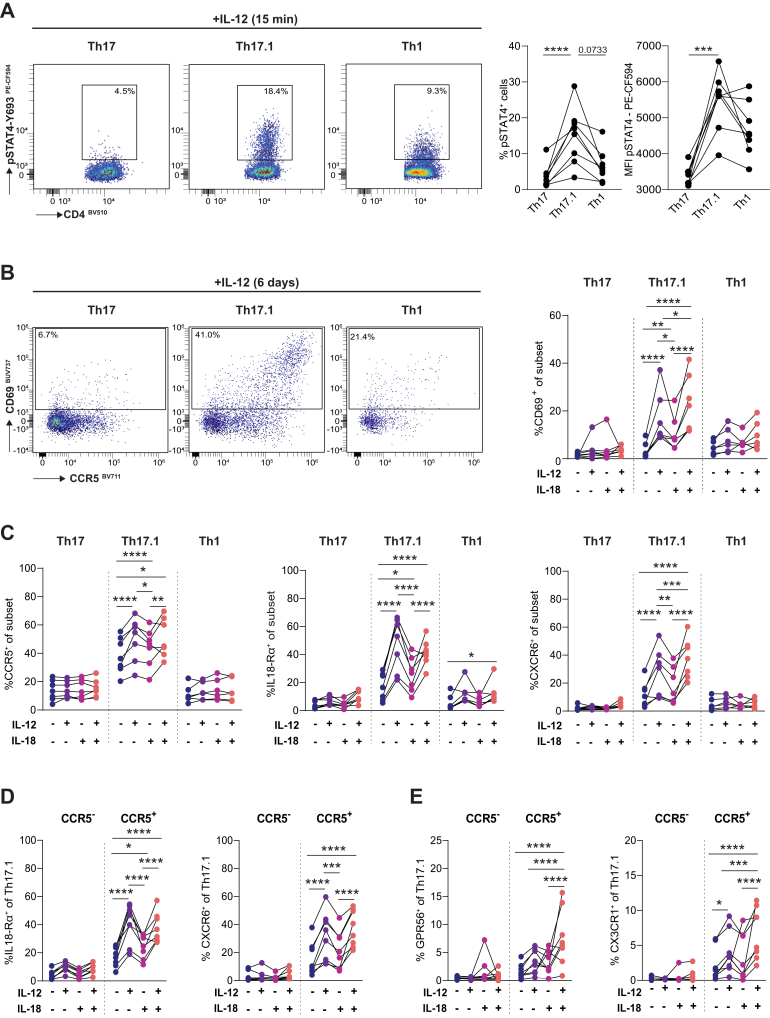
**Differential impact of IL-12 and/or IL-18 on the activation and effector profile of Th17.1 cells *in vitro*.** (A) Representative dot plots (*left*) and quantification of pSTAT4-positive cells (*middle*) and pSTAT4 MFI (*right*) in Th17, Th17.1 and Th1 fractions that were stimulated with IL-12 for 15 min. (B) Representative dot plots (*left*) and quantification (*right*) of CD69-positive cells inTh17, Th17.1 and Th1 fractions that were stimulated with IL-12 and/or IL-18 for 6 days. (C) Relative frequencies of CCR5, IL-18Rα or CXCR6 expressing cells within Th17, Th17.1 or Th1 fractions stimulated with or without IL-12 and/or IL-18. (D, E) Relative frequencies of cells expressing IL-18Rα (D; *left*), CXCR6 (D; *right*), GPR56 (E; *left*) and CX3CR1 (E; *right*) within CCR5^-^ or CCR5^+^ Th17.1 fractions that were stimulated with or without IL-12 and/or IL-18 for 6 days. Each dot in the graphs represents a single individual. n = 6–8 samples per subset. Data were analysed using Friedman test with Dunn's post hoc analysis (A), 2-way ANOVA with post-hoc Tukey (B–E) and obtained with purified subsets from thawed PBMCs of healthy donors.∗P ≤ 0.05, ∗∗P ≤ 0.01, ∗∗∗P ≤ 0.001, ∗∗∗∗P ≤ 0.0001.

We next addressed how this CCR5^high^ subset of Th17.1 cells differentially responds to external triggers and if it can acquire such a cytotoxic phenotype *in vitro*. IL-12 and IL-18 were of particular interest, as genes for these receptors were also expressed on RNA level in cytotoxic-like Th17.1 cells. First of all, after short-term stimulation with IL-12, Th17.1 showed increased phosphorylation of downstream molecule STAT4 as compared to Th17 and Th1 derived from the same blood donors. This was based on both the frequency of pSTAT4-positive cells and pSTAT4 levels on these positive cells (Fig. 4A). In line with that, especially CCR5^+^ Th17.1 cells was more activated after 6 days of IL-12 stimulation, as determined by CD69 surface expression (Fig. 4B, Supplementary Fig. S6). Since IL-18Rα expression is part of its signature (Fig. 1, Fig. 2, Fig. 3), we analysed how IL-12 interacts with IL-18 to polarise Th17.1 cells towards a CCR5-associated effector profile. In contrast to IL-18, IL-12 induced the expression of CCR5 and IL-18Rα on Th17.1 (1.3-fold and 2.7-fold, respectively), which was hardly seen for Th17 and Th1 cells (Fig. 4C). The same was true for CXCR6 (2.3-fold upregulation), although this core signature molecule was on average 3.2-fold upregulated in the presence of both IL-12 and IL-18 (Fig. 4C). In addition to the increased response of the CCR5-positive fraction of Th17.1 to these cytokines (Fig. 4D, Supplementary Fig. S6A), we found that the expression of two cytotoxicity-related surface molecules, GPR56 and CX3CR1, was mostly induced in this particular subset when both IL-12 and IL-18 were added to the cultures (1.4-fold and 3.2-fold, respectively; Fig. 4E). This was not the case for cultures with Th1 cells and to a lesser extend in Th17 cells (Supplementary Fig. S6B and C). These findings suggest that the CCR5^+^ subset within Th17.1 selectively responds to IL-12 and IL-18 to become a cytotoxic CD4^+^ T cell.

*Ex vivo*, we could not detect IL-18 in the CSF (Supplementary Fig. S7A) of MS patients. IL-18 levels in the blood were not affected by NTZ therapy (Supplementary Fig. S7B), but did associate with the presence of Th17.1 cells in both MS blood and CSF (Supplementary Fig. S7C). The latter at least supports the potential of Th17.1 cells to respond to IL-18 as result of IL-12-induced IL-18Rα expression (see Figs. 1B and 4C), thereby possibly promoting their survival and proliferation as well.

### IL-12 and IL-18 synergistically drive secretion of cytolytic proteins by CCR5^+^ Th17.1 cells

**Fig. 5:**
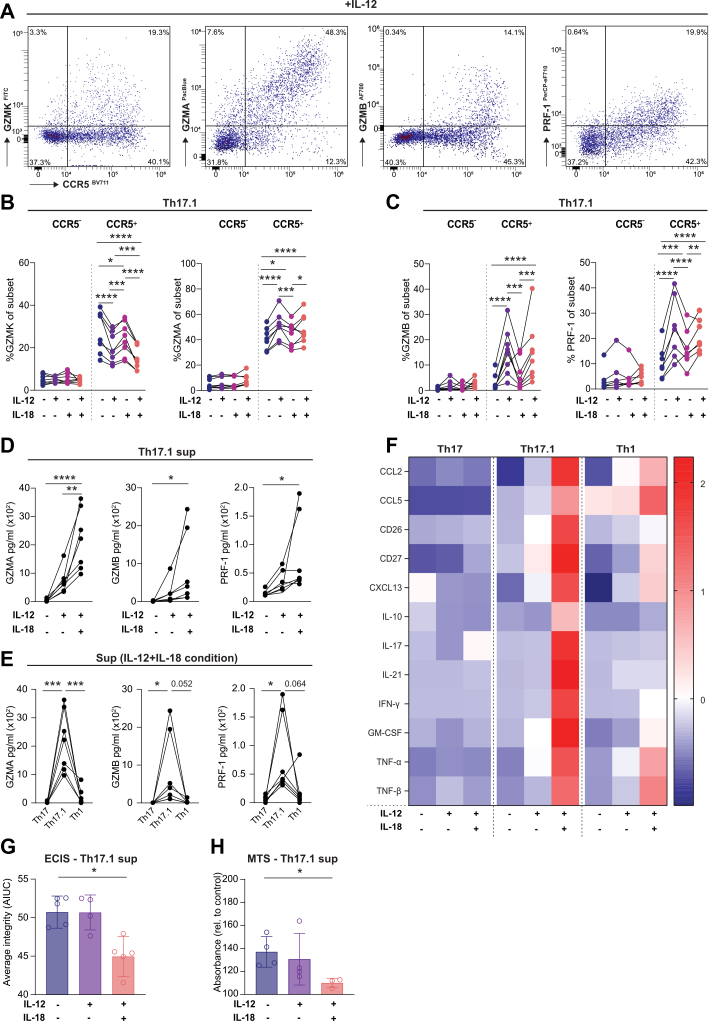
**IL-12 and IL-18 work together to stimulate the expression and secretion of cytotoxic molecules by Th17.1 cells.** (A) Representative dot plots of CCR5 versus granzyme K (GZMK), granzyme A (GZMA), granzyme B (GZMB) and perforin (PRF) expression (B–C) and quantification of relative frequencies of cells positive for GZMK, GZMA, GZMB and PRF within CCR5^-^ or CCR5^+^ Th17.1 fractions that were stimulated with or without IL-12 and/or IL-18 for 6 days. (D) The impact of IL-12 and/or IL-18 on the levels of GZMA (*left*), GZMB (*middle*) and PRF (*right*) in pg/ml within the supernatant from Th17.1 fractions after 6 days of stimulation. (E) Differences in GZMA (*left*), GZMB (*middle*) and PRF (*right*) levels between the supernatant of Th17, Th17.1 and Th1 fractions stimulated with both IL-12 and IL-18 for 6 days. (F) Heatmap showing normalised levels (Z-scores) of excreted inflammatory molecules in the supernatant of Th17, Th17.1 and Th1 fractions stimulated with or without IL-12 and IL-18 for 6 days, as determined by Luminex. (G) The relative integrity of cultured human derived brain endothelial cells (hCMEC/D3) with Th17.1 cell culture supernatants as measured using electrical cell-substrate impedance spectroscopy (ECIS). (H) Relative hCMEC/D3 cell viability as measured by MTS assays after culturing with Th17.1 cell culture supernatants. Each dot in the graphs represents a single individual. n = 4–8 samples per subset. Data were analysed using 2-way ANOVA with post hoc Tukey's (B–E) or Kruskal–Wallis post hoc Dunn (G–H) and obtained with purified subsets from thawed PBMCs of healthy donors.∗P ≤ 0.05, ∗∗P ≤ 0.01, ∗∗∗P ≤ 0.001, ∗∗∗∗P ≤ 0.0001.

When stimulated with IL-12 for 6 days, we found that GZMK was downregulated and GZMA together with GZMB and PRF were strongly upregulated in CCR5^+^ Th17.1 cells (1.3-fold, 5.1-fold and 1.2-fold, respectively; Fig. 5A–C). These effects were not seen in their CCR5^-^ counterparts (Fig. 5A–C). Besides for an extra reduction in GZMK expression, the addition of IL-18 to these cultures did not further increase intracellular expression of GZMA, GZMB and PRF in the CCR5^+^ Th17.1 subset (Fig. 5B and C). However, when we analysed the supernatants from the same cultures, we did see an additional effect of IL-18 on the levels of cytolytic proteins secreted by Th17.1 cells (Fig. 5D). This synergistic effect of IL-12 and IL-18 on the secretion of GZMA, GZMB and PRF was by far the strongest for Th17.1 as compared with Th17 and Th1 cells (Fig. 5E and F). The addition of CCL5 ligands CCL4 and CCL5 did induce such a cytotoxic-like phenotype *in vitro* (Supplementary Fig. S8).

**Fig. 6:**
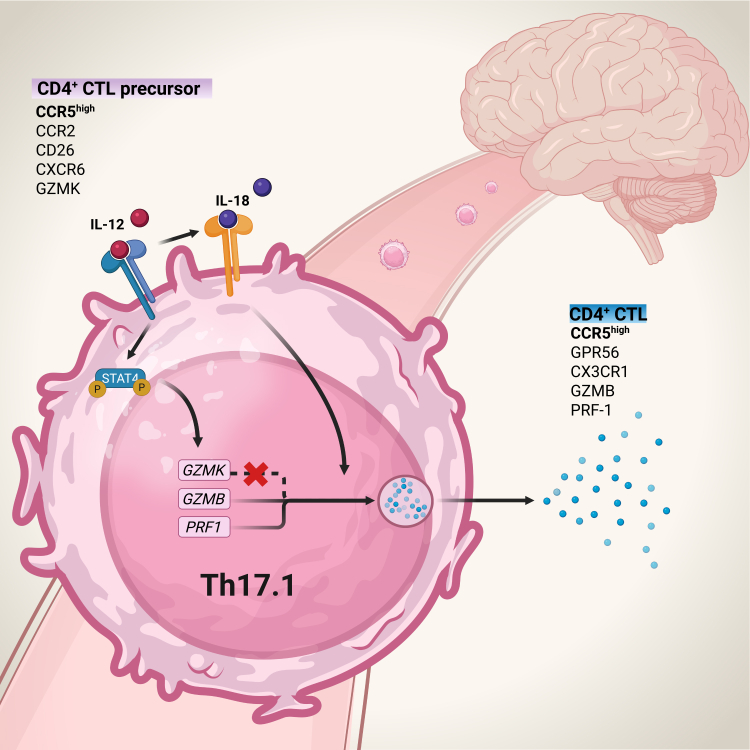
CCR5^high^**Th17.1 cells as** brain-homing cytotoxic precursors **in MS.** IL-12 stimulation induces the phosphorylation of downstream molecule pSTAT4 and the upregulation of the IL-18R. IL-12 and IL-18 stimulation result in increased intracellular expression of GZMB and PRF but not GZMK. The combination of IL-12 and IL-18 stimulation resulted in the excretion of cytolytic mediators PRF and GZMB, but also upregulation of CTL associated surface markers GPR56 and CX3CR1.

Finally, to determine if this only accounted for cytolytic proteins, we also compared the presence of other inflammatory molecules in these supernatants using a multiplex Luminex assay, including CCL2, CCL5, CD26, CD27, CXCL13, IL-10, IL-17, IL-21, IFN-γ, GM-CSF, TNF-α and TNF-β (Fig. 5F). We found that for Th17.1 cells, all these molecules were released mostly under both IL-12 and IL-18-stimulating conditions. This was hardly the case for Th17 cells. For Th1, only the release of CCL5 was more induced than for Th17.1 cells under these conditions. As expected, we did not detect IL-17 in Th1-derived supernatants, thereby serving as a negative control. Hence, these data implicate that CCR5^+^ Th17.1 cells recognise IL-12 to trigger intracellular expression and IL-18 to ensure the release of cytolytic proteins (see also Fig. 6).

Finally, to assess the impact of the IL-12- and IL-18-induced cytotoxic phenotype of Th17.1 cells, we added supernatants from these cell cultures to human brain-derived endothelial monolayers (hCMEC/D3) and analysed the effect on barrier permeability. Using ECIS, we only observed a significant reduction in monolayer integrity following exposure to supernatants from Th17.1 cells that were stimulated with both IL-12 and IL-18 (Fig. 5G; barrier integrity 20% < control). A similar decrease was detected in endothelial cell viability under these conditions using an MTS assay (Fig. 5H). This was not seen for Th17 cells under the same conditions (Supplementary Fig. S9). These findings further support the increased ability of Th17.1 cells to respond to IL-12 and IL-18 and thereby break through the blood–brain barrier *in vitro*.

## Discussion

In this study, we have used an integrated single-cell profiling approach to identify features that discriminate a low-frequency circulating human T cell subset termed Th17.1 from other pro-inflammatory CD4^+^ T cells in the context of MS. Based on high-dimensional spectral flow cytometry, single-cell transcriptomics and *in vitro* stimulation assays for carefully defined subsets, we reveal a unique MS-associated CCR5^high^ cluster within Th17.1 cells with the propensity to gain a cytotoxic phenotype and enter the CNS. Its selective enrichment in the CSF and corresponding suppression by high efficacy drug natalizumab positions the CCR5^high^ Th17.1 cell as a circulating CCR5^high^ CD4^+^ CTL precursor that can be further assessed for its role within the inflamed CNS and use to earlier detect and more accurately monitor treatment responses in MS.

The enrichment of CCR5^high^ Th17.1 cells in the CSF suggests an enhanced ability to cross the blood–brain barrier,^23^^,^^24^ which is supported by the increased levels of CCR5 ligands (e.g. CCL5, CCL3) in CSF of pwMS^25^^,^^26^ and results from previous *in vitro* studies.^8^^,^^9^ The functional relevance of CCR5 has been further demonstrated in studies using CCR5 knockout mice, which revealed reduced CNS trafficking and pathogenicity of CD4^+^ T cells during a viral infection.^27^ The expression of CCR5 could possibly be a common trait of CD4^+^ CNS migration, since this chemokine receptor was also found to be highly expressed on CD4^+^ and CD8^+^ T cells isolated from post-mortem brain tissue.^9^^,^^28^, ^29^, ^30^ Migration towards a CXCL10 gradient over a brain microvascular endothelium layer associated with increased CCR5 expression on CD4^+^ but lower expression of CCR5 on CD8^+^ T cells, which indicates a different association of CCR5 with CXCR3-mediated migration between these T cell types.^30^

Following NTZ treatment, the frequency of CCR5^high^ Th17.1 cluster selectively declined as determined by transcriptomic and protein levels. In contrast, a recent study by Herich et al. showed an increase in CD4^+^ CCR5^high^ GZMK ^+^ T cells after NTZ therapy. This however, is likely due to the fact that we looked into a more defined subset.^9^ Although Th17.1 cells accumulate in the blood after NTZ as previously shown,^4^^,^^5^ our recent findings indicate a phenotypic shift which could also be due to a reduced overall cytotoxic/pathogenic state of the circulating Th17.1 compartment.^31^ Notably, despite expression of *GZMA* and *PRF1* mRNA, the corresponding proteins are not abundantly detected in *ex vivo* Th17.1. This suggests that a subset of circulating Th17.1 cells with a pre-cytotoxic state that may require additional stimulation to acquire full cytotoxic function.

We showed that CCR5^high^ Th17.1 cells have a distinct profile on both protein and transcript level. CD26 and CXCR6 is co-expressed in circulating Th17.1 cells, which links to previous work regarding cytotoxic CD4^+^ T cell phenotypes.^9^^,^^32^ Interestingly, CD26 is known to cleave a myriad of proteins including CCR5 ligand CCL5 (also known as RANTES),^33^ which is found enriched in CSF and brain tissue of pwMS during active disease.^34^ This cleavage results in an isoform with deceased binding activity to its other receptors (CCR1 and CCR3), but a moderately increased activity with CCR5^35^ and therefore promoting specific CNS trafficking of CCR5^+^ T cells. The expression of CXCR6 might also promote CNS trafficking since its ligand CXCL16 is known to be expressed on inflamed astrocytes and glial cells in the CNS during neuroinflammation and MS.^36^, ^37^, ^38^

Furthermore, we showed that CCR5^high^ Th17.1 cells strongly respond to both IL-12 and IL-18 to acquire a cytotoxic effector function. IL-12 seems to be crucial for the expression, while IL-18 renders the secretion of cytolytic proteins by Th17.1 cells. This is of particular interest as there are several MS risk SNPs located in or near genes associated with the IL-12 signalling cascade, including *TYK2*, *SH2B3* and *STAT4*.^39^ Accordingly, the expression of cytotoxicity-related surface proteins GPR56^40^ and CX3CR1^41^ were only seen after combined IL-12 and IL-18 stimulation. Th17.1 cells encountering and communicating with IL-12- and IL-18-producing cells likely corresponds to key events in MS pathogenesis. Infection with the Epstein–Barr Virus (EBV), one of the major MS environmental risk factors,^42^ induces IL-12 and IL-18 production by B cells, which may skew interacting T cells towards a cytotoxic Th17.1 phenotype.^43^, ^44^, ^45^, ^46^, ^47^ EBV-derived microRNAs can further suppress cytokine production, including IL-12.^48^ If EBV indeed promotes Th17.1 polarisation, this effect likely occurs during primary infection, typically years before MS onset,^42^^,^^46^ making it particularly challenging to pinpoint EBV-infected B cells as the definitive cytokine source. During their journey into the CNS, brain-homing T cells encounter the BBB where endothelial-derived IL-18 was shown to further expand CCR5^+^CCR2^+^ T peripheral helper-like cells,^49^ a subset that closely resembles Th17.1 phenotypically. At first stages of lesion formation within the brain of pwMS, myeloid cells were shown to express both IL-18 and CXCL16.^50^^,^^51^ Although not touched upon in this study, this may comprise a niche that attracts CXCR6^+^ Th17.1 cells and promotes their cytotoxic activity through IL-18R signalling. Preferential recruitment of Th17.1 cells to the MS brain was shown by our group previously.^5^ Accordingly, a recent study showed enrichment of CXCR6^+^ CD4^+^ T cells with a tissue-residency phenotype in post-mortem MS normal appearing white matter and lesions as well as the brain tissue of experimental autoimmune encephalomyelitis (EAE) mice.^52^ When both CNS-resident CXCR6^+^ and recirculating CD4^+^ T cells were depleted, ongoing neuroinflammation in EAE was abolished, supporting a key role of CXCR6^+^ CD4^+^ T-cell fractions in the maintenance of chronic neuroinflammation. Since we did find a positive correlation between IL-18 levels in the blood and the presence of Th17.1 cells in the CSF, we cannot rule out that IL-18 also boosts their skewing towards a CTL systemically.

A strength of this study is that we performed deep phenotyping as well as enriched for carefully defined and low-frequency *ex vivo* Th17.1 cells from the blood of the same people with MS before and after natalizumab treatment in order to assess their transcriptome at the single cell level. Also by separately purifying Th17.1, Th17 and Th1 cells from the blood for *in vitro* analyses using spectral flow cytometry, we prevented any bias that may occur when using total memory CD4^+^ T cells and gating for these subsets after culture. While we show enrichment of CCR5^high^ Th17.1 cells in the CSF, we lack direct evidence of their presence, antigen specificity, functional activity and crosstalk with other cells near active MS lesions. To study this, signature markers of Th17.1 cells should be analysed both *ex vivo* and *in situ* in post-mortem brain tissues from MS donors in future studies.

In summary, our study reveals a low-frequency circulating CCR5^high^ Th17.1 subset with cytotoxic potential and implications for early contribution to MS. To verify and elaborate on their pathogenicity, future studies should focus on the presence, clonality and features of CCR5^high^ Th17.1 cells in different CNS tissues of people with MS, their co-existence and direct interaction with B cells and microglia, as well as their specific response to EBV and candidate target antigens related to MS. The distinctive CCR5 signature of these cells could yield opportunities for early detection, monitoring of disease activity upon disease-modifying treatment and the development of more refined therapeutic approaches in MS.

## Contributors

F.v.P., J.R., J.S. and M.M.v.L. contributed to the study concept. F.v.P., J.R., Y.v.H., A.F.W.W., S.S., M.-J.M, E.M.B., H.J.G.v.d.W., W.A.D., C.J.M.v.A., H.E.d.V. and A.C.P acquired and analysed data. F.v.P., J.R., Y.v.H and C.J.M.v.A. accessed and verified the underlying data. R.A.M.K.K., J.d.B., I.S. B.H.W. and J.S. collected samples and clinical data. F.v.P. and J.R. drafted figures. F.v.P., J.R., J.S., and M.M.v.L. verified and interpreted the underlying data and wrote the manuscript. J.S. and M.M.v.L. designed the research, obtained funding, discussed results and supervised the project team. All authors reviewed and approved the manuscript for final publication.

## Data sharing statement

Data supporting our findings will be made available by the corresponding author upon reasonable request, beginning at publication with no end date. Single-cell RNAseq data are available at the DataverseNL platform (see Data availability in the Methods section).

## Declaration of interests

J. de Beukelaar reports leadership positions in the Dutch Society of Neurologists, for the Dutch MS working group (board member) and Dutch MS Registry (board member). J. Smolders reports grants for scientific research from Biogen, Roche, Siemens Healthineers and Hansa Biopharma, and has received speaker and/or consultancy fees from Biogen, Merck, Novartis, Roche and Sanofi and reports leadership roles in the Dutch Society for Neurology (chair guideline committee), Stichting MS Research (scientific advisory board), International Progressive MS Alliance (scientific steering committee) and ECTRIMS counsel (delegate). M.M. van Luijn received research support from EMD Serono, Novartis, GSK, Idorsia Pharmaceutical Ltd, and the healthcare business of Merck KGaA. The remaining authors have no conflicts of interest to declare.

## Appendix A Supplementary data

Supplementary Figures and Tables

Cell-Line-Checklist

Validation document_hCMECD3
