# Supplementary material for: Refined single-cell profiling captures a CCR5high CD4+ cytotoxic T-cell precursor in multiple sclerosis
Source: eBioMedicine. 2026 Jun 6;129:106324. doi: 10.1016/j.ebiom.2026.106324 (PMC13264205; doi:10.1016/j.ebiom.2026.106324)
Supplement: Supplementary Figures and Tables [file mmc1.pdf]

Supplementary material to:

Refined single-cell profiling captures a CCR5^{high} CD4^{+} cytotoxic T-cell precursor in multiple sclerosis

Contents list:

Supplementary Figure 1-9

Supplementary Table 1-6

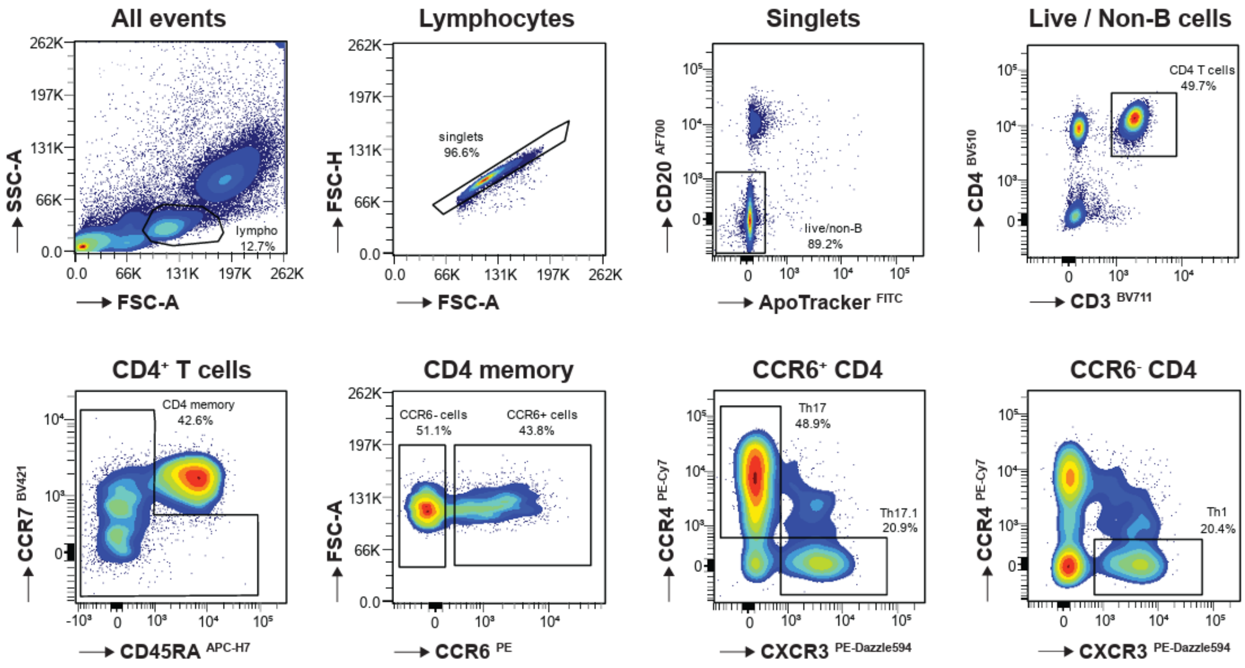

Supplemental figure 1: Gating strategy used for sorting Th1, Th17 and Th17.1 cells from the blood.

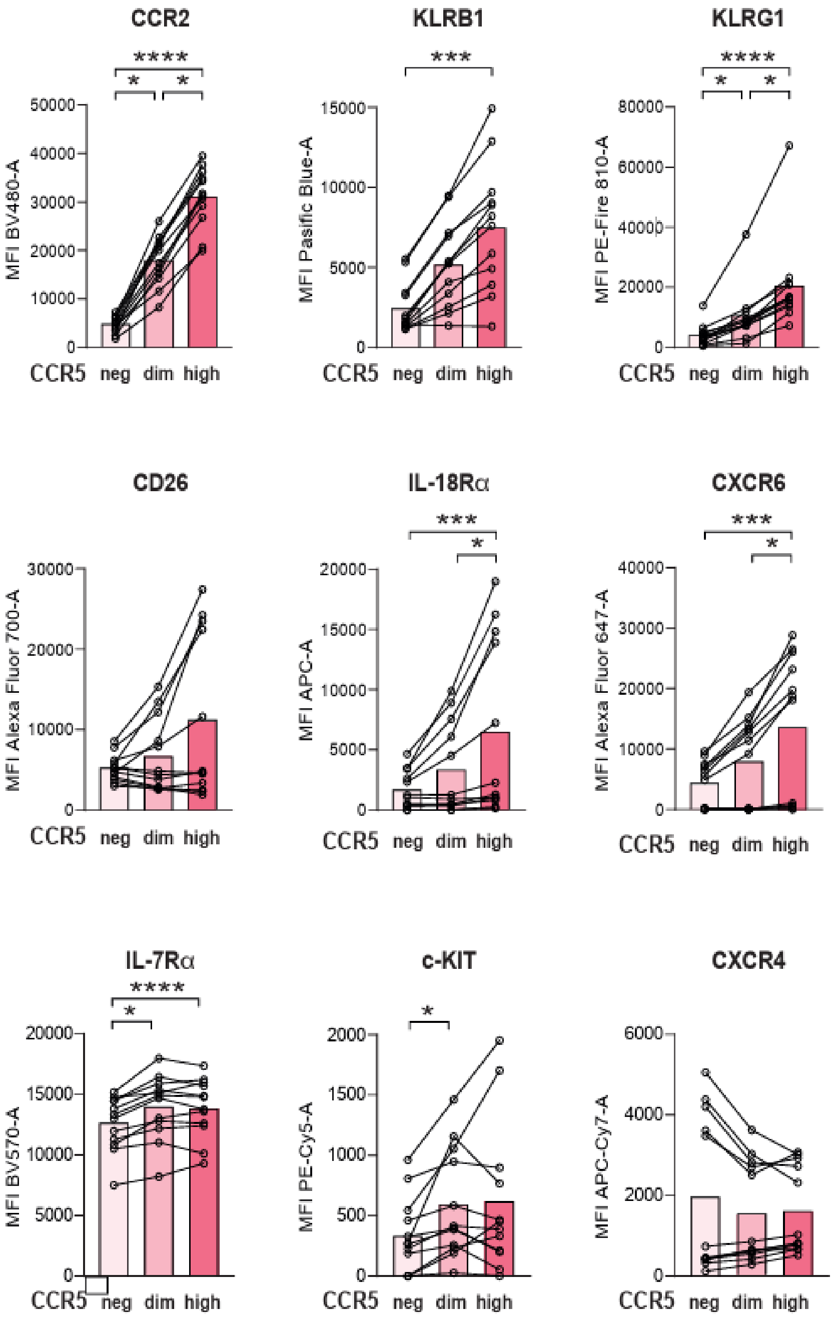

Supplemental figure 2: MFI of Th17.1 signature markers in CCR5 neg, dim and high populations. Mean fluorescence intensity (MFI) effector molecules expressed by $CCR5^-$, $CCR5^{dim}$ and $CCR5^{high}$ Th17.1 cells. Each dot in the graphs represents a single individual. Data were analysed using Friedman tests with Dunn's post hoc analysis (B-C). *P ≤ 0.05, ***P ≤ 0.001, ****P ≤ 0.0001.

A

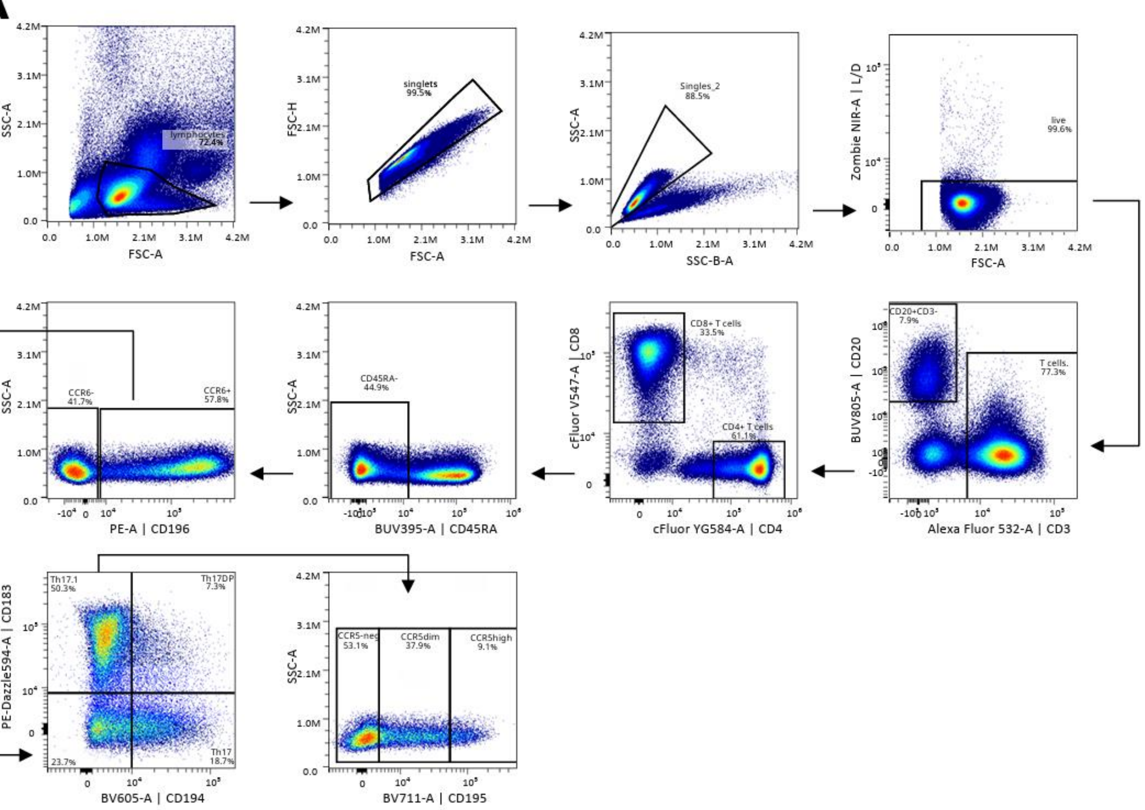
SSC-A
FSC-A
lymphocytes
72.4%
FSC-H
singlets
99.5%
Singles_2
88.5%
SSC-B-A
Zombie NIR-A | L/D
live
99.6%
BUV805-A | CD20
CD20+CD3-
7.9%
T cells.
77.3%
Alexa Fluor 532-A | CD3
cFluor V547-A | CD8
CD8+ T cells
33.5%
CD4+ T cells
61.1%
cFluor YG584-A | CD4
CD45RA-
44.9%
BUV395-A | CD45RA
CCR6-
41.7%
CCR6+
57.8%
PE-A | CD196
PE-Dazzle594-A | CD183
Th17.1
50.3%
Th17DP
7.3%
Th17
18.7%
23.7%
BV605-A | CD194
CCR5-neg
53.1%
CCR5dim
37.9%
CCR5high
9.1%
BV711-A | CD195

B

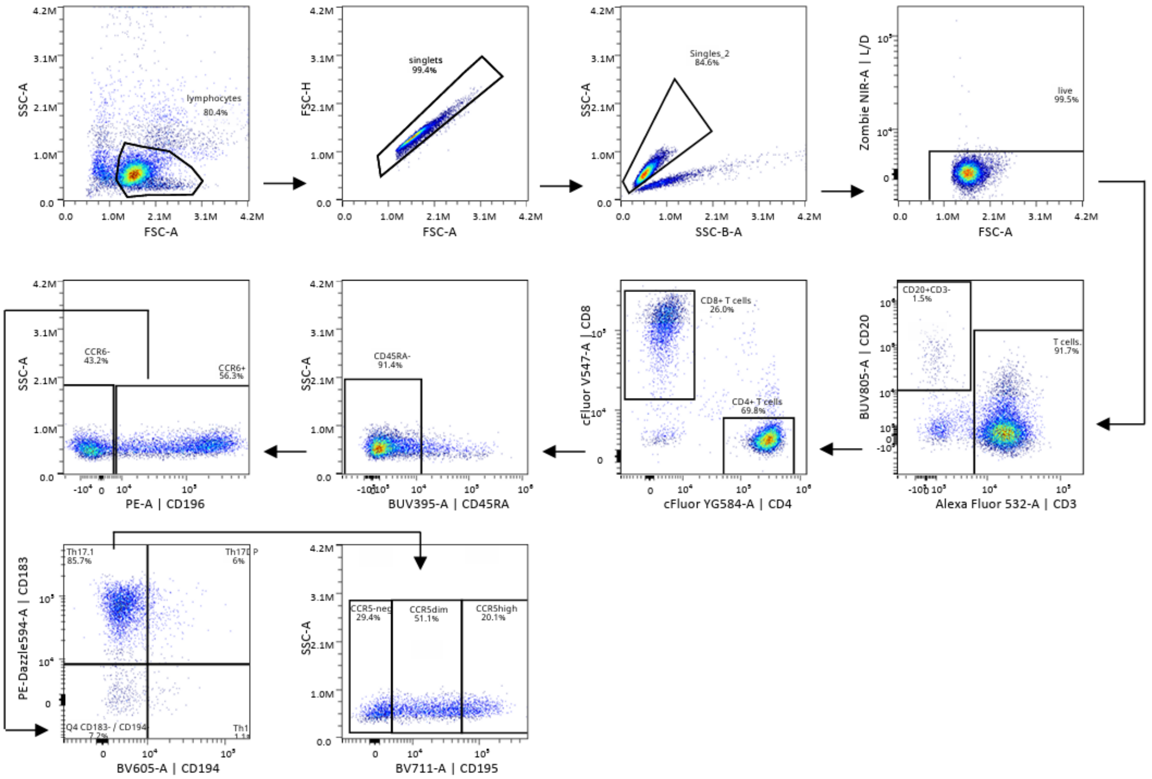
SSC-A
FSC-A
lymphocytes
80.4%
FSC-H
singlets
99.4%
Singles_2
84.6%
SSC-B-A
Zombie NIR-A | L/D
live
99.5%
BUV805-A | CD20
CD20+CD3-
1.5%
T cells.
91.7%
Alexa Fluor 532-A | CD3
cFluor V547-A | CD8
CD8+ T cells
26.0%
CD4+ T cells
69.8%
cFluor YG584-A | CD4
CD45RA-
91.4%
BUV395-A | CD45RA
CCR6-
43.2%
CCR6+
56.3%
PE-A | CD196
PE-Dazzle594-A | CD183
Th17.1
85.7%
6%
Q4 CD183- / CD194
BV605-A | CD194
CCR5-neg
29.4%
CCR5dim
51.1%
CCR5high
20.1%
BV711-A | CD195

Supplemental figure 3: Complete gating strategy of Th17.1 CCR5 neg, dim and high populations. (A) Representative gating strategy in peripheral blood and paired cerebrospinal fluid (B).

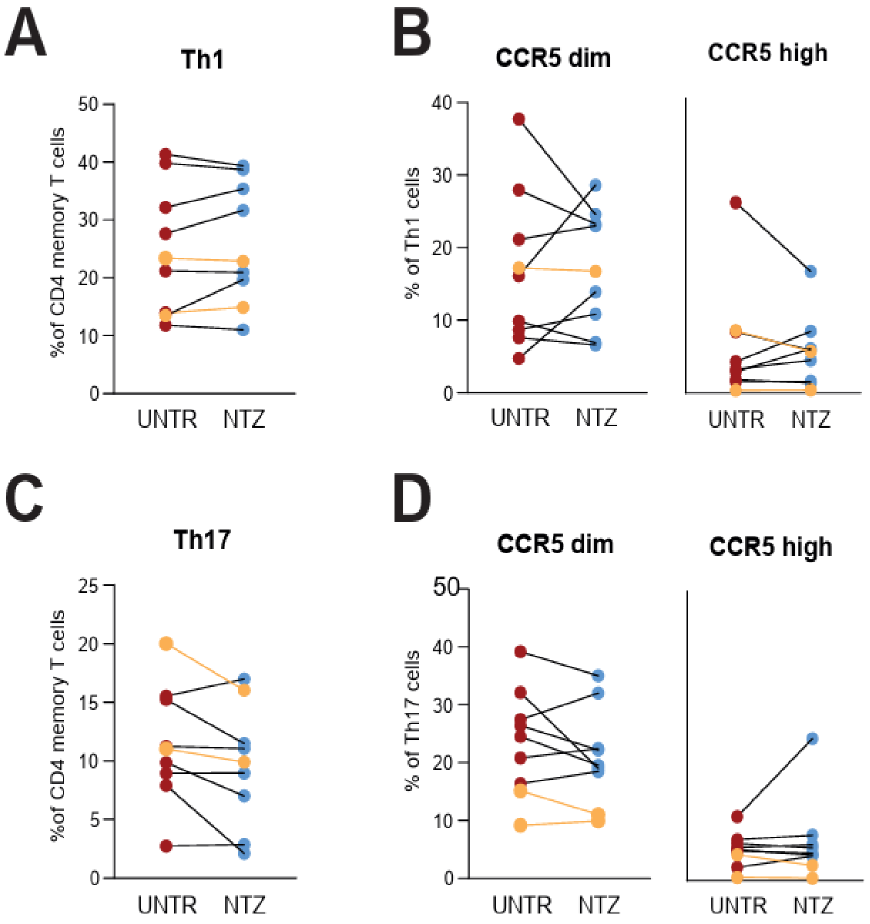

Supplemental figure 4: The effect of natalizumab treatment on Th1 and Th17 subsets. relatively frequency of Th1 (A) and Th17 (C) cells in the MS NTZ (blue) versus MS UNTR (red) group (right). Relative frequencies of CCR5^{dim} (middle) and CCR5^{high} (right) cells within Th1 (B) and Th17 (D) in MS UNTR and MS NTZ group. The orange samples showed decreased frequencies of Th17.1 cells after NTZ treatment. Data were analysed using Wilcoxon signed-rank test

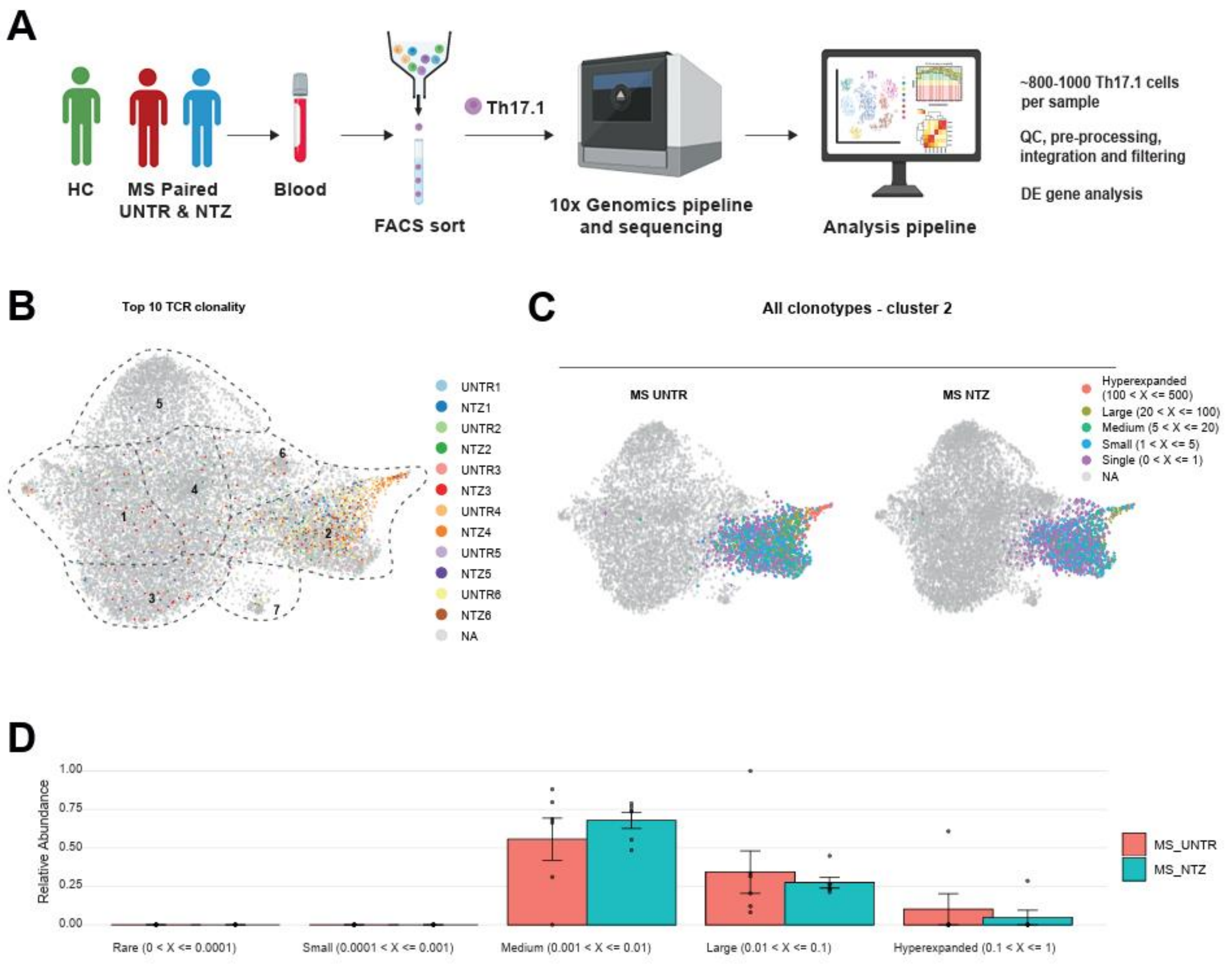

Supplemental figure 5: scRNAseq workflow and TCR clonotype analysis. (A) Graphical representation of the single cell sequencing pipeline (B) Uniform Manifold Approximation and Projection (UMAP) showing 7 clusters with top 10 clones per individual sample in different colours. (C-D) UMAP plot (C) and quantification of relative abundance (D) for clonally-expanded T cells based on the size of the TCR clonality.

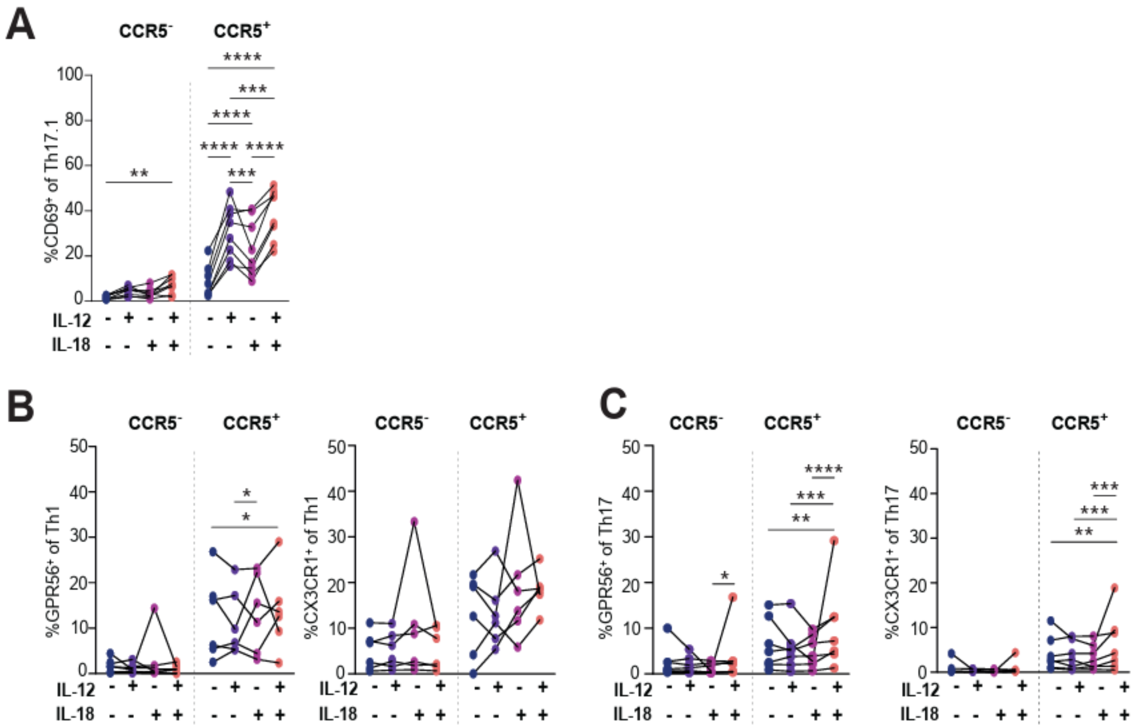

Supplemental figure 6: $CCR5^+$ Th17.1 cells are more activated than $CCR5^-$ counterparts after IL-12 with and without IL-18 stimulation and GPR56 and CX3CR1 are differentially expressed in relation to CCR5 on Th1 or Th17 cells after IL-12 and/or IL-18 stimulation. (A) Relative frequencies of CD69-expressing cells within $CCR5^-$ or $CCR5^+$ Th17.1 after 6 day culture with or without IL-12 and/or IL-18. (B) Relative frequencies of cells expressing GPR56 (A; *left*) and CX3CR1 (A; *right*) within $CCR5^+$ or $CCR5^-$ Th1 and Th17 (C) after the addition of IL-12 and/or IL-18 for 6 days. Each dot in the graphs represents a single individual. Data were analysed using 2-way ANOVA with post-hoc Tukey and obtained after culturing purified subsets from thawed PBMCs of healthy donors. *P ≤ 0.05, **P ≤ 0.01, ***P ≤ 0.001, ****P ≤ 0.0001

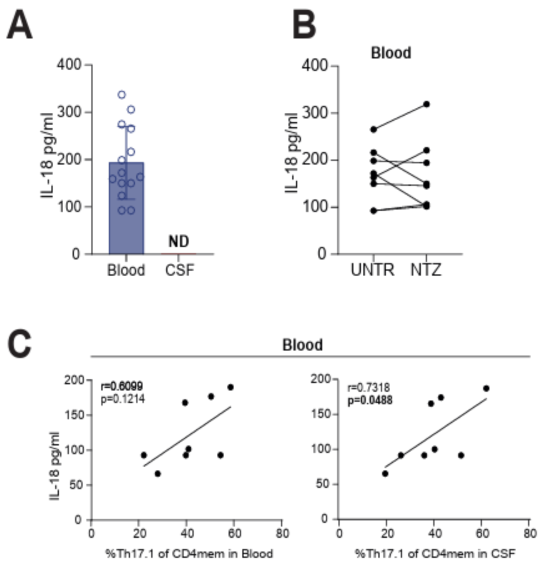

Supplemental figure 7: IL-18 levels in the plasma of untreated pwMS positively correlates with the relative frequencies of Th17.1 cells in the CSF. (A) Concentration of IL-18 (pg/ml) in serum of intreated pwMS and IL-18 concentration pre vs. post natalizumab treatment in the serum of pwMS (B) as measured with Luminex. (C) The correlation of IL-18 serum with the relative frequencies of Th17.1 in peripheral blood (PB; left) and cerebrospinal fluid (CSF; right). Data were analysed using Kruskal-Wallis post hoc Dunn (A-B) or Pearson r test (C). Each dot in the graphs represents a single individual. Data was obtained from thawed material of people with MS .*P ≤ 0.05.

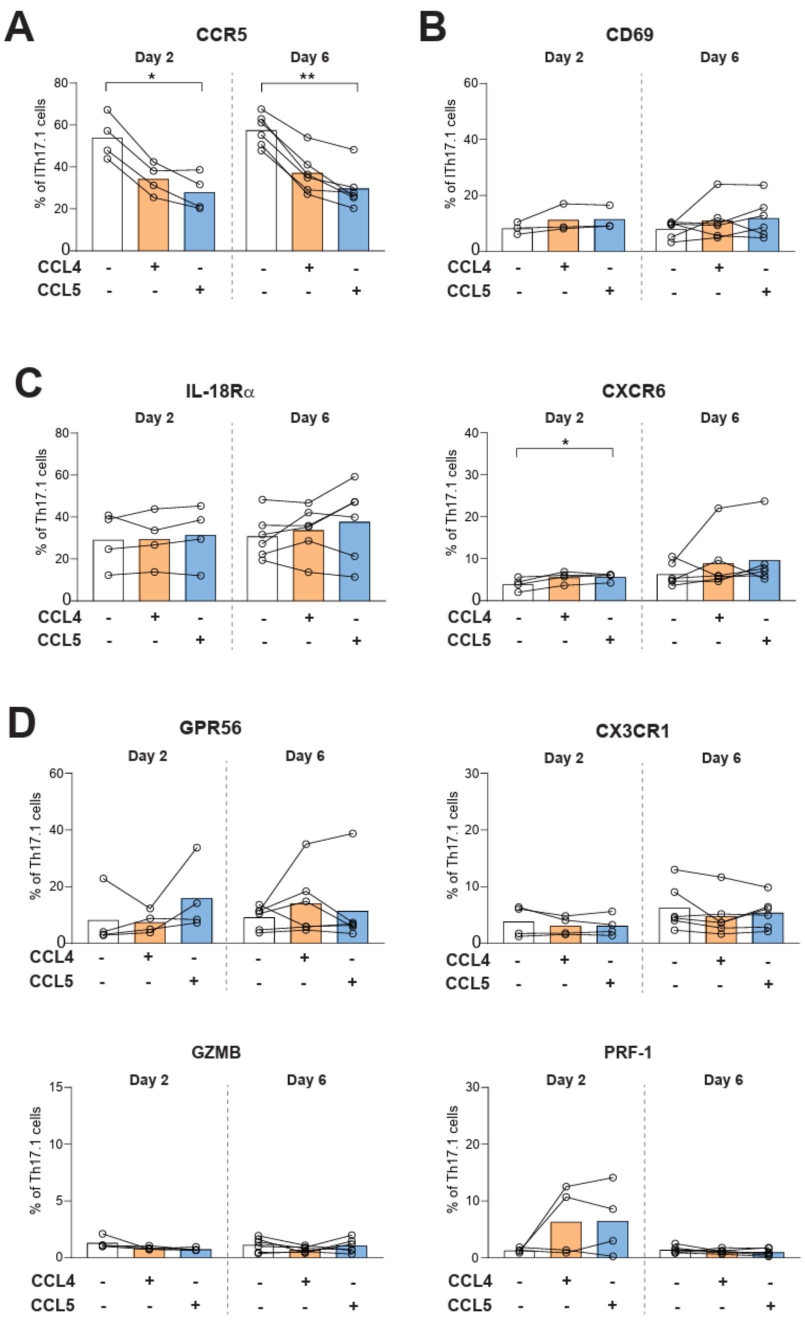

Supplemental figure 8: The effect of CCL4 and CCL5 on Th17.1 signature markers during *in vitro* stimulation. Relative frequencies of chemokine receptor CCR5 (A), early activation marker CD69 (B), Th17.1 associated markers IL-18Rα and CXCR6 (D), cytotoxicity related markers GPR56, CX3CR1, GZMB and PRF-1 (D) after stimulated with both IL-12 and IL-18 for 2 (left) and 6 days (right) in the presence of CCL4 and/or CCL5. Data were analysed using 2-way Friedman test with Dunn's post hoc analysis and obtained with purified subsets from thawed PBMCs of healthy donors.*P ≤ 0.05, **P ≤ 0.01.

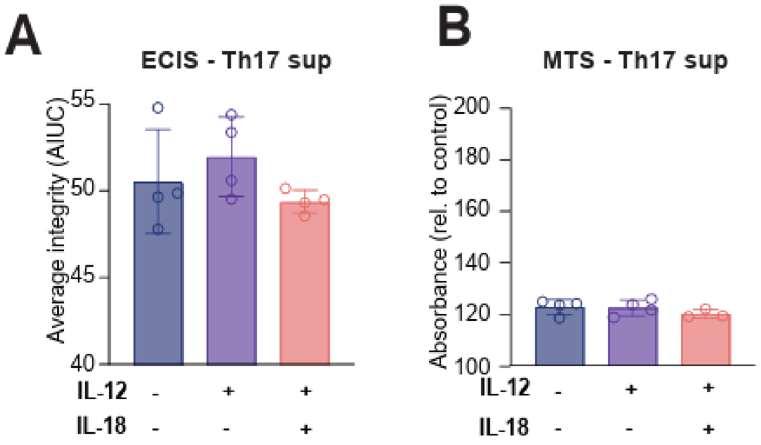

Supplemental figure 9: Supernatant from Th17.1, but not Th17 cells cultured with IL-12+IL-18 lowers *in vitro* blood brain barrier integrity. (A) The relative integrity of cultured human derived brain endothelial cells (hCMEC/D3) with Th17 culture supernatants as measured using electrical cell-substrate impedance spectroscopy (ECIS). (B) Relative hCMEC/D3 cell viability as measured by MTS assays after culturing with Th17 cell culture supernatants for 1 hour. Each dot in the graphs represents a single individual. Data were analysed using Kruskal-Wallis tests post hoc Dunn (G-H) and obtained with purified subsets from thawed PBMCs of healthy donors.*P ≤ 0.05

Supplemental Table 1: List of antibodies

Antibody	Source	Identifier
**Spectral flow cytometry**		
Mouse-anti-human-CD3 (UCHT1) - Alexa Fluor® 532	Invitrogen	Cat# 58-0038-42<br>RRID: AB_11218675
Mouse-anti-human-CD4 (SK3) - cFluor® YG584	Cytek Biosciences	Cat# SKU R7-20042<br>RRID: AB_2885083
Mouse-anti-human-CD8 (SK1) - cFluor® V547	Cytek Biosciences	Cat# SKU R7-20064<br>RRID: AB_3750483
Mouse-anti-human-CD11a (G43-25B) - BUV496	BD Biosciences	Cat# 750663<br>RRID: AB_2874789
Mouse-anti-human-CD16 (3G8) - NovaFluor ™ Blue 610/70s	Invitrogen	Cat# H006T03B06<br>RRID: AB_3098058
Mouse-anti-human-CD19 (HIB19) - eFluor ™ 506	Invitrogen	Cat# 69-0199-42<br>RRID: AB_2637384
Mouse-anti-human-CD20 (2H7) - Brilliant ™ UltraViolet 805	BD Biosciences	Cat# 612905<br>RRID: AB_2870192
Mouse-anti-human-CD20 (2H7) - NovaFluor ™ Blue 610/70S	Invitrogen	Cat# H076T03B06<br>RRID: AB_3098846
Mouse-anti-human-CD25 (2A3) - Brilliant Violet ™ 421	BD Biosciences	Cat# 564033<br>RRID: AB_2738555
Mouse-anti-human-CD25 (3G10) - PE-Alexa Fluor ™ 700	Invitrogen	Cat# MHCD2524<br>RRID: AB_2539740
Mouse-anti-human-CD26 (L272) - Horizon Brilliant ™ Blue 700	BD Biosciences	Cat# 745975<br>RRID: AB_2743381
Rat-anti-human-CD26 (222113) - Alexa Fluor® 700	R&D Systems	Cat# FAB1180N-100UG<br>RRID: AB_3646032
Mouse-anti-human-CD27 (O323) - PerCP-eFluor ™ 710	Invitrogen	Cat# 46-0279-42<br>RRID: AB_1834391
Mouse-anti-human-CD28 (CD28.2) - Brilliant ™ UltraViolet 496	BD Biosciences	Cat# 741168<br>RRID: AB_2870741
Mouse-anti-human-CD29 (MAR4) - Brilliant Violet ™ 750	BD Biosciences	Cat# 755223<br>RRID: AB_3687630
Mouse-anti-human-CD38 (HIT2) - APC/Fire ™ 810	Biolegend	Cat# 303550<br>RRID: AB_2860784
Mouse-anti-human-CD45RA (HI100) - Brilliant ™ UltraViolet 395	BD Biosciences	Cat# 568712<br>RRID: AB_3684488
Mouse-anti-human-CD49d (9F10) - Brilliant Violet ™ 605	Biolegend	Cat# 304324<br>RRID: AB_2566769

Mouse-anti-human-CD56 (B159) - Horizon Brilliant ™ Blue 700	BD Biosciences	Cat# 566400<br>RRID: AB_2722504
Mouse-anti-human-CD56 (HCD6) - APC-Cyanine ™ 7	Biolegend	Cat# 318332<br>RRID: AB_10896424
Mouse-anti-human-CD69 (FN50) - Brilliant ™ UltraViolet 737	BD Biosciences	Cat# 612817<br>RRID: AB_2870141
Mouse-anti-human-CD103 (Ber-ACT8) - Brilliant Violet ™ 421	BD Biosciences	Cat# 747099<br>RRID: AB_2871852
Mouse-anti-human-CD103 (Ber-ACT8) - Brilliant Violet ™ 750	Biolegend	Cat# 350214<br>RRID: AB_2563514
Mouse-anti-human-CD117 (104D2) - PE-Cyanine®5	Biolegend	Cat# 313210<br>RRID: AB_893223
Mouse-anti-human-CD127 (A019D5) - Brilliant Violet ™ 570	Biolegend	Cat# 351308<br>RRID: AB_2832685
Mouse-anti-human-CD160 (BY55) - Alexa Fluor ® 488	BD Biosciences	Cat# 562351<br>RRID: AB_11153688
Mouse-anti-human-CD160 (BY55) - PE-Cyanine®7	Biolegend	Cat# 341212<br>RRID: AB_2562876
Mouse-anti-human-CD161 (HP-3G10) - Pacific Blue ™	Biolegend	Cat# 339926<br>RRID: AB_2563960
Mouse-anti-human-CD183 (G025H7) - PE/Dazzle ™ 594	Biolegend	Cat# 353736<br>RRID: AB_2564288
Mouse-anti-human-CD183 (G025H7) - PE/Fire ™ 810	Biolegend	Cat# 353760<br>RRID: AB_2894484
Mouse-anti-human-CD184 (12G5) - APC-Cyanine ™ 7	Biolegend	Cat# 306528<br>RRID: AB_2565994
Rat-anti-human-CD185 (RF8B2) - Brilliant ™ UltraViolet 395	BD Biosciences	Cat# 740266<br>RRID: AB_2740008
Rat-anti-human-CD185 (RF8B2) - Brilliant ™ UltraViolet 563	BD Biosciences	Cat# 741316<br>RRID: AB_2870835
Mouse-anti-human-CD186 (K041E5) - Alexa Fluor® 647	Biolegend	Cat# 356008<br>RRID: AB_2562225
Mouse-anti-human-CD192 (LS132.1D9) - Brilliant Violet ™ 480	BD Biosciences	Cat# 747852<br>RRID: AB_2872314
Mouse-anti-human-CD194 (L291H4) - Brilliant Violet ™ 605	Biolegend	Cat# 359440<br>RRID: AB_2910405
Mouse-anti-human-CD194 (L291H4) - APC/Fire ™ 810	Biolegend	Cat# 359418<br>RRID: AB_2562483
Rat-anti-human-CD195 (J418F1) - Brilliant Violet ™ 711	Biolegend	Cat# 359130<br>RRID: AB_2734388
Mouse-anti-human-CD196 (G034E3) - PE	Biolegend	Cat# 353410<br>RRID: AB_10913815
Mouse-anti-human-CD197 (G043H7) - Brilliant Violet ™ 785	Biolegend	Cat# 353230<br>RRID: AB_2563630

Mouse-anti-human-CD218a (H44) - APC	Biolegend	Cat# 313814<br>RRID: AB_2800829
Rat-anti-human-CD244 (2B4) - Brilliant Violet ™ 605	Biolegend	Cat# 329536<br>RRID: AB_2814198
Mouse-anti-human-CD244 (eBioC1.7) - PE-Cyanine®5.5	Invitrogen	Cat# 35-5838-42<br>RRID: AB_2784679
Mouse-anti-human-CD279 (EH12.1) - Brilliant ™ UltraViolet 661.	BD Biosciences	Cat# 569246<br>RRID: AB_3684902
Rat-anti-human-Blimp-1 (6D3) - PE-CF®594	BD Biosciences	Cat# 565274<br>RRID: AB_2739147
Mouse-anti-human-CX3CR1 (2A9-1) - Brilliant Violet ™ 650	Biolegend	Cat# 341626<br>RRID: AB_2716245
Mouse-anti-human-Eomes (WD1928) - PE-Cyanine®5.5	Invitrogen	Cat# 35-4877-42<br>RRID: AB_2848321
Mouse-anti-human-GPR56 (CF4.rMAb) - Brilliant ™ UltraViolet 563	BD Biosciences	Cat# 752709<br>RRID: AB_2917690
Mouse-anti-human-GPR56 (CG4) - RealBlue ™ 780	BD Biosciences	Cat# 755559<br>RRID: AB_3687910
Mouse-anti-human-GPR56 (CG4) - PE-Cyanine®7	Biolegend	Cat# 358206<br>RRID: AB_2562090
Mouse-anti-human-Granzyme A (CB9) - Pacific Blue ™	Biolegend	Cat# 507207<br>RRID: AB_439755
Mouse-anti-human-Granzyme B (BG11) - Alexa Fluor ® 700	BD Biosciences	Cat# 560213<br>RRID: AB_1645453
Mouse-anti-human-Granzyme K (GM26E7) - FITC	Biolegend	Cat# 370508<br>RRID: AB_2632846
Mouse-anti-human-HLA-DR (L203.rMAb) - Brilliant™ UltrViolet 615	BD Biosciences	Cat# 752500<br>RRID: AB_2917493
Hamster-anti-human-KLRG1 (SA231A2) - PE/Fire ™ 810	Biolegend	Cat# 138437<br>RRID: AB_2924470
Mouse-anti-human-Perforin-1 (dG9) - PerCP-eFluor ™ 710	Invitrogen	Cat# 46-9994-42<br>RRID: AB_1944475
Mouse-anti-human-Runx3 (R3-5G4) - Brilliant Violet ™ 421	BD Biosciences	Cat# 565742<br>RRID: AB_2916369
Mouse-anti-human-T-bet (4B10) - PE-Cyanine®5	Invitrogen	Cat# 15-5825-82<br>RRID: AB_2815071
ZombieNIR™ Fixable Viability Kit	Biolegend	Cat# 423106<br>RRID: -
Mouse Anti-Stat4 (pY693; clone 38) - PE-CF594	BD Phosflow™	Cat#567631<br>RRID: AB_2916675
BD Horizon™ RB744 Mouse Anti-Human Perforin (dG9)	BD Biosciences	Cat# 570592<br>RRID: AB_3683687

Fluorescent activated cell sorting		
Mouse-anti-human-CD4 (OKT4) - Brilliant Violet ™ 510	Biolegend	Cat#317444 RRID:AB_2561866
Mouse-anti-human-CD3 (SP34-2) - Brilliant Violet ™ 785	BD Biosciences	Cat#563918 RRID: AB_2738487
Mouse-anti-human-CD196 (G034E3) - PE	Biolegend	Cat# 353410 RRID: AB_10913815
Mouse-anti-human-CD183 (G025H7) - PE/Dazzle ™ 594	Biolegend	Cat# 353736 RRID: AB_2564288
Mouse-anti-human-CD184 (12G5) - APC-Cyanine ™ 7	Biolegend	Cat# 306528 RRID: AB_2565994
Mouse-anti-human-CD45RA (HI100) - APC-H7	BD Biosciences	Cat#560674 RRID: AB_1727497
Apotracker ™ green	Biolegend	Cat# 427403 RRID: -

Supplemental Table 2: Luminex analytes

Analyte	1X standard Value pg/ml
CCL2/JE/MCP-1	7,21
CD27/TNFRSF7	24,45
CXCL13/BCL/BCA-1	3,85
GM-CSF	2,58
Granzyme B	7,95
IL-10	1,01
IL-21	6,7
Osteopontin/OPN	394,4
CCL5/RANTES	4,69
CXCL10/IP-10/CGR-2	380
DPPIV/CD26	48,79
Granzyme A	7,28
IFN-gamma	760
IL-17/IL-17A	2.990
Lymphotoxin-alpha/TNF-beta	1,47
TNF-alpha	2,23
IL-12 P70	20.2
IL-18/IL-1F4	1.93

Supplemental Table 3: scRNA seq differentially expressed genes HC vs MS UNTR

Gene	p_value	avg_log2FC	pct.1	pct.2	Adj. P-value	Direction
HLA-C	5,56E-152	0,677303	0,982	0,98	**1,50E-147**	up
SMDT1	3,88E-133	-1,07	0,556	0,846	**1,04E-128**	down
RPS4Y1	4,58E-131	1,285312	0,506	0,209	**1,23E-126**	up
CXCR4	8,91E-115	0,816299	0,882	0,667	**2,40E-110**	up
TXNIP	7,90E-109	0,640212	0,948	0,889	**2,12E-104**	up
TSC22D3	2,28E-98	0,693971	0,915	0,785	**6,14E-94**	up
ZFP36L2	4,42E-97	0,716256	0,969	0,911	**1,19E-92**	up
RPS18	1,11E-73	0,279799	1	1	**2,98E-69**	up
GIMAP7	5,04E-60	-0,74832	0,692	0,784	**1,36E-55**	down
TOB1	4,91E-53	1,151362	0,4	0,246	**1,32E-48**	up
IL32	1,07E-49	0,230033	0,994	0,954	**2,88E-45**	up
ARPC1B	9,89E-48	0,538801	0,697	0,533	**2,66E-43**	up
NINJ1	8,53E-47	-1,0246	0,247	0,33	**2,30E-42**	down
BCL3	1,36E-45	-1,15872	0,172	0,231	**3,66E-41**	down
B2M	3,34E-45	0,101333	1	1	**8,98E-41**	up
RPS10	6,52E-45	0,690632	0,726	0,556	**1,75E-40**	up
PIM3	1,02E-44	-0,838	0,315	0,323	**2,75E-40**	down
GSTM1	1,08E-43	7,850346	0,058	0	**2,92E-39**	up
S100A11	7,52E-43	-0,3949	0,788	0,716	**2,02E-38**	down
CISH	9,08E-43	-1,33432	0,174	0,299	**2,44E-38**	down
S100A4	1,15E-42	0,256734	0,993	0,986	**3,10E-38**	up
PTPRCAP	1,21E-42	0,807213	0,529	0,38	**3,27E-38**	up
HLA-A	1,30E-42	0,191096	0,996	0,994	**3,50E-38**	up
GZMK	2,55E-41	1,013262	0,37	0,204	**6,87E-37**	up
SOCS3	2,72E-41	-1,09015	0,25	0,308	**7,31E-37**	down
DUSP2	3,77E-41	0,728201	0,533	0,346	**1,01E-36**	up
DUSP1	7,06E-41	0,58217	0,732	0,571	**1,90E-36**	up
GPR183	1,33E-39	-0,91401	0,297	0,47	**3,58E-35**	down
JUNB	2,19E-36	0,34512	0,942	0,845	**5,88E-32**	up
OAZ1	3,67E-36	0,151198	0,918	0,8	**9,87E-32**	up
ARHGDIB	1,29E-35	0,190541	0,99	0,968	**3,48E-31**	up
ANXA1	1,73E-34	0,169529	0,905	0,789	**4,65E-30**	up
S100A6	5,17E-34	0,258672	0,971	0,948	**1,39E-29**	up
LYAR	8,47E-34	0,600112	0,637	0,506	**2,28E-29**	up
CFL1	1,07E-33	0,170476	0,991	0,961	**2,87E-29**	up
CD81	2,49E-33	0,428718	0,741	0,625	**6,70E-29**	up
DNAJB1	1,41E-32	0,489686	0,699	0,571	**3,78E-28**	up
MYL12A	4,57E-32	0,109754	0,993	0,954	**1,23E-27**	up
NFKBIA	4,53E-31	0,263717	0,855	0,724	**1,22E-26**	up

ETS1	5,20E-31	0,332087	0,788	0,686	**1,40E-26**	up
CST7	1,20E-30	0,984269	0,327	0,194	**3,23E-26**	up
NKG7	5,06E-30	1,523111	0,339	0,233	**1,36E-25**	up
IL2RG	4,31E-29	0,197626	0,89	0,807	**1,16E-24**	up
RELB	2,17E-28	-0,90593	0,182	0,245	**5,85E-24**	down
NME2	2,36E-28	0,521566	0,699	0,572	**6,35E-24**	up
KLF2	2,68E-28	0,187994	0,967	0,933	**7,21E-24**	up
GZMA	1,82E-27	0,856479	0,366	0,239	**4,89E-23**	up
MYBL1	2,66E-27	0,74916	0,354	0,227	**7,17E-23**	up
MYC	4,47E-27	-0,68921	0,412	0,531	**1,20E-22**	down
MZT2A	5,22E-26	0,421501	0,647	0,514	**1,41E-21**	up
FLNA	1,45E-25	0,467586	0,552	0,446	**3,90E-21**	up
ANXA2	1,73E-25	0,377236	0,598	0,464	**4,65E-21**	up
UBC	2,28E-25	0,197964	0,977	0,949	**6,14E-21**	up
GRASP	2,97E-25	-1,31472	0,097	0,174	**7,99E-21**	down
LCP1	4,71E-25	0,310889	0,737	0,643	**1,27E-20**	up
LIME1	1,11E-24	0,466581	0,516	0,411	**2,97E-20**	up
CTAG2	1,12E-24	-3,9914	0,003	0,041	**3,02E-20**	down
CCDC167	1,86E-24	0,797282	0,236	0,129	**5,00E-20**	up
EEF2	4,61E-24	0,155186	0,986	0,987	**1,24E-19**	up
EZR	1,21E-23	0,425401	0,599	0,474	**3,26E-19**	up
WHAMM	4,38E-23	-0,33929	0,288	0,274	**1,18E-18**	down
HERPUD1	1,43E-22	0,602192	0,403	0,288	**3,84E-18**	up
ITM2B	1,44E-22	0,120344	0,989	0,976	**3,89E-18**	up
SUN2	2,96E-22	0,333557	0,623	0,538	**7,97E-18**	up
GIMAP4	4,68E-22	-0,52389	0,526	0,593	**1,26E-17**	down
DDX24	8,68E-22	-0,49583	0,552	0,633	**2,33E-17**	down
DDIT4	8,98E-22	0,28305	0,641	0,508	**2,41E-17**	up
VIM	1,10E-21	0,166002	0,981	0,975	**2,97E-17**	up
RPL17	1,32E-21	0,244481	0,909	0,831	**3,55E-17**	up
LAPTM5	1,81E-21	0,179028	0,919	0,89	**4,88E-17**	up
CDKN1B	1,87E-21	0,376996	0,626	0,518	**5,03E-17**	up
SRGN	1,98E-21	0,383118	0,712	0,615	**5,34E-17**	up
RPS29	2,03E-21	-0,26503	0,99	0,971	**5,46E-17**	down
CPNE1	2,41E-21	0,755653	0,238	0,136	**6,49E-17**	up
ARL4C	4,89E-21	0,358992	0,733	0,671	**1,32E-16**	up
TNFAIP3	5,31E-21	0,381288	0,68	0,557	**1,43E-16**	up
AHNAK	6,00E-21	0,265439	0,657	0,585	**1,61E-16**	up
CD99	8,82E-21	0,239536	0,873	0,812	**2,37E-16**	up
ALDOA	8,90E-21	0,193919	0,892	0,851	**2,39E-16**	up
SH3BGRL3	1,25E-20	0,110653	0,996	0,988	**3,37E-16**	up

BATF	1,67E-20	-1,04964	0,116	0,19	**4,49E-16**	down
RPS27	2,36E-20	-0,22044	1	1	**6,34E-16**	down
TPT1	2,66E-20	0,11021	1	1	**7,17E-16**	up
TRADD	2,97E-20	0,320878	0,577	0,466	**7,98E-16**	up
RPL36A	3,01E-20	-0,13729	0,85	0,786	**8,10E-16**	down
TGFB1	3,77E-20	0,245683	0,822	0,778	**1,01E-15**	up
ATP5E	3,95E-20	0,133977	0,983	0,972	**1,06E-15**	up
GTF3A	5,88E-20	0,287225	0,754	0,685	**1,58E-15**	up
ENO1	1,11E-19	0,254068	0,721	0,627	**2,98E-15**	up
OASL	1,37E-19	-2,03126	0,032	0,079	**3,69E-15**	down
GNLY	1,82E-19	1,18147	0,112	0,153	**4,91E-15**	up
FAM117A	2,36E-19	0,473339	0,427	0,31	**6,34E-15**	up
UCP2	5,16E-19	0,168252	0,718	0,6	**1,39E-14**	up
EFHD2	7,04E-19	0,591473	0,431	0,332	**1,89E-14**	up
CBWD1	7,20E-19	1,976283	0,061	0,014	**1,94E-14**	up
EVL	8,42E-19	0,157453	0,873	0,799	**2,27E-14**	up
YWHAQ	9,81E-19	0,325888	0,6	0,495	**2,64E-14**	up
CEBPD	1,76E-18	1,034965	0,175	0,09	**4,73E-14**	up
AMBRA1	1,89E-18	0,814728	0,255	0,159	**5,09E-14**	up
RPL26	7,38E-18	0,185518	0,998	0,99	**1,98E-13**	up
PTP4A2	9,19E-18	0,278863	0,725	0,636	**2,47E-13**	up
GAPDH	1,03E-17	0,150968	0,986	0,979	**2,78E-13**	up
CCR6	1,16E-17	0,42012	0,378	0,292	**3,12E-13**	up
ALOX5AP	1,35E-17	0,353832	0,555	0,443	**3,63E-13**	up
GZMH	1,46E-17	2,524733	0,054	0,012	**3,92E-13**	up
PARP8	1,88E-17	0,370002	0,539	0,446	**5,06E-13**	up
FYN	1,98E-17	0,315693	0,623	0,52	**5,32E-13**	up
SNHG8	2,80E-17	-0,31884	0,643	0,658	**7,54E-13**	down
SLC4A10	3,24E-17	1,36073	0,122	0,053	**8,70E-13**	up
UQCR11	5,21E-17	0,240996	0,792	0,719	**1,40E-12**	up
P4HB	5,26E-17	0,340149	0,539	0,457	**1,41E-12**	up
TNFAIP8L2	6,71E-17	-1,59348	0,031	0,084	**1,80E-12**	down
SKP1	7,20E-17	0,176814	0,806	0,721	**1,94E-12**	up
RP11-290D2.6	7,24E-17	0,341978	0,579	0,51	**1,95E-12**	up
LGALS3	8,12E-17	0,511311	0,415	0,306	**2,18E-12**	up
SYTL1	8,54E-17	0,32597	0,554	0,454	**2,30E-12**	up
DNAJA1	9,42E-17	-0,49539	0,378	0,464	**2,53E-12**	down
NSG1	1,51E-16	0,563659	0,324	0,225	**4,05E-12**	up
SIPA1	1,54E-16	0,615758	0,259	0,162	**4,13E-12**	up
TMEM256-PLSCR3	1,68E-16	0,51831	0,299	0,209	**4,51E-12**	up
NCL	1,68E-16	0,242298	0,745	0,653	**4,52E-12**	up

SYNE2	1,79E-16	0,300784	0,588	0,498	**4,82E-12**	up
CD52	1,90E-16	0,126644	0,956	0,938	**5,10E-12**	up
PTPRC	2,30E-16	0,133772	0,925	0,872	**6,18E-12**	up
PSPH	2,36E-16	-1,96438	0,021	0,067	**6,35E-12**	down
RHOA	2,59E-16	0,136615	0,9	0,858	**6,97E-12**	up
CTSH	2,59E-16	0,569221	0,28	0,196	**6,97E-12**	up
HCLS1	3,97E-16	0,220786	0,696	0,592	**1,07E-11**	up
STK17A	6,03E-16	-0,46328	0,417	0,492	**1,62E-11**	down
RBM38	6,16E-16	-0,26739	0,424	0,395	**1,66E-11**	down
MSN	6,33E-16	0,311797	0,569	0,483	**1,70E-11**	up
FAM118A	7,23E-16	-1,05041	0,097	0,172	**1,94E-11**	down
CCL5	7,69E-16	0,432621	0,561	0,458	**2,07E-11**	up
PLEK	8,23E-16	1,261551	0,129	0,064	**2,21E-11**	up
ITGB2	9,15E-16	0,217605	0,658	0,596	**2,46E-11**	up
BIN1	9,82E-16	0,309166	0,629	0,542	**2,64E-11**	up
CYBA	1,03E-15	0,102623	0,943	0,893	**2,76E-11**	up
COMMD6	1,23E-15	0,152495	0,954	0,934	**3,30E-11**	up
EIF4A1	1,39E-15	0,690117	0,232	0,143	**3,73E-11**	up
CNBP	1,69E-15	0,136698	0,929	0,891	**4,55E-11**	up
AC241377.2	2,03E-15	2,566168	0,036	0,005	**5,46E-11**	up
MYH9	2,10E-15	0,248299	0,591	0,517	**5,65E-11**	up
ATF7IP2	2,47E-15	0,462105	0,376	0,283	**6,65E-11**	up
RP11-124N14.3	2,64E-15	0,557122	0,333	0,243	**7,09E-11**	up
DDX5	2,72E-15	0,144373	0,961	0,928	**7,32E-11**	up
CD69	2,93E-15	0,147332	0,802	0,708	**7,88E-11**	up
PRDX2	3,92E-15	0,232029	0,633	0,532	**1,05E-10**	up
KLF3	4,18E-15	0,346537	0,482	0,402	**1,13E-10**	up
GABARAP	4,69E-15	0,169429	0,839	0,78	**1,26E-10**	up
C14orf119	5,59E-15	-0,87193	0,135	0,213	**1,50E-10**	down
LSP1	5,85E-15	0,179354	0,83	0,774	**1,57E-10**	up
ARHGEF1	6,10E-15	0,25555	0,625	0,555	**1,64E-10**	up
GNB2	6,94E-15	0,271917	0,616	0,523	**1,87E-10**	up
CD74	7,45E-15	0,241319	0,807	0,761	**2,00E-10**	up
PRF1	8,80E-15	0,997334	0,222	0,154	**2,37E-10**	up
HNRNPA2B1	1,15E-14	0,124005	0,897	0,837	**3,10E-10**	up
NDUFA3	1,30E-14	0,357226	0,525	0,427	**3,50E-10**	up
KANSL1-AS1	1,72E-14	0,78469	0,196	0,119	**4,61E-10**	up
PIM2	1,72E-14	-0,54145	0,336	0,418	**4,63E-10**	down
ACTN4	2,30E-14	0,593197	0,208	0,132	**6,19E-10**	up
TRAF4	2,52E-14	-1,63993	0,026	0,066	**6,79E-10**	down
RAC2	3,61E-14	-0,10507	0,923	0,863	**9,71E-10**	down

PMAIP1	3,63E-14	-1,42896	0,085	0,112	**9,76E-10**	down
IFITM3	5,04E-14	0,422644	0,33	0,234	**1,35E-09**	up
TNFSF8	5,72E-14	0,474048	0,25	0,162	**1,54E-09**	up
C10orf54	5,99E-14	0,256152	0,581	0,481	**1,61E-09**	up
CALR	7,31E-14	0,257222	0,636	0,546	**1,97E-09**	up
PRAM1	1,04E-13	6,152996	0,017	0	**2,79E-09**	up
COPE	1,14E-13	0,319183	0,502	0,416	**3,06E-09**	up
DYNLT1	1,16E-13	0,29334	0,491	0,407	**3,13E-09**	up
FAM107B	1,27E-13	0,264861	0,681	0,599	**3,42E-09**	up
ADGRE5	1,87E-13	0,34755	0,392	0,305	**5,03E-09**	up
PPIB	2,53E-13	0,143371	0,891	0,839	**6,80E-09**	up
MRPL10	2,95E-13	0,535347	0,272	0,184	**7,92E-09**	up
SAMHD1	3,02E-13	0,197538	0,549	0,446	**8,13E-09**	up
CAPZB	3,32E-13	0,149826	0,762	0,686	**8,93E-09**	up
RBM39	3,35E-13	-0,35882	0,588	0,644	**9,02E-09**	down
DDX21	3,91E-13	-0,46224	0,354	0,395	**1,05E-08**	down
GSTP1	4,36E-13	0,238957	0,583	0,488	**1,17E-08**	up
RPN1	4,96E-13	0,319277	0,385	0,291	**1,33E-08**	up
RASAL3	5,20E-13	0,450826	0,323	0,24	**1,40E-08**	up
HNRNPDL	5,85E-13	0,148986	0,875	0,834	**1,57E-08**	up
EID1	5,87E-13	0,243232	0,631	0,543	**1,58E-08**	up
IL18RAP	8,78E-13	1,44254	0,072	0,027	**2,36E-08**	up
CCSER2	9,82E-13	0,518158	0,277	0,196	**2,64E-08**	up
ANXA6	1,02E-12	0,225537	0,558	0,467	**2,74E-08**	up
HMGB2	1,17E-12	0,345496	0,475	0,384	**3,15E-08**	up
NDUFA1	1,18E-12	0,221547	0,686	0,608	**3,17E-08**	up
SBDS	1,38E-12	-0,12027	0,419	0,386	**3,70E-08**	down
RPN2	1,40E-12	0,278838	0,487	0,409	**3,77E-08**	up
AC245100.1	1,42E-12	0,505589	0,276	0,195	**3,81E-08**	up
MTFP1	1,58E-12	0,669791	0,198	0,129	**4,26E-08**	up
TAGLN2	1,60E-12	0,113261	0,893	0,864	**4,31E-08**	up
VAMP2	2,52E-12	0,41284	0,363	0,277	**6,77E-08**	up
FMN1	2,57E-12	5,886782	0,015	0	**6,91E-08**	up
WIPF1	3,05E-12	0,353963	0,447	0,359	**8,21E-08**	up
EIF1	3,48E-12	-0,18978	0,999	0,997	**9,37E-08**	down
LTK	3,50E-12	1,034356	0,105	0,051	**9,42E-08**	up
SATB1	3,54E-12	0,486257	0,317	0,24	**9,51E-08**	up
HLA-F	3,67E-12	0,219304	0,587	0,517	**9,87E-08**	up
KRTCAP2	5,28E-12	0,202111	0,737	0,654	**1,42E-07**	up
EMB	6,49E-12	0,275921	0,508	0,417	**1,75E-07**	up
ZAP70	6,65E-12	0,264324	0,516	0,439	**1,79E-07**	up

CKLF	7,68E-12	0,639068	0,221	0,148	**2,06E-07**	up
GMFG	7,69E-12	0,126826	0,861	0,818	**2,07E-07**	up
ICOS	1,10E-11	-0,82438	0,108	0,16	**2,95E-07**	down
AUTS2	1,15E-11	0,574251	0,205	0,132	**3,10E-07**	up
LITAF	1,21E-11	0,274339	0,506	0,425	**3,26E-07**	up
STK24	1,24E-11	0,331425	0,397	0,32	**3,34E-07**	up
TSPAN15	2,35E-11	0,881915	0,095	0,049	**6,31E-07**	up
DNAJB6	2,75E-11	-0,39898	0,309	0,347	**7,40E-07**	down
BCL6	3,00E-11	-1,10159	0,036	0,077	**8,06E-07**	down
RNF145	3,06E-11	-0,37113	0,22	0,232	**8,23E-07**	down
RGCC	3,26E-11	-0,68885	0,224	0,307	**8,77E-07**	down
DYNLL1	3,76E-11	-0,5972	0,259	0,325	**1,01E-06**	down
MAP1LC3B	3,85E-11	0,330638	0,459	0,375	**1,03E-06**	up
SNRPN	3,90E-11	0,46509	0,297	0,222	**1,05E-06**	up
PITPNC1	3,89E-11	0,283203	0,441	0,363	**1,05E-06**	up
CRIP2	4,02E-11	0,491239	0,222	0,148	**1,08E-06**	up
GNAI2	4,15E-11	0,153618	0,734	0,676	**1,12E-06**	up
H1FX	4,34E-11	0,289327	0,713	0,645	**1,17E-06**	up
BIN2	4,89E-11	0,164199	0,672	0,615	**1,31E-06**	up
CCND3	5,22E-11	0,108632	0,813	0,751	**1,41E-06**	up
STX11	5,71E-11	-1,18802	0,029	0,065	**1,54E-06**	down
MIF	5,83E-11	0,12308	0,95	0,925	**1,57E-06**	up
WAS	6,09E-11	0,232014	0,533	0,458	**1,64E-06**	up
DENND2D	6,92E-11	-0,51124	0,299	0,363	**1,86E-06**	down
FYB	7,57E-11	0,145874	0,703	0,639	**2,04E-06**	up
NGFRAP1	8,02E-11	0,733272	0,14	0,08	**2,16E-06**	up
LSM7	8,06E-11	0,202982	0,628	0,547	**2,17E-06**	up
M6PR	1,08E-10	0,189486	0,466	0,374	**2,91E-06**	up
RHOB	1,17E-10	2,37823	0,036	0,009	**3,14E-06**	up
ANXA5	1,20E-10	0,294498	0,374	0,291	**3,21E-06**	up
ECH1	1,24E-10	0,582443	0,166	0,102	**3,35E-06**	up
TRBV7-2	1,44E-10	-1,1289	0,034	0,075	**3,88E-06**	down
BSG	1,48E-10	0,187203	0,507	0,414	**3,98E-06**	up
NCF1	1,62E-10	-0,64788	0,205	0,283	**4,36E-06**	down
ZFP36	2,05E-10	0,150776	0,752	0,669	**5,51E-06**	up
BHLHE40	2,46E-10	0,39352	0,352	0,267	**6,62E-06**	up
CHST12	3,25E-10	0,641353	0,19	0,125	**8,75E-06**	up
CTDNEP1	3,37E-10	0,251127	0,419	0,362	**9,07E-06**	up
KCNAB2	3,40E-10	0,412332	0,232	0,162	**9,16E-06**	up
CCR7	3,54E-10	-0,66568	0,246	0,321	**9,53E-06**	down
PKM	3,70E-10	0,135484	0,615	0,565	**9,96E-06**	up

SIT1	4,27E-10	-0,54868	0,278	0,344	**1,15E-05**	down
DNLZ	5,08E-10	0,531987	0,224	0,156	**1,37E-05**	up
PAIP2	5,16E-10	0,22532	0,603	0,525	**1,39E-05**	up
JUN	6,01E-10	0,372478	0,515	0,434	**1,62E-05**	up
PDIA3	6,20E-10	0,232834	0,629	0,558	**1,67E-05**	up
EIF1AY	6,36E-10	1,670991	0,056	0,022	**1,71E-05**	up
CTSD	6,45E-10	0,359751	0,34	0,262	**1,74E-05**	up
TIMM17A	6,55E-10	-0,65337	0,104	0,144	**1,76E-05**	down
IFIT2	6,62E-10	-1,81512	0,02	0,048	**1,78E-05**	down
AKNA	6,86E-10	0,354947	0,29	0,228	**1,84E-05**	up
MINOS1	7,10E-10	0,368808	0,327	0,254	**1,91E-05**	up
PSMA6	7,30E-10	0,314957	0,415	0,34	**1,96E-05**	up
LAIR2	7,65E-10	-1,03098	0,02	0,051	**2,06E-05**	down
AC092580.4	9,91E-10	0,72452	0,169	0,11	**2,67E-05**	up
LY6E	9,98E-10	0,134017	0,786	0,751	**2,68E-05**	up
LCP2	1,04E-09	0,205662	0,527	0,451	**2,81E-05**	up
PLP2	1,06E-09	0,148046	0,616	0,538	**2,85E-05**	up
RAP1A	1,10E-09	0,27376	0,473	0,398	**2,96E-05**	up
ABHD17A	1,56E-09	0,181231	0,553	0,483	**4,19E-05**	up
TSC22D4	1,63E-09	0,261062	0,426	0,343	**4,38E-05**	up
NDUFA13	1,89E-09	0,532455	0,203	0,139	**5,08E-05**	up
CRIP1	1,94E-09	0,162454	0,62	0,552	**5,21E-05**	up
LEPROTL1	2,09E-09	0,139015	0,766	0,726	**5,61E-05**	up
CTD-3184A7.4	2,34E-09	0,396495	0,294	0,223	**6,29E-05**	up
GLIPR1	2,40E-09	0,31752	0,34	0,265	**6,45E-05**	up
ATPIF1	2,44E-09	0,152049	0,661	0,593	**6,55E-05**	up
ERP29	2,69E-09	0,114549	0,786	0,754	**7,23E-05**	up
SCAND1	2,74E-09	0,215546	0,554	0,502	**7,38E-05**	up
MPRIP	2,84E-09	0,456789	0,219	0,154	**7,63E-05**	up
TLE1	2,88E-09	1,709944	0,045	0,015	**7,76E-05**	up
EIF5A	2,91E-09	-0,11613	0,699	0,664	**7,83E-05**	down
ARHGAP15	2,95E-09	-0,30822	0,816	0,865	**7,93E-05**	down
MXRA7	3,01E-09	0,892221	0,081	0,04	**8,09E-05**	up
CD96	3,02E-09	0,209356	0,545	0,467	**8,11E-05**	up
RALA	3,10E-09	-0,54267	0,165	0,219	**8,34E-05**	down
COLQ	4,06E-09	1,032908	0,075	0,036	**1,09E-04**	up
NCK2	4,25E-09	0,213019	0,501	0,428	**1,14E-04**	up
RNF19A	4,26E-09	0,467779	0,226	0,158	**1,15E-04**	up
PIM1	4,77E-09	-0,35343	0,516	0,564	**1,28E-04**	down
SAP30BP	4,76E-09	-0,53144	0,181	0,237	**1,28E-04**	down
SMAD3	4,81E-09	0,539885	0,189	0,135	**1,29E-04**	up

U2AF1L5	5,15E-09	-1,40026	0,011	0,035	**1,39E-04**	down
MAP2K2	5,20E-09	0,226566	0,49	0,416	**1,40E-04**	up
RNPEPL1	5,35E-09	0,272838	0,386	0,309	**1,44E-04**	up
FO538757.2	6,33E-09	0,478302	0,193	0,135	**1,70E-04**	up
HLA-DRB1	6,31E-09	1,30464	0,061	0,029	**1,70E-04**	up
BCAP31	6,54E-09	0,170619	0,598	0,519	**1,76E-04**	up
KDM5D	6,90E-09	1,760355	0,045	0,016	**1,85E-04**	up
PRR13	6,90E-09	0,146449	0,688	0,636	**1,86E-04**	up
TUBA1C	7,54E-09	0,581247	0,184	0,123	**2,03E-04**	up
IER2	7,75E-09	0,18994	0,707	0,677	**2,08E-04**	up
RBX1	8,74E-09	-0,39579	0,438	0,509	**2,35E-04**	down
ADRBK1	9,61E-09	0,278846	0,408	0,342	**2,58E-04**	up
PTGER2	9,59E-09	0,140144	0,46	0,379	**2,58E-04**	up
TLN1	9,58E-09	0,25381	0,418	0,341	**2,58E-04**	up
CTBP1	9,58E-09	0,2903	0,351	0,284	**2,58E-04**	up
ERN1	9,77E-09	0,294741	0,436	0,368	**2,63E-04**	up
SRSF5	9,82E-09	-0,22745	0,877	0,908	**2,64E-04**	down
TRBV3-1	9,89E-09	1,337683	0,066	0,037	**2,66E-04**	up
HCG18	1,01E-08	0,483238	0,142	0,089	**2,72E-04**	up
LST1	1,02E-08	0,557105	0,168	0,116	**2,75E-04**	up
ADGRE2	1,05E-08	-4,04681	0,001	0,013	**2,82E-04**	down
CNOT1	1,05E-08	-0,38866	0,214	0,243	**2,83E-04**	down
ADAM12	1,10E-08	1,424542	0,042	0,014	**2,97E-04**	up
FAM13A	1,12E-08	1,078734	0,054	0,023	**3,01E-04**	up
COL5A3	1,13E-08	1,14113	0,046	0,019	**3,04E-04**	up
CTA-29F11.1	1,15E-08	0,563355	0,15	0,095	**3,09E-04**	up
NDUFA11	1,29E-08	0,434843	0,244	0,177	**3,47E-04**	up
15-sep	1,40E-08	0,209246	0,515	0,443	**3,75E-04**	up
GLRX	1,40E-08	-0,5155	0,281	0,347	**3,77E-04**	down
CAPN2	1,42E-08	0,249053	0,433	0,365	**3,81E-04**	up
LRRN3	1,52E-08	1,640275	0,045	0,016	**4,10E-04**	up
C17orf97	1,57E-08	5,403039	0,01	0	**4,22E-04**	up
PHACTR2	1,62E-08	0,42434	0,309	0,246	**4,35E-04**	up
PLD3	1,64E-08	0,403096	0,236	0,171	**4,42E-04**	up
PIK3IP1	1,77E-08	0,247211	0,453	0,392	**4,77E-04**	up
KDSR	1,86E-08	0,345682	0,237	0,173	**4,99E-04**	up
SPTAN1	1,88E-08	0,229791	0,391	0,327	**5,06E-04**	up
PBXIP1	1,89E-08	0,197432	0,505	0,436	**5,09E-04**	up
FAM26F	1,99E-08	0,789956	0,11	0,065	**5,35E-04**	up
SSBP4	2,06E-08	0,270263	0,465	0,39	**5,54E-04**	up
TAPBP	2,21E-08	0,241081	0,443	0,385	**5,96E-04**	up

ADAM8	2,33E-08	0,360517	0,245	0,184	**6,27E-04**	up
CANX	2,50E-08	0,275586	0,401	0,331	**6,72E-04**	up
HNRNPR	2,52E-08	0,268294	0,413	0,341	**6,77E-04**	up
MAD1L1	2,69E-08	0,39647	0,257	0,191	**7,24E-04**	up
MYO1G	2,79E-08	0,303911	0,36	0,296	**7,51E-04**	up
PARP10	2,98E-08	0,467755	0,147	0,1	**8,03E-04**	up
ANXA11	2,99E-08	0,139161	0,624	0,555	**8,04E-04**	up
TRBV4-2	2,99E-08	-1,3395	0,012	0,034	**8,05E-04**	down
XRCC5	3,07E-08	0,165692	0,565	0,494	**8,25E-04**	up
SNHG25	3,22E-08	0,39786	0,268	0,204	**8,67E-04**	up
HMHA1	3,24E-08	0,254542	0,448	0,385	**8,71E-04**	up
IGFLR1	3,30E-08	0,668411	0,125	0,078	**8,87E-04**	up
PLAC8	4,13E-08	-0,43342	0,3	0,373	**0,001112**	down
RASGEF1B	4,18E-08	-0,90099	0,043	0,078	**0,001123**	down
TMEM109	4,19E-08	0,327898	0,31	0,246	**0,001128**	up
PGK1	4,22E-08	0,104411	0,741	0,687	**0,001134**	up
COX5B	4,45E-08	0,104348	0,794	0,75	**0,001197**	up
NDFIP1	4,49E-08	0,182572	0,586	0,525	**0,001209**	up
PIP4K2A	4,94E-08	0,29621	0,359	0,284	**0,001328**	up
PLEKHJ1	5,40E-08	0,318793	0,333	0,262	**0,001453**	up
XRRA1	5,46E-08	0,928626	0,079	0,042	**0,001469**	up
TP53I13	5,60E-08	0,422695	0,229	0,171	**0,001506**	up
ARRB2	6,01E-08	0,172867	0,522	0,476	**0,001618**	up
PDIA6	6,18E-08	0,234593	0,426	0,358	**0,001663**	up
ITPKB	6,85E-08	0,410249	0,197	0,141	**0,001843**	up
EOMES	7,20E-08	1,065261	0,068	0,035	**0,001937**	up
DPP4	7,86E-08	0,38999	0,242	0,178	**0,002115**	up
HIF1A	7,91E-08	-0,36985	0,181	0,204	**0,002129**	down
ESYT1	8,32E-08	0,293434	0,247	0,193	**0,002237**	up
STAT1	8,52E-08	-0,65668	0,239	0,289	**0,002293**	down
LAMTOR5	8,93E-08	-0,42735	0,247	0,312	**0,002401**	down
LPIN2	9,02E-08	0,318275	0,277	0,214	**0,002426**	up
LINC00402	9,27E-08	-1,31928	0,024	0,053	**0,002493**	down
SSBP3	9,38E-08	0,447092	0,186	0,132	**0,002523**	up
TMC8	9,53E-08	0,273274	0,332	0,272	**0,002565**	up
IQGAP1	9,63E-08	0,32106	0,308	0,252	**0,002591**	up
MVP	9,65E-08	0,240304	0,306	0,237	**0,002595**	up
CEBPZ	1,04E-07	-0,35823	0,258	0,295	**0,002787**	down
DBI	1,10E-07	0,157017	0,526	0,449	**0,00295**	up
PRKRA	1,10E-07	-0,52477	0,154	0,203	**0,002952**	down
DDOST	1,12E-07	0,178798	0,566	0,504	**0,003011**	up

CHCHD3	1,15E-07	0,363827	0,345	0,273	**0,003085**	up
CAPNS1	1,17E-07	0,220753	0,421	0,366	**0,003139**	up
GLTP	1,17E-07	-0,7201	0,105	0,156	**0,003157**	down
GPI	1,23E-07	0,216103	0,454	0,398	**0,003312**	up
GTF3C1	1,25E-07	0,795145	0,093	0,053	**0,003371**	up
PEPD	1,29E-07	0,302183	0,279	0,215	**0,003471**	up
NELL2	1,34E-07	-0,39431	0,22	0,285	**0,003592**	down
POLR2J3	1,36E-07	0,979205	0,077	0,04	**0,003659**	up
ZFAS1	1,37E-07	0,12895	0,805	0,753	**0,003692**	up
ID3	1,39E-07	-1,7122	0,011	0,033	**0,003727**	down
OBFC1	1,41E-07	0,295893	0,308	0,241	**0,003805**	up
TBCD	1,42E-07	0,591668	0,136	0,087	**0,003807**	up
ATRAID	1,52E-07	0,218072	0,415	0,347	**0,0041**	up
NOSIP	1,53E-07	-0,2936	0,843	0,846	**0,00411**	down
GPR65	1,54E-07	0,348468	0,356	0,29	**0,004135**	up
RP11-437B10.1	1,60E-07	-0,13749	0,27	0,256	**0,004305**	down
ACSL5	1,62E-07	0,773857	0,098	0,058	**0,004359**	up
CFH	1,65E-07	0,73815	0,104	0,062	**0,004426**	up
IL18R1	1,65E-07	0,669389	0,123	0,077	**0,004426**	up
METTL9	1,68E-07	0,218547	0,39	0,339	**0,004522**	up
CD4	1,70E-07	0,240914	0,383	0,313	**0,004578**	up
OSER1	1,72E-07	-0,21734	0,212	0,212	**0,004623**	down
B4GALT1	1,83E-07	0,524024	0,176	0,127	**0,004933**	up
DDX3Y	1,95E-07	0,366717	0,095	0,08	**0,00524**	up
PCMTD2	1,99E-07	0,509377	0,152	0,104	**0,00534**	up
LIPC	2,02E-07	-2,63293	0,004	0,018	**0,005436**	down
ACAP1	2,20E-07	0,124175	0,552	0,515	**0,005911**	up
PPP6R1	2,21E-07	0,409372	0,201	0,146	**0,00595**	up
PDE6D	2,31E-07	0,483928	0,159	0,107	**0,006224**	up
INPP4B	2,37E-07	0,238785	0,454	0,389	**0,00638**	up
MAP4K1	2,45E-07	0,33106	0,243	0,19	**0,006596**	up
TSTD1	2,52E-07	0,158601	0,586	0,527	**0,00678**	up
ME1	2,65E-07	2,252032	0,023	0,005	**0,007118**	up
ZC3H12A	2,89E-07	0,461091	0,156	0,105	**0,007766**	up
CCNG2	2,93E-07	1,179043	0,042	0,016	**0,007881**	up
DHRS3	3,02E-07	-0,68047	0,072	0,11	**0,008136**	down
ATP2B4	3,04E-07	0,433629	0,154	0,103	**0,00817**	up
TRGV5	3,18E-07	1,467436	0,045	0,019	**0,008549**	up
U2AF1	3,34E-07	0,871373	0,078	0,042	**0,008985**	up
ATP1A1	3,39E-07	0,212786	0,297	0,249	**0,009111**	up
CMTM7	3,56E-07	0,209544	0,352	0,302	**0,009569**	up

CLDND1	3,57E-07	0,41006	0,313	0,249	**0,009591**	up
MFHAS1	3,72E-07	0,46522	0,127	0,085	**0,01001**	up
HNRNPK	3,75E-07	0,114903	0,77	0,716	**0,010086**	up
NDUFV1	3,75E-07	0,238758	0,366	0,319	**0,010086**	up
STXBP2	3,95E-07	0,824078	0,091	0,053	**0,010625**	up
CPSF1	4,00E-07	0,521405	0,126	0,082	**0,010746**	up
SKAP1	4,06E-07	0,149601	0,587	0,516	**0,010932**	up
MAZ	4,25E-07	0,221657	0,462	0,403	**0,011439**	up
APMAP	4,25E-07	0,4187	0,215	0,157	**0,011441**	up
NCF4	4,60E-07	0,519611	0,16	0,111	**0,012359**	up
P2RY8	4,70E-07	0,262027	0,427	0,365	**0,012646**	up
GZMB	4,80E-07	-1,44988	0,013	0,034	**0,012922**	down
SPATA20	4,85E-07	1,239204	0,043	0,018	**0,01304**	up
AC090498.1	4,98E-07	0,120298	0,873	0,854	**0,013401**	up
09-sep	5,13E-07	0,110431	0,691	0,646	**0,013807**	up
TTC39C-AS1	5,32E-07	-0,78142	0,111	0,161	**0,014311**	down
TAF15	5,55E-07	0,346453	0,251	0,193	**0,01493**	up
PGAM1	5,90E-07	0,141852	0,572	0,527	**0,015871**	up
BTG3	6,08E-07	-0,38284	0,137	0,157	**0,016347**	down
NAA10	6,58E-07	0,524766	0,133	0,094	**0,017696**	up
CXCR3	6,78E-07	-0,49609	0,237	0,292	**0,018227**	down
NFKBIZ	6,79E-07	-0,29546	0,25	0,265	**0,018252**	down
SDF4	6,85E-07	0,257967	0,373	0,313	**0,018435**	up
STK17B	7,14E-07	0,19511	0,61	0,554	**0,019202**	up
IFIT3	7,27E-07	-1,93731	0,007	0,024	**0,019541**	down
NDUFB9	7,33E-07	0,110771	0,698	0,663	**0,019726**	up
EML3	7,50E-07	0,514532	0,131	0,089	**0,020166**	up
RP11-539L10.2	7,67E-07	0,648538	0,096	0,057	**0,020617**	up
PRDM1	7,70E-07	0,296867	0,387	0,318	**0,020704**	up
JAKMIP1	7,80E-07	1,01159	0,051	0,024	**0,020981**	up
FKBP5	7,99E-07	0,31317	0,247	0,19	**0,021492**	up
PDE4D	8,05E-07	0,196043	0,462	0,393	**0,021641**	up
ATP6V0E2	8,44E-07	0,285052	0,301	0,238	**0,022703**	up
SSR1	8,77E-07	0,313777	0,327	0,26	**0,023594**	up
HLA-DPA1	8,89E-07	0,569419	0,124	0,081	**0,023898**	up
LGALS3BP	9,58E-07	0,44174	0,153	0,105	**0,025759**	up
IGLV2-14	9,71E-07	1,253901	0,045	0,02	**0,026126**	up
MGAT4A	9,88E-07	0,104248	0,624	0,608	**0,026585**	up
KCNQ5	1,00E-06	-1,85105	0,006	0,022	**0,026993**	down
TMC6	1,00E-06	0,182916	0,449	0,387	**0,027003**	up
CXCR6	1,01E-06	1,253657	0,047	0,021	**0,027083**	up

NEDD8	1,03E-06	0,178085	0,486	0,419	**0,027805**	up
CHURC1	1,06E-06	0,265523	0,229	0,172	**0,02858**	up
DIAPH1	1,07E-06	0,188384	0,214	0,191	**0,028679**	up
CH17-373J23.1	1,07E-06	0,381926	0,154	0,105	**0,028713**	up
LINC00278	1,07E-06	4,250593	0,011	0	**0,028841**	up
TNFSF13B	1,17E-06	0,41878	0,14	0,093	**0,031566**	up
CASP1	1,20E-06	0,271285	0,293	0,235	**0,032296**	up
ANXA4	1,25E-06	0,58031	0,111	0,07	**0,033523**	up
HNRNPA3	1,27E-06	0,171488	0,549	0,499	**0,034069**	up
DSEL	1,28E-06	1,361344	0,034	0,012	**0,034515**	up
HIST1H1D	1,38E-06	0,267957	0,458	0,41	**0,037252**	up
PNP	1,39E-06	-0,53625	0,169	0,213	**0,037379**	down
SAP30	1,50E-06	0,734663	0,069	0,038	**0,040296**	up
CIR1	1,50E-06	-0,44353	0,19	0,227	**0,040304**	down
TNFRSF1B	1,51E-06	0,370149	0,187	0,141	**0,040735**	up
CMTM8	1,51E-06	-0,70504	0,126	0,177	**0,040741**	down
FBLN5	1,52E-06	-0,87423	0,062	0,098	**0,040993**	down
GHITM	1,53E-06	0,12975	0,512	0,474	**0,041192**	up
CTSC	1,56E-06	0,261258	0,347	0,3	**0,041832**	up
NUAK2	1,56E-06	-1,34478	0,019	0,043	**0,041991**	down
C17orf62	1,57E-06	0,225962	0,32	0,255	**0,042176**	up
GLG1	1,57E-06	0,316633	0,245	0,19	**0,042249**	up
ITPA	1,60E-06	0,302336	0,219	0,172	**0,042937**	up
TRAV8-4	1,65E-06	0,881958	0,077	0,049	**0,044388**	up
LL22NC03-75H12.2	1,65E-06	0,800741	0,074	0,045	**0,044424**	up
ACAA2	1,66E-06	0,521969	0,11	0,072	**0,044532**	up
IRF2BP2	1,67E-06	-0,14281	0,264	0,245	**0,044861**	down
TKT	1,83E-06	0,225898	0,413	0,345	**0,049099**	up

Supplemental Table 4: scRNA seq differentially expressed genes MS UNTR vs NTZ

rowname	p_value	avg_log2FC	pct.1	pct.2	Adj. P-value	Direction
RPS4Y1	1,24E-126	-1,0682	0,255	0,506	**3,34E-122**	down
KLF6	5,05E-125	0,667356	0,822	0,672	**1,36E-120**	up
RPS27	2,42E-113	0,170853	1	1	**6,51E-109**	up
HLA-C	1,32E-108	0,319565	0,993	0,982	**3,55E-104**	up
GIMAP7	1,25E-107	0,54216	0,804	0,692	**3,37E-103**	up
CXCR4	2,49E-103	-0,81671	0,718	0,882	**6,69E-99**	down
CD69	8,27E-100	-0,85019	0,6	0,802	**2,22E-95**	down
RPL34	1,60E-89	0,139157	1	1	**4,30E-85**	up
RPL37	2,09E-89	0,166933	0,999	1	**5,62E-85**	up
DUSP2	1,50E-86	-1,10476	0,318	0,533	**4,04E-82**	down
JUNB	2,80E-86	-0,82323	0,843	0,942	**7,53E-82**	down
RPL39	6,92E-82	0,115747	1	1	**1,86E-77**	up
RPL38	9,07E-80	0,242956	0,987	0,97	**2,44E-75**	up
RPS21	6,40E-77	0,119888	1	1	**1,72E-72**	up
TAGAP	6,10E-76	0,521253	0,775	0,649	**1,64E-71**	up
S100A11	1,07E-75	0,369303	0,86	0,788	**2,88E-71**	up
RPL36	4,60E-75	0,114648	1	1	**1,24E-70**	up
RPS29	2,77E-74	0,2405	0,996	0,99	**7,46E-70**	up
RPL41	3,58E-74	0,109068	1	1	**9,64E-70**	up
RPL35A	1,81E-72	0,113186	1	0,999	**4,88E-68**	up
DDIT4	1,58E-69	-0,88026	0,465	0,641	**4,25E-65**	down
ATP5E	3,91E-69	0,167295	0,994	0,983	**1,05E-64**	up
GIMAP4	3,32E-59	0,430243	0,649	0,526	**8,94E-55**	up
TNFAIP3	1,44E-57	-0,81934	0,523	0,68	**3,87E-53**	down
RPL22	4,34E-57	0,130564	0,997	0,993	**1,17E-52**	up
RPS4X	8,25E-56	0,134419	1	1	**2,22E-51**	up
TMA7	4,66E-55	0,161265	0,976	0,952	**1,25E-50**	up
HIST1H4C	2,62E-54	0,436257	0,77	0,672	**7,05E-50**	up
RPS26	7,25E-51	0,309896	0,991	0,982	**1,95E-46**	up
LDHB	6,48E-50	0,13543	0,986	0,96	**1,74E-45**	up
HSPA8	2,57E-49	0,157308	0,973	0,953	**6,91E-45**	up
EEF2	1,96E-48	0,131035	0,993	0,986	**5,28E-44**	up
RPL37A	3,56E-48	0,162945	0,983	0,97	**9,58E-44**	up
PDE4D	6,29E-46	-0,90346	0,312	0,462	**1,69E-41**	down
RHOB	3,07E-42	1,739581	0,115	0,036	**8,25E-38**	up
IL7R	2,45E-39	0,12045	0,992	0,99	**6,59E-35**	up
TRAF3IP3	7,00E-38	0,233243	0,73	0,637	**1,88E-33**	up
CD2	1,11E-37	0,269319	0,726	0,61	**2,99E-33**	up
IL2RG	1,49E-35	0,133507	0,932	0,89	**4,01E-31**	up

GLTSCR2	4,11E-35	0,144596	0,931	0,883	**1,11E-30**	up
NOSIP	5,10E-33	0,175665	0,892	0,843	**1,37E-28**	up
AC090498.1	7,75E-33	0,156346	0,934	0,873	**2,08E-28**	up
TUBA1B	2,45E-32	0,30709	0,586	0,473	**6,59E-28**	up
CD52	2,69E-32	0,101901	0,975	0,956	**7,24E-28**	up
LIMD2	5,12E-32	0,120866	0,935	0,899	**1,38E-27**	up
ZFP36L2	2,54E-31	-0,45599	0,95	0,969	**6,84E-27**	down
TNFAIP8L2	2,88E-31	1,480106	0,093	0,031	**7,73E-27**	up
FYB	1,68E-29	0,203277	0,781	0,703	**4,51E-25**	up
RPL13A	6,21E-29	0,129045	0,959	0,923	**1,67E-24**	up
AQP3	1,50E-28	0,213007	0,776	0,697	**4,03E-24**	up
TUBB	2,49E-28	0,303808	0,574	0,474	**6,69E-24**	up
TAGLN2	1,48E-27	0,143097	0,919	0,893	**3,98E-23**	up
RPL17	4,32E-27	0,100645	0,943	0,909	**1,16E-22**	up
ARL4C	9,87E-27	-0,57043	0,675	0,733	**2,66E-22**	down
LBH	7,28E-26	0,287037	0,479	0,382	**1,96E-21**	up
HLA-F	7,40E-26	0,210301	0,681	0,587	**1,99E-21**	up
GPR183	1,33E-25	0,410909	0,399	0,297	**3,56E-21**	up
STAT1	1,41E-25	0,60496	0,318	0,239	**3,79E-21**	up
FAM46C	1,61E-25	-0,94287	0,174	0,271	**4,32E-21**	down
NKG7	1,64E-25	-0,97718	0,236	0,339	**4,42E-21**	down
RGCC	1,72E-25	0,55436	0,317	0,224	**4,64E-21**	up
BHLHE40	1,73E-25	-0,83379	0,248	0,352	**4,66E-21**	down
USMG5	1,87E-25	0,167113	0,743	0,649	**5,03E-21**	up
CD27	2,93E-25	0,269563	0,577	0,484	**7,88E-21**	up
HNRNPF	3,79E-25	0,17531	0,646	0,552	**1,02E-20**	up
SYTL3	5,00E-25	-0,84097	0,208	0,305	**1,34E-20**	down
PTPN6	5,94E-25	0,364375	0,389	0,295	**1,60E-20**	up
S1PR1	9,70E-25	0,309536	0,461	0,369	**2,61E-20**	up
UXT	1,49E-24	0,130416	0,812	0,753	**4,02E-20**	up
SON	1,82E-24	0,152746	0,768	0,699	**4,89E-20**	up
CISH	6,53E-24	0,549283	0,246	0,174	**1,76E-19**	up
ARL6IP5	7,16E-24	0,106567	0,857	0,792	**1,93E-19**	up
UBXN1	8,14E-24	0,128705	0,772	0,718	**2,19E-19**	up
CH17-373J23.1	1,76E-23	0,70109	0,237	0,154	**4,73E-19**	up
RPL36A	2,21E-23	0,129997	0,902	0,85	**5,94E-19**	up
EIF3L	3,50E-23	0,125129	0,851	0,795	**9,41E-19**	up
LFNG	4,47E-23	0,638199	0,217	0,14	**1,20E-18**	up
PLEKHA7	7,18E-23	0,59608	0,236	0,149	**1,93E-18**	up
TCF7	9,73E-23	0,147409	0,806	0,73	**2,62E-18**	up
CCR7	1,35E-22	0,531558	0,332	0,246	**3,63E-18**	up

LINC00861	1,48E-22	0,235372	0,582	0,483	**3,98E-18**	up
CXCR3	2,15E-22	0,414859	0,313	0,237	**5,77E-18**	up
ATP5I	2,70E-22	0,21825	0,628	0,534	**7,26E-18**	up
DUSP1	3,52E-21	-0,54174	0,645	0,732	**9,48E-17**	down
ITGA4	4,15E-21	0,209049	0,638	0,553	**1,12E-16**	up
GIMAP1	7,41E-21	0,593782	0,216	0,138	**1,99E-16**	up
RPS10	1,00E-20	0,187191	0,794	0,726	**2,70E-16**	up
EIF2S3	1,39E-20	0,232514	0,593	0,493	**3,74E-16**	up
ZFP36	3,98E-20	-0,57088	0,69	0,752	**1,07E-15**	down
S1PR4	4,34E-20	0,233796	0,554	0,462	**1,17E-15**	up
TBC1D10C	4,45E-20	0,134936	0,666	0,598	**1,20E-15**	up
SELPLG	5,08E-20	0,121648	0,685	0,603	**1,37E-15**	up
ARPC1B	5,20E-20	0,121029	0,755	0,697	**1,40E-15**	up
GZMA	5,38E-20	-0,78159	0,274	0,366	**1,45E-15**	down
AC006129.2	7,89E-20	0,355275	0,379	0,292	**2,12E-15**	up
PSMB8	2,30E-19	0,100222	0,69	0,614	**6,19E-15**	up
CDK2AP2	2,67E-19	0,37096	0,286	0,204	**7,19E-15**	up
C19orf43	6,57E-19	0,102619	0,781	0,726	**1,77E-14**	up
FCMR	6,78E-19	0,196076	0,56	0,472	**1,82E-14**	up
EZR	1,22E-18	0,157139	0,673	0,599	**3,29E-14**	up
IRF1	1,64E-18	0,294044	0,465	0,38	**4,41E-14**	up
CSK	1,72E-18	0,180896	0,562	0,484	**4,63E-14**	up
LIME1	1,99E-18	0,183614	0,595	0,516	**5,35E-14**	up
ICAM2	2,05E-18	0,149235	0,543	0,453	**5,51E-14**	up
TNFRSF14	2,56E-18	0,229563	0,536	0,448	**6,88E-14**	up
CRIP2	3,22E-18	0,486534	0,3	0,222	**8,67E-14**	up
CRIP1	3,28E-18	0,168498	0,693	0,62	**8,83E-14**	up
SLC9A3R1	7,76E-18	0,128965	0,583	0,494	**2,09E-13**	up
ITGB7	1,06E-17	0,241722	0,469	0,386	**2,85E-13**	up
UCP2	1,21E-17	0,10995	0,767	0,718	**3,25E-13**	up
LDLRAP1	1,22E-17	0,332276	0,362	0,276	**3,27E-13**	up
YWHAH	1,29E-17	0,218094	0,487	0,414	**3,47E-13**	up
BTN3A2	1,75E-17	0,397027	0,307	0,226	**4,70E-13**	up
MYC	1,85E-17	0,25053	0,498	0,412	**4,98E-13**	up
SEC62	2,35E-17	0,21031	0,537	0,454	**6,32E-13**	up
MYBL1	2,60E-17	-0,66604	0,268	0,354	**6,98E-13**	down
CDC42SE1	3,34E-17	0,212847	0,515	0,436	**8,99E-13**	up
09-sep	3,94E-17	0,100459	0,758	0,691	**1,06E-12**	up
EIF2S2	4,48E-17	0,32711	0,365	0,279	**1,21E-12**	up
GIMAP5	5,12E-17	0,531451	0,192	0,124	**1,38E-12**	up
MYADM	1,44E-16	0,595063	0,184	0,121	**3,88E-12**	up

TRADD	1,50E-16	0,145304	0,655	0,577	**4,04E-12**	up
DYNLL1	1,63E-16	0,287626	0,335	0,259	**4,39E-12**	up
FOSL2	1,75E-16	-1,15329	0,061	0,114	**4,72E-12**	down
SMDT1	3,45E-16	0,222507	0,639	0,556	**9,28E-12**	up
PLP2	4,19E-16	0,137931	0,692	0,616	**1,13E-11**	up
C14orf119	4,78E-16	0,43768	0,198	0,135	**1,29E-11**	up
NDUFA3	5,18E-16	0,145144	0,603	0,525	**1,39E-11**	up
SAMHD1	5,62E-16	0,134336	0,615	0,549	**1,51E-11**	up
PIM2	1,21E-15	0,208873	0,402	0,336	**3,26E-11**	up
RAB1B	1,32E-15	0,311487	0,313	0,237	**3,56E-11**	up
CITED2	1,66E-15	0,215789	0,485	0,402	**4,48E-11**	up
NR4A2	1,78E-15	-0,99006	0,103	0,161	**4,77E-11**	down
TWF2	2,02E-15	0,225415	0,42	0,35	**5,43E-11**	up
ATP2B1	2,74E-15	0,36227	0,309	0,234	**7,38E-11**	up
TMBIM1	2,91E-15	0,324533	0,301	0,232	**7,82E-11**	up
TUBA1A	4,17E-15	0,227764	0,479	0,405	**1,12E-10**	up
TOB1	5,14E-15	-0,56891	0,324	0,4	**1,38E-10**	down
MID1IP1	5,34E-15	0,565665	0,147	0,09	**1,44E-10**	up
DDX3Y	7,11E-15	-1,21321	0,05	0,095	**1,91E-10**	down
NCR3	1,05E-14	-0,97623	0,119	0,175	**2,84E-10**	down
RGS14	1,06E-14	0,221387	0,369	0,3	**2,85E-10**	up
GZMK	1,30E-14	-0,53042	0,289	0,37	**3,49E-10**	down
AMBRA1	1,31E-14	0,321493	0,334	0,255	**3,51E-10**	up
PPP1R15A	1,41E-14	0,320918	0,249	0,196	**3,79E-10**	up
NEAT1	1,80E-14	0,286587	0,363	0,291	**4,84E-10**	up
DGKA	1,94E-14	0,22956	0,426	0,348	**5,23E-10**	up
C11orf68	2,01E-14	0,522409	0,161	0,102	**5,41E-10**	up
SIT1	2,09E-14	0,235002	0,346	0,278	**5,63E-10**	up
LAT	2,19E-14	0,105859	0,656	0,602	**5,90E-10**	up
DUSP5	2,46E-14	-0,70871	0,141	0,207	**6,63E-10**	down
LIMS1	3,20E-14	0,204207	0,431	0,351	**8,61E-10**	up
CSRNP1	3,24E-14	-0,83649	0,153	0,216	**8,72E-10**	down
C16orf54	4,33E-14	0,382404	0,243	0,173	**1,16E-09**	up
PSIP1	4,96E-14	0,133328	0,566	0,487	**1,33E-09**	up
STMN3	7,43E-14	0,208162	0,434	0,359	**2,00E-09**	up
GDI2	7,90E-14	0,121826	0,543	0,478	**2,13E-09**	up
PKM	8,75E-14	0,105322	0,69	0,615	**2,35E-09**	up
RP5-1171I10.5	9,49E-14	0,44362	0,209	0,146	**2,55E-09**	up
UBE2L6	9,80E-14	0,159141	0,458	0,383	**2,64E-09**	up
SCML4	1,01E-13	0,274832	0,378	0,305	**2,72E-09**	up
TUBB4B	1,29E-13	0,315069	0,265	0,196	**3,46E-09**	up

ADGRE5	1,50E-13	0,195285	0,462	0,392	**4,03E-09**	up
ILK	1,96E-13	0,291108	0,249	0,189	**5,28E-09**	up
TAP1	2,61E-13	0,201133	0,392	0,324	**7,03E-09**	up
GIMAP2	3,19E-13	0,292549	0,279	0,211	**8,59E-09**	up
FBXW5	3,24E-13	0,180312	0,459	0,386	**8,73E-09**	up
SOCS3	3,42E-13	-0,74412	0,182	0,25	**9,19E-09**	down
TNFRSF25	4,48E-13	0,21574	0,383	0,314	**1,21E-08**	up
ZMYM6NB	4,65E-13	0,259172	0,3	0,239	**1,25E-08**	up
CBLL1	4,98E-13	0,260534	0,364	0,295	**1,34E-08**	up
PNP	6,10E-13	0,259684	0,218	0,169	**1,64E-08**	up
SNHG8	6,79E-13	0,10953	0,714	0,643	**1,83E-08**	up
DNAJA1	6,88E-13	0,117635	0,43	0,378	**1,85E-08**	up
ST13	7,50E-13	0,114503	0,625	0,564	**2,02E-08**	up
CDC25B	8,07E-13	0,214542	0,427	0,357	**2,17E-08**	up
FOS	1,16E-12	-0,1463	0,404	0,451	**3,12E-08**	down
DPP7	1,42E-12	0,100929	0,562	0,5	**3,82E-08**	up
ATP5H	1,68E-12	0,108008	0,645	0,578	**4,51E-08**	up
LEF1	2,29E-12	0,239046	0,393	0,325	**6,16E-08**	up
TECR	2,32E-12	0,104293	0,572	0,512	**6,24E-08**	up
MAP3K8	2,84E-12	-0,92124	0,08	0,129	**7,64E-08**	down
GIMAP6	3,01E-12	0,43219	0,168	0,114	**8,09E-08**	up
PA2G4	3,10E-12	0,147756	0,502	0,422	**8,35E-08**	up
CNPPD1	3,33E-12	0,288897	0,284	0,215	**8,96E-08**	up
ID3	3,84E-12	1,519343	0,034	0,011	**1,03E-07**	up
HIST1H1D	4,59E-12	-0,14181	0,411	0,458	**1,23E-07**	down
PDE4B	4,80E-12	-0,75289	0,184	0,246	**1,29E-07**	down
GPR65	4,91E-12	-0,57018	0,282	0,356	**1,32E-07**	down
SLAMF1	5,24E-12	0,367094	0,186	0,132	**1,41E-07**	up
RUNX3	5,46E-12	-0,46794	0,421	0,471	**1,47E-07**	down
AP1G2	5,45E-12	0,286036	0,273	0,206	**1,47E-07**	up
FOSB	5,61E-12	0,623723	0,098	0,058	**1,51E-07**	up
ITK	5,64E-12	0,191047	0,412	0,349	**1,52E-07**	up
TBCA	5,78E-12	0,115273	0,584	0,518	**1,55E-07**	up
MAZ	6,32E-12	0,139158	0,531	0,462	**1,70E-07**	up
NOP10	6,36E-12	0,221442	0,322	0,262	**1,71E-07**	up
KRAS	6,39E-12	0,280525	0,309	0,24	**1,72E-07**	up
ITGB1	6,51E-12	0,241171	0,519	0,451	**1,75E-07**	up
CCR6	7,18E-12	-0,47167	0,337	0,378	**1,93E-07**	down
CYTH1	7,65E-12	0,11763	0,541	0,476	**2,06E-07**	up
TESPA1	8,82E-12	0,213761	0,377	0,31	**2,37E-07**	up
IGBP1	9,16E-12	0,128099	0,486	0,416	**2,46E-07**	up

NDUFB1	9,33E-12	0,19253	0,426	0,355	**2,51E-07**	up
HAX1	1,01E-11	0,204467	0,408	0,337	**2,70E-07**	up
TCF25	1,02E-11	0,183169	0,474	0,408	**2,74E-07**	up
PSPH	1,16E-11	1,2303	0,05	0,021	**3,12E-07**	up
RP1-111C20.4	1,42E-11	0,635916	0,098	0,058	**3,81E-07**	up
MAL	1,68E-11	0,129357	0,587	0,513	**4,51E-07**	up
DNAJB1	1,85E-11	-0,41105	0,637	0,699	**4,97E-07**	down
RCSD1	1,98E-11	0,135618	0,512	0,443	**5,34E-07**	up
IRF3	2,01E-11	0,287843	0,255	0,203	**5,40E-07**	up
MPHOSPH8	2,02E-11	0,106469	0,506	0,445	**5,43E-07**	up
SPSB3	2,13E-11	0,119928	0,564	0,49	**5,73E-07**	up
MT2A	3,23E-11	-0,45215	0,339	0,413	**8,68E-07**	down
NDUFC1	3,28E-11	0,122735	0,485	0,415	**8,83E-07**	up
SNHG7	4,40E-11	0,190492	0,428	0,365	**1,18E-06**	up
DEF6	5,24E-11	0,125706	0,418	0,357	**1,41E-06**	up
GBP4	5,41E-11	0,329474	0,192	0,136	**1,45E-06**	up
DEK	7,03E-11	0,104713	0,555	0,488	**1,89E-06**	up
RPA2	7,51E-11	0,165201	0,339	0,274	**2,02E-06**	up
ZNF706	7,68E-11	0,151544	0,441	0,372	**2,07E-06**	up
FAM102A	7,92E-11	0,288998	0,263	0,202	**2,13E-06**	up
TUBA4A	8,32E-11	0,168174	0,433	0,362	**2,24E-06**	up
C19orf66	9,14E-11	0,143045	0,437	0,373	**2,46E-06**	up
LEPROTL1	9,23E-11	-0,3467	0,716	0,766	**2,48E-06**	down
GZMH	9,26E-11	-0,97339	0,026	0,054	**2,49E-06**	down
ARFRP1	9,47E-11	0,347695	0,186	0,132	**2,55E-06**	up
HSPB1	9,68E-11	0,163146	0,481	0,408	**2,60E-06**	up
GADD45B	1,18E-10	0,152125	0,431	0,378	**3,16E-06**	up
HMOX2	1,27E-10	0,12338	0,442	0,381	**3,41E-06**	up
ZRSR2	1,47E-10	0,397702	0,192	0,137	**3,94E-06**	up
BRD2	1,46E-10	0,216877	0,272	0,216	**3,94E-06**	up
CDK9	1,49E-10	0,351573	0,173	0,121	**4,00E-06**	up
TNF	1,62E-10	0,617776	0,099	0,06	**4,36E-06**	up
EIF1AX	1,82E-10	0,227612	0,399	0,33	**4,90E-06**	up
KLHDC3	1,87E-10	0,421697	0,174	0,125	**5,02E-06**	up
PER1	2,03E-10	-0,66196	0,124	0,169	**5,46E-06**	down
JUN	2,16E-10	0,151597	0,54	0,515	**5,81E-06**	up
TMEM63A	2,44E-10	0,386011	0,183	0,132	**6,56E-06**	up
TMEM107	2,67E-10	0,43555	0,151	0,104	**7,19E-06**	up
RPS20	2,73E-10	0,131802	0,553	0,494	**7,35E-06**	up
CARD16	3,13E-10	0,19205	0,347	0,292	**8,42E-06**	up
DENND2D	3,30E-10	0,124641	0,351	0,299	**8,87E-06**	up

FDFT1	3,51E-10	0,148729	0,407	0,342	**9,45E-06**	up
PPT1	3,65E-10	0,226628	0,26	0,207	**9,81E-06**	up
ANXA2R	4,02E-10	0,231763	0,284	0,228	**1,08E-05**	up
STK17B	4,37E-10	-0,44486	0,554	0,61	**1,18E-05**	down
MTCH1	4,43E-10	0,154732	0,387	0,321	**1,19E-05**	up
GBP2	4,78E-10	0,192695	0,285	0,231	**1,29E-05**	up
CST7	5,28E-10	-0,58804	0,262	0,327	**1,42E-05**	down
HSPD1	5,37E-10	0,145769	0,438	0,375	**1,44E-05**	up
PIK3IP1	5,86E-10	0,103992	0,525	0,453	**1,58E-05**	up
YWHAE	6,22E-10	0,286421	0,201	0,153	**1,67E-05**	up
SNHG12	6,51E-10	0,267705	0,221	0,17	**1,75E-05**	up
SRGN	8,73E-10	-0,40733	0,658	0,712	**2,35E-05**	down
DCTN2	8,89E-10	0,171875	0,349	0,288	**2,39E-05**	up
PAQR8	1,05E-09	0,742679	0,066	0,036	**2,83E-05**	up
RPS6KA1	1,07E-09	0,273614	0,244	0,186	**2,88E-05**	up
CA5B	1,23E-09	0,224945	0,369	0,317	**3,30E-05**	up
GNLY	1,47E-09	-1,08051	0,077	0,112	**3,94E-05**	down
FAM89B	1,60E-09	0,133686	0,262	0,228	**4,31E-05**	up
ING1	1,91E-09	0,508272	0,12	0,078	**5,14E-05**	up
GSTM1	2,10E-09	0,661436	0,096	0,058	**5,66E-05**	up
CH507-528H12.1	2,22E-09	0,423774	0,132	0,091	**5,98E-05**	up
SUSD3	2,23E-09	0,201593	0,282	0,228	**6,01E-05**	up
AC004556.1	2,25E-09	1,644169	0,025	0,008	**6,06E-05**	up
SERINC5	2,90E-09	0,20065	0,32	0,258	**7,79E-05**	up
MKNK2	3,04E-09	0,173542	0,383	0,321	**8,17E-05**	up
C9orf69	3,07E-09	0,31004	0,153	0,11	**8,27E-05**	up
PIK3R1	3,49E-09	-0,46549	0,426	0,491	**9,38E-05**	down
TRAT1	3,66E-09	0,124008	0,4	0,344	**9,85E-05**	up
DOK2	3,78E-09	0,159903	0,351	0,29	**1,02E-04**	up
IL27RA	3,85E-09	0,204857	0,269	0,211	**1,03E-04**	up
MAP3K1	4,10E-09	0,265651	0,206	0,156	**1,10E-04**	up
MAML2	4,16E-09	0,211862	0,344	0,29	**1,12E-04**	up
DYNLT1	4,51E-09	-0,38634	0,436	0,491	**1,21E-04**	down
CLN3	4,64E-09	0,271024	0,169	0,128	**1,25E-04**	up
PRKCQ	4,71E-09	0,236332	0,295	0,24	**1,27E-04**	up
TMEM256	5,33E-09	0,156918	0,359	0,303	**1,43E-04**	up
MRPS26	5,45E-09	0,204797	0,261	0,205	**1,47E-04**	up
PCID2	5,53E-09	0,294305	0,193	0,142	**1,49E-04**	up
EGLN2	5,95E-09	0,551561	0,088	0,054	**1,60E-04**	up
APOA1BP	6,47E-09	0,203398	0,322	0,263	**1,74E-04**	up
EPSTI1	6,58E-09	0,603817	0,115	0,077	**1,77E-04**	up

GSTM2	7,09E-09	0,552045	0,096	0,06	**1,91E-04**	up
ZNF331	7,25E-09	-0,67181	0,135	0,181	**1,95E-04**	down
TMEM256-PLSCR3	7,28E-09	0,175858	0,361	0,299	**1,96E-04**	up
MRPS5	8,00E-09	0,228075	0,222	0,169	**2,15E-04**	up
OSM	8,26E-09	-0,98935	0,055	0,087	**2,22E-04**	down
09-mrt	8,69E-09	0,185457	0,286	0,227	**2,34E-04**	up
FAM50A	9,10E-09	0,114479	0,327	0,268	**2,45E-04**	up
HADHA	9,55E-09	0,101563	0,451	0,391	**2,57E-04**	up
AREG	1,06E-08	-1,0515	0,049	0,079	**2,86E-04**	down
ARHGAP25	1,09E-08	0,22243	0,233	0,187	**2,94E-04**	up
UTY	1,14E-08	-0,89562	0,048	0,08	**3,07E-04**	down
VTA1	1,15E-08	0,319741	0,146	0,102	**3,10E-04**	up
VPS35	1,33E-08	0,320473	0,209	0,159	**3,58E-04**	up
WSB1	1,35E-08	0,184053	0,267	0,22	**3,64E-04**	up
RBM38	1,40E-08	-0,41302	0,367	0,424	**3,77E-04**	down
GADD45A	1,51E-08	-0,93769	0,054	0,085	**4,06E-04**	down
NAA60	1,64E-08	0,324772	0,13	0,089	**4,40E-04**	up
MITD1	1,71E-08	0,308428	0,181	0,133	**4,61E-04**	up
RHOT2	1,73E-08	0,227969	0,245	0,19	**4,66E-04**	up
MEA1	1,77E-08	0,134182	0,321	0,264	**4,76E-04**	up
HIST1H1E	1,83E-08	-0,19434	0,65	0,696	**4,93E-04**	down
MED16	1,90E-08	0,32012	0,185	0,14	**5,12E-04**	up
MGAT1	2,10E-08	0,197459	0,279	0,223	**5,65E-04**	up
ANXA5	2,13E-08	0,10998	0,435	0,374	**5,74E-04**	up
MVP	2,48E-08	0,161972	0,362	0,306	**6,68E-04**	up
S1PR5	2,58E-08	-1,12307	0,026	0,051	**6,94E-04**	down
TEX14	2,59E-08	0,401748	0,196	0,157	**6,96E-04**	up
FLI1	2,74E-08	0,114347	0,4	0,338	**7,37E-04**	up
TMEM187	2,81E-08	0,664104	0,059	0,033	**7,55E-04**	up
ID2	2,95E-08	-0,59654	0,154	0,204	**7,93E-04**	down
APBB1	3,08E-08	0,332091	0,161	0,116	**8,28E-04**	up
METTL12	3,16E-08	0,450276	0,099	0,066	**8,50E-04**	up
BBC3	3,51E-08	0,491514	0,113	0,076	**9,45E-04**	up
LRP10	3,64E-08	0,13593	0,385	0,33	**9,79E-04**	up
TM9SF2	4,14E-08	0,133643	0,324	0,267	**0,001114**	up
RSBN1L	4,30E-08	0,15071	0,252	0,205	**0,001157**	up
TRAV9-2	4,47E-08	0,331079	0,085	0,084	**0,001201**	up
PSMB8-AS1	4,51E-08	0,115698	0,325	0,27	**0,001214**	up
TRIM28	5,04E-08	0,165722	0,33	0,271	**0,001356**	up
MRFAP1L1	5,10E-08	0,1294	0,259	0,213	**0,001372**	up
THOC3	5,27E-08	0,190717	0,299	0,248	**0,001418**	up

MTFP1	5,62E-08	-0,59584	0,151	0,198	**0,001512**	down
EIF1AY	5,63E-08	-1,13892	0,031	0,056	**0,001515**	down
MUS81	5,80E-08	0,433648	0,116	0,079	**0,001561**	up
TRPV2	5,94E-08	0,235278	0,201	0,153	**0,001597**	up
EEF1G	5,95E-08	0,218835	0,269	0,215	**0,0016**	up
CCL5	6,02E-08	-0,15229	0,514	0,561	**0,00162**	down
TTTY15	6,05E-08	-0,98284	0,035	0,062	**0,001628**	down
RASSF7	6,16E-08	0,181695	0,274	0,22	**0,001658**	up
HERPUD1	6,23E-08	-0,41569	0,354	0,403	**0,001676**	down
GBP1	6,74E-08	0,419342	0,132	0,095	**0,001814**	up
RP11-124N14.3	7,14E-08	0,132393	0,373	0,333	**0,001921**	up
TRIM69	7,22E-08	0,33008	0,137	0,097	**0,001943**	up
SLC25A29	7,43E-08	0,605077	0,074	0,045	**0,001998**	up
SRPK2	7,47E-08	0,134057	0,353	0,298	**0,00201**	up
TMCO1	7,79E-08	0,150385	0,272	0,218	**0,002094**	up
C12orf65	9,94E-08	0,314389	0,156	0,113	**0,002674**	up
NBPF15	1,01E-07	0,415539	0,125	0,088	**0,002715**	up
VAT1	1,02E-07	0,353696	0,155	0,114	**0,002746**	up
RP11-511B23.2	1,09E-07	0,647712	0,085	0,055	**0,002936**	up
PMPCB	1,13E-07	0,165061	0,323	0,269	**0,003043**	up
STK38	1,21E-07	0,122785	0,33	0,295	**0,003248**	up
CCM2	1,26E-07	0,11857	0,353	0,298	**0,003382**	up
HGS	1,35E-07	0,265292	0,169	0,129	**0,003641**	up
CCDC97	1,36E-07	0,403121	0,111	0,077	**0,003651**	up
SARS	1,37E-07	0,115842	0,291	0,239	**0,003675**	up
RNF5	1,39E-07	0,16084	0,212	0,165	**0,003744**	up
MRPL18	1,45E-07	0,197495	0,214	0,169	**0,003888**	up
RPP21	1,59E-07	0,278535	0,183	0,141	**0,004281**	up
DNAJB2	1,65E-07	0,355097	0,12	0,087	**0,004432**	up
TRNAU1AP	1,68E-07	0,296858	0,133	0,097	**0,00452**	up
CALHM2	1,79E-07	0,214731	0,131	0,094	**0,004816**	up
IGLV2-14	1,82E-07	-1,09199	0,023	0,045	**0,004904**	down
BTG2	1,83E-07	-0,42703	0,36	0,412	**0,004924**	down
PURA	2,13E-07	0,162074	0,272	0,222	**0,005742**	up
PYGO2	2,37E-07	0,428283	0,094	0,062	**0,006379**	up
CCNL2	2,40E-07	0,238181	0,174	0,13	**0,006466**	up
UNG	2,49E-07	0,432411	0,098	0,065	**0,006705**	up
CLEC2B	2,65E-07	-0,40527	0,47	0,526	**0,007134**	down
AC006369.2	2,74E-07	0,19734	0,307	0,255	**0,007357**	up
CEBPD	2,82E-07	-0,59805	0,132	0,175	**0,007583**	down

CHURC1	2,91E-07	0,183031	0,272	0,229	**0,007815**	up
MFSD10	2,92E-07	0,135062	0,349	0,294	**0,007864**	up
IFITM3	2,96E-07	0,162106	0,381	0,33	**0,007974**	up
FAM45A	3,18E-07	0,394427	0,128	0,09	**0,008541**	up
HIST1H2BG	3,30E-07	0,93113	0,032	0,014	**0,008872**	up
SLC35F1	3,33E-07	0,748609	0,057	0,032	**0,008949**	up
ARHGEF7	3,54E-07	-0,65287	0,099	0,124	**0,009524**	down
LYL1	3,59E-07	0,876912	0,042	0,021	**0,009663**	up
MRPL51	3,61E-07	0,119717	0,281	0,233	**0,009703**	up
TMEM204	3,72E-07	0,252571	0,215	0,169	**0,010018**	up
AP1S2	3,74E-07	0,238385	0,253	0,204	**0,010057**	up
RPP38	3,88E-07	0,406464	0,107	0,074	**0,01044**	up
TNFSF13B	3,99E-07	0,310122	0,172	0,14	**0,010738**	up
NSUN5	4,01E-07	0,293069	0,179	0,136	**0,010791**	up
MRPS18C	4,26E-07	0,235207	0,191	0,147	**0,011469**	up
MOB3A	4,45E-07	0,315709	0,151	0,111	**0,01197**	up
CREM	4,45E-07	-0,59239	0,147	0,191	**0,011973**	down
TRIM21	4,79E-07	0,419165	0,093	0,062	**0,012887**	up
METTL17	4,83E-07	0,219849	0,169	0,129	**0,012993**	up
FTH1	5,05E-07	-0,24971	0,997	0,999	**0,013576**	down
THUMPD3-AS1	5,09E-07	0,291794	0,183	0,14	**0,013692**	up
NDUFAF3	5,25E-07	0,121968	0,347	0,299	**0,014108**	up
GTF2F1	5,43E-07	0,234365	0,241	0,197	**0,01461**	up
NAP1L4	5,80E-07	0,103452	0,389	0,335	**0,015613**	up
PLEKHO1	6,07E-07	0,213937	0,228	0,18	**0,016328**	up
SRSF4	6,08E-07	0,141999	0,295	0,246	**0,01636**	up
USP10	6,14E-07	0,171678	0,399	0,346	**0,016525**	up
POLD4	6,24E-07	0,116069	0,381	0,332	**0,01679**	up
SGK223	6,54E-07	0,619139	0,056	0,032	**0,017587**	up
SPOP	7,03E-07	0,271001	0,15	0,113	**0,018903**	up
VTI1B	7,21E-07	0,184127	0,234	0,186	**0,019404**	up
NUAK2	7,38E-07	0,902266	0,038	0,019	**0,019856**	up
GALT	7,53E-07	0,216479	0,172	0,134	**0,020258**	up
TIMM8B	7,64E-07	0,246397	0,181	0,14	**0,020561**	up
LINC00936	7,75E-07	0,374108	0,142	0,106	**0,020838**	up
BLOC1S1	7,75E-07	0,176932	0,236	0,189	**0,020844**	up
SNHG25	7,92E-07	0,152135	0,304	0,268	**0,021309**	up
HIBCH	8,24E-07	0,497457	0,086	0,057	**0,022171**	up
OGFR	8,26E-07	0,169174	0,23	0,184	**0,022225**	up
MARCKSL1	8,43E-07	0,294638	0,154	0,114	**0,022688**	up
UTP6	8,99E-07	0,291532	0,101	0,069	**0,02419**	up

GIMAP8	9,40E-07	0,483268	0,077	0,049	**0,025287**	up
PYCARD	9,46E-07	0,140513	0,275	0,228	**0,02544**	up
NRBP1	9,57E-07	0,21119	0,231	0,188	**0,025741**	up
SH3BP5	1,05E-06	0,140779	0,374	0,324	**0,028244**	up
PTOV1	1,08E-06	0,153709	0,303	0,257	**0,028916**	up
HIST2H2BF	1,09E-06	0,43389	0,065	0,04	**0,029276**	up
PRKCA	1,09E-06	0,222846	0,229	0,185	**0,029365**	up
TNFRSF1A	1,11E-06	0,362629	0,117	0,082	**0,029963**	up
LGALS3	1,12E-06	-0,44154	0,359	0,415	**0,030126**	down
SAMD1	1,21E-06	0,382337	0,09	0,062	**0,032466**	up
TMEM140	1,24E-06	0,774787	0,045	0,024	**0,033281**	up
CFAP36	1,24E-06	0,157137	0,271	0,222	**0,03332**	up
GPR25	1,25E-06	0,699833	0,065	0,042	**0,033639**	up
DBP	1,28E-06	0,164569	0,223	0,178	**0,034347**	up
MRPL46	1,37E-06	0,360319	0,117	0,083	**0,036753**	up
SIMC1	1,37E-06	0,547739	0,067	0,041	**0,036868**	up
RP11-160E2.6	1,39E-06	0,279964	0,137	0,102	**0,037506**	up
PDE7A	1,49E-06	0,103672	0,317	0,273	**0,040055**	up
SPN	1,51E-06	0,126473	0,308	0,262	**0,040741**	up
RNF44	1,57E-06	0,180578	0,222	0,18	**0,042344**	up
PIH1D1	1,59E-06	0,121821	0,273	0,224	**0,042672**	up
TMEM173	1,63E-06	0,102788	0,342	0,292	**0,043961**	up
MMD	1,67E-06	-0,84714	0,045	0,069	**0,04501**	down
EXOSC6	1,84E-06	-0,46707	0,19	0,233	**0,049587**	down

Supplemental Table 5: Overlapping differentially expressed genes

	Genes
1	HLA-C
2	SMDT1
3	RPS4Y1
4	CXCR4
5	ZFP36L2
6	GIMAP7
7	TOB1
8	ARPC1B
9	RPS10
10	GSTM1
11	S100A11
12	CISH
13	GZMK
14	SOCS3
15	DUSP2
16	DUSP1
17	GPR183
18	JUNB
19	DNAJB1
20	CST7
21	NKG7
22	IL2RG
23	GZMA
24	MYBL1
25	MYC
26	LIME1
27	EEF2
28	EZR
29	HERPUD1
30	GIMAP4
31	DDIT4
32	RPL17
33	SRGN
34	RPS29
35	ARL4C
36	TNFAIP3
37	RPS27
38	TRADD
39	RPL36A

40	ATP5E
41	GNLY
42	UCP2
43	CEBPD
44	AMBRA1
45	CCR6
46	GZMH
47	SNHG8
48	TNFAIP8L2
49	LGALS3
50	DNAJA1
51	TMEM256-PLSCR3
52	CD52
53	PSPH
54	RBM38
55	CCL5
56	RP11-124N14.3
57	CD69
58	C14orf119
59	NDUFA3
60	PIM2
61	IFITM3
62	DYNLT1
63	ADGRE5
64	SAMHD1
65	MTFP1
66	TAGLN2
67	HLA-F
68	RGCC
69	DYNLL1
70	CRIP2
71	DENND2D
72	FYB
73	RHOB
74	ANXA5
75	ZFP36
76	BHLHE40
77	CCR7
78	PKM
79	SIT1
80	JUN

81	EIF1AY
82	PLP2
83	CRIP1
84	LEPROTL1
85	PIK3IP1
86	SNHG25
87	STAT1
88	MVP
89	ID3
90	NOSIP
91	GPR65
92	DDX3Y
93	MAZ
94	AC090498.1
95	SEPT9
96	CXCR3
97	STK17B
98	PDE4D
99	IGLV2-14
100	CHURC1
101	CH17-373J23.1
102	TNFSF13B
103	HIST1H1D
104	PNP
105	NUAK2

Supplemental Table 6: scRNA seq annotation

Gene	p-value	avg_log2FC	adj. p-value	cluster
**RPL6**	0	0,28341475	0	1
**RPL11**	0	0,19458502	0	1
**RPS3A**	0	0,23116266	0	1
**EEF1A1**	0	0,23876298	0	1
**RPL10**	0	0,19363516	0	1
**RPL7A**	0	0,23577904	0	1
**RPLP0**	0	0,34119831	0	1
**RPL19**	0	0,19425998	0	1
**RPL18**	5,605E-292	0,22061248	1,508E-291	1
**RPL29**	2,585E-291	0,2064481	6,953E-292	1
**RPL18A**	7,228E-292	0,1981972	1,944E-288	1
**RPL32**	7,783E-288	0,1730187	2,093E-283	1
**RPL14**	1,367E-278	0,20732004	3,677E-274	1
**RPS12**	9,04E-266	0,17278293	2,432E-261	1
**RPS6**	1,782E-263	0,23729765	4,792E-259	1
**RPL13**	3,343E-259	0,17136618	8,99E-255	1
**RPS27A**	6,93E-257	0,17020009	1,864E-252	1
**RPS5**	2,295E-252	0,24127482	6,173E-248	1
**RPL5**	1,757E-241	0,23099005	4,726E-237	1
**RPS8**	9,73E-239	0,1983119	2,617E-234	1
**RPS13**	4,362E-229	0,1792085	1,173E-224	1
**RPL12**	6,69E-226	0,19468773	1,799E-221	1
**RPL8**	2,611E-225	0,20416696	7,022E-221	1
**RPS3**	7,212E-213	0,14976417	1,94E-208	1
**RPL28**	7,873E-211	0,14792294	2,118E-206	1
**RPS19**	8,201E-210	0,18675188	2,206E-205	1
**RPSA**	1,026E-208	0,27206436	2,76E-204	1
**RPS23**	2,847E-202	0,1525267	7,656E-198	1
**RPS14**	3,491E-191	0,15983683	9,391E-187	1
**RPL34**	6,449E-182	0,15815904	1,735E-177	1
**RPL3**	3,503E-180	0,15477378	9,421E-176	1
**RPL30**	4,202E-173	0,11992427	1,13E-168	1
**RPL10A**	9,978E-166	0,23163906	2,684E-161	1
**RPS2**	2,137E-163	0,20848522	5,749E-159	1
**RPS25**	6,785E-163	0,15575017	1,825E-158	1
**RPS4X**	4,075E-161	0,18253886	1,096E-156	1
**RPS15A**	5,089E-157	0,13321307	1,369E-152	1
**NACA**	1,48E-153	0,18708602	3,982E-149	1
**EEF1B2**	1,01E-144	0,20543006	2,716E-140	1

**RPS15**	7,224E-143	0,13207906	1,943E-138	1
**RPL22**	1,016E-140	0,24198751	2,732E-136	1
**RPLP1**	2,021E-140	0,17811909	5,436E-136	1
**RPL9**	4,476E-130	0,18176265	1,204E-125	1
**GNB2L1**	4,353E-121	0,16164752	1,171E-116	1
**RPS18**	3,8E-120	0,1433475	1,022E-115	1
**RPL24**	1,267E-118	0,1735318	3,409E-114	1
**RPL35**	5,269E-115	0,15452414	1,417E-110	1
**RPS7**	2,157E-114	0,13288128	5,801E-110	1
**RPL35A**	6,177E-110	0,13373945	1,661E-105	1
**TPT1**	1,442E-107	0,20936872	3,879E-103	1
**RPS9**	1,159E-99	0,14292713	3,1187E-95	1
**RPL39**	4,7841E-97	0,1227249	1,2868E-92	1
**FAU**	4,3413E-95	0,11986654	1,1677E-90	1
**RPS28**	3,1469E-94	0,11013031	8,4642E-90	1
**RPL15**	2,4655E-93	0,1763317	6,6315E-89	1
**RPL23A**	7,6288E-91	0,1819482	2,0519E-86	1
**RPL4**	1,5138E-73	0,18863609	4,0716E-69	1
**RPL36**	6,885E-71	0,10970925	1,8519E-66	1
**RPS24**	1,3874E-68	0,11644522	3,7316E-64	1
**TNFRSF4**	2,0445E-68	0,71214163	5,499E-64	1
**BTF3**	5,8512E-64	0,16005883	1,5738E-59	1
**LDHB**	2,3339E-60	0,18938053	6,2776E-56	1
**RPL7**	5,6746E-58	0,20318074	1,5263E-53	1
**FXYD5**	4,7099E-56	0,17811408	1,2668E-51	1
**RPS16**	4,2612E-55	0,16951659	1,1461E-50	1
**TIMP1**	5,21E-55	0,3982682	1,4013E-50	1
**RPS21**	7,1784E-55	0,10633216	1,9308E-50	1
**TNFRSF18**	1,2549E-51	0,84922458	3,3754E-47	1
**NPM1**	5,8083E-49	0,15828909	1,5622E-44	1
**RPL21**	2,2888E-47	0,13618123	6,1562E-43	1
**RPL27**	6,7555E-46	0,13467839	1,817E-41	1
**EEF1D**	1,7017E-45	0,12965694	4,5772E-41	1
**NPDC1**	2,8769E-40	0,65794085	7,7381E-36	1
**RPL26**	5,5439E-40	0,13520713	1,4911E-35	1
**COX4I1**	6,9849E-40	0,14397296	1,8787E-35	1
**LTB**	1,512E-35	0,14851128	4,0668E-31	1
**EIF3H**	2,6596E-33	0,19300829	7,1536E-29	1
**NME2**	1,238E-32	0,26688435	3,3298E-28	1
**UBA52**	4,2968E-30	0,10299333	1,1557E-25	1
**SLC25A6**	4,5234E-30	0,1744356	1,2167E-25	1

**EEF2**	1,0757E-29	0,11240694	2,8933E-25	1
**VIM**	3,4807E-28	0,13865379	9,3619E-24	1
**YBX1**	5,5209E-28	0,16686345	1,485E-23	1
**ATP5L**	6,0772E-28	0,1273966	1,6346E-23	1
**ACTG1**	1,3437E-27	0,14826434	3,6141E-23	1
**HNRNPA1**	2,9409E-27	0,11127981	7,9102E-23	1
**RPL17**	1,587E-26	0,17058313	4,2684E-22	1
**GLTSCR2**	5,9273E-26	0,17112851	1,5943E-21	1
**S100A4**	1,1685E-25	0,1158665	3,1429E-21	1
**PLP2**	1,1545E-24	0,2389188	3,1053E-20	1
**EIF3K**	2,0635E-23	0,12992035	5,5503E-19	1
**SSR2**	1,3538E-22	0,14969999	3,6412E-18	1
**EIF3L**	2,2024E-22	0,17154608	5,9239E-18	1
**GAPDH**	3,271E-20	0,11346762	8,798E-16	1
**PPA1**	9,4719E-19	0,24327744	2,5477E-14	1
**NOSIP**	6,3786E-18	0,1479544	1,7157E-13	1
**RPL31**	1,4476E-17	0,10131004	3,8937E-13	1
**RPL36A**	1,6472E-17	0,18473709	4,4306E-13	1
**LDHA**	1,4884E-16	0,20086455	4,0033E-12	1
**RPS11**	1,4499E-15	0,11452799	3,8997E-11	1
**CTSH**	2,5746E-15	0,32965198	6,9249E-11	1
**RP11-356J5.12**	2,2521E-14	0,66157604	6,0574E-10	1
**C12orf75**	3,8745E-14	0,24061969	1,0421E-09	1
**EIF3E**	6,3207E-13	0,12477375	1,7001E-08	1
**SERP1**	7,2821E-13	0,1338977	1,9587E-08	1
**UQCRB**	1,1761E-12	0,12366859	3,1633E-08	1
**SNRPD2**	3,6478E-12	0,10575189	9,8114E-08	1
**EIF3F**	5,1695E-12	0,11153724	1,3904E-07	1
**IFITM2**	5,1865E-12	0,12861034	1,395E-07	1
**PSME1**	9,9586E-12	0,11001378	2,6786E-07	1
**LGALS3**	3,1363E-11	0,24262811	8,4358E-07	1
**RP11-229P13.19**	1,6065E-10	0,74013957	4,3211E-06	1
**MZT2B**	2,6646E-10	0,103778	7,167E-06	1
**USP10**	4,4667E-10	0,16404236	1,2014E-05	1
**LGALS1**	5,0283E-10	0,21115249	1,3525E-05	1
**UXT**	6,2493E-10	0,11317676	1,6809E-05	1
**RHBDL1**	8,1417E-10	0,68406151	2,1899E-05	1
**PHB2**	9,9574E-10	0,16448704	2,6782E-05	1
**DDIT4**	1,2118E-09	0,16550674	3,2594E-05	1
**HSD11B1**	1,3143E-09	1,0713753	3,5352E-05	1
**ITM2C**	3,1111E-09	0,32589958	8,3678E-05	1

**SLC25A3**	5,0108E-09	0,12328443	0,00013478	1
**HIGD2A**	1,2465E-08	0,11035156	0,00033526	1
**IMPDH2**	1,3215E-08	0,24079653	0,00035543	1
**KIT**	1,579E-08	0,89624609	0,0004247	1
**CCDC85B**	2,4129E-08	0,14977591	0,000649	1
**C19orf53**	2,5612E-08	0,11953455	0,00068888	1
**PPP1R14B**	6,7564E-08	0,18590468	0,00181726	1
**OSTC**	7,8891E-08	0,17709075	0,00212194	1
**IL4I1**	1,4118E-07	0,41413118	0,00379721	1
**NHP2**	2,5778E-07	0,18475068	0,00693343	1
**PI16**	3,1606E-07	0,41703504	0,00850094	1
**UQCRH**	1,1021E-06	0,11524315	0,02964274	1
**LKAAEAR1**	1,3609E-06	1,2278893	0,03660481	1
**GZMK**	0	3,27972273	0	2
**GZMA**	0	3,1743971	0	2
**NKG7**	0	3,97528716	0	2
**CST7**	0	2,83434764	0	2
**PRF1**	0	3,08128902	0	2
**CCL5**	0	0,99460633	0	2
**CEBPD**	0	3,2187351	0	2
**EFHD2**	0	1,77507116	0	2
**PHACTR2**	0	1,70151732	0	2
**DUSP2**	0	1,71442945	0	2
**KLRG1**	0	1,30357595	0	2
**ALOX5AP**	0	1,20358413	0	2
**PLEK**	0	2,92452332	0	2
**SLC4A10**	0	2,68954703	0	2
**NCR3**	0	2,36937798	0	2
**EOMES**	0	4,31940193	0	2
**S1PR5**	0	5,36168254	0	2
**IL18RAP**	0	3,12945427	0	2
**GZMH**	0	5,47639956	0	2
**CXCR6**	0	3,61987334	0	2
**HCST**	0	0,90646248	0	2
**CD74**	0	0,97027815	0	2
**HLA-B**	0	0,45833133	0	2
**IL32**	3,548E-293	0,48468279	9,543E-292	2
**MYBL1**	5,248E-288	1,45179878	1,412E-283	2
**ARL4C**	1,313E-285	0,91120997	3,532E-281	2
**MAP3K8**	1,825E-266	2,32833297	4,909E-262	2
**B2M**	7,545E-266	0,19878402	2,029E-261	2

**RP11-222K16.2**	3,043E-258	2,66558247	8,185E-254	2
**SLAMF7**	2,556E-254	4,29172113	6,874E-250	2
**GZMM**	6,757E-251	1,00479074	1,817E-246	2
**CMC1**	5,456E-246	1,8647471	1,468E-241	2
**CD81**	5,769E-242	0,73393779	1,552E-237	2
**LYAR**	9,84E-228	0,80849663	2,647E-223	2
**GNLY**	4,013E-226	3,86144492	1,079E-221	2
**ADRB2**	5,657E-224	2,35059733	1,522E-219	2
**SAMD3**	1,509E-222	1,48581165	4,06E-218	2
**GPR65**	5,856E-218	1,28257399	1,575E-213	2
**CTSW**	1,201E-217	1,10887406	3,231E-213	2
**HLA-DRB1**	2,464E-215	2,78930134	6,628E-211	2
**S100A4**	2,237E-212	0,38417364	6,017E-208	2
**MATK**	4,412E-208	1,4669663	1,187E-203	2
**SRGN**	3,804E-205	0,7915411	1,023E-200	2
**HLA-A**	4,567E-199	0,33575294	1,228E-194	2
**PTGDR**	7,571E-199	2,23134475	2,036E-194	2
**IL18R1**	6,602E-193	1,96913613	1,776E-188	2
**GYG1**	8,542E-178	1,30142084	2,298E-173	2
**SYTL2**	2,174E-169	1,72601872	5,847E-165	2
**IGLV2-14**	2,454E-164	2,90812227	6,601E-160	2
**CLIC1**	8,256E-162	0,56968481	2,221E-157	2
**BHLHE40**	2,773E-157	1,11456369	7,459E-153	2
**CTSC**	1,709E-146	1,05643532	4,597E-142	2
**HOPX**	1,344E-136	0,88375844	3,614E-132	2
**TMEM171**	3,579E-134	4,77484991	9,627E-130	2
**TLE1**	1,052E-133	2,76787072	2,829E-129	2
**PARP8**	1,728E-130	0,7308952	4,648E-126	2
**HLA-DPA1**	1,132E-126	1,50326769	3,044E-122	2
**PFN1**	1,918E-123	0,28568484	5,16E-119	2
**GBP5**	5,43E-122	0,90080284	1,461E-117	2
**ITGB2**	9,73E-122	0,62433571	2,617E-117	2
**S100A9**	4,609E-120	2,73491132	1,24E-115	2
**YWHAQ**	3,922E-117	0,62870057	1,055E-112	2
**VAV3**	2,99E-116	2,02444213	8,042E-112	2
**PLCB1**	8,373E-115	1,11692009	2,252E-110	2
**JAKMIP1**	3,347E-113	2,22483487	9,003E-109	2
**SYNE2**	1,014E-112	0,66914479	2,728E-108	2
**TBX21**	1,209E-112	1,6125358	3,252E-108	2
**XBP1**	2,568E-111	0,61578128	6,907E-107	2
**TGFB1**	1,592E-109	0,43775909	4,282E-105	2

**LTK**	4,948E-109	1,67118383	1,331E-104	2
**CCL4**	5,305E-108	3,46379064	1,427E-103	2
**IGFBP4**	9,536E-108	2,48554226	2,565E-103	2
**FAM46C**	7,869E-107	1,0984785	2,116E-102	2
**AC092580.4**	2,073E-105	1,33176474	5,576E-101	2
**TGFBR3**	2,053E-100	1,53399245	5,5217E-96	2
**BTG1**	5,4835E-98	0,31730474	1,4749E-93	2
**UBXN10-AS1**	8,2849E-98	2,32001005	2,2284E-93	2
**ME1**	9,5022E-96	3,25408622	2,5558E-91	2
**IFNG-AS1**	9,1428E-90	1,09150196	2,4591E-85	2
**APMAP**	1,0424E-88	1,00363364	2,8037E-84	2
**APOBEC3G**	1,8679E-88	1,00105743	5,0241E-84	2
**TRGV4**	2,0934E-87	3,34410023	5,6307E-83	2
**CALM1**	2,7793E-87	0,24594591	7,4756E-83	2
**LCP1**	3,8029E-87	0,50266142	1,0229E-82	2
**DPP4**	1,6205E-84	0,97820832	4,3587E-80	2
**ID2**	1,332E-83	1,04024229	3,5826E-79	2
**ARHGAP26**	4,141E-83	1,42462603	1,1138E-78	2
**IFNGR1**	4,5883E-79	1,06117346	1,2341E-74	2
**IL23R**	2,1579E-78	2,68973923	5,8042E-74	2
**SPON2**	7,7341E-78	2,0045714	2,0802E-73	2
**CXCR4**	8,3435E-78	0,51520251	2,2442E-73	2
**CYBA**	4,0653E-77	0,30857583	1,0934E-72	2
**CNN2**	1,0375E-76	0,38549928	2,7905E-72	2
**KLRF1**	4,3252E-76	3,52119429	1,1633E-71	2
**CCR2**	5,0684E-74	1,61101279	1,3632E-69	2
**ASCL2**	5,25E-74	3,04820682	1,4121E-69	2
**TRBV6-4**	1,37E-73	3,17041403	3,6848E-69	2
**TRAV1-2**	2,0274E-73	2,2857065	5,4531E-69	2
**TRGV5**	3,1497E-73	1,92472755	8,4717E-69	2
**RORC**	4,4131E-73	1,21591592	1,187E-68	2
**MIR4435-2HG**	1,0152E-72	2,24036304	2,7307E-68	2
**CD99**	9,8549E-72	0,33143054	2,6507E-67	2
**COLQ**	2,0686E-71	1,54454585	5,5638E-67	2
**HLA-DRB5**	1,0083E-69	3,36493397	2,7121E-65	2
**MT-CO1**	5,7566E-69	0,24733878	1,5483E-64	2
**RHOU**	1,9632E-68	2,14680342	5,2805E-64	2
**CKLF**	2,0254E-68	0,93408394	5,4478E-64	2
**KDSR**	1,9985E-67	0,91873174	5,3753E-63	2
**PTPRC**	2,2451E-67	0,30936992	6,0385E-63	2
**RUNX3**	1,213E-66	0,63337416	3,2625E-62	2

**ESM1**	2,4444E-66	2,54919554	6,5748E-62	2
**CHST12**	3,3361E-66	0,96500197	8,9732E-62	2
**TMCC3**	6,9461E-65	2,79421996	1,8683E-60	2
**CX3CR1**	7,5984E-65	3,61132637	2,0437E-60	2
**MYO1F**	1,2543E-64	0,87524317	3,3737E-60	2
**FEZ1**	2,2999E-64	4,02068996	6,1859E-60	2
**IL12RB2**	2,3494E-63	1,4197888	6,3193E-59	2
**ZFP36L2**	2,3913E-63	0,3322968	6,4319E-59	2
**MYL6**	2,4748E-63	0,2620118	6,6566E-59	2
**SH3BGRL3**	5,0716E-63	0,20054236	1,3641E-58	2
**HLA-DPB1**	6,1633E-63	1,29149793	1,6577E-58	2
**CCR5**	7,3346E-63	3,02379186	1,9728E-58	2
**ACTB**	9,5E-63	0,25482504	2,5552E-58	2
**LGALS3**	2,3395E-62	0,59214038	6,2925E-58	2
**C12orf75**	1,0114E-60	0,6578411	2,7204E-56	2
**S100A6**	3,4469E-60	0,25636443	9,2711E-56	2
**HLA-DQB1**	1,6498E-59	1,95419659	4,4376E-55	2
**SLC15A4**	1,7648E-59	1,59629383	4,7469E-55	2
**SYNE1**	1,862E-58	1,03610733	5,0083E-54	2
**SYTL3**	2,0738E-58	0,79767247	5,5779E-54	2
**CBLB**	3,5741E-58	0,77031271	9,6133E-54	2
**ARPC5L**	6,1943E-58	0,67848224	1,6661E-53	2
**RAP1B**	1,253E-57	0,40906411	3,3702E-53	2
**ADGRG5**	2,0902E-57	2,32375368	5,622E-53	2
**PLA2G16**	6,9353E-57	1,42214053	1,8654E-52	2
**ACTN4**	2,3212E-56	0,86021019	6,2433E-52	2
**LINC00299**	2,5153E-56	2,08335385	6,7654E-52	2
**MT2A**	5,1197E-56	0,74326617	1,377E-51	2
**ZEB2**	1,7923E-55	2,80864698	4,8208E-51	2
**CD69**	2,4858E-54	0,41728731	6,686E-50	2
**DUSP1**	3,3058E-54	0,45652509	8,8917E-50	2
**PTPRCAP**	8,7257E-54	0,48895708	2,3469E-49	2
**LITAF**	1,4293E-53	0,51933187	3,8444E-49	2
**GNPDA1**	1,4644E-53	1,17905917	3,9388E-49	2
**SIPA1**	5,8018E-53	0,77958986	1,5605E-48	2
**DFNB31**	1,147E-51	1,97848074	3,0852E-47	2
**SH2D2A**	1,39E-51	0,72626792	3,7386E-47	2
**USP45**	1,7138E-51	1,38872936	4,6097E-47	2
**ATF7IP2**	2,6332E-51	0,642259	7,0826E-47	2
**SEPT7**	3,0591E-51	0,3745697	8,2281E-47	2
**FOSL2**	1,3193E-50	1,34008707	3,5484E-46	2

**FLNA**	1,3685E-50	0,49694093	3,6807E-46	2
**CHD9**	2,3893E-50	0,91453486	6,4265E-46	2
**IKZF2**	9,9374E-50	2,51992663	2,6729E-45	2
**SH2D1A**	1,2663E-49	0,90130644	3,4059E-45	2
**RHOC**	3,0535E-49	1,16300369	8,2129E-45	2
**ABHD17A**	4,4908E-49	0,49771491	1,2079E-44	2
**DNAJC1**	1,2705E-48	0,73427955	3,4173E-44	2
**LL22NC03-75H12.2**	2,614E-48	1,38032254	7,0308E-44	2
**KLRB1**	1,1019E-47	0,52007878	2,9637E-43	2
**AGTRAP**	2,6935E-47	0,94193073	7,2447E-43	2
**SSR4**	3,1347E-47	0,26496508	8,4313E-43	2
**PTPRM**	2,5587E-46	1,2035803	6,8822E-42	2
**CCDC107**	4,6584E-46	0,53396759	1,253E-41	2
**CLEC2B**	9,6467E-46	0,48624812	2,5947E-41	2
**TMSB4X**	1,0401E-45	0,12802224	2,7975E-41	2
**IL7R**	1,152E-45	0,20961782	3,0984E-41	2
**APOBEC3H**	1,0599E-44	1,66980327	2,8509E-40	2
**ITGAM**	2,3658E-44	2,33783226	6,3634E-40	2
**ABCB1**	7,0027E-44	1,00580222	1,8835E-39	2
**BCL7C**	8,2599E-43	0,50671516	2,2217E-38	2
**ABI3**	3,1682E-42	1,08380814	8,5215E-38	2
**C1orf21**	3,6773E-42	3,17041208	9,8908E-38	2
**PRR5L**	5,0754E-42	2,11256318	1,3651E-37	2
**LINC00152**	5,3282E-42	0,77344458	1,4331E-37	2
**PLXND1**	6,6401E-42	1,82136204	1,786E-37	2
**CD53**	8,5657E-42	0,3689572	2,3039E-37	2
**USP28**	9,5069E-42	1,61909234	2,5571E-37	2
**SLC9A3R1**	9,6848E-42	0,43077604	2,6049E-37	2
**EML4**	1,2875E-41	0,40213327	3,4631E-37	2
**ADAM12**	1,6145E-41	1,86336207	4,3425E-37	2
**NCAM1**	2,4323E-41	4,63225354	6,5422E-37	2
**E2F3**	4,9317E-41	1,52719744	1,3265E-36	2
**EVA1B**	6,4274E-40	1,19591328	1,7288E-35	2
**CFL1**	1,8054E-39	0,17245412	4,856E-35	2
**PTPN22**	2,3052E-39	0,91478626	6,2002E-35	2
**STK17A**	4,0868E-39	0,45340599	1,0992E-34	2
**PDIA3**	4,5671E-39	0,36284124	1,2284E-34	2
**ARAP2**	1,9393E-38	0,70919507	5,2161E-34	2
**PPP2R2B**	1,96E-38	0,98306167	5,2717E-34	2
**PZP**	6,0805E-38	1,27021229	1,6355E-33	2
**RARRES3**	9,0044E-38	0,28812486	2,4219E-33	2

**RAB18**	2,1466E-37	0,57671342	5,7738E-33	2
**LYN**	2,8845E-37	3,11909231	7,7584E-33	2
**ERN1**	4,6537E-37	0,48448492	1,2517E-32	2
**CD2**	5,3354E-36	0,37423739	1,4351E-31	2
**LAIR2**	8,0005E-36	1,61493337	2,1519E-31	2
**HDAC9**	1,684E-35	2,90176443	4,5295E-31	2
**MAPKAPK2**	1,9339E-35	0,69847056	5,2015E-31	2
**PSMB9**	2,5691E-35	0,32454817	6,9101E-31	2
**CASC8**	3,193E-35	2,78743281	8,5881E-31	2
**RPS26**	6,3564E-35	0,18989256	1,7097E-30	2
**PIP4K2A**	9,4793E-35	0,5243393	2,5497E-30	2
**MTRNR2L12**	1,6198E-34	0,21897176	4,3568E-30	2
**SPTSSB**	3,1989E-34	2,95018473	8,6041E-30	2
**ANXA2**	4,5641E-34	0,33223635	1,2276E-29	2
**RGS3**	1,0896E-33	1,41544163	2,9308E-29	2
**ITCH**	1,0908E-33	0,86122931	2,9338E-29	2
**UBL5**	3,5756E-33	0,25896392	9,6173E-29	2
**AGAP1**	3,6284E-33	1,44061441	9,7594E-29	2
**THEMIS**	3,7561E-33	0,57291619	1,0103E-28	2
**CPNE2**	6,3651E-33	1,98885808	1,712E-28	2
**REEP5**	1,0672E-32	0,46393674	2,8705E-28	2
**TMEM109**	1,0675E-32	0,54025345	2,8712E-28	2
**TRG-AS1**	1,1771E-32	0,62347342	3,166E-28	2
**RASAL3**	1,5988E-32	0,50949502	4,3003E-28	2
**RP11-81H14.2**	2,9682E-32	1,84252248	7,9835E-28	2
**C9orf142**	3,339E-32	0,27478976	8,981E-28	2
**AF131217.1**	5,4971E-32	1,7671015	1,4786E-27	2
**TRBV24-1**	5,6455E-32	1,57699752	1,5185E-27	2
**IL2RG**	8,8504E-32	0,20935351	2,3805E-27	2
**GTDC1**	9,0343E-32	1,18789883	2,43E-27	2
**AC131056.3**	9,2533E-32	2,32070093	2,4889E-27	2
**LBH**	2,4647E-31	0,43510331	6,6294E-27	2
**STAT4**	2,9153E-31	0,48910796	7,8413E-27	2
**ICAM3**	3,9672E-31	0,25354999	1,0671E-26	2
**HSP90B1**	5,1785E-31	0,32173801	1,3929E-26	2
**TRGV2**	6,1046E-31	1,55304473	1,642E-26	2
**CDC42EP3**	8,5071E-31	0,55109855	2,2881E-26	2
**NUCB2**	1,0182E-30	0,53991972	2,7386E-26	2
**IRF1**	1,0837E-30	0,42896376	2,9148E-26	2
**TPRG1**	1,4428E-30	1,50100863	3,8806E-26	2
**CAP1**	3,1942E-30	0,3403473	8,5914E-26	2

**ORAI1**	3,5636E-30	0,38433412	9,5849E-26	2
**PYHIN1**	3,5768E-30	0,71566851	9,6206E-26	2
**UTRN**	3,8578E-30	0,53948034	1,0376E-25	2
**CASP1**	5,1545E-30	0,54059122	1,3864E-25	2
**COL6A2**	7,4979E-30	1,27255402	2,0167E-25	2
**EMB**	1,1156E-29	0,40789375	3,0007E-25	2
**RP5-1028K7.2**	1,4973E-29	1,89229379	4,0274E-25	2
**AC133644.2**	1,6175E-29	1,23437406	4,3507E-25	2
**TSEN54**	2,1782E-29	0,5965273	5,8586E-25	2
**CYTH3**	2,2102E-29	1,28785916	5,9446E-25	2
**MSN**	3,2203E-29	0,35976353	8,6617E-25	2
**GAB3**	4,5246E-29	1,00768126	1,217E-24	2
**CXXC5**	6,5053E-29	2,44919321	1,7497E-24	2
**SASH3**	7,6163E-29	0,54375469	2,0486E-24	2
**RBL2**	1,3458E-28	0,37078277	3,6199E-24	2
**PLD1**	4,6707E-28	2,69513967	1,2563E-23	2
**RGS2**	5,0426E-28	1,08557381	1,3563E-23	2
**MICAL2**	6,7478E-28	1,30494995	1,815E-23	2
**TERF2IP**	1,3131E-27	0,39123695	3,5319E-23	2
**NFKBIA**	1,6781E-27	0,27840836	4,5137E-23	2
**RAB29**	1,7538E-27	0,69092515	4,7172E-23	2
**NEK7**	1,9737E-27	0,74942681	5,3086E-23	2
**MNDA**	3,5405E-27	4,23628342	9,5229E-23	2
**GTF3A**	4,0435E-27	0,26215071	1,0876E-22	2
**CD300A**	5,9198E-27	0,86743158	1,5922E-22	2
**LPCAT1**	6,14E-27	1,1011966	1,6515E-22	2
**CD40LG**	6,8719E-27	0,50696559	1,8483E-22	2
**RAB6B**	6,9955E-27	2,23002346	1,8816E-22	2
**TLN1**	2,1052E-26	0,43969877	5,6623E-22	2
**GAPDH**	3,1681E-26	0,13451827	8,5212E-22	2
**PTPRJ**	1,1571E-25	0,7902978	3,1122E-21	2
**ANXA4**	1,2451E-25	0,85869655	3,349E-21	2
**GAS7**	1,5106E-25	2,33874597	4,0631E-21	2
**CISD3**	1,8019E-25	0,61212042	4,8465E-21	2
**ARHGDIB**	1,8787E-25	0,11965911	5,0532E-21	2
**TNFAIP3**	2,1037E-25	0,36575696	5,6583E-21	2
**FGFBP2**	2,2823E-25	2,45326958	6,1387E-21	2
**PIK3R1**	2,61E-25	0,42672363	7,0201E-21	2
**IFITM2**	3,996E-25	0,19148493	1,0748E-20	2
**PPIB**	5,0041E-25	0,19093497	1,346E-20	2
**TP53I11**	5,2153E-25	2,07405446	1,4027E-20	2

**GALC**	5,6518E-25	0,93337842	1,5202E-20	2
**GTF3C1**	6,0864E-25	0,99361181	1,6371E-20	2
**TNFSF14**	6,1033E-25	1,30828004	1,6416E-20	2
**HSPA5**	6,1425E-25	0,36644954	1,6521E-20	2
**TRGC2**	1,5901E-24	1,10833514	4,2768E-20	2
**CFH**	1,8554E-24	0,89923889	4,9905E-20	2
**AOAH**	1,9307E-24	1,96776622	5,1931E-20	2
**F2R**	2,5151E-24	1,25086107	6,7648E-20	2
**JAK1**	4,7315E-24	0,25310985	1,2726E-19	2
**MKNK1**	5,4904E-24	0,82275707	1,4768E-19	2
**IL2RB**	6,7164E-24	0,77798525	1,8065E-19	2
**AC013461.1**	6,8126E-24	1,84046764	1,8324E-19	2
**MT-ND5**	6,888E-24	0,1771945	1,8527E-19	2
**PDE4B**	7,472E-24	0,56226802	2,0097E-19	2
**CFLAR**	7,5262E-24	0,42611202	2,0243E-19	2
**WIPF1**	9,7776E-24	0,35663974	2,6299E-19	2
**MT-CO2**	1,6885E-23	0,11601659	4,5416E-19	2
**VPS37B**	1,9581E-23	0,65261706	5,2667E-19	2
**AGPAT4**	2,1766E-23	0,87237173	5,8544E-19	2
**RAB27A**	2,2257E-23	0,67121125	5,9863E-19	2
**SPIDR**	3,3383E-23	0,71863238	8,979E-19	2
**HLA-DMB**	4,6127E-23	1,87076912	1,2407E-18	2
**GNG2**	4,8734E-23	0,42946413	1,3108E-18	2
**HLA-E**	4,9195E-23	0,12089864	1,3232E-18	2
**NTN4**	5,2077E-23	1,59214197	1,4007E-18	2
**CORO1A**	5,5542E-23	0,14943562	1,4939E-18	2
**MCTP2**	5,8638E-23	0,85968046	1,5772E-18	2
**TRGV10**	5,9222E-23	1,06617855	1,5929E-18	2
**CD244**	8,0705E-23	2,16153077	2,1707E-18	2
**MRPL10**	1,0018E-22	0,51275897	2,6947E-18	2
**FHL3**	1,5504E-22	1,46716627	4,17E-18	2
**BSG**	2,2616E-22	0,31068107	6,083E-18	2
**HLA-DMA**	3,0873E-22	1,44054718	8,3039E-18	2
**BRK1**	3,2286E-22	0,26190224	8,6841E-18	2
**PMAIP1**	6,7966E-22	0,54713099	1,8281E-17	2
**PTP4A2**	8,9083E-22	0,24360102	2,3961E-17	2
**SOX13**	1,0243E-21	1,72187796	2,7551E-17	2
**FAM49B**	1,0265E-21	0,417868	2,7609E-17	2
**ANXA1**	1,1261E-21	0,11430105	3,0289E-17	2
**PSMB8**	1,2014E-21	0,26400514	3,2313E-17	2
**SLC20A1**	1,3691E-21	0,7188909	3,6823E-17	2

**IQGAP1**	2,3567E-21	0,44689935	6,3387E-17	2
**MT-ND4L**	2,4972E-21	0,1115187	6,7167E-17	2
**PDE4D**	2,6948E-21	0,37892547	7,2482E-17	2
**TRGV7**	2,902E-21	1,68068675	7,8055E-17	2
**OAZ1**	3,1003E-21	0,16988235	8,339E-17	2
**TMEM65**	3,5173E-21	0,68923856	9,4604E-17	2
**RPS4Y1**	3,5631E-21	0,31595579	9,5836E-17	2
**PTPRE**	3,6719E-21	0,84054905	9,8763E-17	2
**PSME2**	3,9628E-21	0,24042704	1,0659E-16	2
**MAP1LC3B**	4,6394E-21	0,38304763	1,2478E-16	2
**DSEL**	5,3317E-21	1,34914056	1,4341E-16	2
**DUSP5**	6,0345E-21	0,63491345	1,6231E-16	2
**DIP2A**	6,1865E-21	0,6451076	1,664E-16	2
**TNFRSF1A**	6,5402E-21	0,73873966	1,7591E-16	2
**AKNA**	9,1667E-21	0,45306007	2,4656E-16	2
**AHNAK**	9,2375E-21	0,23840537	2,4846E-16	2
**RHBDF2**	1,1376E-20	0,9670755	3,0597E-16	2
**ADGRG1**	1,7773E-20	2,81857759	4,7804E-16	2
**GFPT2**	2,1163E-20	2,69779347	5,6922E-16	2
**SLC2A8**	2,3054E-20	2,30828363	6,2008E-16	2
**PDCD4**	2,4733E-20	0,32132741	6,6524E-16	2
**ARPC2**	3,0115E-20	0,16506257	8,1E-16	2
**LINC00869**	3,1782E-20	0,40298905	8,5484E-16	2
**SEC61B**	4,0419E-20	0,23331856	1,0871E-15	2
**ROR2**	7,0291E-20	2,68388502	1,8906E-15	2
**YPEL1**	7,7417E-20	1,5683714	2,0823E-15	2
**RRAS2**	9,1495E-20	0,77206251	2,4609E-15	2
**CCNH**	1,0072E-19	0,49956865	2,709E-15	2
**RAC2**	2,5807E-19	0,14525306	6,9414E-15	2
**RNF213**	2,642E-19	0,3222025	7,1062E-15	2
**PRNP**	2,7737E-19	0,42407971	7,4604E-15	2
**RP4-728D4.2**	3,2466E-19	0,88612038	8,7325E-15	2
**SLC4A4**	4,7612E-19	2,16920127	1,2806E-14	2
**SELT**	4,9571E-19	0,34566997	1,3333E-14	2
**CRTAM**	5,1046E-19	2,72125803	1,373E-14	2
**HSP90AA1**	5,5507E-19	0,16988337	1,493E-14	2
**MAN1A1**	7,5269E-19	0,79953861	2,0245E-14	2
**CLINT1**	8,097E-19	0,53882456	2,1778E-14	2
**ATPIF1**	1,0384E-18	0,24105683	2,7931E-14	2
**FYN**	1,0567E-18	0,26877465	2,8422E-14	2
**SMAD3**	1,0679E-18	0,51427912	2,8723E-14	2

**CTSD**	1,1961E-18	0,42524262	3,217E-14	2
**FYCO1**	1,2834E-18	0,92443573	3,452E-14	2
**HMGB1**	1,3908E-18	0,15948707	3,7409E-14	2
**ZFP36**	1,8669E-18	0,29419473	5,0213E-14	2
**PPP1R18**	2,0922E-18	0,34288778	5,6275E-14	2
**ITPRIP**	2,1409E-18	1,20384582	5,7584E-14	2
**GATA3**	2,2179E-18	0,37012607	5,9654E-14	2
**PTCH1**	2,2655E-18	1,31908973	6,0934E-14	2
**PPP2R5C**	2,5961E-18	0,33809267	6,9827E-14	2
**MAP4K1**	2,6768E-18	0,46915741	7,1997E-14	2
**CYP4F22**	3,3338E-18	2,16003951	8,9669E-14	2
**CALR**	3,7804E-18	0,29437718	1,0168E-13	2
**EIF4G3**	3,8082E-18	0,81755408	1,0243E-13	2
**UBC**	4,6924E-18	0,12068778	1,2621E-13	2
**TAP1**	5,2058E-18	0,39018753	1,4002E-13	2
**GUK1**	5,4351E-18	0,21013486	1,4619E-13	2
**PAM**	6,5934E-18	0,77582668	1,7734E-13	2
**GALNT3**	7,0684E-18	1,9592421	1,9012E-13	2
**TPST2**	8,0671E-18	0,5738009	2,1698E-13	2
**PILRB**	8,6677E-18	0,86421452	2,3313E-13	2
**ADAP1**	1,3903E-17	1,1339923	3,7395E-13	2
**HCLS1**	2,2088E-17	0,21233998	5,941E-13	2
**TSPAN15**	2,2413E-17	0,81132553	6,0284E-13	2
**CAMK2N1**	2,4439E-17	0,77326149	6,5735E-13	2
**A2M**	3,7966E-17	0,91380528	1,0212E-12	2
**YWHAZ**	3,8027E-17	0,17696693	1,0228E-12	2
**DBI**	4,5751E-17	0,27508463	1,2306E-12	2
**GPR35**	5,3301E-17	1,98851167	1,4336E-12	2
**KLHDC4**	9,442E-17	0,7333001	2,5396E-12	2
**B3GNT8**	9,443E-17	1,08605205	2,5399E-12	2
**FOXN3**	9,7319E-17	0,34409973	2,6176E-12	2
**WDR83OS**	1,1432E-16	0,24660337	3,075E-12	2
**SIT1**	1,1813E-16	0,37386691	3,1774E-12	2
**PLEKHG3**	1,191E-16	0,78740646	3,2034E-12	2
**GLCCI1**	1,2137E-16	0,49562957	3,2646E-12	2
**YARS**	1,2174E-16	0,59807967	3,2744E-12	2
**KLF3**	1,2318E-16	0,31370915	3,3132E-12	2
**TRIQK**	1,4563E-16	1,00512176	3,917E-12	2
**SRSF7**	1,813E-16	0,23731625	4,8764E-12	2
**C1orf141**	2,1209E-16	2,8077307	5,7046E-12	2
**TRGV9**	2,2308E-16	2,28028876	6,0001E-12	2

**PRSS23**	2,4948E-16	2,39847626	6,7103E-12	2
**DHRS7**	3,0247E-16	0,27367894	8,1355E-12	2
**B4GALT1**	3,1159E-16	0,59202467	8,381E-12	2
**SESN1**	3,2604E-16	0,4027145	8,7694E-12	2
**CYTIP**	3,5534E-16	0,25968926	9,5576E-12	2
**PITPNC1**	4,401E-16	0,33087395	1,1837E-11	2
**UBXN10**	6,0983E-16	2,61851445	1,6403E-11	2
**STX3**	6,1812E-16	1,18355366	1,6625E-11	2
**GOLIM4**	6,3408E-16	2,61801694	1,7055E-11	2
**GPR68**	7,5247E-16	1,20179269	2,0239E-11	2
**CCDC85B**	7,5997E-16	0,24326836	2,0441E-11	2
**KLF13**	7,9631E-16	0,32834253	2,1418E-11	2
**ELK3**	9,6008E-16	0,54527328	2,5823E-11	2
**IKZF3**	1,0639E-15	0,45944205	2,8615E-11	2
**LYSMD2**	1,4501E-15	0,415025	3,9004E-11	2
**P4HB**	1,4504E-15	0,26488828	3,9012E-11	2
**ZNF600**	1,5164E-15	0,78397904	4,0785E-11	2
**RASSF1**	1,7329E-15	0,47868751	4,6609E-11	2
**RUNX2**	1,7547E-15	0,50546285	4,7197E-11	2
**SEC61G**	1,7724E-15	0,21907294	4,7672E-11	2
**TRGV8**	1,8226E-15	2,36537774	4,9022E-11	2
**IL4I1**	2,0011E-15	0,8035511	5,3824E-11	2
**SLFN11**	2,01E-15	1,31459377	5,4064E-11	2
**P2RY8**	2,0653E-15	0,32676196	5,555E-11	2
**TRBV3-1**	2,2748E-15	0,94299328	6,1185E-11	2
**RP11-539L10.2**	2,8816E-15	0,6767218	7,7507E-11	2
**GPI**	3,085E-15	0,32672484	8,2978E-11	2
**FASLG**	3,4373E-15	2,14750959	9,2453E-11	2
**MVB12B**	4,0968E-15	1,00703896	1,1019E-10	2
**CEBPB**	4,508E-15	0,38210771	1,2125E-10	2
**DENND4C**	4,9747E-15	0,49543117	1,338E-10	2
**S100A11**	5,1674E-15	0,15586455	1,3899E-10	2
**FAM107B**	5,2596E-15	0,21228542	1,4147E-10	2
**SQSTM1**	5,3367E-15	0,2777574	1,4354E-10	2
**PBX4**	5,6206E-15	0,58151352	1,5118E-10	2
**SKAP1**	6,7215E-15	0,22536294	1,8079E-10	2
**ELL**	6,7404E-15	0,78176955	1,813E-10	2
**EAF1**	6,8398E-15	0,98586026	1,8397E-10	2
**PLEKHF1**	6,9268E-15	0,7774584	1,8631E-10	2
**RNF167**	7,1036E-15	0,33971778	1,9107E-10	2
**C9orf16**	7,7932E-15	0,1863131	2,0961E-10	2

**SERTAD3**	8,1776E-15	1,14642351	2,1995E-10	2
**AC006369.2**	8,8369E-15	0,39822085	2,3768E-10	2
**SPOCK2**	9,0971E-15	0,22616096	2,4469E-10	2
**TYROBP**	1,132E-14	2,2218073	3,0447E-10	2
**C1orf61**	1,5077E-14	1,25807703	4,0554E-10	2
**PSMB3**	1,5386E-14	0,29512773	4,1383E-10	2
**CREB3L2**	2,0152E-14	0,69803575	5,4203E-10	2
**WNT1**	2,2712E-14	1,03620702	6,1088E-10	2
**CAST**	2,3558E-14	0,23667266	6,3363E-10	2
**FBXO6**	2,3611E-14	0,90341567	6,3508E-10	2
**AUTS2**	2,6314E-14	0,51106791	7,0777E-10	2
**PTK2B**	2,6326E-14	0,55483565	7,0809E-10	2
**PXN**	2,7153E-14	0,4648662	7,3032E-10	2
**OSTF1**	2,9452E-14	0,27869648	7,9217E-10	2
**RPL22L1**	3,0402E-14	0,27619837	8,1771E-10	2
**TESC**	3,0761E-14	1,07019619	8,2738E-10	2
**LYST**	3,1139E-14	0,63346614	8,3755E-10	2
**SIPA1L2**	3,3921E-14	1,71795144	9,1239E-10	2
**ZDHHC14**	3,4262E-14	1,02457286	9,2154E-10	2
**SKAP2**	3,8138E-14	0,90575117	1,0258E-09	2
**ARF1**	3,8634E-14	0,18148298	1,0391E-09	2
**DIAPH1**	4,2039E-14	0,48807172	1,1307E-09	2
**TPBG**	4,5989E-14	1,03602427	1,237E-09	2
**TRBV10-2**	4,9432E-14	1,22480492	1,3296E-09	2
**PDIA6**	5,1335E-14	0,32115676	1,3807E-09	2
**LINC01146**	5,6995E-14	1,48654134	1,533E-09	2
**TMEM258**	6,9627E-14	0,20640036	1,8728E-09	2
**DDOST**	8,6695E-14	0,22839055	2,3318E-09	2
**PARVG**	9,0735E-14	0,49848838	2,4405E-09	2
**RNF7**	9,7885E-14	0,28380138	2,6328E-09	2
**CLU**	1,2102E-13	1,33073481	3,255E-09	2
**ZNF92**	1,2544E-13	0,58252164	3,3739E-09	2
**SPN**	1,3247E-13	0,39125302	3,563E-09	2
**DERL1**	1,3469E-13	0,52567964	3,6228E-09	2
**PTPN4**	1,3861E-13	0,42749196	3,7281E-09	2
**ZBTB16**	1,4359E-13	1,18293636	3,8621E-09	2
**PACSIN1**	1,858E-13	2,23974879	4,9975E-09	2
**GK5**	1,9193E-13	0,86970851	5,1624E-09	2
**NDUFA3**	2,0678E-13	0,24669751	5,5617E-09	2
**SYNRG**	2,2511E-13	0,3964009	6,0547E-09	2
**DGKZ**	2,3267E-13	0,33095272	6,2581E-09	2

**FKBP11**	2,5256E-13	0,25056108	6,7931E-09	2
**RHOF**	3,4416E-13	0,30233366	9,2568E-09	2
**UBQLN4**	4,09E-13	0,76200306	1,1001E-08	2
**FAM117A**	4,3765E-13	0,29589181	1,1771E-08	2
**CYB5A**	4,6089E-13	0,49398355	1,2396E-08	2
**ADAM8**	4,9435E-13	0,40893417	1,3297E-08	2
**UAP1**	5,1449E-13	0,77132569	1,3838E-08	2
**WDR1**	5,2631E-13	0,25073174	1,4156E-08	2
**LLGL2**	5,9744E-13	0,7198435	1,6069E-08	2
**CERK**	6,0513E-13	0,42132771	1,6276E-08	2
**SPCS3**	6,1952E-13	0,39496882	1,6663E-08	2
**TRGV3**	6,201E-13	0,99134648	1,6679E-08	2
**ITGAL**	6,3325E-13	0,43927273	1,7033E-08	2
**SEC24C**	6,6652E-13	0,60796294	1,7927E-08	2
**NPC1**	6,941E-13	0,76309128	1,8669E-08	2
**TNIP3**	7,5764E-13	1,78079261	2,0378E-08	2
**FURIN**	1,0314E-12	0,74971354	2,7741E-08	2
**PSME1**	1,1091E-12	0,13234165	2,983E-08	2
**LCK**	1,1226E-12	0,16106407	3,0193E-08	2
**CMIP**	1,1566E-12	0,82708593	3,1109E-08	2
**BATF**	1,2706E-12	0,39097939	3,4175E-08	2
**DYNLT1**	1,3756E-12	0,27015003	3,7001E-08	2
**HERPUD1**	1,6729E-12	0,34692535	4,4997E-08	2
**CDKN2A**	1,9377E-12	0,93853979	5,2119E-08	2
**KMT2E**	2,1106E-12	0,23343927	5,677E-08	2
**TBKBP1**	2,218E-12	1,15477953	5,9658E-08	2
**SLC35D1**	2,3181E-12	0,94090299	6,235E-08	2
**B4GALT5**	2,7287E-12	0,87199878	7,3394E-08	2
**CPNE7**	2,844E-12	1,52946909	7,6495E-08	2
**PNP**	2,8696E-12	0,47871063	7,7182E-08	2
**LPXN**	3,6262E-12	0,32706075	9,7535E-08	2
**ACBD5**	3,6956E-12	0,82780348	9,9402E-08	2
**GALNT1**	3,9136E-12	0,67481632	1,0526E-07	2
**ARF6**	3,9823E-12	0,21394161	1,0711E-07	2
**HSH2D**	4,4696E-12	0,61575186	1,2022E-07	2
**FAM96B**	4,5416E-12	0,29488628	1,2216E-07	2
**PLIN2**	4,8991E-12	0,5082259	1,3177E-07	2
**MBD2**	4,9511E-12	0,31772297	1,3317E-07	2
**NCL**	5,1978E-12	0,17210033	1,3981E-07	2
**GFI1**	5,5316E-12	0,8664799	1,4878E-07	2
**ACAA2**	6,1778E-12	0,61729189	1,6617E-07	2

**TGFBR1**	6,845E-12	0,78714398	1,8411E-07	2
**PTPN12**	6,919E-12	0,897461	1,861E-07	2
**MAD1L1**	6,985E-12	0,39320184	1,8788E-07	2
**LCP2**	7,2531E-12	0,22207769	1,9509E-07	2
**HM13**	8,1028E-12	0,31664618	2,1794E-07	2
**RAB27B**	8,4229E-12	1,09849843	2,2655E-07	2
**CDKN2C**	1,0331E-11	0,93984012	2,7788E-07	2
**GSR**	1,0972E-11	0,63411247	2,9512E-07	2
**IL16**	1,2404E-11	0,2468591	3,3362E-07	2
**SELK**	1,259E-11	0,29144313	3,3864E-07	2
**OSTC**	1,3404E-11	0,26358877	3,6052E-07	2
**MAPK1**	1,4507E-11	0,49526193	3,902E-07	2
**MTFP1**	1,5738E-11	0,49025032	4,2331E-07	2
**IRF5**	1,9467E-11	0,90697894	5,236E-07	2
**NFKBIB**	2,1428E-11	0,59404767	5,7635E-07	2
**H3F3A**	2,4881E-11	0,10530866	6,6924E-07	2
**TRAV8-4**	2,6635E-11	0,71420411	7,164E-07	2
**GSAP**	2,8024E-11	0,92560259	7,5377E-07	2
**CCDC88C**	2,811E-11	0,32321841	7,5606E-07	2
**BUB3**	2,8649E-11	0,22478381	7,7058E-07	2
**PGAM1**	2,9092E-11	0,19327851	7,8248E-07	2
**TNFSF12**	3,0057E-11	0,58159916	8,0844E-07	2
**ZBTB44**	3,0952E-11	0,52967842	8,3251E-07	2
**RGS9**	3,2247E-11	1,75572073	8,6734E-07	2
**STK39**	3,3534E-11	0,5643125	9,0195E-07	2
**RNF125**	3,4724E-11	0,34995408	9,3397E-07	2
**MIS18BP1**	3,6275E-11	0,48712643	9,7568E-07	2
**SNX10**	3,9049E-11	0,64745918	1,0503E-06	2
**KIF5C**	4,1334E-11	0,56450585	1,1118E-06	2
**SIRT2**	4,5637E-11	0,49426922	1,2275E-06	2
**TRGC1**	5,4098E-11	1,46072549	1,4551E-06	2
**HLA-F**	5,5998E-11	0,20902249	1,5062E-06	2
**AGPAT2**	5,9389E-11	0,71306955	1,5974E-06	2
**STX11**	6,1756E-11	0,86635349	1,6611E-06	2
**OST4**	6,1889E-11	0,11458525	1,6646E-06	2
**SURF4**	6,4912E-11	0,41369304	1,7459E-06	2
**DDAH2**	6,5598E-11	0,76860582	1,7644E-06	2
**FAM19A1**	6,6644E-11	0,64185895	1,7925E-06	2
**APOBEC3C**	6,7562E-11	0,56091904	1,8172E-06	2
**CASP4**	7,0595E-11	0,35152622	1,8988E-06	2
**MRPL14**	7,1979E-11	0,33959801	1,936E-06	2

**TCEB2**	7,2461E-11	0,14345061	1,949E-06	2
**GEMIN7**	7,3306E-11	0,59001069	1,9717E-06	2
**AKR1C3**	7,5834E-11	1,3615264	2,0397E-06	2
**DENND2D**	7,8412E-11	0,33151733	2,109E-06	2
**TMEM50A**	8,1508E-11	0,20094155	2,1923E-06	2
**CALHM2**	8,6513E-11	0,54091859	2,3269E-06	2
**C19orf43**	8,7089E-11	0,15044093	2,3424E-06	2
**C20orf24**	9,1226E-11	0,34379395	2,4537E-06	2
**LAPTM5**	9,6211E-11	0,1080405	2,5878E-06	2
**ITPRIPL1**	9,6216E-11	1,08728512	2,5879E-06	2
**PPP2R5A**	1,0525E-10	0,44851054	2,8308E-06	2
**TIPARP**	1,091E-10	0,45449343	2,9344E-06	2
**RORA**	1,1807E-10	0,1919611	3,1758E-06	2
**GSDMA**	1,2569E-10	1,46838814	3,3808E-06	2
**UQCR11**	1,2909E-10	0,14505453	3,4722E-06	2
**TSTD3**	1,4038E-10	0,98481461	3,7759E-06	2
**PDZD8**	1,4166E-10	0,54529965	3,8102E-06	2
**FCRL6**	1,4424E-10	1,42194892	3,8796E-06	2
**CTSH**	1,4475E-10	0,28641861	3,8934E-06	2
**H2AFV**	1,5131E-10	0,2414924	4,0698E-06	2
**TMED9**	1,5659E-10	0,24612335	4,2118E-06	2
**YES1**	1,6202E-10	0,82527082	4,3578E-06	2
**ATP2B4**	1,9855E-10	0,50247028	5,3404E-06	2
**PBXIP1**	2,0221E-10	0,22274406	5,4387E-06	2
**EHD1**	2,0609E-10	0,35436119	5,5432E-06	2
**ARHGAP25**	2,2305E-10	0,42294014	5,9994E-06	2
**TMEM117**	2,3452E-10	0,77231603	6,3079E-06	2
**CMTM3**	2,3701E-10	0,3195492	6,3748E-06	2
**CIDEB**	2,4512E-10	0,53999377	6,5929E-06	2
**TAPBP**	2,5708E-10	0,24924405	6,9147E-06	2
**MXRA7**	2,6143E-10	0,60001904	7,0317E-06	2
**ARPC3**	2,7404E-10	0,1187841	7,3708E-06	2
**DLEU2**	2,9645E-10	0,51105393	7,9736E-06	2
**DLEU1**	3,0752E-10	0,57164348	8,2714E-06	2
**EMC7**	3,2472E-10	0,3437325	8,7341E-06	2
**RAB9A**	3,4044E-10	0,4133515	9,1569E-06	2
**ABCA2**	3,4244E-10	0,73959247	9,2105E-06	2
**AGO4**	3,4666E-10	0,61243291	9,324E-06	2
**MAP2K3**	3,8955E-10	0,47220104	1,0478E-05	2
**RUNX1**	3,8991E-10	0,43444421	1,0487E-05	2
**ADSS**	3,8994E-10	0,37953654	1,0488E-05	2

**MMP25-AS1**	3,9114E-10	0,65215005	1,052E-05	2
**TGFBI**	4,0015E-10	1,70388723	1,0763E-05	2
**HLTF**	4,0513E-10	0,73174766	1,0897E-05	2
**MSC-AS1**	4,3556E-10	1,64415407	1,1715E-05	2
**STOM**	4,921E-10	0,32739825	1,3236E-05	2
**LUZP1**	5,0019E-10	0,58575614	1,3453E-05	2
**ELOVL5**	5,0344E-10	0,32938816	1,3541E-05	2
**COX6A1**	5,3638E-10	0,12835794	1,4427E-05	2
**LMO4**	5,6212E-10	0,55994391	1,5119E-05	2
**NR4A2**	5,8546E-10	0,57370425	1,5747E-05	2
**GALNT10**	5,941E-10	0,53463044	1,5979E-05	2
**ZC3H12A**	6,2706E-10	0,51390108	1,6866E-05	2
**EVI2A**	6,3303E-10	0,52927544	1,7027E-05	2
**RP11-18H21.1**	6,339E-10	0,37567566	1,705E-05	2
**RP11-118B22.4**	6,5587E-10	1,00851248	1,7641E-05	2
**ELOVL6**	7,2036E-10	1,1353562	1,9375E-05	2
**SYNM**	7,4054E-10	1,03494263	1,9918E-05	2
**BCL2L1**	7,5771E-10	0,60297535	2,038E-05	2
**RER1**	7,6444E-10	0,279373	2,0561E-05	2
**BCL6**	7,6483E-10	0,76120985	2,0572E-05	2
**GPR108**	7,6515E-10	0,3675366	2,058E-05	2
**MXD4**	7,6716E-10	0,40190599	2,0634E-05	2
**SS18L2**	7,964E-10	0,26929036	2,1421E-05	2
**MAF**	8,0852E-10	0,54597712	2,1747E-05	2
**SRXN1**	8,9637E-10	0,85663832	2,411E-05	2
**PPP1CA**	9,3488E-10	0,14155554	2,5146E-05	2
**TOMM5**	9,568E-10	0,25512144	2,5735E-05	2
**COPE**	1,0231E-09	0,20972123	2,7519E-05	2
**PLEKHA5**	1,0569E-09	0,68218663	2,8427E-05	2
**WDSUB1**	1,1593E-09	0,79404259	3,1181E-05	2
**CD70**	1,1989E-09	0,88896909	3,2246E-05	2
**PSTPIP1**	1,255E-09	0,41008259	3,3756E-05	2
**SRSF3**	1,2839E-09	0,18814774	3,4534E-05	2
**MMP23B**	1,5297E-09	1,15552054	4,1145E-05	2
**CTBP1**	1,6216E-09	0,30378652	4,3616E-05	2
**ARPC1B**	1,6611E-09	0,16720507	4,4678E-05	2
**HNRNPA2B1**	1,6996E-09	0,11730038	4,5713E-05	2
**SRPRA**	1,7432E-09	0,32925104	4,6886E-05	2
**PHLDA1**	1,7497E-09	1,09996253	4,7061E-05	2
**LPP**	1,7905E-09	0,40799606	4,816E-05	2
**RASSF2**	1,875E-09	0,61771766	5,0431E-05	2

**IL17RE**	1,9971E-09	1,1050075	5,3716E-05	2
**VASP**	2,0743E-09	0,35105982	5,5793E-05	2
**SHISA5**	2,1149E-09	0,2231801	5,6884E-05	2
**CHN2**	2,1262E-09	0,97032766	5,7187E-05	2
**IGF2R**	2,4055E-09	0,42840532	6,47E-05	2
**GOLGA4**	2,6817E-09	0,34642633	7,2128E-05	2
**ST8SIA4**	2,6974E-09	0,60868329	7,2552E-05	2
**CAPN12**	2,8573E-09	0,73943529	7,6852E-05	2
**SRM**	2,9587E-09	0,28341196	7,9581E-05	2
**SACM1L**	3,0319E-09	0,47892999	8,1548E-05	2
**METRNL**	3,1696E-09	0,89931153	8,5254E-05	2
**RNF115**	3,4723E-09	0,47833024	9,3394E-05	2
**PLPP2**	3,5069E-09	1,87465656	9,4326E-05	2
**DENND3**	3,5241E-09	1,00992142	9,4789E-05	2
**NMU**	3,5976E-09	1,59525197	9,6766E-05	2
**CACNA2D4**	3,6365E-09	0,6138492	9,7811E-05	2
**YWHAB**	4,0306E-09	0,12677661	0,00010841	2
**CTNNBIP1**	4,5677E-09	0,73393886	0,00012286	2
**GNA15**	4,5831E-09	0,86755397	0,00012327	2
**HSP90AB1**	4,7838E-09	0,12604883	0,00012867	2
**KRTCAP2**	4,967E-09	0,14763438	0,0001336	2
**TMEM59**	5,5186E-09	0,17769642	0,00014843	2
**ACTG1**	5,6225E-09	0,11122413	0,00015123	2
**ARFGAP3**	5,73E-09	0,45947062	0,00015412	2
**FAM173A**	5,7749E-09	0,40842065	0,00015533	2
**CCNDBP1**	5,7764E-09	0,28349794	0,00015537	2
**XRCC5**	6,4869E-09	0,1941075	0,00017448	2
**METTL9**	6,6328E-09	0,23892495	0,0001784	2
**ADAM10**	7,0003E-09	0,37922012	0,00018829	2
**BCL2L11**	7,1992E-09	0,72145454	0,00019364	2
**MVD**	7,2969E-09	0,52378726	0,00019627	2
**KDELR2**	7,8289E-09	0,2746534	0,00021057	2
**KIF21A**	8,2222E-09	0,65548886	0,00022115	2
**TBK1**	8,3581E-09	0,54354729	0,00022481	2
**EIF6**	8,375E-09	0,3395584	0,00022526	2
**DAPK2**	8,4184E-09	1,1009573	0,00022643	2
**ERMP1**	8,5352E-09	0,80677511	0,00022957	2
**MBOAT1**	8,6064E-09	0,82860061	0,00023149	2
**AKIRIN2**	8,9003E-09	0,39053026	0,00023939	2
**RNF149**	8,9889E-09	0,28861773	0,00024177	2
**UBE2E3**	9,5614E-09	0,3319809	0,00025717	2

**SYTL1**	9,648E-09	0,19251783	0,0002595	2
**TUBA4A**	1,004E-08	0,26269193	0,00027004	2
**UBB**	1,0442E-08	0,11815607	0,00028086	2
**SNTA1**	1,0732E-08	0,58749643	0,00028865	2
**FOXP4**	1,1718E-08	0,8313184	0,00031519	2
**SIPA1L1**	1,2002E-08	0,50183728	0,00032282	2
**LAIR1**	1,2197E-08	0,47744158	0,00032805	2
**PRELID1**	1,2279E-08	0,19090726	0,00033026	2
**RP11-325F22.2**	1,2564E-08	0,72696708	0,00033792	2
**MIER1**	1,2584E-08	0,25502512	0,00033848	2
**FLT4**	1,2908E-08	1,31253582	0,0003472	2
**ATP6V0C**	1,3344E-08	0,17905917	0,00035892	2
**SSBP4**	1,3462E-08	0,1933014	0,0003621	2
**CCDC159**	1,348E-08	0,69953871	0,00036258	2
**LBR**	1,6659E-08	0,32857328	0,00044809	2
**C10orf54**	1,6736E-08	0,17605235	0,00045016	2
**TCOF1**	1,76E-08	0,63167812	0,00047338	2
**TAX1BP1**	1,7634E-08	0,20804044	0,0004743	2
**MT1E**	1,7795E-08	0,79000062	0,00047864	2
**ANXA6**	1,8304E-08	0,17959372	0,00049232	2
**PPP1R14B**	1,843E-08	0,25805391	0,00049571	2
**NDUFB7**	1,9179E-08	0,20901676	0,00051585	2
**NDUFB2**	1,9183E-08	0,16005483	0,00051595	2
**BTBD11**	1,9462E-08	0,50652129	0,00052346	2
**SIK3**	2,0029E-08	0,35470242	0,00053872	2
**PSMA5**	2,0574E-08	0,24423428	0,00055338	2
**BIN2**	2,0586E-08	0,15717938	0,00055371	2
**RPA2**	2,1157E-08	0,27686912	0,00056907	2
**TOR2A**	2,3641E-08	0,56382429	0,00063586	2
**AFF3**	2,418E-08	1,13841464	0,00065036	2
**RP11-169D4.2**	2,5174E-08	0,73908137	0,00067711	2
**NFIL3**	2,5721E-08	0,89053314	0,00069182	2
**RASGEF1A**	2,5949E-08	0,90951854	0,00069794	2
**ADA**	2,8631E-08	0,41127116	0,00077009	2
**FAM129A**	2,9021E-08	0,48277007	0,00078057	2
**ZBP1**	2,9226E-08	0,42077731	0,0007861	2
**EDEM2**	3,0372E-08	0,44997623	0,00081691	2
**MT1X**	3,0438E-08	0,33963385	0,0008187	2
**CABIN1**	3,1568E-08	0,35610945	0,00084907	2
**GLS**	3,1768E-08	0,28447201	0,00085446	2
**HOTAIRM1**	3,4479E-08	0,56540438	0,00092737	2

**FMNL1**	3,5866E-08	0,25197681	0,00096468	2
**UQCR10**	3,7376E-08	0,1443845	0,0010053	2
**DOK2**	4,0045E-08	0,27753728	0,00107708	2
**GRAP2**	4,0261E-08	0,37804989	0,00108289	2
**SCD5**	4,3858E-08	1,25936883	0,00117964	2
**IDS**	4,4696E-08	0,26233899	0,00120218	2
**ETHE1**	4,5659E-08	0,33932419	0,00122808	2
**FRMD4B**	4,6001E-08	0,68925712	0,0012373	2
**JAKMIP2**	4,6157E-08	1,0476185	0,00124148	2
**RP11-473M20.9**	4,7699E-08	0,89871302	0,00128297	2
**SCAMP2**	5,039E-08	0,30287428	0,00135533	2
**LMAN2**	5,15E-08	0,22929056	0,00138519	2
**PSMB6**	5,7116E-08	0,20039339	0,00153624	2
**DNAJB6**	6,0597E-08	0,30862957	0,00162989	2
**ENPP5**	6,3375E-08	1,10182138	0,00170459	2
**PEX2**	6,4917E-08	0,47801735	0,00174607	2
**MPST**	6,6344E-08	0,5751404	0,00178445	2
**PDE4A**	6,6949E-08	0,91970986	0,00180072	2
**FCHO2**	7,1589E-08	0,94433266	0,00192553	2
**TTC38**	7,7404E-08	1,04038261	0,00208193	2
**HEG1**	8,2836E-08	0,78972949	0,00222804	2
**SAT1**	8,2963E-08	0,26180146	0,00223146	2
**TC2N**	8,4935E-08	0,19707296	0,00228451	2
**EDF1**	8,5758E-08	0,1057788	0,00230662	2
**SNHG22**	9,024E-08	0,9013133	0,00242718	2
**SELPLG**	9,1238E-08	0,15681023	0,00245404	2
**CLSTN3**	9,4502E-08	0,60175092	0,00254181	2
**PDLIM2**	9,6242E-08	0,29122494	0,00258862	2
**SPTY2D1**	9,6898E-08	0,52311362	0,00260627	2
**PVRIG**	9,875E-08	0,93476608	0,00265607	2
**TSC22D4**	9,8908E-08	0,22172633	0,00266034	2
**TAF10**	1,0344E-07	0,15241198	0,00278233	2
**ICAM1**	1,0398E-07	1,10751893	0,0027968	2
**SNRPD3**	1,0652E-07	0,24194917	0,00286519	2
**TBC1D31**	1,0889E-07	0,74963059	0,00292884	2
**TNFSF10**	1,1202E-07	0,35284932	0,00301312	2
**WWOX**	1,1287E-07	0,47894308	0,00303592	2
**DNAJC3**	1,1414E-07	0,40114244	0,00307009	2
**LTB4R2**	1,2241E-07	0,65133158	0,00329258	2
**KEAP1**	1,2321E-07	0,55112246	0,00331397	2
**TMED10**	1,2844E-07	0,2150786	0,00345463	2

**RNF19A**	1,3126E-07	0,30796718	0,00353042	2
**MANF**	1,3557E-07	0,37042468	0,00364654	2
**TTC22**	1,4448E-07	0,9438727	0,00388621	2
**TRAV21**	1,4574E-07	0,48718343	0,00392003	2
**SSSCA1**	1,4769E-07	0,41961937	0,00397241	2
**PSMB10**	1,4939E-07	0,18604396	0,00401816	2
**PIK3CG**	1,5012E-07	0,71303766	0,00403778	2
**FAM111A**	1,6112E-07	0,43144028	0,00433367	2
**GABARAPL2**	1,6864E-07	0,17262327	0,00453604	2
**MYH9**	1,7228E-07	0,13406555	0,00463376	2
**SPATS2L**	1,7276E-07	0,67866504	0,00464678	2
**XCL1**	1,7602E-07	1,23960928	0,00473453	2
**COMMD7**	1,8566E-07	0,31752897	0,00499369	2
**HPS3**	1,9367E-07	0,56068868	0,00520904	2
**PPP5C**	1,9634E-07	0,4528488	0,00528104	2
**SEC61A1**	2,0266E-07	0,36596496	0,00545096	2
**SRSF9**	2,0573E-07	0,15147651	0,00553347	2
**PREP**	2,0885E-07	0,45356233	0,00561744	2
**LATS2**	2,1504E-07	1,11441019	0,00578388	2
**PTRH1**	2,2911E-07	0,59298774	0,00616227	2
**ATG2A**	2,2965E-07	0,57358203	0,00617702	2
**SESN2**	2,3286E-07	0,79352722	0,00626313	2
**TRIM22**	2,3674E-07	0,22410045	0,00636763	2
**PDE8A**	2,3877E-07	0,57338583	0,00642208	2
**SLC25A20**	2,3891E-07	0,69041968	0,00642594	2
**UCK2**	2,5137E-07	1,04677061	0,00676102	2
**RP3-395M20.8**	2,5227E-07	0,8524941	0,0067854	2
**TAPBPL**	2,5498E-07	0,39010687	0,0068583	2
**TPM3**	2,5618E-07	0,11498844	0,00689034	2
**CYCS**	2,6196E-07	0,24907501	0,00704588	2
**TES**	2,8923E-07	0,28763093	0,00777942	2
**SLC1A5**	2,92E-07	0,72877607	0,00785404	2
**HSPA1A**	3,1282E-07	0,44604598	0,00841396	2
**TACC1**	3,2316E-07	0,43797316	0,00869197	2
**PRR13**	3,4427E-07	0,11731778	0,00925976	2
**LAG3**	3,4491E-07	0,48987474	0,0092771	2
**AC015849.2**	3,4828E-07	0,72821905	0,00936764	2
**SET**	3,6038E-07	0,13519698	0,00969323	2
**EIF4A1**	3,6288E-07	0,3600065	0,00976029	2
**RARG**	3,7461E-07	0,46663471	0,01007585	2
**CARD11**	3,7495E-07	0,35638969	0,01008496	2

**VPS36**	3,799E-07	0,35290586	0,0102182	2
**FUT11**	3,8078E-07	0,75898303	0,01024188	2
**MESDC2**	3,9198E-07	0,33180302	0,01054318	2
**TMEM64**	3,9802E-07	0,72060853	0,01070554	2
**HNRNPLL**	4,0592E-07	0,34255773	0,01091804	2
**RNPEPL1**	4,0781E-07	0,24007843	0,0109688	2
**ANKRD39**	4,1367E-07	0,49584271	0,01112659	2
**ABHD17C**	4,1905E-07	1,09588378	0,01127115	2
**GLUL**	4,2493E-07	0,42588869	0,0114294	2
**RSU1**	4,2606E-07	0,3375063	0,01145964	2
**SNAP29**	4,4449E-07	0,47986362	0,01195532	2
**PTPN7**	4,5365E-07	0,37960933	0,01220183	2
**IQSEC1**	4,5405E-07	0,52401286	0,01221261	2
**TTC16**	4,8392E-07	0,85916086	0,01301608	2
**INPP5D**	4,8881E-07	0,3632525	0,01314739	2
**PI4K2A**	5,0098E-07	0,84945122	0,01347476	2
**SDF2L1**	5,3025E-07	0,30992611	0,01426207	2
**USP34**	5,6886E-07	0,32566412	0,01530068	2
**CAMK2D**	5,7388E-07	0,40632282	0,01543556	2
**SLC27A3**	6,0011E-07	0,69712578	0,01614115	2
**LSM2**	6,0676E-07	0,29431708	0,01631996	2
**USB1**	6,2914E-07	0,44688725	0,01692205	2
**PSTPIP2**	6,3704E-07	0,69867821	0,01713444	2
**TMED4**	6,6259E-07	0,28471978	0,01782168	2
**BAX**	6,7222E-07	0,23063733	0,01808067	2
**NFATC2**	6,7987E-07	0,38562258	0,01828652	2
**SPCS2**	6,9291E-07	0,20974939	0,01863707	2
**S1PR1**	6,9295E-07	0,2208146	0,01863823	2
**SKIL**	6,9445E-07	0,84947823	0,01867862	2
**DMKN**	6,9448E-07	1,18014485	0,01867933	2
**PAFAH2**	7,0925E-07	0,97799015	0,01907672	2
**ARNTL**	7,3612E-07	0,39527198	0,01979947	2
**CCDC167**	7,3653E-07	0,33316315	0,01981039	2
**SRA1**	7,3738E-07	0,40190785	0,0198332	2
**PDHB**	7,403E-07	0,39034502	0,01991189	2
**PSMA6**	7,4043E-07	0,20493794	0,01991546	2
**TMBIM6**	7,4683E-07	0,1369342	0,02008752	2
**RBM18**	7,4934E-07	0,54388132	0,02015507	2
**LRIG1**	7,7202E-07	0,4917925	0,02076508	2
**GALM**	8,3745E-07	0,38292606	0,02252495	2
**ABCF2**	8,5831E-07	0,37574588	0,02308609	2

**NDUFA1**	8,7852E-07	0,11956506	0,02362954	2
**FABP5**	8,8118E-07	0,90101576	0,02370109	2
**UBE2L6**	9,159E-07	0,21724635	0,02463506	2
**STK24**	9,4092E-07	0,21835806	0,02530799	2
**S1PR4**	9,4452E-07	0,16891395	0,02540488	2
**EVA1C**	9,5025E-07	0,48476823	0,02555882	2
**CDCA4**	9,6344E-07	0,5925705	0,02591375	2
**POLE3**	1,0076E-06	0,32194311	0,02710011	2
**SMIM14**	1,0134E-06	0,55105418	0,02725705	2
**CPD**	1,0163E-06	0,50475187	0,02733616	2
**MAGOH**	1,021E-06	0,18657761	0,02746117	2
**SNRK**	1,0254E-06	0,32239218	0,02758039	2
**ALDH9A1**	1,0463E-06	0,35622288	0,02814216	2
**KLRD1**	1,0528E-06	1,48180839	0,02831745	2
**SP140**	1,0685E-06	0,31149053	0,0287404	2
**AL450992.2**	1,0794E-06	1,10764799	0,02903381	2
**SAP30**	1,0878E-06	0,54149731	0,02925897	2
**PRKCD**	1,1262E-06	0,60209828	0,03029114	2
**ADGRE5**	1,2005E-06	0,1825802	0,03229006	2
**ATP5J2**	1,2192E-06	0,15770591	0,03279391	2
**CD72**	1,2464E-06	1,02476352	0,03352526	2
**A2M-AS1**	1,2822E-06	0,90166859	0,03448685	2
**DNAJC8**	1,2842E-06	0,22266754	0,03454204	2
**S100PBP**	1,292E-06	0,43170704	0,03475105	2
**TRBV14**	1,4143E-06	0,78513218	0,03803972	2
**CLEC2D**	1,425E-06	0,18996535	0,0383293	2
**TRBV7-6**	1,4546E-06	0,67651567	0,03912495	2
**ATG16L2**	1,4895E-06	0,40357011	0,04006324	2
**GMIP**	1,5074E-06	0,42646128	0,04054511	2
**CREM**	1,5667E-06	0,41537511	0,04213944	2
**KIAA1551**	1,6046E-06	0,17455598	0,04315845	2
**RP11-239H6.2**	1,618E-06	1,03809488	0,04352044	2
**SLC38A10**	1,6225E-06	0,3808919	0,04363953	2
**GGT1**	1,6299E-06	0,63771536	0,04383965	2
**HOXB2**	1,6412E-06	0,3437804	0,04414286	2
**ATP5B**	1,6445E-06	0,12198121	0,04423212	2
**DEK**	1,6542E-06	0,18094998	0,04449424	2
**LINC01260**	1,6681E-06	1,03320424	0,04486822	2
**MAD2L2**	1,7409E-06	0,37915089	0,04682528	2
**RP11-47L3.1**	1,7551E-06	0,83204011	0,04720692	2
**PHKB**	1,7679E-06	0,46470771	0,04755067	2

**SLAMF1**	1,7832E-06	0,38304947	0,04796203	2
**ITGB1**	0	1,07673002	0	3
**USP10**	0	1,3439686	0	3
**EDA**	8,288E-195	1,93463693	2,229E-190	3
**TNFRSF4**	4,505E-188	1,12297901	1,212E-183	3
**CTSH**	4,722E-176	1,08036985	1,27E-171	3
**PI16**	6,116E-172	1,68650264	1,645E-167	3
**MAP3K1**	1,556E-171	1,21209903	4,184E-167	3
**MAL**	5,628E-163	0,6772643	1,514E-158	3
**NPDC1**	5,095E-160	1,16701758	1,371E-155	3
**TOB1**	4,301E-153	0,95092569	1,157E-148	3
**CMTM6**	5,795E-152	0,95107154	1,559E-147	3
**LTB**	2,087E-147	0,37961523	5,614E-143	3
**VIM**	3,759E-144	0,34518677	1,011E-139	3
**ARHGAP15**	1,386E-140	0,4838391	3,728E-136	3
**SLC40A1**	6,699E-139	1,45474764	1,802E-134	3
**FRY**	1,402E-135	2,03709573	3,77E-131	3
**BCL2**	2,49E-134	0,75551238	6,696E-130	3
**TAGLN2**	1,347E-123	0,48351377	3,624E-119	3
**FAM13A**	5,851E-123	2,06073502	1,574E-118	3
**CISH**	2,491E-119	1,10125609	6,7E-115	3
**GREM2**	1,076E-114	1,95702817	2,895E-110	3
**PDE4D**	1,251E-109	0,72863174	3,365E-105	3
**ALOX5**	1,608E-103	1,52430445	4,324E-99	3
**TAB2**	1,018E-100	0,75833651	2,7371E-96	3
**SOCS2**	4,466E-100	1,25475378	1,2011E-95	3
**SASH1**	8,403E-100	2,86151229	2,2602E-95	3
**PTPN13**	6,783E-99	1,60489539	1,8243E-94	3
**CR1**	1,817E-97	1,786891	4,8872E-93	3
**DST**	6,7042E-89	2,86889404	1,8032E-84	3
**ANK1**	2,4518E-86	1,92312859	6,5946E-82	3
**PKIA**	3,8843E-86	1,33689681	1,0448E-81	3
**AHNAK**	1,1395E-82	0,47005431	3,0649E-78	3
**NEFL**	1,6778E-79	1,99708731	4,5128E-75	3
**MAP3K4**	1,1854E-78	0,72883823	3,1883E-74	3
**RP11-124N14.3**	1,5015E-75	0,65617111	4,0386E-71	3
**CDK6**	1,8395E-75	0,85400414	4,9476E-71	3
**OSM**	6,9946E-75	1,21989631	1,8813E-70	3
**NCAPG2**	7,4671E-74	1,55375306	2,0084E-69	3
**FAM107B**	1,4063E-72	0,43123966	3,7826E-68	3
**MB21D2**	1,0567E-71	1,462026	2,8422E-67	3

**LEF1**	1,9421E-69	0,51379418	5,2236E-65	3
**CD55**	2,3957E-69	0,61877291	6,4438E-65	3
**TPO**	2,789E-69	2,87158202	7,5016E-65	3
**LMNA**	2,2104E-66	1,8623233	5,9454E-62	3
**CSGALNACT1**	1,5748E-65	0,9430787	4,2358E-61	3
**NELL2**	1,9711E-65	0,63335671	5,3017E-61	3
**ANXA1**	8,2004E-64	0,41987981	2,2057E-59	3
**C1orf186**	1,4093E-63	2,05808499	3,7907E-59	3
**MDFIC**	1,6625E-62	0,50813653	4,4716E-58	3
**RP11-382A20.3**	2,1161E-61	1,4814498	5,6918E-57	3
**RPL13A**	3,9505E-61	0,24181742	1,0626E-56	3
**GLUL**	4,3667E-61	0,89466588	1,1745E-56	3
**PIM1**	1,4757E-60	0,44353357	3,9692E-56	3
**MYADM**	2,0222E-60	0,93632071	5,439E-56	3
**BACH2**	1,7507E-58	0,62288554	4,7088E-54	3
**RUNX2**	1,6171E-57	0,66318463	4,3495E-53	3
**AUTS2**	4,7277E-57	0,67702578	1,2716E-52	3
**USP46**	8,0867E-57	0,93821225	2,1751E-52	3
**TTC39C-AS1**	8,4946E-57	0,88595183	2,2848E-52	3
**NFKB1**	1,534E-56	0,56953304	4,1259E-52	3
**RP11-342D11.3**	2,0623E-56	1,13915246	5,5469E-52	3
**CRIP2**	1,9644E-55	0,586692	5,2837E-51	3
**CTSA**	2,5311E-54	0,67621445	6,8078E-50	3
**SELL**	5,4514E-54	0,34950541	1,4663E-49	3
**TNFRSF18**	1,4446E-53	0,7696464	3,8854E-49	3
**LRRFIP1**	1,1165E-52	0,43571144	3,0031E-48	3
**RORA**	3,3896E-51	0,34719569	9,1169E-47	3
**TSPAN2**	5,157E-51	1,21432743	1,3871E-46	3
**NSG1**	9,9337E-51	0,4823728	2,6719E-46	3
**GPR25**	1,5037E-50	1,14928607	4,0444E-46	3
**TIMP1**	7,2507E-50	0,3605927	1,9502E-45	3
**FOS**	3,0029E-49	0,53664758	8,0768E-45	3
**GRASP**	6,1869E-49	0,73806106	1,6641E-44	3
**IL7R**	1,8235E-48	0,18515283	4,9047E-44	3
**KLHL5**	4,2726E-48	0,67725074	1,1492E-43	3
**NOSIP**	1,0904E-46	0,25121616	2,9328E-42	3
**S100A10**	5,1013E-46	0,24496653	1,3721E-41	3
**TNFAIP3**	1,1894E-44	0,43659853	3,199E-40	3
**C4orf32**	5,2023E-44	0,81020449	1,3993E-39	3
**CCR7**	1,5646E-43	0,42163243	4,2082E-39	3
**WDFY2**	4,2552E-43	0,71429413	1,1445E-38	3

**PLCL1**	7,4301E-43	0,85902503	1,9985E-38	3
**CRIP1**	9,2734E-43	0,3521247	2,4943E-38	3
**RFX2**	1,0456E-42	1,70387779	2,8124E-38	3
**PRKCA**	1,1119E-42	0,53621052	2,9907E-38	3
**RARA**	1,5605E-42	0,64735524	4,1973E-38	3
**ATP2B1**	3,879E-42	0,54519325	1,0433E-37	3
**HPGD**	5,7107E-42	1,01593809	1,536E-37	3
**EEPD1**	6,3181E-42	0,80984181	1,6994E-37	3
**RGCC**	1,4295E-41	0,62281905	3,845E-37	3
**ADAM23**	4,3995E-41	0,93246322	1,1833E-36	3
**TRIQK**	8,2668E-41	1,06026656	2,2235E-36	3
**ADGRE5**	1,1252E-40	0,42294661	3,0264E-36	3
**MLLT4**	2,9317E-40	0,80920075	7,8853E-36	3
**KAT2B**	4,0702E-40	0,64675393	1,0948E-35	3
**MTUS2**	2,2228E-39	2,80518402	5,9786E-35	3
**LPGAT1**	2,7906E-38	0,57501746	7,5059E-34	3
**TMEM123**	7,355E-38	0,30041961	1,9783E-33	3
**TUBA1A**	1,3395E-37	0,39682369	3,6029E-33	3
**AFF1**	1,7602E-37	0,54370492	4,7344E-33	3
**GPRIN3**	2,2228E-37	0,44931606	5,9787E-33	3
**SAMHD1**	6,2679E-37	0,30053396	1,6859E-32	3
**KLF7**	6,4729E-37	1,16615865	1,741E-32	3
**RIPK2**	7,3519E-37	0,60085705	1,9774E-32	3
**AQP3**	3,9623E-36	0,25063865	1,0657E-31	3
**REEP3**	1,876E-35	0,53435876	5,0458E-31	3
**PLP2**	2,7481E-35	0,26387429	7,3917E-31	3
**JUND**	5,1657E-35	0,2257075	1,3894E-30	3
**PPP1R15A**	1,4081E-34	0,53941316	3,7873E-30	3
**GPR146**	1,4669E-34	1,00248806	3,9454E-30	3
**NEDD4L**	2,2624E-34	1,98244647	6,0853E-30	3
**ADAM19**	5,4032E-34	0,72296997	1,4533E-29	3
**SOS1**	8,796E-34	0,52118344	2,3659E-29	3
**EGLN3**	1,3339E-33	0,79129679	3,5878E-29	3
**AGPAT4**	2,9529E-33	0,62082406	7,9423E-29	3
**CDC14A**	3,7789E-33	0,41607784	1,0164E-28	3
**BHLHE40**	7,9259E-33	0,37796143	2,1318E-28	3
**ADAM10**	1,1609E-32	0,40256842	3,1226E-28	3
**MAP1A**	1,2474E-32	2,17252031	3,3552E-28	3
**PTGER2**	3,3318E-32	0,32710738	8,9616E-28	3
**UBE2H**	3,4962E-32	0,53022625	9,4036E-28	3
**PIM2**	8,4155E-32	0,35085686	2,2635E-27	3

**S100A11**	8,6535E-32	0,26036338	2,3275E-27	3
**LFNG**	4,9798E-31	0,51798214	1,3394E-26	3
**FXYD7**	7,4061E-31	0,61099908	1,992E-26	3
**DOCK10**	1,0624E-30	0,33557159	2,8576E-26	3
**FLT3LG**	1,092E-30	0,21259488	2,9372E-26	3
**SLC2A3**	1,475E-30	0,38064679	3,9672E-26	3
**PTPN4**	3,3479E-30	0,37882875	9,0048E-26	3
**SOCS3**	1,0587E-29	0,52224923	2,8477E-25	3
**GATA3**	1,2266E-29	0,35462441	3,2991E-25	3
**TRABD2A**	1,4791E-29	0,43862859	3,9784E-25	3
**SNTG2**	2,6177E-29	1,80240987	7,0408E-25	3
**PBXIP1**	3,182E-29	0,296422	8,5586E-25	3
**SYNE3**	3,6623E-29	0,80482756	9,8505E-25	3
**RBMS1**	6,3326E-29	0,32965422	1,7033E-24	3
**WNT7A**	8,7844E-29	0,72640748	2,3627E-24	3
**SVIL**	1,5889E-28	0,50458998	4,2737E-24	3
**AP3M2**	2,3379E-28	0,52135765	6,2883E-24	3
**SUSD4**	5,1173E-28	1,17347923	1,3764E-23	3
**ANK3**	8,7766E-28	0,35316294	2,3606E-23	3
**MCL1**	9,3699E-28	0,3155484	2,5202E-23	3
**FAM129A**	1,1124E-27	0,5014989	2,992E-23	3
**C16orf54**	1,1949E-27	0,45956277	3,214E-23	3
**SATB1**	1,4705E-27	0,34426234	3,9551E-23	3
**THOC3**	1,7997E-27	0,36087092	4,8405E-23	3
**ZC3H12D**	1,969E-27	0,73087464	5,296E-23	3
**AP000769.1**	6,4287E-27	0,56672768	1,7291E-22	3
**LGALS1**	8,0819E-27	0,31269253	2,1738E-22	3
**ADK**	2,085E-26	0,35766037	5,608E-22	3
**COL5A3**	2,5528E-26	0,91969289	6,8663E-22	3
**MYOM2**	2,8958E-26	1,54505779	7,7889E-22	3
**SNX9**	5,2727E-26	0,40763796	1,4182E-21	3
**RP11-356J5.12**	5,7767E-26	0,70770271	1,5538E-21	3
**KANK1**	1,3767E-25	1,16068303	3,7029E-21	3
**EPHA4**	1,585E-25	0,60542024	4,2633E-21	3
**CA5B**	1,7961E-25	0,36902954	4,831E-21	3
**VIPR1**	2,0484E-25	0,48799518	5,5095E-21	3
**FXYD5**	2,1341E-25	0,11036834	5,7401E-21	3
**UBE2Q2**	3,6054E-25	0,40011085	9,6974E-21	3
**BCL3**	3,9466E-25	0,35628891	1,0615E-20	3
**FLNB**	5,288E-25	0,80796326	1,4223E-20	3
**BIRC3**	5,795E-25	0,35965935	1,5587E-20	3

**SNED1**	7,031E-25	1,00883815	1,8911E-20	3
**WHSC1L1**	7,446E-25	0,27120713	2,0027E-20	3
**ARL4A**	7,6964E-25	0,40611025	2,0701E-20	3
**SPTAN1**	8,4161E-25	0,28078779	2,2637E-20	3
**WDR86**	2,7982E-24	0,62690698	7,5264E-20	3
**RP11-78B10.2**	3,0271E-24	2,41831255	8,1419E-20	3
**AKT3**	3,8627E-24	0,33876954	1,0389E-19	3
**ISM1**	3,9424E-24	1,16933898	1,0604E-19	3
**KIF5C**	5,1392E-24	0,54750311	1,3823E-19	3
**RP11-195F19.9**	8,0215E-24	0,86529675	2,1576E-19	3
**ESYT2**	1,0615E-23	0,36017221	2,8551E-19	3
**SUPT3H**	1,167E-23	0,33541181	3,139E-19	3
**CAPG**	1,6774E-23	0,49973542	4,5117E-19	3
**DUSP16**	2,0258E-23	0,47267982	5,4489E-19	3
**RCAN3**	2,2414E-23	0,28021086	6,0288E-19	3
**DPP4**	2,7082E-23	0,32384904	7,2842E-19	3
**RPL37A**	3,7307E-23	0,11788796	1,0034E-18	3
**ZFP36**	6,5095E-23	0,25784939	1,7509E-18	3
**CAST**	8,9096E-23	0,25417038	2,3964E-18	3
**CYLD**	1,794E-22	0,28844687	4,8253E-18	3
**PIM3**	2,4878E-22	0,27245232	6,6915E-18	3
**TSPAN15**	2,5365E-22	0,60266282	6,8224E-18	3
**FAM19A1**	2,549E-22	0,63781416	6,856E-18	3
**FHL1**	2,8895E-22	0,39679275	7,7718E-18	3
**LDLRAD4**	3,2848E-22	0,45969417	8,835E-18	3
**RORA-AS1**	3,5297E-22	0,40983435	9,4939E-18	3
**CCND2**	4,9896E-22	0,34790752	1,3421E-17	3
**TESPA1**	6,9182E-22	0,27893494	1,8608E-17	3
**S100A6**	7,5488E-22	0,11911308	2,0304E-17	3
**FBXL16**	7,7715E-22	0,36730649	2,0903E-17	3
**FLNA**	8,5002E-22	0,22037454	2,2863E-17	3
**HIST1H4C**	1,1621E-21	0,18831356	3,1257E-17	3
**FNDC3B**	1,1846E-21	0,61764102	3,1862E-17	3
**FAM63B**	1,2373E-21	0,55713025	3,3279E-17	3
**TAGAP**	1,5454E-21	0,17575719	4,1567E-17	3
**EHD4**	1,8037E-21	0,54524333	4,8513E-17	3
**CHD1**	2,298E-21	0,31746554	6,181E-17	3
**AIM1**	2,666E-21	0,34666023	7,1706E-17	3
**HTATSF1**	4,1082E-21	0,31172361	1,105E-16	3
**PAG1**	4,8663E-21	0,317856	1,3089E-16	3
**N4BP2**	5,7729E-21	0,55523285	1,5527E-16	3

**RFX3**	6,1406E-21	0,50456107	1,6516E-16	3
**SLCO3A1**	7,7457E-21	0,4306314	2,0834E-16	3
**RGMB**	9,7021E-21	0,89818902	2,6096E-16	3
**TCP11L2**	1,1049E-20	0,38259024	2,9717E-16	3
**PDS5A**	1,2994E-20	0,32435054	3,4949E-16	3
**DAAM1**	1,3388E-20	0,65395042	3,6011E-16	3
**NTN4**	1,4601E-20	0,88378613	3,9272E-16	3
**TMEM156**	1,5446E-20	0,40653147	4,1544E-16	3
**RPL23**	1,6593E-20	0,14862985	4,4629E-16	3
**KIAA0355**	1,678E-20	0,6923404	4,5133E-16	3
**TXN**	1,6826E-20	0,29681481	4,5256E-16	3
**TRAK2**	2,1852E-20	0,45787886	5,8775E-16	3
**CKAP4**	2,3076E-20	0,56381248	6,2067E-16	3
**EEF2**	2,5891E-20	0,10482556	6,9638E-16	3
**SORL1**	2,886E-20	0,29088029	7,7625E-16	3
**RPL27A**	3,3305E-20	0,10608387	8,9581E-16	3
**ABO**	3,8564E-20	1,29005719	1,0372E-15	3
**MGAT5**	4,641E-20	0,37017567	1,2483E-15	3
**KLF8**	4,7437E-20	1,27364399	1,2759E-15	3
**STXBP1**	4,7629E-20	1,3342441	1,2811E-15	3
**IL4I1**	5,5083E-20	0,62187343	1,4816E-15	3
**CCNY**	5,8878E-20	0,28818069	1,5836E-15	3
**MCOLN3**	6,1192E-20	1,56345024	1,6459E-15	3
**LL22NC03-75H12.2**	6,2695E-20	0,53486592	1,6863E-15	3
**CDK11A**	6,7086E-20	0,45545248	1,8044E-15	3
**AC008937.2**	7,5863E-20	1,31073482	2,0405E-15	3
**PDE3B**	9,6735E-20	0,23512027	2,6019E-15	3
**DUSP1**	1,4694E-19	0,27198619	3,9521E-15	3
**DPYD**	1,8205E-19	0,25217449	4,8967E-15	3
**LTA**	2,6465E-19	0,496608	7,1183E-15	3
**FUT7**	3,6897E-19	1,38719018	9,9241E-15	3
**PTTG1IP**	3,8291E-19	0,35877487	1,0299E-14	3
**RASA3**	3,8606E-19	0,23285712	1,0384E-14	3
**DDX21**	4,169E-19	0,25156526	1,1213E-14	3
**TNFSF13B**	4,9315E-19	0,3595392	1,3264E-14	3
**MAPK10**	9,3287E-19	2,22263749	2,5091E-14	3
**IER2**	1,0667E-18	0,21616934	2,8692E-14	3
**IGSF9B**	1,067E-18	1,45486193	2,8699E-14	3
**PLCL2**	1,0838E-18	0,27122649	2,915E-14	3
**SAMD12**	1,1011E-18	0,65626885	2,9618E-14	3
**TSPAN32**	1,2057E-18	0,48781527	3,2429E-14	3

**TXK**	1,4283E-18	0,36357599	3,8416E-14	3
**LST1**	1,8471E-18	0,33827521	4,9682E-14	3
**KLF3**	3,3965E-18	0,21641228	9,1356E-14	3
**UCP2**	3,6125E-18	0,23310142	9,7165E-14	3
**IER5**	3,9129E-18	0,37753852	1,0524E-13	3
**ZNF462**	5,5111E-18	1,61312557	1,4823E-13	3
**AC016831.7**	6,5323E-18	0,60892585	1,757E-13	3
**PAK2**	7,4366E-18	0,25036761	2,0002E-13	3
**RP11-229P13.19**	8,4423E-18	0,80194923	2,2707E-13	3
**PHTF2**	9,4099E-18	0,37097634	2,531E-13	3
**YWHAH**	9,8052E-18	0,21949123	2,6373E-13	3
**CYTH1**	9,9719E-18	0,23069719	2,6821E-13	3
**MTPN**	9,9825E-18	0,21747177	2,685E-13	3
**REXO2**	1,0254E-17	0,26783866	2,758E-13	3
**WDR59**	1,0466E-17	0,44921758	2,815E-13	3
**ANXA2**	1,5985E-17	0,19128556	4,2995E-13	3
**CFH**	1,8698E-17	0,43589269	5,0293E-13	3
**SPTBN1**	1,9023E-17	0,31042279	5,1167E-13	3
**P2RY8**	2,0353E-17	0,22730164	5,4744E-13	3
**IL2RA**	2,2105E-17	0,60221623	5,9456E-13	3
**JUN**	2,3427E-17	0,28265444	6,3012E-13	3
**FMN1**	2,5412E-17	1,11581319	6,8351E-13	3
**SELPLG**	2,7074E-17	0,17695678	7,282E-13	3
**PEPD**	3,1033E-17	0,27415868	8,347E-13	3
**DAB1**	3,2477E-17	0,84790973	8,7353E-13	3
**SIGMAR1**	3,3639E-17	0,32166485	9,0478E-13	3
**LINC01006**	3,4871E-17	1,17121637	9,3793E-13	3
**CCR6**	3,5306E-17	0,2620602	9,4963E-13	3
**CFAP36**	5,2209E-17	0,28782133	1,4043E-12	3
**ARL6IP5**	5,6419E-17	0,15822784	1,5175E-12	3
**LINC-PINT**	5,8101E-17	0,26283858	1,5627E-12	3
**IL4R**	5,977E-17	0,44472697	1,6076E-12	3
**MEF2A**	6,3972E-17	0,3786806	1,7206E-12	3
**GOLGB1**	7,2974E-17	0,34187911	1,9628E-12	3
**PDP1**	7,4167E-17	0,54467088	1,9949E-12	3
**ZFP36L1**	8,1873E-17	0,219025	2,2022E-12	3
**RPS11**	8,3573E-17	0,11214853	2,2479E-12	3
**FAM126A**	1,0826E-16	0,67192552	2,9118E-12	3
**STAT5B**	1,1072E-16	0,29497037	2,9781E-12	3
**KAT6B**	1,11E-16	0,33279785	2,9855E-12	3
**SNHG15**	1,1748E-16	0,29471533	3,1599E-12	3

**KDSR**	1,2041E-16	0,25953415	3,2388E-12	3
**SIPA1L1**	1,4652E-16	0,36783478	3,9409E-12	3
**MT-ND5**	1,812E-16	0,1145034	4,8737E-12	3
**HAPLN3**	1,9219E-16	0,44906352	5,1694E-12	3
**AP1S2**	2,2222E-16	0,26471621	5,9771E-12	3
**GPHN**	3,0371E-16	0,33095299	8,169E-12	3
**PRKX**	3,409E-16	0,29910401	9,1693E-12	3
**RTKN2**	3,4389E-16	0,90181513	9,2497E-12	3
**ELOVL4**	3,5443E-16	0,54338688	9,5331E-12	3
**ANXA5**	4,3833E-16	0,19372078	1,179E-11	3
**RHBDL1**	4,5061E-16	0,68166449	1,212E-11	3
**RARG**	4,8144E-16	0,42359738	1,2949E-11	3
**KCNN4**	5,4665E-16	0,69372425	1,4703E-11	3
**PELI2**	5,666E-16	0,61355429	1,524E-11	3
**SMAP1**	6,111E-16	0,2247713	1,6437E-11	3
**ZBTB20**	6,3816E-16	0,23242269	1,7165E-11	3
**GLUD1**	7,0245E-16	0,25942755	1,8894E-11	3
**EIF2S3**	7,551E-16	0,20219816	2,031E-11	3
**PCGF5**	8,8477E-16	0,21321073	2,3798E-11	3
**HIVEP1**	9,1726E-16	0,50667821	2,4672E-11	3
**TMED8**	9,749E-16	0,70834407	2,6222E-11	3
**INTS6**	1,0059E-15	0,29746571	2,7056E-11	3
**NGFRAP1**	1,1915E-15	0,31770058	3,2047E-11	3
**HSPA8**	1,6577E-15	0,12097184	4,4587E-11	3
**LSR**	1,6803E-15	0,25768134	4,5195E-11	3
**RP11-779O18.3**	1,9361E-15	0,3510783	5,2076E-11	3
**CMTM8**	1,9623E-15	0,31508993	5,2781E-11	3
**SERINC5**	1,9746E-15	0,22228397	5,311E-11	3
**GLG1**	2,0348E-15	0,24735891	5,4731E-11	3
**SSBP2**	2,0359E-15	0,26764511	5,476E-11	3
**AGAP1**	2,0726E-15	0,72625411	5,5747E-11	3
**UGCG**	2,2791E-15	0,37884758	6,1301E-11	3
**TUBB4B**	3,1133E-15	0,25874066	8,374E-11	3
**TRADD**	3,377E-15	0,17652441	9,0832E-11	3
**PTP4A1**	3,4276E-15	0,23214918	9,2193E-11	3
**TUBB2A**	3,5417E-15	0,54338164	9,526E-11	3
**KLF9**	4,0268E-15	0,29109345	1,0831E-10	3
**RPS6KA3**	4,7188E-15	0,2395088	1,2692E-10	3
**HIF1A**	4,9919E-15	0,30772833	1,3427E-10	3
**GAB2**	5,1157E-15	0,99150072	1,376E-10	3
**JUNB**	5,2333E-15	0,16261295	1,4076E-10	3

**SERP1**	5,4178E-15	0,13219647	1,4572E-10	3
**USP3**	5,8482E-15	0,23695181	1,573E-10	3
**CXorf57**	7,3405E-15	0,74903112	1,9744E-10	3
**AKAP13**	7,5564E-15	0,20613311	2,0324E-10	3
**RAMP1**	8,0472E-15	1,65094583	2,1644E-10	3
**CD2AP**	8,2204E-15	0,31540948	2,211E-10	3
**TLE4**	1,1603E-14	0,25889202	3,1209E-10	3
**STX2**	1,2178E-14	0,35282128	3,2754E-10	3
**SH3BP5**	1,5072E-14	0,21426748	4,0538E-10	3
**RP11-570L15.1**	1,5437E-14	0,78938214	4,1521E-10	3
**DNAH6**	1,6051E-14	0,96286958	4,3173E-10	3
**NBPF15**	1,7126E-14	0,3675351	4,6064E-10	3
**FAM65B**	1,9437E-14	0,1930209	5,228E-10	3
**AC018816.3**	2,1982E-14	0,5296608	5,9125E-10	3
**GSPT1**	2,3775E-14	0,20459627	6,3947E-10	3
**PFKFB3**	2,4462E-14	0,38702291	6,5795E-10	3
**CSRNP1**	2,6413E-14	0,31598682	7,1044E-10	3
**FOSL2**	2,7268E-14	0,3785389	7,3344E-10	3
**GALT**	2,7942E-14	0,36358044	7,5155E-10	3
**PER1**	2,8199E-14	0,29713847	7,5846E-10	3
**PLEC**	2,8342E-14	0,29913633	7,6231E-10	3
**ZNF516**	2,8815E-14	0,87882318	7,7505E-10	3
**HK1**	3,0379E-14	0,257638	8,1712E-10	3
**RALGPS2**	3,0393E-14	0,68156328	8,1749E-10	3
**ATP1A1**	3,2337E-14	0,21891478	8,6977E-10	3
**AGPAT5**	3,2694E-14	0,37983181	8,7937E-10	3
**ACTR2**	3,3793E-14	0,1856217	9,0894E-10	3
**IL6R**	3,4436E-14	0,37319735	9,2624E-10	3
**RIN3**	3,5214E-14	0,37131956	9,4714E-10	3
**ZNF331**	3,6116E-14	0,27169822	9,714E-10	3
**PPP3CA**	3,7074E-14	0,30842182	9,9718E-10	3
**EIF4B**	3,8563E-14	0,16697709	1,0372E-09	3
**RAB11A**	4,3517E-14	0,21401545	1,1705E-09	3
**ATP8B2**	4,9428E-14	0,28788251	1,3295E-09	3
**FAM153C**	4,9455E-14	0,73015044	1,3302E-09	3
**G3BP1**	5,4834E-14	0,18141897	1,4749E-09	3
**ERO1A**	5,712E-14	0,25861395	1,5363E-09	3
**PCSK5**	5,7414E-14	0,61382694	1,5443E-09	3
**BAG3**	5,7825E-14	0,3385948	1,5553E-09	3
**IVNS1ABP**	6,1345E-14	0,2757355	1,65E-09	3
**DCTPP1**	6,4247E-14	0,29103921	1,7281E-09	3

**JMJD1C**	6,5288E-14	0,23483536	1,7561E-09	3
**NAP1L1**	6,6062E-14	0,16364713	1,7769E-09	3
**AHR**	6,6297E-14	0,32922739	1,7832E-09	3
**DENND4A**	6,8787E-14	0,32385867	1,8502E-09	3
**MPRIP**	6,97E-14	0,20921962	1,8747E-09	3
**MIB1**	7,3834E-14	0,47690754	1,9859E-09	3
**PCED1B**	7,8252E-14	0,22206033	2,1047E-09	3
**EPC2**	7,9077E-14	0,30488643	2,1269E-09	3
**ARL3**	8,9151E-14	0,34119682	2,3979E-09	3
**FBXL3**	9,6426E-14	0,32088135	2,5936E-09	3
**NCALD**	1,0618E-13	0,28049889	2,8561E-09	3
**CDC42BPB**	1,0841E-13	1,45542985	2,9158E-09	3
**ESYT1**	1,1063E-13	0,23632619	2,9756E-09	3
**UBE2D1**	1,192E-13	0,29594961	3,206E-09	3
**VSIG1**	1,2115E-13	0,35790256	3,2586E-09	3
**NXT1**	1,2714E-13	0,25035232	3,4197E-09	3
**VPS50**	1,2752E-13	0,4654574	3,4299E-09	3
**AC103563.8**	1,3581E-13	0,44612181	3,653E-09	3
**SPOCK2**	1,4379E-13	0,14463345	3,8676E-09	3
**RASA1**	1,4418E-13	0,3670303	3,8781E-09	3
**PLXNA4**	1,5175E-13	1,23049046	4,0816E-09	3
**RPS6KA5**	1,6346E-13	0,26335368	4,3966E-09	3
**NHSL2**	1,6661E-13	0,61973819	4,4813E-09	3
**SGK1**	1,6701E-13	0,6002613	4,4921E-09	3
**PGAP1**	1,9579E-13	1,04497546	5,266E-09	3
**FOSB**	2,026E-13	0,47709023	5,4493E-09	3
**ICAM2**	2,0927E-13	0,19332185	5,6286E-09	3
**RP11-290D2.6**	2,1661E-13	0,1512557	5,8261E-09	3
**LINC00511**	2,1758E-13	1,68020971	5,8523E-09	3
**CCSAP**	2,2963E-13	0,62014272	6,1763E-09	3
**GNAQ**	2,3265E-13	0,41725426	6,2575E-09	3
**BTG2**	2,4583E-13	0,20255682	6,6121E-09	3
**ADD3**	3,4044E-13	0,16874781	9,1567E-09	3
**PCSK1N**	3,5328E-13	0,47286054	9,5021E-09	3
**DOCK11**	3,6671E-13	0,26389371	9,8634E-09	3
**ODF2L**	3,9713E-13	0,20216307	1,0682E-08	3
**NT5E**	4,2533E-13	0,49808751	1,144E-08	3
**RALGDS**	4,4634E-13	0,29509307	1,2005E-08	3
**ETS1**	4,6357E-13	0,12701935	1,2469E-08	3
**WHAMM**	5,1357E-13	0,24886389	1,3813E-08	3
**RAB11FIP1**	5,2393E-13	0,41658025	1,4092E-08	3

**B4GALT1**	7,875E-13	0,29442715	2,1181E-08	3
**FAM69A**	8,207E-13	0,27696776	2,2074E-08	3
**SLC39A8**	8,3919E-13	0,28552986	2,2572E-08	3
**GATAD2B**	9,2445E-13	0,34121033	2,4865E-08	3
**DDIT4**	9,3379E-13	0,21935686	2,5116E-08	3
**HOOK2**	9,7702E-13	0,29416825	2,6279E-08	3
**FAM35A**	1,0223E-12	0,36591417	2,7497E-08	3
**ENTPD4**	1,1319E-12	0,37144291	3,0446E-08	3
**TMF1**	1,167E-12	0,24191355	3,1388E-08	3
**MAML2**	1,1688E-12	0,16542865	3,1438E-08	3
**CEP350**	1,305E-12	0,24610259	3,51E-08	3
**DCAF12**	1,3587E-12	0,46010923	3,6545E-08	3
**LACTB**	1,4213E-12	0,45997881	3,823E-08	3
**EFCAB14**	1,5272E-12	0,28560075	4,1076E-08	3
**AIG1**	1,5629E-12	0,32471142	4,2038E-08	3
**CDKN2D**	1,6774E-12	0,14341214	4,5117E-08	3
**TNF**	2,0183E-12	0,38372193	5,4286E-08	3
**ZC2HC1A**	2,1329E-12	1,02586572	5,7368E-08	3
**STT3B**	2,3742E-12	0,18466747	6,3859E-08	3
**MED25**	2,4316E-12	0,44798098	6,5403E-08	3
**MYLIP**	2,4436E-12	0,25450037	6,5726E-08	3
**TMEM43**	2,4822E-12	0,28638047	6,6763E-08	3
**NRIP1**	2,6862E-12	0,39175835	7,2252E-08	3
**SYNJ2**	2,8688E-12	0,30352974	7,7161E-08	3
**FBXL8**	2,9651E-12	0,32335003	7,9752E-08	3
**CAMTA1**	3,0366E-12	0,23283611	8,1676E-08	3
**YWHAQ**	3,1006E-12	0,14061033	8,3398E-08	3
**HEATR5B**	3,3263E-12	0,37047739	8,9466E-08	3
**FAM134B**	3,6016E-12	0,28426003	9,6871E-08	3
**MAFF**	3,6024E-12	0,61708286	9,6893E-08	3
**CRY1**	3,6104E-12	0,29297283	9,7109E-08	3
**ATP2A2**	3,7794E-12	0,40566161	1,0165E-07	3
**XYLT1**	4,4423E-12	0,47360052	1,1949E-07	3
**MAP3K2**	4,7671E-12	0,27910575	1,2822E-07	3
**RP11-356I2.4**	4,7825E-12	0,40045856	1,2863E-07	3
**RGS16**	5,381E-12	1,52112273	1,4473E-07	3
**WDR43**	5,6954E-12	0,24066072	1,5319E-07	3
**PAN3**	5,8075E-12	0,25407428	1,562E-07	3
**MT-ND6**	5,9347E-12	0,16035817	1,5963E-07	3
**HSD11B1**	6,4724E-12	0,96252883	1,7409E-07	3
**NCOA7**	6,5385E-12	0,20326702	1,7587E-07	3

**HIVEP2**	6,7895E-12	0,2403895	1,8262E-07	3
**RORC**	7,0826E-12	0,24523915	1,905E-07	3
**POLR2A**	7,1486E-12	0,22715604	1,9228E-07	3
**MALT1**	7,4954E-12	0,21046554	2,016E-07	3
**G0S2**	7,7601E-12	1,17941157	2,0872E-07	3
**TMEM65**	8,8635E-12	0,31523002	2,384E-07	3
**LARP1**	1,0297E-11	0,30803077	2,7696E-07	3
**GFOD2**	1,0731E-11	0,55863329	2,8863E-07	3
**MAGI3**	1,1152E-11	0,51600288	2,9996E-07	3
**RANBP9**	1,1242E-11	0,24574685	3,0238E-07	3
**KIAA2026**	1,1412E-11	0,39393346	3,0696E-07	3
**CRYBG3**	1,1508E-11	0,60267963	3,0953E-07	3
**NEAT1**	1,1607E-11	0,15987854	3,122E-07	3
**VCL**	1,2599E-11	0,28802546	3,3887E-07	3
**IARS2**	1,3124E-11	0,26032433	3,5301E-07	3
**ITSN2**	1,3492E-11	0,19157869	3,6291E-07	3
**DDX3X**	1,3925E-11	0,19247536	3,7455E-07	3
**DENND5A**	1,4408E-11	0,5735359	3,8752E-07	3
**C11orf21**	1,5171E-11	0,40282919	4,0804E-07	3
**SUN1**	1,6222E-11	0,36900788	4,3631E-07	3
**WRN**	1,6695E-11	0,53013304	4,4904E-07	3
**PLK3**	1,6815E-11	0,34117524	4,5228E-07	3
**AC058791.1**	1,896E-11	0,47125195	5,0998E-07	3
**TNIK**	1,9969E-11	0,22105552	5,3709E-07	3
**CARS**	2,0986E-11	0,31018047	5,6446E-07	3
**COMTD1**	2,1436E-11	0,26296194	5,7658E-07	3
**CD46**	2,1895E-11	0,17947434	5,889E-07	3
**NUFIP2**	2,3202E-11	0,21878643	6,2407E-07	3
**OGDH**	2,3416E-11	0,19269718	6,2981E-07	3
**CDK17**	2,3959E-11	0,22373956	6,4442E-07	3
**NFKBIZ**	2,427E-11	0,22370898	6,5278E-07	3
**C1GALT1**	2,5607E-11	0,17193702	6,8876E-07	3
**LMBR1**	2,5784E-11	0,31667672	6,9352E-07	3
**NME2**	2,5886E-11	0,14813001	6,9625E-07	3
**SBK1**	2,6139E-11	0,41824861	7,0307E-07	3
**PASK**	2,6199E-11	0,16339488	7,0467E-07	3
**LPIN2**	2,8968E-11	0,1921146	7,7915E-07	3
**TIAM1**	2,9868E-11	0,57374423	8,0336E-07	3
**USP14**	3,118E-11	0,2376405	8,3865E-07	3
**USP22**	3,3541E-11	0,23580834	9,0216E-07	3
**RAPGEF1**	3,6812E-11	0,23161809	9,9012E-07	3

**HGSNAT**	3,6868E-11	0,3406732	9,9164E-07	3
**MAP4**	3,8437E-11	0,23855565	1,0339E-06	3
**HNRNPAB**	4,1416E-11	0,21393164	1,114E-06	3
**POLH**	4,4273E-11	0,44302919	1,1908E-06	3
**GABBR1**	4,6512E-11	0,43371402	1,251E-06	3
**ELK3**	4,7847E-11	0,22896349	1,2869E-06	3
**ROCK1**	4,9098E-11	0,18204622	1,3206E-06	3
**PDE4B**	5,028E-11	0,20980329	1,3524E-06	3
**RNF157**	5,1195E-11	0,4105462	1,377E-06	3
**LDHA**	5,1872E-11	0,12106626	1,3952E-06	3
**IMPDH2**	5,2987E-11	0,15415038	1,4252E-06	3
**MCF2L2**	5,8097E-11	0,64148821	1,5626E-06	3
**HOOK3**	5,8331E-11	0,25453095	1,5689E-06	3
**NIPA2**	5,9864E-11	0,31312384	1,6102E-06	3
**CLSTN1**	6,0272E-11	0,25915945	1,6211E-06	3
**PACSIN2**	6,379E-11	0,40906209	1,7158E-06	3
**MCMBP**	6,4668E-11	0,25699427	1,7394E-06	3
**BMI1**	6,7443E-11	0,24478506	1,814E-06	3
**MED13L**	6,8521E-11	0,31954435	1,843E-06	3
**TTC3**	6,9241E-11	0,18255731	1,8624E-06	3
**TNKS2**	6,9373E-11	0,18021139	1,8659E-06	3
**SERPINB9**	6,9836E-11	0,22577155	1,8784E-06	3
**JAM3**	7,0857E-11	0,94662692	1,9058E-06	3
**USP3-AS1**	7,297E-11	0,4639666	1,9627E-06	3
**FAM153B**	7,6126E-11	0,87881428	2,0476E-06	3
**CEP112**	7,8827E-11	0,78215974	2,1202E-06	3
**KMT2C**	8,0675E-11	0,20160033	2,1699E-06	3
**INPP4B**	8,5263E-11	0,1774476	2,2933E-06	3
**PLCD1**	9,0859E-11	0,34097329	2,4438E-06	3
**CAMK1D**	9,2476E-11	0,20364717	2,4873E-06	3
**UBQLN2**	9,2823E-11	0,22776477	2,4967E-06	3
**BCR**	9,6845E-11	0,26350472	2,6048E-06	3
**ZDHHC11B**	1,0301E-10	1,55791466	2,7705E-06	3
**CCDC53**	1,0351E-10	0,17336731	2,7841E-06	3
**IPO7**	1,111E-10	0,20092966	2,9882E-06	3
**KAT6A**	1,2188E-10	0,2142146	3,2782E-06	3
**LEPROT**	1,2903E-10	0,29727157	3,4705E-06	3
**ST6GALNAC1**	1,3595E-10	0,64803332	3,6566E-06	3
**EXOC8**	1,4405E-10	0,3804715	3,8744E-06	3
**SCCPDH**	1,5045E-10	0,65386588	4,0467E-06	3
**TMEM50A**	1,5294E-10	0,13128148	4,1135E-06	3

**ASXL1**	1,5463E-10	0,17048966	4,1591E-06	3
**TP53BP1**	1,5543E-10	0,44359929	4,1807E-06	3
**EHMT1**	1,5696E-10	0,20318294	4,2217E-06	3
**GPAT3**	1,7393E-10	0,4595468	4,6783E-06	3
**RPGR**	1,7738E-10	0,62186175	4,7711E-06	3
**RRAGD**	1,7836E-10	0,36591814	4,7974E-06	3
**MARCH8**	1,8809E-10	0,35406067	5,0591E-06	3
**PBRM1**	2,0138E-10	0,22345497	5,4164E-06	3
**PIK3CB**	2,028E-10	0,39525405	5,4546E-06	3
**SLC25A24**	2,099E-10	0,23826115	5,6457E-06	3
**GDI1**	2,2034E-10	0,18156816	5,9264E-06	3
**EFCAB2**	2,3099E-10	0,3991387	6,213E-06	3
**WIPI2**	2,3536E-10	0,20531295	6,3304E-06	3
**LILRB3**	2,4043E-10	1,04597516	6,4669E-06	3
**FAM175A**	2,6777E-10	0,22726747	7,2023E-06	3
**PRKDC**	2,6809E-10	0,17518933	7,2109E-06	3
**EMP1**	2,7108E-10	1,44486117	7,2913E-06	3
**ARHGAP5**	2,7495E-10	0,36777812	7,3953E-06	3
**TET2**	2,7872E-10	0,44284509	7,4966E-06	3
**DOCK8**	2,8984E-10	0,15608729	7,796E-06	3
**TRBV27**	2,9151E-10	0,5447577	7,8409E-06	3
**WDR19**	3,1325E-10	0,65421964	8,4255E-06	3
**ZNF593**	3,1651E-10	0,30052914	8,5131E-06	3
**MTR**	3,2032E-10	0,26898283	8,6157E-06	3
**SLC12A7**	3,3335E-10	0,53185385	8,966E-06	3
**KDM6A**	3,452E-10	0,22555625	9,2849E-06	3
**PLXNC1**	3,4522E-10	0,42567011	9,2853E-06	3
**COPG1**	3,5252E-10	0,24435474	9,4818E-06	3
**ZBTB10**	3,5303E-10	0,40139727	9,4956E-06	3
**NOLC1**	3,6674E-10	0,21361868	9,8642E-06	3
**RP11-386G11.10**	3,7642E-10	0,61731454	1,0125E-05	3
**DNAJC3**	3,7991E-10	0,18276258	1,0219E-05	3
**IL11RA**	3,9372E-10	0,47734581	1,059E-05	3
**EZR**	4,0362E-10	0,14778575	1,0856E-05	3
**SLFN12L**	4,0884E-10	0,19343089	1,0997E-05	3
**DYNLL1**	4,3555E-10	0,14510024	1,1715E-05	3
**C4orf48**	4,6056E-10	0,15587092	1,2388E-05	3
**PIANP**	4,8901E-10	0,59110705	1,3153E-05	3
**KDM6B**	4,941E-10	0,31507915	1,329E-05	3
**CCSER2**	5,2448E-10	0,17697766	1,4107E-05	3
**PIK3CD**	5,2649E-10	0,21756943	1,4161E-05	3

**CERK**	5,4723E-10	0,1621986	1,4719E-05	3
**LTK**	5,6035E-10	0,15373195	1,5072E-05	3
**NUP153**	5,6258E-10	0,36520727	1,5132E-05	3
**NR3C2**	5,8623E-10	0,20449683	1,5768E-05	3
**LINC01420**	5,8808E-10	0,14862437	1,5818E-05	3
**EP300**	5,9849E-10	0,27656999	1,6098E-05	3
**CHORDC1**	6,5131E-10	0,22374826	1,7518E-05	3
**INADL**	7,0898E-10	0,25511228	1,9069E-05	3
**TMEM245**	7,3538E-10	0,36031382	1,978E-05	3
**KCTD15**	7,3658E-10	0,7821114	1,9812E-05	3
**AP3D1**	7,7509E-10	0,19707688	2,0848E-05	3
**NKTR**	7,9052E-10	0,14696768	2,1263E-05	3
**DMGDH**	8,2164E-10	0,59007353	2,21E-05	3
**ZMIZ1**	8,3445E-10	0,47723959	2,2444E-05	3
**RTN4**	8,539E-10	0,15883736	2,2967E-05	3
**KDM3A**	8,773E-10	0,30574452	2,3597E-05	3
**CDKN2B**	8,798E-10	1,49259492	2,3664E-05	3
**CD44**	8,8339E-10	0,1241847	2,3761E-05	3
**HIPK1**	8,8884E-10	0,2813949	2,3907E-05	3
**NBPF9**	9,1433E-10	0,78225728	2,4593E-05	3
**RP11-21J7.1**	9,4067E-10	1,25320098	2,5301E-05	3
**PUM1**	9,6817E-10	0,20162863	2,6041E-05	3
**CHN1**	1,0375E-09	0,75531065	2,7907E-05	3
**GPR155**	1,0409E-09	0,37457888	2,7996E-05	3
**SLAIN2**	1,0477E-09	0,22092573	2,8181E-05	3
**RABIF**	1,0572E-09	0,30677707	2,8435E-05	3
**SCO2**	1,0632E-09	0,3446779	2,8597E-05	3
**C16orf74**	1,0676E-09	0,32250916	2,8716E-05	3
**EEA1**	1,0988E-09	0,25278449	2,9555E-05	3
**HIVEP3**	1,1062E-09	0,2436401	2,9754E-05	3
**ANKRD12**	1,123E-09	0,12972697	3,0206E-05	3
**FBRS**	1,134E-09	0,25422219	3,0501E-05	3
**TNFSF10**	1,1363E-09	0,19102813	3,0564E-05	3
**JMY**	1,1415E-09	0,23812863	3,0702E-05	3
**AGFG1**	1,1709E-09	0,25491298	3,1494E-05	3
**NDFIP1**	1,181E-09	0,14073148	3,1765E-05	3
**SMARCC2**	1,2102E-09	0,1632848	3,255E-05	3
**EVA1C**	1,237E-09	0,28183305	3,3271E-05	3
**HIST1H1D**	1,3208E-09	0,14850548	3,5525E-05	3
**ADAM12**	1,3587E-09	0,5398727	3,6546E-05	3
**AIDA**	1,3629E-09	0,28135553	3,6659E-05	3

**UBL3**	1,4237E-09	0,16639824	3,8294E-05	3
**CAMK2N1**	1,4468E-09	0,2175134	3,8915E-05	3
**LDOC1**	1,4898E-09	0,33876837	4,0072E-05	3
**DIAPH2**	1,5498E-09	0,2882453	4,1686E-05	3
**KLF12**	1,5525E-09	0,12410632	4,1758E-05	3
**ZFC3H1**	1,6175E-09	0,1449481	4,3507E-05	3
**UGP2**	1,739E-09	0,1646251	4,6775E-05	3
**TRAM1**	1,7593E-09	0,14795858	4,7319E-05	3
**PPARD**	1,8005E-09	0,37776498	4,8427E-05	3
**PRDX3**	1,8046E-09	0,14620224	4,8539E-05	3
**APPL1**	1,8125E-09	0,15456576	4,8752E-05	3
**SCAMP1**	1,8141E-09	0,22351598	4,8794E-05	3
**IQGAP1**	1,8447E-09	0,11746309	4,9617E-05	3
**SKI**	1,8874E-09	0,50615116	5,0766E-05	3
**RP11-360F5.1**	1,9035E-09	1,19814674	5,1199E-05	3
**A2M**	1,9254E-09	0,38494771	5,1786E-05	3
**GOLGA7**	1,9473E-09	0,13711249	5,2377E-05	3
**MACF1**	1,9683E-09	0,16563541	5,2943E-05	3
**RAD51B**	1,9773E-09	0,28038989	5,3184E-05	3
**CYSTM1**	2,005E-09	0,36711008	5,3929E-05	3
**LRRC8C**	2,0372E-09	0,23714092	5,4795E-05	3
**IFNGR2**	2,0711E-09	0,4681221	5,5706E-05	3
**DDT**	2,1469E-09	0,13804596	5,7746E-05	3
**NIP7**	2,1642E-09	0,24113576	5,8211E-05	3
**TMEM30A**	2,1975E-09	0,18709941	5,9106E-05	3
**VMA21**	2,2641E-09	0,18569956	6,0899E-05	3
**GALNT7**	2,2721E-09	0,41011386	6,1113E-05	3
**PSEN1**	2,3362E-09	0,41784747	6,2837E-05	3
**CSPP1**	2,5519E-09	0,388971	6,8637E-05	3
**TTC39C**	2,578E-09	0,13444558	6,9341E-05	3
**SFMBT1**	2,6619E-09	0,30467639	7,1597E-05	3
**JAML**	2,7432E-09	0,17673651	7,3783E-05	3
**BPTF**	2,7515E-09	0,14875788	7,4006E-05	3
**ZNF148**	2,7655E-09	0,20379728	7,4383E-05	3
**RP11-326C3.2**	2,7708E-09	0,40697419	7,4527E-05	3
**FAM169A**	2,774E-09	0,56891593	7,4614E-05	3
**KLF11**	2,8302E-09	0,75060905	7,6124E-05	3
**UTRN**	2,8457E-09	0,13985998	7,6542E-05	3
**PDE8A**	2,8829E-09	0,28666192	7,7541E-05	3
**CYB561**	2,9218E-09	0,19494685	7,8587E-05	3
**RAB6A**	2,9755E-09	0,20238526	8,0033E-05	3

**KLHL28**	2,9834E-09	0,20402484	8,0243E-05	3
**ARHGEF7**	3,0094E-09	0,25790182	8,0944E-05	3
**GART**	3,038E-09	0,28514439	8,1713E-05	3
**UBIAD1**	3,0698E-09	0,23751137	8,2569E-05	3
**FLJ31356**	3,1047E-09	0,46874309	8,3507E-05	3
**PTBP3**	3,565E-09	0,1700266	9,5888E-05	3
**CDC42**	3,5805E-09	0,11490739	9,6304E-05	3
**SLC25A38**	3,6503E-09	0,17325561	9,8182E-05	3
**INO80D**	3,7086E-09	0,27915366	9,9749E-05	3
**JAK2**	3,8171E-09	0,30487985	0,00010267	3
**SLC7A1**	3,889E-09	0,40435954	0,0001046	3
**CLTC**	3,9042E-09	0,20353396	0,00010501	3
**RBFA**	4,0066E-09	0,29288852	0,00010777	3
**GPR150**	4,2209E-09	0,4049388	0,00011353	3
**FAM177A1**	4,3175E-09	0,11199822	0,00011613	3
**FAM105A**	4,3899E-09	0,16668991	0,00011807	3
**ARCN1**	4,5091E-09	0,20600078	0,00012128	3
**EIF2S1**	4,6418E-09	0,18036693	0,00012485	3
**RECQL**	4,6477E-09	0,13740448	0,00012501	3
**PRDM2**	4,6525E-09	0,15394521	0,00012514	3
**ID2**	4,6679E-09	0,22900852	0,00012555	3
**MTHFD2**	4,7588E-09	0,28711175	0,000128	3
**SIK3**	4,7688E-09	0,15833848	0,00012827	3
**PPP3R1**	4,8705E-09	0,18874141	0,000131	3
**ANP32E**	5,1005E-09	0,12728985	0,00013719	3
**PRDX4**	5,3126E-09	0,20046608	0,00014289	3
**ITGA6**	5,3828E-09	0,17702621	0,00014478	3
**TRIM35**	5,4279E-09	0,4562853	0,00014599	3
**NBL1**	5,8344E-09	0,41284099	0,00015693	3
**HIPK2**	5,9109E-09	0,26623438	0,00015899	3
**NOP14**	6,0152E-09	0,26002532	0,00016179	3
**RP11-570L15.2**	6,0636E-09	0,79550305	0,00016309	3
**MICU3**	6,1883E-09	0,61543622	0,00016645	3
**FOXJ3**	6,3695E-09	0,16869782	0,00017132	3
**SBF2**	6,4988E-09	0,29047881	0,0001748	3
**SENP6**	6,5444E-09	0,14943149	0,00017602	3
**IMMT**	6,6716E-09	0,20233449	0,00017944	3
**KLF13**	6,8381E-09	0,13294455	0,00018393	3
**SREK1**	6,9278E-09	0,20769627	0,00018634	3
**YWHAE**	6,9659E-09	0,17755278	0,00018736	3
**TRAT1**	7,0186E-09	0,15130601	0,00018878	3

**RNF19A**	7,1359E-09	0,152524	0,00019194	3
**TCERG1**	7,3218E-09	0,16845642	0,00019693	3
**NFATC3**	7,3331E-09	0,13419313	0,00019724	3
**WWC2**	7,4577E-09	0,51175654	0,00020059	3
**FOXK1**	7,6209E-09	0,25951638	0,00020498	3
**COPA**	7,7036E-09	0,19864819	0,0002072	3
**STAG2**	7,8939E-09	0,12949306	0,00021232	3
**SLC35G1**	7,9865E-09	0,71184183	0,00021481	3
**XPC**	8,3042E-09	0,21865168	0,00022336	3
**MAST4**	8,3836E-09	0,50336238	0,00022549	3
**JAK3**	9,0238E-09	0,18863391	0,00024271	3
**SEMA6C**	9,054E-09	0,94857371	0,00024352	3
**CS**	9,1993E-09	0,21625749	0,00024743	3
**H2AFZ**	9,3128E-09	0,11692817	0,00025049	3
**ERN1**	9,3654E-09	0,13470587	0,0002519	3
**PRPF8**	9,3814E-09	0,12166883	0,00025233	3
**SEC24B**	9,5989E-09	0,19466264	0,00025818	3
**CFAP97**	9,9428E-09	0,1327047	0,00026743	3
**SCFD2**	1,0088E-08	0,24496379	0,00027135	3
**RP11-439L18.2**	1,0099E-08	0,34952037	0,00027165	3
**BLVRA**	1,0186E-08	0,20717402	0,00027397	3
**CHD7**	1,041E-08	0,24429258	0,00027999	3
**RP11-123O10.4**	1,0604E-08	0,92112679	0,00028523	3
**HAGH**	1,081E-08	0,16324147	0,00029076	3
**PPP6R3**	1,0967E-08	0,17899653	0,00029498	3
**HIPK3**	1,1118E-08	0,2578193	0,00029903	3
**FNDC3A**	1,1233E-08	0,19563825	0,00030213	3
**LINC01492**	1,1269E-08	0,1935294	0,00030311	3
**PCYOX1**	1,1384E-08	0,3793526	0,0003062	3
**RAB3GAP1**	1,1396E-08	0,23427937	0,00030652	3
**NEO1**	1,1602E-08	0,44223501	0,00031207	3
**MAPK14**	1,1957E-08	0,27956539	0,0003216	3
**AC107218.3**	1,247E-08	1,08815244	0,00033541	3
**SEC23A**	1,2518E-08	0,29770534	0,0003367	3
**OSBPL3**	1,2559E-08	0,29318994	0,0003378	3
**NFKB2**	1,2767E-08	0,27111134	0,00034341	3
**ABCC1**	1,2813E-08	0,27602649	0,00034464	3
**PFKL**	1,2896E-08	0,16429814	0,00034687	3
**PRRC2A**	1,2943E-08	0,18385511	0,00034813	3
**IQCK**	1,3551E-08	1,02917116	0,00036449	3
**MYH9**	1,3725E-08	0,12855874	0,00036917	3

**ARNTL**	1,374E-08	0,18833901	0,00036956	3
**GPR183**	1,3887E-08	0,14785146	0,00037353	3
**SLC4A7**	1,4676E-08	0,18321033	0,00039473	3
**ANTXR2**	1,4731E-08	0,22748214	0,00039621	3
**CREBL2**	1,5114E-08	0,20806954	0,00040651	3
**CLN5**	1,5124E-08	0,30208743	0,00040679	3
**RELB**	1,5602E-08	0,1531295	0,00041964	3
**ARIH1**	1,5886E-08	0,18917476	0,00042728	3
**SPG20**	1,6266E-08	0,19370463	0,0004375	3
**EPB41**	1,7285E-08	0,11554329	0,0004649	3
**ABCD2**	1,7406E-08	0,37376856	0,00046816	3
**HPRT1**	1,7492E-08	0,15827143	0,00047049	3
**CCNC**	1,7621E-08	0,14313076	0,00047395	3
**BBS9**	1,7704E-08	0,320246	0,0004762	3
**RIF1**	1,8551E-08	0,22482528	0,00049897	3
**CTB-66B24.1**	1,8857E-08	1,52133202	0,00050719	3
**PMM2**	1,9459E-08	0,21594706	0,00052339	3
**AREG**	2,0305E-08	0,27796254	0,00054614	3
**TBC1D14**	2,0445E-08	0,21464221	0,0005499	3
**RNF145**	2,1052E-08	0,14091714	0,00056625	3
**CELF1**	2,108E-08	0,21883743	0,00056699	3
**TRIM33**	2,1585E-08	0,19737185	0,00058058	3
**CH507-528H12.1**	2,2322E-08	0,19940066	0,00060039	3
**EIF3J**	2,2517E-08	0,19883741	0,00060564	3
**TSPAN18**	2,3176E-08	0,2549757	0,00062336	3
**TMEM181**	2,3276E-08	0,25724281	0,00062606	3
**ATF6**	2,5745E-08	0,24390177	0,00069247	3
**TLN1**	2,5801E-08	0,11356728	0,00069397	3
**ANKLE2**	2,5825E-08	0,18980642	0,00069462	3
**TRIM46**	2,6069E-08	0,57764926	0,00070118	3
**ANKRD28**	2,7049E-08	0,28476843	0,00072753	3
**VPS13B**	2,7364E-08	0,18855168	0,00073601	3
**VIM-AS1**	2,8785E-08	0,18298151	0,00077422	3
**OSBPL8**	2,9018E-08	0,11594796	0,00078048	3
**TBC1D22A**	2,906E-08	0,22714255	0,00078164	3
**IFFO2**	2,9248E-08	0,27858544	0,00078669	3
**GNA13**	2,9541E-08	0,14011455	0,00079456	3
**RP11-703M24.5**	3,0057E-08	0,2116776	0,00080846	3
**FAM3C**	3,0191E-08	0,26519216	0,00081203	3
**ELMO1**	3,0612E-08	0,15313787	0,00082337	3
**GPBP1**	3,0854E-08	0,11994261	0,00082987	3

**TMEM163**	3,1326E-08	0,83374939	0,00084258	3
**IFI44L**	3,1479E-08	0,22477734	0,0008467	3
**FRYL**	3,1513E-08	0,15104668	0,00084761	3
**CRTC3**	3,1735E-08	0,24884217	0,00085356	3
**RP11-126O1.4**	3,1822E-08	0,52262431	0,00085591	3
**RPL7L1**	3,2041E-08	0,21955023	0,00086182	3
**RRM1**	3,2173E-08	0,30530005	0,00086536	3
**SEC22C**	3,2317E-08	0,20367372	0,00086922	3
**KPNA1**	3,2651E-08	0,22258942	0,00087821	3
**ASH1L**	3,3011E-08	0,13337576	0,00088789	3
**MARCH9**	3,3344E-08	0,14466627	0,00089686	3
**MVP**	3,3685E-08	0,12704548	0,00090604	3
**CD151**	3,382E-08	0,24317005	0,00090967	3
**ZNF292**	3,4132E-08	0,15320501	0,00091806	3
**RP13-726E6.2**	3,4214E-08	1,06858038	0,00092027	3
**ZNF398**	3,4297E-08	0,42965188	0,00092248	3
**PLD6**	3,4651E-08	0,4255704	0,00093202	3
**IFRD1**	3,501E-08	0,16440917	0,00094166	3
**TMOD3**	3,5178E-08	0,1467008	0,00094618	3
**MFSD14B**	3,7809E-08	0,28670811	0,00101696	3
**REEP5**	3,8129E-08	0,11472013	0,00102554	3
**DOPEY2**	3,8551E-08	0,33275385	0,00103692	3
**AGTPBP1**	3,8902E-08	0,21831779	0,00104635	3
**TPGS1**	4,1659E-08	0,12756919	0,0011205	3
**B4GALT5**	4,2726E-08	0,35232348	0,00114921	3
**GTF2H1**	4,2742E-08	0,18584518	0,00114964	3
**SPCS3**	4,3044E-08	0,11280881	0,00115775	3
**FAM122C**	4,3451E-08	0,45855027	0,00116871	3
**IRF2BP2**	4,367E-08	0,17366785	0,00117459	3
**AAED1**	4,4684E-08	0,2376149	0,00120187	3
**GDPD5**	4,4833E-08	0,33757195	0,00120587	3
**MAPK6**	4,5045E-08	0,30783676	0,00121156	3
**INSIG2**	4,5257E-08	0,28399054	0,00121727	3
**PDE4DIP**	4,6989E-08	0,26839227	0,00126386	3
**NUP98**	4,7047E-08	0,21546425	0,00126541	3
**RP1-159A19.4**	4,7613E-08	0,80781416	0,00128066	3
**ICMT**	4,8954E-08	0,41789795	0,00131671	3
**SPAG9**	4,9685E-08	0,2101112	0,00133637	3
**RP11-195F19.5**	5,008E-08	0,58154031	0,001347	3
**ZDHHC2**	5,0424E-08	0,20677441	0,00135626	3
**TNFRSF25**	5,4661E-08	0,12703317	0,00147021	3

**RPTOR**	5,5872E-08	0,31275875	0,0015028	3
**BEGAIN**	5,6298E-08	1,004774	0,00151425	3
**TTC19**	5,6752E-08	0,16643548	0,00152646	3
**LINS1**	5,7241E-08	0,20870521	0,00153961	3
**CASC4**	5,7271E-08	0,16350055	0,00154041	3
**RBM6**	5,7301E-08	0,15403171	0,00154123	3
**TFRC**	5,7756E-08	0,35206436	0,00155345	3
**NR1D1**	5,7867E-08	0,22450316	0,00155644	3
**PNPT1**	5,7977E-08	0,25112544	0,0015594	3
**LATS1**	5,8224E-08	0,20690254	0,00156605	3
**CBLL1**	5,8795E-08	0,15624862	0,0015814	3
**AEBP2**	5,883E-08	0,17888686	0,00158236	3
**PHF3**	5,9376E-08	0,10536138	0,00159703	3
**NFE2L2**	5,9792E-08	0,13667937	0,00160823	3
**CCDC93**	6,0106E-08	0,26262708	0,00161668	3
**CFL2**	6,0248E-08	0,33805924	0,00162049	3
**ASCC3**	6,1044E-08	0,16341289	0,0016419	3
**PZP**	6,2191E-08	0,25430596	0,00167274	3
**CD28**	6,2799E-08	0,12108916	0,0016891	3
**PPIL4**	6,2862E-08	0,19475224	0,0016908	3
**SLC30A7**	6,3317E-08	0,2718109	0,00170302	3
**COG5**	6,3939E-08	0,17633916	0,00171977	3
**FNBP4**	6,4131E-08	0,10394511	0,00172493	3
**DLEU1**	6,6983E-08	0,20176248	0,00180164	3
**PURA**	6,7228E-08	0,14745239	0,00180823	3
**GMPS**	6,9459E-08	0,16765454	0,00186823	3
**TMED10**	7,0108E-08	0,10906072	0,00188569	3
**UST**	7,0817E-08	0,26915169	0,00190476	3
**HRAS**	7,1097E-08	0,18664638	0,0019123	3
**PIK3R5**	7,1438E-08	0,14035704	0,00192147	3
**PRKCSH**	7,311E-08	0,14554653	0,00196643	3
**ARL14EP**	7,6492E-08	0,14416021	0,00205742	3
**CELF2**	7,9122E-08	0,10960643	0,00212814	3
**RP5-857K21.4**	7,9603E-08	0,11457946	0,00214109	3
**MFHAS1**	7,9876E-08	0,16215686	0,00214842	3
**UBE3A**	8,0225E-08	0,12833392	0,00215781	3
**FANCE**	8,0328E-08	0,46482838	0,00216058	3
**MAP4K5**	8,219E-08	0,20949499	0,00221067	3
**DALRD3**	8,2237E-08	0,14486965	0,00221193	3
**ARL2BP**	8,3162E-08	0,10187967	0,00223681	3
**CCT6A**	8,3816E-08	0,12403963	0,00225439	3

**OXA1L**	8,4058E-08	0,1221349	0,00226092	3
**RNF168**	8,4275E-08	0,15867962	0,00226675	3
**PRKCA-AS1**	8,5746E-08	0,87734356	0,00230631	3
**PRPF6**	8,6799E-08	0,12352878	0,00233463	3
**LONRF1**	8,7299E-08	0,58515422	0,00234808	3
**HSPD1**	8,9687E-08	0,11247742	0,0024123	3
**NAMPT**	9,0616E-08	0,28468086	0,00243729	3
**BRAF**	9,0714E-08	0,21221079	0,00243994	3
**FGD5-AS1**	9,1875E-08	0,17172123	0,00247115	3
**CXorf38**	9,6026E-08	0,21014722	0,00258282	3
**HIST1H1C**	9,7548E-08	0,11216182	0,00262375	3
**RUNX3**	9,8597E-08	0,11724404	0,00265197	3
**UHMK1**	1,0556E-07	0,13427108	0,00283925	3
**NR4A2**	1,084E-07	0,13725708	0,00291575	3
**GALNT10**	1,0999E-07	0,19343251	0,00295827	3
**ECE1**	1,113E-07	0,23134711	0,00299355	3
**HEATR1**	1,128E-07	0,29566282	0,00303392	3
**PARK2**	1,1488E-07	0,35591332	0,00308985	3
**ZNRF1**	1,1515E-07	0,34099512	0,00309706	3
**TRIM37**	1,1591E-07	0,48466343	0,00311755	3
**TMBIM1**	1,1595E-07	0,13047213	0,00311879	3
**ICK**	1,1615E-07	0,42729074	0,00312399	3
**WDR48**	1,1696E-07	0,21789747	0,00314579	3
**MRPS27**	1,1955E-07	0,20533772	0,00321552	3
**MRPS36**	1,1966E-07	0,12951111	0,00321852	3
**FARS2**	1,1984E-07	0,20139583	0,00322339	3
**PLEKHB2**	1,2369E-07	0,19650028	0,00332688	3
**PIGX**	1,2581E-07	0,2814871	0,00338393	3
**FUT8**	1,2602E-07	0,22899791	0,00338954	3
**HSPA9**	1,275E-07	0,14990922	0,00342926	3
**BTBD9**	1,2972E-07	0,20275469	0,00348915	3
**EPS8**	1,306E-07	0,35922994	0,00351286	3
**SMG1**	1,3123E-07	0,11738228	0,00352979	3
**MTFP1**	1,3547E-07	0,15488995	0,00364386	3
**MAGT1**	1,3904E-07	0,21304892	0,00373987	3
**ADAM17**	1,3966E-07	0,25049675	0,00375643	3
**RP11-700H6.1**	1,4006E-07	1,30916964	0,00376728	3
**DOCK9**	1,4126E-07	0,17092745	0,00379936	3
**MKNK2**	1,4158E-07	0,12740606	0,00380816	3
**UMAD1**	1,4403E-07	0,16421869	0,00387392	3
**SNAI3**	1,4457E-07	0,22948316	0,00388859	3

**SRSF4**	1,4659E-07	0,12704916	0,00394292	3
**OXR1**	1,4726E-07	0,17777717	0,00396089	3
**TANC2**	1,4804E-07	0,35580449	0,00398188	3
**EPB41L4A**	1,5139E-07	1,1736465	0,00407203	3
**FAAH2**	1,5702E-07	0,18840803	0,00422336	3
**VPS35**	1,5993E-07	0,13898145	0,00430166	3
**NSMCE1**	1,6119E-07	0,14950638	0,00433553	3
**RALGAPB**	1,615E-07	0,21106473	0,00434395	3
**DYM**	1,6188E-07	0,17908264	0,00435404	3
**NRROS**	1,6533E-07	0,55854708	0,00444676	3
**C10orf128**	1,6911E-07	0,28936796	0,00454849	3
**ZXDC**	1,7013E-07	0,209888	0,00457601	3
**MMS19**	1,7067E-07	0,20909197	0,00459047	3
**MSI2**	1,71E-07	0,21739611	0,00459946	3
**FAM120A**	1,7242E-07	0,17440726	0,00463749	3
**GNB1**	1,7451E-07	0,11919743	0,00469392	3
**PDE5A**	1,7527E-07	0,70984657	0,00471421	3
**NIPBL**	1,7561E-07	0,11511019	0,00472329	3
**CAMKMT**	1,7679E-07	0,21750361	0,004755	3
**ZBTB44**	1,7996E-07	0,19823546	0,00484032	3
**FNBP1**	1,807E-07	0,10844396	0,00486034	3
**RPS17**	1,8238E-07	0,10268688	0,00490547	3
**BAZ1B**	1,8279E-07	0,18118764	0,00491655	3
**LRPPRC**	1,9147E-07	0,10257833	0,00514986	3
**NAPG**	1,9726E-07	0,1755873	0,00530576	3
**PPM1B**	1,9752E-07	0,16858912	0,00531271	3
**RNH1**	1,9885E-07	0,14719096	0,00534841	3
**ZC4H2**	1,9885E-07	0,37658862	0,0053486	3
**RNF125**	1,9918E-07	0,1109462	0,00535741	3
**SSR3**	2,0328E-07	0,13960628	0,0054676	3
**ANO6**	2,0604E-07	0,21019357	0,00554175	3
**AMPD2**	2,0982E-07	0,2474389	0,00564361	3
**MANEA**	2,0993E-07	0,41188634	0,00564648	3
**ZEB1**	2,1001E-07	0,10404237	0,0056487	3
**KLF16**	2,1231E-07	0,37208812	0,00571045	3
**CDC42SE1**	2,1847E-07	0,11635279	0,00587631	3
**IKBKAP**	2,1918E-07	0,34448007	0,00589537	3
**FAM206A**	2,2194E-07	0,25522298	0,00596962	3
**DHX9**	2,2198E-07	0,14554837	0,00597056	3
**STT3A**	2,2489E-07	0,12473292	0,0060488	3
**ZNF664**	2,3049E-07	0,234936	0,00619943	3

**S1PR4**	2,3457E-07	0,12010542	0,00630936	3
**CTLA4**	2,3541E-07	0,50534888	0,00633172	3
**BLMH**	2,3721E-07	0,24642891	0,00638027	3
**C21orf91**	2,4E-07	0,15167752	0,00645522	3
**SAFB2**	2,4012E-07	0,11380734	0,00645839	3
**PGM2L1**	2,4215E-07	0,40787073	0,00651312	3
**PDCD6IP**	2,4444E-07	0,10805286	0,00657482	3
**FAM153A**	2,4623E-07	0,54912327	0,00662276	3
**B3GNT2**	2,4771E-07	0,22834385	0,00666257	3
**CDK8**	2,5E-07	0,26945647	0,00672417	3
**SPTY2D1**	2,5186E-07	0,2336418	0,00677433	3
**CORO2A**	2,5427E-07	0,35095873	0,00683903	3
**RP4-575N6.4**	2,5905E-07	0,5095765	0,00696776	3
**RICTOR**	2,6374E-07	0,13418689	0,00709368	3
**FDFT1**	2,7322E-07	0,1135879	0,00734876	3
**HERC1**	2,7437E-07	0,1562752	0,00737978	3
**TPM4**	2,7483E-07	0,10572569	0,00739212	3
**MORF4L2**	2,8469E-07	0,11734729	0,00765725	3
**DIMT1**	2,8784E-07	0,15819588	0,00774202	3
**BTBD11**	2,9027E-07	0,21825773	0,00780734	3
**ZMYM1**	2,9083E-07	0,31014844	0,00782235	3
**PPP1R14B**	2,9123E-07	0,12327173	0,00783333	3
**ERC1**	2,925E-07	0,21110475	0,00786744	3
**MMD**	2,9416E-07	0,37089973	0,00791209	3
**MOSPD3**	2,946E-07	0,16177706	0,00792387	3
**CREBBP**	2,9689E-07	0,19976714	0,00798548	3
**LINC00936**	3,0656E-07	0,20300215	0,00824552	3
**CCDC91**	3,094E-07	0,12471018	0,00832194	3
**ARRDC2**	3,1073E-07	0,15881787	0,00835777	3
**PTPN11**	3,1414E-07	0,17172612	0,00844942	3
**AXIN2**	3,2348E-07	0,4840282	0,00870057	3
**POLR1E**	3,2533E-07	0,21570126	0,00875048	3
**RUFY1**	3,261E-07	0,25081593	0,00877119	3
**PRNP**	3,288E-07	0,1068966	0,00884369	3
**PCF11**	3,2938E-07	0,13287088	0,00885945	3
**GMDS**	3,3924E-07	0,16253631	0,00912441	3
**TPP2**	3,4048E-07	0,11586351	0,00915802	3
**RP11-462L8.1**	3,4157E-07	0,86399132	0,0091873	3
**OSTC**	3,4372E-07	0,1072211	0,00924504	3
**FXYD1**	3,5206E-07	0,33720806	0,00946942	3
**RFWD2**	3,5376E-07	0,15278933	0,00951495	3

**ARL6IP6**	3,5818E-07	0,12605554	0,00963408	3
**ARFGAP3**	3,5909E-07	0,11643405	0,00965836	3
**TRIM2**	3,6136E-07	0,85467424	0,00971957	3
**POGK**	3,6242E-07	0,25168375	0,00974808	3
**FNIP2**	3,6291E-07	0,71515713	0,00976122	3
**SLC35C1**	3,6491E-07	0,41164112	0,00981489	3
**RAB18**	3,6503E-07	0,10474637	0,0098183	3
**SLC7A5**	3,7508E-07	0,31172405	0,01008863	3
**CAPNS1**	3,8047E-07	0,1254371	0,01023353	3
**ZDHHC20**	3,8082E-07	0,18113221	0,0102428	3
**CD82**	3,8727E-07	0,10771317	0,01041629	3
**POGZ**	4,0343E-07	0,18622449	0,01085098	3
**MTMR6**	4,0448E-07	0,26396719	0,01087932	3
**MYO9A**	4,0611E-07	0,25758675	0,01092303	3
**LINC00657**	4,115E-07	0,11995552	0,01106819	3
**CHRAC1**	4,1215E-07	0,16305551	0,01108554	3
**AREL1**	4,2117E-07	0,39972036	0,01132834	3
**CBFB**	4,2131E-07	0,2399177	0,01133198	3
**SMAD2**	4,2242E-07	0,14852493	0,0113617	3
**PITPNA**	4,326E-07	0,33140579	0,01163562	3
**TFEB**	4,3784E-07	0,28889326	0,01177653	3
**MAP4K2**	4,4308E-07	0,12800864	0,01191754	3
**STK17B**	4,5449E-07	0,10304825	0,01222443	3
**UBE2A**	4,6428E-07	0,16025961	0,01248767	3
**LNPEP**	4,6581E-07	0,11733806	0,01252882	3
**FAM76A**	4,7681E-07	0,29765445	0,01282483	3
**RP11-138A9.2**	4,8289E-07	0,54786827	0,01298832	3
**RAD23B**	4,8955E-07	0,13261951	0,01316751	3
**LTC4S**	4,947E-07	0,30506155	0,01330608	3
**DHX32**	5,1506E-07	0,46153235	0,01385368	3
**NDRG1**	5,1619E-07	0,20348296	0,0138839	3
**ZFX**	5,1726E-07	0,18258648	0,0139127	3
**DUSP5**	5,1771E-07	0,11668267	0,01392488	3
**GLRX**	5,2186E-07	0,10694812	0,01403655	3
**TADA2A**	5,2281E-07	0,48229898	0,01406205	3
**PHF21A**	5,236E-07	0,17371614	0,0140833	3
**WASF2**	5,2438E-07	0,10264967	0,01410414	3
**ZZEF1**	5,2641E-07	0,20279675	0,01415896	3
**CBR3**	5,3006E-07	0,32136559	0,01425708	3
**CLIC4**	5,3119E-07	1,01268396	0,01428747	3
**PITPNB**	5,4182E-07	0,16750394	0,01457337	3

**KLHL15**	5,4881E-07	0,43921838	0,01476128	3
**GOLPH3**	5,5576E-07	0,11848627	0,01494837	3
**ADCK3**	5,5842E-07	0,24313342	0,01501972	3
**BTF3L4**	5,596E-07	0,11510424	0,01505148	3
**PPIL6**	5,6286E-07	1,30509538	0,01513937	3
**KLHDC10**	5,7512E-07	0,27179482	0,01546891	3
**ACOT7**	5,785E-07	0,49261933	0,01555998	3
**KDM2A**	5,8068E-07	0,12436096	0,01561868	3
**PICALM**	5,8517E-07	0,16419302	0,01573941	3
**CDK12**	5,8524E-07	0,16072956	0,01574112	3
**TYW3**	5,8582E-07	0,22592502	0,01575693	3
**BCAS3**	5,8593E-07	0,18731393	0,0157597	3
**CTB-133G6.1**	5,999E-07	0,13666009	0,01613549	3
**ARHGEF40**	6,1338E-07	1,1261718	0,01649816	3
**SIRT1**	6,1408E-07	0,27313873	0,01651698	3
**DCAF11**	6,1601E-07	0,14494659	0,01656893	3
**NR4A1**	6,2454E-07	0,71515981	0,01679814	3
**HSPA4**	6,2538E-07	0,21046093	0,01682097	3
**ICE1**	6,3019E-07	0,12918611	0,01695034	3
**TXNDC11**	6,4354E-07	0,15725387	0,01730934	3
**TMEM57**	6,4439E-07	0,17844736	0,01733221	3
**NT5DC1**	6,6184E-07	0,10618272	0,01780142	3
**SEC14L2**	6,6185E-07	0,71111425	0,01780177	3
**BTBD6**	6,7386E-07	0,12692165	0,01812487	3
**IFNAR1**	6,7666E-07	0,14779932	0,01820003	3
**HN1L**	6,8034E-07	0,39805148	0,01829921	3
**LRP10**	6,9038E-07	0,10637229	0,01856905	3
**NFX1**	6,9889E-07	0,17550463	0,01879802	3
**CCT5**	7,0101E-07	0,12293174	0,01885507	3
**SLC35E2**	7,0487E-07	0,3457371	0,01895893	3
**KNOP1**	7,055E-07	0,29132787	0,01897587	3
**UBQLN1**	7,1568E-07	0,1278581	0,01924973	3
**RRAGC**	7,4301E-07	0,18048491	0,01998486	3
**IP6K2**	7,4506E-07	0,12500633	0,02003989	3
**PHC3**	7,5579E-07	0,12839094	0,02032842	3
**CEP57**	7,6507E-07	0,13029646	0,02057821	3
**SETD5**	7,7612E-07	0,1610612	0,02087532	3
**SMYD3**	7,8278E-07	0,14231132	0,02105443	3
**RRS1**	7,8773E-07	0,21653652	0,02118769	3
**TSPYL1**	7,9126E-07	0,18997664	0,02128249	3
**DPYD-AS1**	7,9173E-07	0,51033121	0,02129504	3

**HIBADH**	8,0708E-07	0,17171712	0,02170813	3
**KMT5A**	8,1502E-07	0,20757985	0,02192152	3
**AP3B1**	8,341E-07	0,15659308	0,0224347	3
**ASTE1**	8,4209E-07	0,21893441	0,02264982	3
**ATXN2L**	8,4373E-07	0,10306717	0,02269374	3
**KIAA1328**	8,4745E-07	0,23987614	0,02279375	3
**VEZT**	8,6347E-07	0,17730928	0,02322462	3
**EFHC2**	8,6519E-07	0,65131166	0,02327101	3
**SOD2**	8,8847E-07	0,10530181	0,02389711	3
**RB1CC1**	8,903E-07	0,15452707	0,02394635	3
**GPR132**	8,9407E-07	0,24656828	0,02404785	3
**MAPKAPK5**	9,0487E-07	0,14852539	0,02433819	3
**NUS1**	9,1977E-07	0,17735272	0,02473904	3
**AC026471.6**	9,1987E-07	0,69437547	0,02474171	3
**LUZP1**	9,2261E-07	0,1753424	0,02481553	3
**HSPH1**	9,2726E-07	0,17127575	0,02494045	3
**SMC5**	9,3682E-07	0,16208341	0,02519761	3
**FAF1**	9,4381E-07	0,11528553	0,02538576	3
**SCAF4**	9,52E-07	0,13147093	0,02560586	3
**TFDP1**	9,5424E-07	0,14753168	0,0256661	3
**VDAC1**	1,0014E-06	0,10522625	0,02693496	3
**ATG10**	1,0029E-06	0,34221523	0,02697602	3
**RRP1B**	1,0278E-06	0,15366591	0,02764504	3
**ANKRD13A**	1,0341E-06	0,19059127	0,02781466	3
**PRKAG2-AS1**	1,0513E-06	0,1777337	0,02827588	3
**HECTD4**	1,0653E-06	0,19609212	0,02865394	3
**C2orf81**	1,0668E-06	0,32570259	0,02869423	3
**ZNF44**	1,0867E-06	0,22849971	0,02922883	3
**WAC**	1,1007E-06	0,12210319	0,0296055	3
**PSMD6-AS2**	1,1114E-06	0,52396745	0,02989351	3
**RASSF7**	1,1227E-06	0,12822028	0,03019787	3
**A1BG**	1,1385E-06	0,10659476	0,03062225	3
**MRPL40**	1,139E-06	0,10418216	0,030635	3
**RHEBL1**	1,1468E-06	0,31671053	0,03084479	3
**DUSP8**	1,1509E-06	0,53148307	0,03095446	3
**MDN1**	1,1662E-06	0,17381394	0,03136703	3
**CRKL**	1,1667E-06	0,23414564	0,03138171	3
**SLC25A29**	1,167E-06	0,26881216	0,03138909	3
**SYT11**	1,1743E-06	0,36878027	0,03158385	3
**CTC-523E23.11**	1,1765E-06	0,13501337	0,03164324	3
**RAB10**	1,1934E-06	0,11263681	0,03209919	3

**LATS2**	1,2005E-06	0,67149875	0,03228877	3
**CRAMP1**	1,2175E-06	0,50708062	0,03274595	3
**BUD13**	1,2361E-06	0,30021447	0,03324682	3
**MAP3K3**	1,2571E-06	0,22711283	0,03381226	3
**RGS14**	1,263E-06	0,11952932	0,03397183	3
**B3GLCT**	1,2665E-06	0,26182114	0,03406409	3
**NR1D2**	1,289E-06	0,12390412	0,03467076	3
**DPM3**	1,2904E-06	0,13469964	0,03470856	3
**PAFAH1B1**	1,2956E-06	0,12906414	0,03484644	3
**PKD2**	1,2975E-06	0,54676841	0,03489776	3
**TOMM70A**	1,3046E-06	0,19140606	0,03509108	3
**NEK11**	1,3094E-06	0,664423	0,03521776	3
**C16orf72**	1,315E-06	0,18972166	0,03537072	3
**HDAC4**	1,3197E-06	0,21997356	0,03549479	3
**CRLF3**	1,3421E-06	0,11918381	0,0360985	3
**NOP56**	1,3579E-06	0,11950472	0,03652273	3
**CCDC7.1**	1,3604E-06	0,42456688	0,03659096	3
**SRPK1**	1,3604E-06	0,1684373	0,0365914	3
**TULP4**	1,4062E-06	0,17448283	0,0378234	3
**PCNT**	1,4076E-06	0,24244401	0,03786052	3
**CASP10**	1,4105E-06	0,38234835	0,0379373	3
**ZC3H11A**	1,4426E-06	0,10762047	0,03880028	3
**SLC38A2**	1,4491E-06	0,11940865	0,03897609	3
**SLC35E2B**	1,4577E-06	0,17246894	0,03920656	3
**ARHGAP10**	1,4667E-06	0,32952425	0,03944997	3
**GNL3**	1,4677E-06	0,16358514	0,0394774	3
**EGLN2**	1,473E-06	0,25801516	0,0396181	3
**USP36**	1,474E-06	0,14771614	0,03964685	3
**LRRC40**	1,4824E-06	0,24521154	0,03987295	3
**MIR22HG**	1,4827E-06	0,44197761	0,03987953	3
**EIF4G1**	1,4918E-06	0,13874222	0,04012403	3
**UHRF1BP1L**	1,5135E-06	0,2456892	0,04070757	3
**TLR2**	1,5165E-06	1,18241047	0,04078978	3
**ULK2**	1,5193E-06	0,58934532	0,04086388	3
**UFM1**	1,5212E-06	0,11488124	0,04091658	3
**CROT**	1,554E-06	0,2830814	0,04179772	3
**PTGDS**	1,5641E-06	0,50625593	0,04207068	3
**BACH1**	1,6104E-06	0,20023021	0,04331448	3
**G3BP2**	1,6274E-06	0,10393822	0,04377312	3
**TTLL4**	1,6308E-06	0,81676214	0,04386469	3
**PRRC1**	1,649E-06	0,16818405	0,04435283	3

**PTPN1**	1,6607E-06	0,12377527	0,04466802	3
**KIAA1033**	1,6678E-06	0,14871406	0,04485866	3
**LRRFIP2**	1,669E-06	0,14007216	0,04489243	3
**NINJ1**	1,6731E-06	0,13589807	0,04500252	3
**RP1-111C20.4**	1,6991E-06	0,24652853	0,04569939	3
**MRPS16**	1,7164E-06	0,14441503	0,04616497	3
**GTF3C4**	1,7311E-06	0,3642741	0,04656054	3
**ZNF714**	1,7585E-06	0,50111865	0,04729862	3
**HAUS4**	1,7655E-06	0,1096497	0,0474874	3
**ANGPTL6**	1,8028E-06	0,75913059	0,04848992	3
**AP1B1**	1,8275E-06	0,24369327	0,04915296	3
**OSBP**	1,8304E-06	0,20664699	0,04923203	3
**UCKL1**	1,8321E-06	0,16112871	0,04927716	3
**MGAT2**	1,8488E-06	0,23494991	0,04972597	3
**CCL5**	0	1,19808049	0	4
**CCR9**	0	3,84827532	0	4
**ITGA4**	6,249E-234	0,87607284	1,681E-229	4
**MALAT1**	2,669E-145	0,32293367	7,18E-141	4
**KLF6**	3,681E-139	0,64666435	9,901E-135	4
**LRRN3**	2,631E-121	2,29280286	7,076E-117	4
**IGFBP3**	3,119E-118	1,82277093	8,389E-114	4
**NT5E**	1,227E-99	1,73765329	3,3012E-95	4
**TXNIP**	1,0185E-92	0,3711709	2,7395E-88	4
**RHOB**	2,174E-81	1,78892129	5,8473E-77	4
**FTL**	2,8354E-71	0,22614018	7,6265E-67	4
**GLIPR1**	1,7832E-70	0,74306009	4,7962E-66	4
**KLRB1**	9,6857E-68	0,43235476	2,6052E-63	4
**ITM2B**	1,0081E-64	0,22936736	2,7114E-60	4
**ITGA1**	5,6821E-64	3,07158504	1,5283E-59	4
**LIMS1**	1,168E-63	0,61722345	3,1417E-59	4
**SESN1**	3,2177E-63	0,7378137	8,6545E-59	4
**TMIGD2**	5,0559E-63	1,14466188	1,3599E-58	4
**MYO16**	6,1669E-53	1,96585841	1,6587E-48	4
**TNFSF13B**	1,1488E-52	0,99215387	3,09E-48	4
**AC104820.2**	1,7819E-52	0,98879481	4,7929E-48	4
**SCML4**	1,2762E-50	0,63878516	3,4326E-46	4
**IL7R**	1,1577E-49	0,2134553	3,1139E-45	4
**LZTFL1**	3,1319E-47	1,43990952	8,424E-43	4
**CLIC3**	1,4838E-45	1,02654954	3,9909E-41	4
**SERINC5**	1,0317E-44	0,65772503	2,775E-40	4
**ZBTB20**	6,8523E-44	0,58241969	1,8431E-39	4

**CAMK4**	8,7404E-44	0,45915481	2,3509E-39	4
**TSC22D3**	1,0044E-43	0,28794736	2,7016E-39	4
**ADAM23**	2,3721E-43	1,17941923	6,3804E-39	4
**MGAT5**	8,1756E-42	0,72197525	2,199E-37	4
**PABPC1**	6,5384E-41	0,17066894	1,7586E-36	4
**AIF1**	2,8987E-39	1,22118392	7,7967E-35	4
**HIST1H1E**	6,991E-38	0,41532129	1,8804E-33	4
**TAGAP**	1,1321E-37	0,36207208	3,0451E-33	4
**DOCK10**	2,1372E-37	0,50203523	5,7484E-33	4
**LAG3**	2,1123E-35	1,02984972	5,6816E-31	4
**SMCHD1**	2,6476E-35	0,33373348	7,1211E-31	4
**TAB2**	3,0064E-34	0,53043499	8,0863E-30	4
**MT-ATP6**	3,5042E-34	0,1631482	9,4251E-30	4
**CD96**	1,9655E-33	0,37047479	5,2866E-29	4
**COTL1**	5,2261E-33	0,26598287	1,4057E-28	4
**RP11-1399P15.1**	5,5614E-33	1,04963038	1,4959E-28	4
**TC2N**	1,5302E-31	0,35066608	4,1159E-27	4
**KLF12**	2,251E-31	0,46542833	6,0544E-27	4
**GSTK1**	6,523E-31	0,22831252	1,7545E-26	4
**DIRC3**	7,7789E-31	1,7247953	2,0923E-26	4
**MIAT**	9,5009E-31	1,40009451	2,5554E-26	4
**FYB**	8,9687E-30	0,25249451	2,4123E-25	4
**RAB37**	9,2349E-30	0,92238264	2,4839E-25	4
**FAM26F**	9,9941E-30	0,7705667	2,6881E-25	4
**DPYD**	2,1368E-29	0,429099	5,7473E-25	4
**GPR171**	6,2087E-29	0,7520204	1,6699E-24	4
**FYN**	6,667E-29	0,30332565	1,7932E-24	4
**H1FX**	7,9711E-29	0,28894125	2,144E-24	4
**PNRC1**	8,3368E-29	0,15406924	2,2424E-24	4
**HOPX**	1,9559E-28	0,38807767	5,2608E-24	4
**EPB41**	2,7991E-28	0,34652643	7,5287E-24	4
**GSTM2**	4,8167E-28	0,92363257	1,2955E-23	4
**CHN2**	6,3864E-28	1,4877211	1,7178E-23	4
**NEAT1**	3,0166E-27	0,45081998	8,1137E-23	4
**BAIAP3**	3,1335E-27	1,3848971	8,4283E-23	4
**HIST1H2AC**	4,0605E-27	0,69261033	1,0922E-22	4
**EVL**	6,6621E-27	0,20122693	1,7919E-22	4
**MT-ND4**	1,5128E-26	0,17335495	4,069E-22	4
**AC006129.2**	1,6377E-26	0,43040368	4,4049E-22	4
**PLAG1**	2,0148E-25	1,3657869	5,4193E-21	4
**RPL13A**	2,0797E-25	0,16720067	5,5936E-21	4

**GPR55**	2,3454E-25	2,76498216	6,3085E-21	4
**LAIR1**	4,3529E-25	0,70518474	1,1708E-20	4
**HIC1**	4,899E-25	2,00240904	1,3177E-20	4
**ITM2A**	8,9788E-25	0,30220199	2,415E-20	4
**SARAF**	1,6474E-24	0,13269005	4,4309E-20	4
**RABGAP1L**	2,5961E-24	0,42374545	6,9828E-20	4
**ADAM19**	2,807E-24	0,8429226	7,55E-20	4
**RORA**	2,8524E-24	0,30321639	7,6722E-20	4
**LINC-PINT**	4,7373E-24	0,37590261	1,2742E-19	4
**MT-CO1**	8,5799E-24	0,11626497	2,3077E-19	4
**MT-CO3**	2,2451E-23	0,13997541	6,0387E-19	4
**CH17-373J23.1**	1,1468E-22	0,71704974	3,0844E-18	4
**SAMSN1**	4,0257E-22	0,4890966	1,0828E-17	4
**ETS1**	5,3847E-22	0,23797058	1,4483E-17	4
**TCF7**	6,992E-22	0,21178511	1,8807E-17	4
**CELF2**	1,1699E-21	0,31583372	3,1466E-17	4
**PROK2**	1,3155E-21	2,09565463	3,5384E-17	4
**EZR**	1,8637E-21	0,27665336	5,0128E-17	4
**PAG1**	3,3059E-21	0,4178365	8,8918E-17	4
**ITK**	4,0285E-21	0,35251293	1,0836E-16	4
**SLC38A2**	5,7287E-21	0,53808519	1,5408E-16	4
**LINC00152**	6,3526E-21	0,58270578	1,7087E-16	4
**MAML2**	9,1116E-21	0,41865342	2,4508E-16	4
**ANKRD44**	1,2993E-20	0,28083365	3,4947E-16	4
**UBL3**	2,2254E-20	0,52429479	5,9857E-16	4
**OGT**	3,4533E-20	0,44799048	9,2882E-16	4
**PRDM1**	6,8608E-20	0,35433932	1,8453E-15	4
**RP11-290D2.6**	7,878E-20	0,27205129	2,1189E-15	4
**ZBTB38**	1,7357E-19	0,44944877	4,6686E-15	4
**FOXJ3**	2,1415E-19	0,47574348	5,7599E-15	4
**ST3GAL1**	9,0427E-19	0,46370769	2,4322E-14	4
**HIST1H1D**	1,8E-18	0,29554621	4,8416E-14	4
**MPP1**	3,2105E-18	0,96162625	8,6354E-14	4
**AC015849.2**	5,0452E-18	1,36462577	1,357E-13	4
**EPS15**	6,1517E-18	0,38804893	1,6546E-13	4
**NIN**	6,4165E-18	0,50136618	1,7259E-13	4
**PDE3B**	6,5064E-18	0,33066433	1,75E-13	4
**SUPT3H**	7,2495E-18	0,3546226	1,9499E-13	4
**NCF4**	8,1667E-18	0,52979881	2,1966E-13	4
**IL12RB2**	8,7503E-18	0,77703192	2,3536E-13	4
**PARP8**	9,3137E-18	0,27747922	2,5051E-13	4

**MT-CYB**	1,3324E-17	0,10767081	3,5837E-13	4
**CDC14A**	1,413E-17	0,32045108	3,8004E-13	4
**PTGER2**	1,8004E-17	0,32951852	4,8425E-13	4
**DDX17**	2,0071E-17	0,28049279	5,3985E-13	4
**AKT3**	2,9595E-17	0,41457746	7,9603E-13	4
**DZIP3**	5,2826E-17	0,87049114	1,4209E-12	4
**COL18A1**	5,5634E-17	1,21844579	1,4964E-12	4
**GPR15**	5,9765E-17	1,31296946	1,6075E-12	4
**MBNL1**	6,4167E-17	0,19569918	1,7259E-12	4
**HIST1H1C**	1,351E-16	0,39372084	3,6339E-12	4
**PDCD4**	1,6556E-16	0,22706993	4,4529E-12	4
**SYNE2**	4,1591E-16	0,21737744	1,1187E-11	4
**CHD2**	4,3954E-16	0,35797197	1,1822E-11	4
**SF1**	4,4426E-16	0,20456288	1,1949E-11	4
**RPS6KA5**	6,0832E-16	0,52335878	1,6362E-11	4
**LINC00092**	9,6819E-16	1,42056203	2,6041E-11	4
**STK4**	1,4723E-15	0,24058716	3,96E-11	4
**CASP8**	1,5203E-15	0,38655578	4,0891E-11	4
**NCALD**	1,9112E-15	0,48671861	5,1406E-11	4
**YPEL2**	3,0504E-15	0,79219618	8,2047E-11	4
**GBP2**	3,131E-15	0,37566523	8,4215E-11	4
**CCDC109B**	3,6308E-15	0,19850358	9,7656E-11	4
**TMEM200A**	4,1754E-15	1,80358149	1,1231E-10	4
**PPP3CA**	5,9725E-15	0,44678221	1,6064E-10	4
**LPIN2**	6,3608E-15	0,36215123	1,7109E-10	4
**PCED1B**	8,3981E-15	0,30801376	2,2588E-10	4
**N4BP2L2**	1,2865E-14	0,21864478	3,4602E-10	4
**ZSWIM5**	1,3975E-14	0,83826463	3,7587E-10	4
**S100A10**	1,5041E-14	0,12579712	4,0456E-10	4
**CDC42SE1**	1,8193E-14	0,24064748	4,8934E-10	4
**RP11-223C24.1**	1,9363E-14	0,65289455	5,208E-10	4
**GFI1**	2,0973E-14	0,81265481	5,6411E-10	4
**TEX14**	3,0424E-14	0,67341662	8,1831E-10	4
**ZRANB2**	3,1959E-14	0,29459952	8,5959E-10	4
**TUBA1A**	4,4033E-14	0,28395289	1,1843E-09	4
**CITED4**	5,5084E-14	0,44759027	1,4816E-09	4
**HIST1H2AG**	8,7221E-14	0,60709769	2,346E-09	4
**CLDND1**	1,0607E-13	0,33316302	2,8529E-09	4
**RCAN3**	1,0726E-13	0,25188147	2,8849E-09	4
**ODF2L**	1,2314E-13	0,31989636	3,3121E-09	4
**JUN**	1,26E-13	0,29803139	3,389E-09	4

**PRKCQ**	1,3743E-13	0,36468723	3,6964E-09	4
**MYC**	1,464E-13	0,25282646	3,9377E-09	4
**PVRL3**	1,5161E-13	0,79738065	4,0779E-09	4
**ADK**	3,7516E-13	0,34843477	1,0091E-08	4
**KLF10**	4,3871E-13	0,78484153	1,18E-08	4
**SLC44A3**	4,6052E-13	1,47797062	1,2387E-08	4
**HERC1**	4,834E-13	0,37943861	1,3002E-08	4
**H1F0**	5,0693E-13	1,19461077	1,3635E-08	4
**AHR**	5,0971E-13	0,50521937	1,371E-08	4
**PHF3**	6,3514E-13	0,27502293	1,7083E-08	4
**SLC4A7**	6,6646E-13	0,47700546	1,7926E-08	4
**PERP**	7,278E-13	0,5112867	1,9576E-08	4
**OXNAD1**	1,0261E-12	0,29845573	2,7598E-08	4
**MT-ND2**	1,0594E-12	0,1205533	2,8494E-08	4
**TRADD**	1,3639E-12	0,21884836	3,6685E-08	4
**CDK6**	1,4364E-12	0,40537739	3,8634E-08	4
**PVT1**	1,5046E-12	0,32544465	4,0469E-08	4
**AP000769.1**	1,6176E-12	0,57220871	4,3508E-08	4
**PCNX**	1,6232E-12	0,31471324	4,3659E-08	4
**SLFN12L**	2,1662E-12	0,32221064	5,8265E-08	4
**RORA-AS1**	2,1886E-12	0,45947862	5,8867E-08	4
**ARGLU1**	2,4411E-12	0,1929596	6,5659E-08	4
**RAP1A**	2,7816E-12	0,220535	7,4817E-08	4
**STK17B**	3,3956E-12	0,19250322	9,1331E-08	4
**UBE2E2**	3,4065E-12	1,21357759	9,1626E-08	4
**TNRC6B**	3,6973E-12	0,27281163	9,9445E-08	4
**SPRY1**	4,4163E-12	1,02510816	1,1879E-07	4
**RP5-1171I10.5**	4,5696E-12	0,38454216	1,2291E-07	4
**APBB1IP**	5,2379E-12	0,21971952	1,4088E-07	4
**PPP2R5C**	8,3403E-12	0,20992145	2,2433E-07	4
**VOPP1**	1,0319E-11	0,27765389	2,7754E-07	4
**SSBP2**	1,0644E-11	0,41865362	2,8629E-07	4
**NRIP1**	1,0773E-11	0,58814225	2,8976E-07	4
**VPS13C**	1,148E-11	0,24312664	3,0879E-07	4
**ARHGEF3**	1,3557E-11	0,34403725	3,6464E-07	4
**ELF1**	1,4176E-11	0,24941929	3,813E-07	4
**SON**	1,5654E-11	0,15889261	4,2103E-07	4
**ACVR1**	1,7237E-11	0,71861172	4,6363E-07	4
**SPINK2**	1,884E-11	1,10705036	5,0675E-07	4
**PTP4A2**	2,4639E-11	0,1577688	6,6272E-07	4
**SRPK2**	2,7178E-11	0,27388157	7,3101E-07	4

**HELZ**	2,7336E-11	0,39628572	7,3526E-07	4
**BBC3**	2,8324E-11	0,48020451	7,6183E-07	4
**ATP2B1**	3,0728E-11	0,32276232	8,2648E-07	4
**SPON1**	3,2289E-11	0,93141453	8,6847E-07	4
**PUM2**	3,3034E-11	0,3494064	8,8853E-07	4
**TMEM123**	3,531E-11	0,16997354	9,4972E-07	4
**FBXL8**	3,7916E-11	0,4585344	1,0198E-06	4
**LSP1**	4,5646E-11	0,13598234	1,2277E-06	4
**ANK3**	5,0079E-11	0,27250102	1,347E-06	4
**ARRDC3**	5,3112E-11	0,45944962	1,4286E-06	4
**NCOA3**	5,3556E-11	0,47174562	1,4405E-06	4
**TTC14**	5,406E-11	0,35138851	1,4541E-06	4
**RPS20**	5,5257E-11	0,22369594	1,4863E-06	4
**CGGBP1**	5,8353E-11	0,28158144	1,5695E-06	4
**MGEA5**	6,0108E-11	0,27699689	1,6167E-06	4
**RAB34**	6,2745E-11	0,98292691	1,6877E-06	4
**USP33**	6,325E-11	0,40685634	1,7012E-06	4
**HIST2H2BF**	6,5597E-11	0,71755648	1,7644E-06	4
**TMX4**	6,6414E-11	0,34050531	1,7863E-06	4
**MPZL3**	7,3389E-11	0,35315791	1,9739E-06	4
**YPEL3**	7,6091E-11	0,1415423	2,0466E-06	4
**ARL6IP5**	7,7557E-11	0,12427576	2,086E-06	4
**KLF2**	9,958E-11	0,10360163	2,6784E-06	4
**C9orf142**	9,977E-11	0,14671571	2,6835E-06	4
**USP25**	1,0104E-10	0,37841725	2,7177E-06	4
**MIS18BP1**	1,1931E-10	0,35050395	3,2092E-06	4
**TMEM220**	1,3211E-10	0,98014786	3,5533E-06	4
**ATF7IP**	1,3227E-10	0,31581778	3,5576E-06	4
**CYLD**	1,3684E-10	0,2356546	3,6806E-06	4
**NR3C1**	1,4429E-10	0,27195288	3,8809E-06	4
**GCC2**	1,4909E-10	0,26288157	4,01E-06	4
**FUS**	1,5229E-10	0,20972876	4,0963E-06	4
**ZNF75A**	1,8016E-10	0,56559373	4,8459E-06	4
**PACS1**	1,8746E-10	0,29799205	5,0421E-06	4
**PRRC2C**	2,0117E-10	0,20241181	5,4109E-06	4
**RNF38**	2,1599E-10	0,37596883	5,8095E-06	4
**PDE7A**	2,1905E-10	0,29346355	5,8917E-06	4
**MDFIC**	2,2284E-10	0,25110982	5,9937E-06	4
**SRSF10**	2,3574E-10	0,18348778	6,3408E-06	4
**HACD4**	2,3621E-10	0,31508589	6,3532E-06	4
**MAPRE2**	3,1539E-10	0,3533703	8,483E-06	4

**PLEKHO1**	3,3636E-10	0,31160083	9,0469E-06	4
**C16orf74**	3,3655E-10	0,50892851	9,0523E-06	4
**CHD7**	3,6818E-10	0,45217612	9,9029E-06	4
**ISYNA1**	3,8752E-10	0,48720081	1,0423E-05	4
**CXCR3**	3,9839E-10	0,2216695	1,0715E-05	4
**THUMPD3-AS1**	4,3005E-10	0,42130407	1,1567E-05	4
**PIK3R5**	4,5659E-10	0,30063309	1,2281E-05	4
**TP53INP1**	4,6813E-10	0,59527785	1,2591E-05	4
**HIST1H2BC**	4,9419E-10	0,41879905	1,3292E-05	4
**CBLB**	4,9588E-10	0,28021897	1,3338E-05	4
**DDHD1**	5,3234E-10	0,4274096	1,4318E-05	4
**RPS6KA3**	5,4294E-10	0,25137079	1,4603E-05	4
**FCMR**	6,0399E-10	0,20185372	1,6246E-05	4
**FOXP1**	6,1626E-10	0,1762266	1,6576E-05	4
**CD101**	7,4086E-10	1,36771056	1,9927E-05	4
**CD3G**	8,7755E-10	0,15265114	2,3604E-05	4
**SMAD5**	9,1515E-10	0,61085655	2,4615E-05	4
**CAST**	9,3689E-10	0,16489077	2,52E-05	4
**STT3B**	1,0367E-09	0,22311618	2,7884E-05	4
**DDX24**	1,0421E-09	0,17726614	2,803E-05	4
**ZNF800**	1,0825E-09	0,26207634	2,9117E-05	4
**RBL2**	1,218E-09	0,19369251	3,276E-05	4
**ZC3HAV1**	1,2291E-09	0,19879587	3,3058E-05	4
**ZFAS1**	1,2792E-09	0,11807301	3,4407E-05	4
**PROSER2**	1,3098E-09	1,36493905	3,5229E-05	4
**HIST1H2BG**	1,3395E-09	0,92032436	3,6028E-05	4
**CEACAM21**	1,3428E-09	0,38434635	3,6118E-05	4
**HNRNPU**	1,6697E-09	0,17847377	4,491E-05	4
**ANKRD12**	1,6727E-09	0,16363864	4,4991E-05	4
**RP11-325F22.2**	1,791E-09	0,59545699	4,8173E-05	4
**RICTOR**	1,8069E-09	0,3380874	4,8599E-05	4
**RP11-160E2.6**	1,9417E-09	0,58061405	5,2227E-05	4
**AC009299.3**	1,9517E-09	0,63189175	5,2494E-05	4
**RP11-96H19.1**	2,3937E-09	0,86138197	6,4383E-05	4
**NME7**	2,4138E-09	0,5652063	6,4924E-05	4
**UBALD2**	2,4769E-09	0,15307005	6,662E-05	4
**LUC7L3**	2,7387E-09	0,26466794	7,3662E-05	4
**RP11-123O10.4**	2,8069E-09	1,29306392	7,5496E-05	4
**ARHGAP15**	3,282E-09	0,12377792	8,8275E-05	4
**PIK3CA**	3,409E-09	0,46414439	9,1692E-05	4
**PTP4A3**	3,5983E-09	0,46569142	9,6785E-05	4

**SRSF5**	3,9972E-09	0,10627775	0,00010751	4
**KRAS**	4,0196E-09	0,30569077	0,00010811	4
**LEPROTL1**	4,2925E-09	0,13257436	0,00011546	4
**KIF2A**	4,4307E-09	0,1719337	0,00011917	4
**FLOT1**	4,6123E-09	0,32797142	0,00012406	4
**UHRF2**	4,6284E-09	0,49334121	0,00012449	4
**IQGAP2**	5,4377E-09	0,19356942	0,00014626	4
**CEP95**	5,4924E-09	0,39716854	0,00014773	4
**GSTP1**	5,6765E-09	0,16939576	0,00015268	4
**DOCK8**	6,1476E-09	0,27683917	0,00016535	4
**INPP4B**	6,1859E-09	0,19609921	0,00016638	4
**GNAO1**	6,5242E-09	1,04579876	0,00017548	4
**PRDM2**	7,1708E-09	0,42221069	0,00019287	4
**PRKACB**	7,2377E-09	0,27406781	0,00019467	4
**RIMKLB**	7,5375E-09	0,75979755	0,00020274	4
**FBXL17**	7,8351E-09	0,33258418	0,00021074	4
**FBXW7**	8,6255E-09	0,39976202	0,000232	4
**AMD1**	9,2732E-09	0,2893806	0,00024942	4
**HAGHL**	9,3224E-09	0,54935555	0,00025074	4
**TANC2**	9,9126E-09	0,59850317	0,00026662	4
**FKBP5**	9,928E-09	0,26502891	0,00026703	4
**FAM102A**	1,0414E-08	0,29020117	0,00028009	4
**RSRP1**	1,0882E-08	0,17671059	0,0002927	4
**MFNG**	1,1609E-08	0,21451819	0,00031225	4
**ZNF644**	1,1772E-08	0,27565591	0,00031664	4
**HECA**	1,1929E-08	0,30784024	0,00032085	4
**DENND4A**	1,2627E-08	0,34120439	0,00033963	4
**TGFBR2**	1,307E-08	0,26389194	0,00035154	4
**REPS1**	1,3778E-08	0,38178411	0,0003706	4
**CD7**	1,4538E-08	0,13559449	0,00039102	4
**HEXDC**	1,4704E-08	0,33258366	0,00039549	4
**KPNB1**	1,4992E-08	0,29668544	0,00040323	4
**SECISBP2**	1,6079E-08	0,28606002	0,00043248	4
**CTB-4E7.1**	1,6518E-08	0,66034555	0,00044429	4
**SSBP4**	1,6965E-08	0,18879714	0,0004563	4
**UBASH3B**	1,6987E-08	0,64193355	0,00045689	4
**PRKD3**	1,7844E-08	0,42517755	0,00047996	4
**CLEC2D**	1,8991E-08	0,20831547	0,0005108	4
**TESPA1**	1,9222E-08	0,24834158	0,00051702	4
**RAP2C-AS1**	2,2597E-08	0,79905347	0,00060779	4
**PLB1**	2,3589E-08	1,03588439	0,00063449	4

**STK38**	2,4166E-08	0,23711584	0,00064998	4
**TBRG1**	2,6687E-08	0,21914779	0,00071779	4
**NMRK1**	2,6998E-08	0,28151249	0,00072617	4
**NBPF19**	2,8237E-08	0,4031836	0,00075949	4
**THADA**	2,8373E-08	0,45518864	0,00076316	4
**RNF214**	2,9517E-08	0,35650533	0,00079392	4
**ADGRE5**	2,9617E-08	0,23611962	0,00079661	4
**LINC00243**	2,9711E-08	0,52767965	0,00079915	4
**MRPL10**	3,0635E-08	0,23966984	0,00082399	4
**ESYT2**	3,3895E-08	0,28061064	0,00091168	4
**U2SURP**	3,3943E-08	0,20965392	0,00091296	4
**RP1-111C20.4**	3,5527E-08	0,52710266	0,00095558	4
**MSH3**	3,5573E-08	0,45181464	0,0009568	4
**TRAF3IP3**	3,7048E-08	0,12555102	0,00099649	4
**SOS2**	3,8043E-08	0,43625041	0,00102325	4
**HNRNPH1**	3,8333E-08	0,23784645	0,00103106	4
**FTX**	3,8392E-08	0,48408478	0,00103263	4
**STAG1**	4,5642E-08	0,3140186	0,00122763	4
**SP100**	4,76E-08	0,15029156	0,0012803	4
**RP4-666F24.3**	4,9483E-08	0,73065789	0,00133095	4
**HIST1H4C**	5,1329E-08	0,16319604	0,0013806	4
**SNHG25**	5,1922E-08	0,20752095	0,00139655	4
**TAF15**	5,8196E-08	0,27321905	0,00156529	4
**MYO15B**	5,8703E-08	1,12112653	0,00157895	4
**BRWD1**	6,2756E-08	0,26533016	0,00168796	4
**SERPINB6**	6,44E-08	0,35594491	0,00173216	4
**PAN3**	6,5452E-08	0,32013869	0,00176045	4
**RP11-703M24.5**	6,7593E-08	0,46404059	0,00181804	4
**CHST11**	7,09E-08	0,23608996	0,00190699	4
**TRAT1**	7,1211E-08	0,2249042	0,00191536	4
**REV3L**	7,3747E-08	0,2945199	0,00198358	4
**ZNF22**	7,6313E-08	0,34657034	0,00205259	4
**PIK3IP1**	7,8798E-08	0,18851677	0,00211943	4
**ITGAE**	7,9534E-08	0,37849101	0,00213923	4
**CD55**	8,1452E-08	0,23847302	0,00219082	4
**LBH**	8,1886E-08	0,15259821	0,00220249	4
**FLJ37453**	8,3979E-08	0,43533841	0,00225879	4
**CNST**	8,7659E-08	0,29202816	0,00235776	4
**KLF9**	9,8305E-08	0,29510042	0,0026441	4
**LANCL1**	1,0281E-07	0,51062161	0,0027654	4
**PCNXL2**	1,113E-07	0,32905524	0,0029935	4

**LINC00954**	1,1223E-07	0,80958485	0,00301861	4
**NAP1L4**	1,1314E-07	0,20679174	0,00304314	4
**INADL**	1,1535E-07	0,36724818	0,00310245	4
**CPSF6**	1,2329E-07	0,23743942	0,00331625	4
**LGALS1**	1,2593E-07	0,29516815	0,00338718	4
**MYADM**	1,2844E-07	0,27406685	0,00345452	4
**SEPT6**	1,3038E-07	0,14377434	0,00350686	4
**PRKCH**	1,3986E-07	0,18340231	0,0037618	4
**KMT2A**	1,4051E-07	0,20866684	0,00377935	4
**NUCKS1**	1,4099E-07	0,17842135	0,0037922	4
**IKZF3**	1,4173E-07	0,21210418	0,00381224	4
**SRSF11**	1,4708E-07	0,15671959	0,00395609	4
**JMJD1C**	1,5333E-07	0,23489868	0,00412414	4
**GABPB1-AS1**	1,62E-07	0,32072738	0,0043572	4
**UST**	1,6346E-07	0,50091286	0,00439646	4
**LINC01259**	1,6885E-07	0,79172174	0,00454153	4
**PTMS**	1,6981E-07	0,79659075	0,00456732	4
**NCOA2**	1,7173E-07	0,34675667	0,00461911	4
**PKN2**	1,7404E-07	0,3377563	0,00468103	4
**XRN1**	1,7457E-07	0,25776965	0,00469539	4
**EIF5A**	1,7494E-07	0,13159918	0,00470539	4
**AMMECR1**	1,7893E-07	0,46223688	0,00481267	4
**NSMAF**	1,8873E-07	0,34991364	0,00507616	4
**CDC42SE2**	1,9734E-07	0,14369727	0,00530785	4
**NPC1**	2,0117E-07	0,47094567	0,00541082	4
**KIAA0040**	2,0723E-07	0,43586386	0,00557377	4
**FNBP4**	2,1065E-07	0,20283785	0,00566578	4
**CTD-3184A7.4**	2,1162E-07	0,23181501	0,005692	4
**JAK1**	2,1975E-07	0,10717234	0,00591059	4
**ZC3H12B**	2,2339E-07	1,13352767	0,0060085	4
**UBR2**	2,3032E-07	0,29491257	0,00619487	4
**UXS1**	2,3914E-07	0,32072813	0,0064322	4
**EMB**	2,7547E-07	0,17415227	0,00740927	4
**PELO**	2,77E-07	0,46302484	0,00745046	4
**THEMIS**	2,7898E-07	0,16860776	0,00750384	4
**FOXP3**	2,9348E-07	0,96825811	0,00789362	4
**ZDHHC14**	2,9452E-07	0,57415733	0,00792172	4
**EIF4E3**	2,9624E-07	0,30337877	0,00796794	4
**GPHN**	3,1197E-07	0,3410805	0,00839115	4
**PLCL2**	3,1287E-07	0,21554717	0,00841534	4
**BCL2**	3,3747E-07	0,17312846	0,00907693	4

**PLCD1**	3,4834E-07	0,40706341	0,00936919	4
**RNF157**	3,7179E-07	0,41066166	0,00999999	4
**SLK**	3,7805E-07	0,31657912	0,01016832	4
**PCM1**	3,8324E-07	0,22853202	0,01030806	4
**CTB-133G6.1**	3,949E-07	0,2904955	0,01062159	4
**MEGF6**	4,0684E-07	0,65162323	0,01094266	4
**DDX3X**	4,1183E-07	0,24174221	0,01107708	4
**BTBD9**	4,139E-07	0,31758961	0,0111327	4
**CCNL1**	4,1695E-07	0,14011824	0,0112146	4
**TARSL2**	4,2776E-07	0,27645934	0,01150533	4
**CUL5**	4,3528E-07	0,32806356	0,01170766	4
**USP24**	4,6703E-07	0,37562606	0,0125618	4
**ERN1**	4,7396E-07	0,16689232	0,01274799	4
**DNAI2**	4,8561E-07	0,7521539	0,01306138	4
**TRAF1**	4,8982E-07	0,34315333	0,01317474	4
**CACNA1I**	5,3702E-07	0,95950997	0,01444431	4
**CLASP1**	5,3736E-07	0,41087812	0,01445348	4
**CDR2**	5,7584E-07	0,25036761	0,01548844	4
**TNKS**	5,7637E-07	0,40822055	0,01550272	4
**CSDE1**	5,8621E-07	0,13451926	0,0157672	4
**SMURF2**	6,0238E-07	0,4322418	0,0162023	4
**SNHG7**	6,0423E-07	0,17841675	0,01625185	4
**AC073115.6**	6,6072E-07	1,49333768	0,01777131	4
**ZNF665**	6,657E-07	0,75623277	0,01790522	4
**RCSD1**	6,8452E-07	0,15622111	0,01841156	4
**CHRM3-AS2**	6,9381E-07	0,39521462	0,01866132	4
**ZDBF2**	6,9467E-07	0,62322223	0,01868454	4
**TTC17**	7,2441E-07	0,26909533	0,01948434	4
**UBE2E1**	7,4235E-07	0,27655021	0,01996686	4
**TRBC2**	7,4763E-07	0,12676011	0,02010906	4
**TNFRSF1B**	7,5209E-07	0,26045564	0,02022885	4
**NDFIP1**	7,7841E-07	0,15400558	0,020937	4
**MED23**	7,9734E-07	0,32520299	0,02144612	4
**WDR63**	8,5414E-07	1,23866762	0,0229739	4
**PPP3CC**	8,6038E-07	0,1926245	0,02314165	4
**STMN3**	8,6149E-07	0,18074361	0,02317156	4
**CFLAR**	8,7478E-07	0,17483851	0,02352907	4
**SFPQ**	8,8379E-07	0,14738902	0,0237713	4
**SMAD4**	9,1176E-07	0,28547789	0,02452373	4
**SND1**	9,4171E-07	0,25609627	0,02532914	4
**PHC3**	1,0166E-06	0,25887748	0,02734336	4

**PLCB1**	1,0322E-06	0,19790639	0,02776402	4
**NKTR**	1,103E-06	0,21969097	0,02966782	4
**CISH**	1,1145E-06	0,30572951	0,02997758	4
**AC131056.3**	1,1234E-06	1,08119492	0,03021677	4
**NAP1L1**	1,137E-06	0,12540116	0,03058056	4
**CLECL1**	1,1459E-06	0,87733109	0,03082044	4
**KLHL5**	1,1532E-06	0,35355702	0,03101841	4
**CDV3**	1,1667E-06	0,18590282	0,0313806	4
**HSBP1L1**	1,1854E-06	0,29044589	0,03188385	4
**MAPKAPK5-AS1**	1,2144E-06	0,19577384	0,03266272	4
**ZNF618**	1,229E-06	1,17573361	0,03305634	4
**DCAF8**	1,2869E-06	0,44102508	0,03461376	4
**ZEB1**	1,3126E-06	0,27818269	0,03530401	4
**GNB5**	1,3268E-06	0,41597972	0,03568655	4
**RASSF1**	1,3368E-06	0,21635798	0,03595484	4
**FOXO3**	1,4178E-06	0,40601706	0,03813329	4
**MAN1A1**	1,4725E-06	0,3327602	0,03960619	4
**ZSCAN18**	1,5336E-06	0,41613402	0,0412481	4
**SH3KBP1**	1,5408E-06	0,17135504	0,04144411	4
**SMARCC1**	1,5506E-06	0,23611415	0,0417076	4
**POT1-AS1**	1,5576E-06	1,14387555	0,0418955	4
**ROCK1**	1,6074E-06	0,24791413	0,04323491	4
**MAP3K5**	1,6124E-06	0,24816194	0,0433693	4
**SYNC**	1,6188E-06	1,19811462	0,04354066	4
**CYTH1**	1,6293E-06	0,12814993	0,04382428	4
**EZH1**	1,6665E-06	0,43132493	0,04482466	4
**RP11-293M10.1**	1,7917E-06	0,66419013	0,04819246	4
**LEF1**	0	1,44920505	0	5
**CCR7**	0	1,69134641	0	5
**SELL**	0	1,56519577	0	5
**TSHZ2**	0	4,13857669	0	5
**PASK**	1,498E-289	1,97741867	4,03E-285	5
**ANKRD55**	9,268E-259	4,3092308	2,493E-254	5
**ADTRP**	1,53E-257	3,22338645	4,116E-253	5
**TMSB10**	2,546E-253	0,41816699	6,849E-249	5
**TCF7**	4,162E-228	0,79676084	1,119E-223	5
**CXCR5**	5,691E-225	4,83150232	1,531E-220	5
**LINC00402**	5,23E-224	2,99206985	1,407E-219	5
**ACTN1**	1,596E-208	2,31037216	4,292E-204	5
**TCEA3**	1,795E-199	2,24281847	4,828E-195	5
**MARCKSL1**	6,965E-187	1,70397048	1,873E-182	5

**CD27**	3,877E-179	0,86327074	1,043E-174	5
**TIGIT**	1,408E-176	3,23283427	3,786E-172	5
**SARAF**	8,197E-160	0,42019395	2,205E-155	5
**MALAT1**	1,01E-154	0,376291	2,716E-150	5
**IL6ST**	2,276E-151	1,53548829	6,121E-147	5
**ARID5B**	5,213E-146	1,29719639	1,402E-141	5
**FYB**	1,67E-140	0,67512154	4,491E-136	5
**NGFRAP1**	4,422E-135	1,51038617	1,189E-130	5
**AK5**	9,825E-127	2,34425347	2,643E-122	5
**TBC1D4**	4,724E-126	1,61501188	1,271E-121	5
**FOXP1**	4,166E-117	0,76857232	1,12E-112	5
**MAL**	8,657E-113	0,68823671	2,328E-108	5
**CERS6**	3,937E-102	2,18209252	1,059E-97	5
**AIF1**	3,669E-101	2,04833174	9,8676E-97	5
**RIC3**	3,901E-100	1,58167996	1,0493E-95	5
**MDS2**	8,7919E-93	1,61815125	2,3647E-88	5
**SESN3**	1,0282E-92	1,69521833	2,7656E-88	5
**SULT1B1**	4,4791E-85	2,71007732	1,2047E-80	5
**LEPROTL1**	1,2315E-78	0,51441621	3,3124E-74	5
**C1orf228**	8,5341E-76	1,08855346	2,2954E-71	5
**MAN2A1**	2,1692E-73	1,33695549	5,8344E-69	5
**MAML2**	2,5464E-73	0,85302884	6,8492E-69	5
**EPHA1-AS1**	5,4687E-72	2,23231824	1,4709E-67	5
**LINC00861**	1,3688E-71	0,60124158	3,6817E-67	5
**CMTM8**	1,8247E-71	1,13410868	4,9079E-67	5
**FCMR**	2,9022E-71	0,6423782	7,8061E-67	5
**PELI1**	1,7399E-68	1,32374041	4,6799E-64	5
**LINC01550**	8,5942E-68	1,13142956	2,3116E-63	5
**SERINC5**	1,365E-67	0,79852565	3,6715E-63	5
**PIK3IP1**	1,8126E-65	0,60355694	4,8753E-61	5
**PRKCA**	3,3904E-64	0,89515992	9,1191E-60	5
**ICOS**	7,0825E-64	1,20147537	1,905E-59	5
**APP**	7,6324E-64	1,27143245	2,0529E-59	5
**RPL39**	1,115E-63	0,12307287	2,9991E-59	5
**INPP4B**	2,9987E-61	0,62826159	8,0656E-57	5
**EPHX2**	3,0328E-61	1,21559039	8,1573E-57	5
**RP4-678D15.1**	1,231E-60	5,31828813	3,3111E-56	5
**SMCHD1**	3,1893E-60	0,54474432	8,5782E-56	5
**SFXN1**	3,5359E-59	0,82161077	9,5104E-55	5
**ITGB2-AS1**	2,7796E-58	0,84004754	7,4764E-54	5
**RP11-223C24.1**	3,4697E-58	1,3523995	9,3323E-54	5

**NDFIP1**	5,4448E-58	0,53807157	1,4645E-53	5
**CCDC109B**	1,0099E-57	0,47062171	2,7163E-53	5
**NELL2**	2,541E-57	0,81398819	6,8344E-53	5
**C1orf162**	5,2348E-56	1,08195986	1,408E-51	5
**LIMS1**	6,656E-56	0,70832688	1,7903E-51	5
**CD37**	7,8115E-56	0,30284672	2,1011E-51	5
**FAAH2**	1,9317E-55	1,03719431	5,1956E-51	5
**AC097713.4**	2,0796E-55	2,04658137	5,5934E-51	5
**ITPKB**	2,535E-53	0,919381	6,8183E-49	5
**AP3M2**	5,6125E-52	1,04864116	1,5096E-47	5
**HSPB1**	1,3034E-51	0,54650905	3,5057E-47	5
**GPA33**	2,5103E-51	2,2116881	6,7518E-47	5
**SEPT6**	4,7852E-51	0,52046475	1,2871E-46	5
**VOPP1**	7,5547E-51	0,63064026	2,032E-46	5
**SARDH**	1,6183E-47	2,43589768	4,3526E-43	5
**RP11-61O1.1**	6,6734E-47	3,42287927	1,795E-42	5
**C12orf57**	2,0693E-46	0,29991695	5,5657E-42	5
**CHMP7**	7,3304E-46	0,8841656	1,9717E-41	5
**SCML1**	2,8024E-45	2,4239194	7,5376E-41	5
**ID3**	7,1557E-45	1,85476086	1,9247E-40	5
**HRH2**	1,4591E-44	2,07301139	3,9246E-40	5
**SLC22A17**	3,7232E-44	1,99225353	1,0014E-39	5
**TMSB4X**	7,2908E-44	0,1629217	1,961E-39	5
**CAMK4**	9,0655E-44	0,48305681	2,4383E-39	5
**TGFBR2**	1,4463E-43	0,70805778	3,8901E-39	5
**TMEM2**	4,6721E-43	0,90489836	1,2567E-38	5
**LDHB**	8,1296E-43	0,19998056	2,1866E-38	5
**ITK**	2,9604E-42	0,58683409	7,9626E-38	5
**SH3YL1**	2,985E-42	0,62089768	8,0288E-38	5
**TMEM45B**	4,7296E-41	1,65902784	1,2721E-36	5
**SNHG25**	1,3558E-40	0,63002483	3,6467E-36	5
**MARCH3**	1,944E-40	2,24751207	5,2289E-36	5
**TXK**	2,1894E-40	0,89096749	5,8887E-36	5
**HINT1**	3,2058E-40	0,19369391	8,6226E-36	5
**BTG1**	5,9791E-40	0,20298313	1,6082E-35	5
**TCEAL2**	8,6256E-40	2,97264495	2,32E-35	5
**TRABD2A**	1,832E-39	0,7647817	4,9275E-35	5
**RASSF6**	3,4878E-39	2,95007981	9,3811E-35	5
**TIAM1**	3,7578E-39	1,34730478	1,0107E-34	5
**PTK2**	4,303E-38	3,52508461	1,1574E-33	5
**RAB3GAP1**	2,8292E-37	0,93064144	7,6097E-33	5

**EPHB6**	4,9045E-37	1,40637731	1,3192E-32	5
**RHOH**	7,5132E-37	0,45612887	2,0208E-32	5
**TESPA1**	2,3199E-36	0,52616105	6,2399E-32	5
**LDLRAP1**	1,0268E-35	0,59409873	2,7619E-31	5
**CMTM7**	1,2411E-35	0,55102036	3,3381E-31	5
**RBM38**	1,8583E-35	0,45420755	4,9983E-31	5
**ATM**	3,6994E-35	0,53815994	9,9503E-31	5
**DGKA**	5,518E-35	0,50494225	1,4842E-30	5
**LINC01336**	8,4561E-35	2,66513296	2,2744E-30	5
**SLC16A10**	1,0422E-34	2,2486734	2,8033E-30	5
**DDX5**	1,3007E-34	0,22325102	3,4986E-30	5
**BTG2**	1,3166E-34	0,54843571	3,5413E-30	5
**IL6R**	1,4568E-34	1,04813813	3,9184E-30	5
**RASA2**	2,3816E-34	0,59011783	6,4057E-30	5
**CDHR3**	3,0919E-34	1,87799601	8,3162E-30	5
**LIMS2**	6,1564E-34	1,70069573	1,6559E-29	5
**SUSD3**	6,8428E-34	0,61108592	1,8405E-29	5
**CD7**	1,8962E-33	0,39465156	5,1001E-29	5
**SRSF5**	3,7886E-33	0,24340526	1,019E-28	5
**RNASET2**	8,2609E-33	0,31803886	2,2219E-28	5
**APBA2**	9,1294E-33	1,09671201	2,4555E-28	5
**PPP1R2**	2,0772E-32	0,3968625	5,587E-28	5
**SPINT2**	2,3531E-32	0,65380566	6,3291E-28	5
**PNISR**	2,4915E-32	0,35639281	6,7014E-28	5
**FAM117B**	4,516E-32	1,10985192	1,2147E-27	5
**H1FX**	7,5187E-32	0,32051726	2,0223E-27	5
**CALM3**	1,432E-31	0,38081547	3,8517E-27	5
**PLAG1**	1,7491E-31	1,51709468	4,7046E-27	5
**RALGPS2**	9,4748E-31	1,45394671	2,5484E-26	5
**PKM**	6,7033E-30	0,32219416	1,803E-25	5
**ST8SIA1**	7,1828E-30	1,6268894	1,932E-25	5
**LINC01089**	7,9859E-30	0,96317282	2,148E-25	5
**EPHA1**	1,5222E-29	1,6741694	4,0942E-25	5
**BCL11B**	2,217E-29	0,49081043	5,9631E-25	5
**COL18A1**	3,7116E-29	1,54605873	9,9832E-25	5
**ATP6V0E2**	1,2938E-28	0,54719793	3,48E-24	5
**NAP1L4**	1,4175E-28	0,47410505	3,8127E-24	5
**PPP1CC**	1,4709E-28	0,4686936	3,9562E-24	5
**SGPP2**	1,5033E-28	2,14258237	4,0435E-24	5
**TLK1**	1,9539E-28	0,63815975	5,2555E-24	5
**FKBP1A**	2,3618E-28	0,44916652	6,3526E-24	5

**HMGN2**	3,0368E-28	0,31749835	8,1681E-24	5
**RNF138**	4,5435E-28	0,65501695	1,2221E-23	5
**BEX2**	4,984E-28	0,62683978	1,3406E-23	5
**MSL3**	5,4166E-28	0,77884161	1,4569E-23	5
**FYN**	8,5602E-28	0,32582175	2,3024E-23	5
**UBASH3B**	1,3327E-27	1,14874983	3,5847E-23	5
**FAM13A**	1,9566E-27	1,24188418	5,2627E-23	5
**GCNT4**	2,132E-27	2,80972624	5,7345E-23	5
**FAM118A**	3,9866E-27	0,83097045	1,0723E-22	5
**MICU3**	4,9591E-27	1,57004293	1,3339E-22	5
**SEPT9**	5,1175E-27	0,31444591	1,3765E-22	5
**STMN1**	5,3489E-27	0,98137297	1,4387E-22	5
**SMAP2**	5,6079E-27	0,37580949	1,5084E-22	5
**RAB30**	5,6111E-27	1,30640054	1,5092E-22	5
**OXNAD1**	7,5425E-27	0,4717992	2,0287E-22	5
**DENND5A**	8,9071E-27	1,14965721	2,3957E-22	5
**GCC2**	2,0229E-26	0,43699354	5,441E-22	5
**JUNB**	2,4546E-26	0,43150478	6,6021E-22	5
**TMEM123**	2,4918E-26	0,3059793	6,7021E-22	5
**SSH2**	3,8316E-26	0,51534501	1,0306E-21	5
**SNN**	8,8627E-26	1,18326151	2,3838E-21	5
**SOD1**	9,9393E-26	0,24926684	2,6734E-21	5
**TNFSF8**	1,2523E-25	0,53841205	3,3684E-21	5
**AC074289.1**	1,2954E-25	2,24038356	3,4841E-21	5
**HSBP1L1**	1,463E-25	0,85989807	3,9351E-21	5
**GPCPD1**	1,8556E-25	0,644105	4,9909E-21	5
**MAD1L1**	2,4254E-25	0,53435369	6,5235E-21	5
**LAT**	2,9618E-25	0,33939998	7,9665E-21	5
**PCED1B**	3,7996E-25	0,48554347	1,022E-20	5
**FKBP5**	7,0853E-25	0,59603406	1,9057E-20	5
**TPR**	1,3098E-24	0,51063942	3,5231E-20	5
**PRKCH**	1,6569E-24	0,36804844	4,4566E-20	5
**GPR183**	1,8131E-24	0,44369508	4,8767E-20	5
**TOX**	1,863E-24	1,48753183	5,011E-20	5
**COTL1**	1,9643E-24	0,26756971	5,2833E-20	5
**MPHOSPH8**	2,7992E-24	0,33923923	7,5291E-20	5
**CEP68**	3,1621E-24	0,83182915	8,5052E-20	5
**DOCK10**	3,1725E-24	0,39918096	8,533E-20	5
**PPP3CC**	4,2003E-24	0,43712596	1,1298E-19	5
**RCBTB2**	4,4557E-24	0,8475863	1,1984E-19	5
**MLLT3**	5,9904E-24	0,62080507	1,6112E-19	5

**FLOT1**	8,1154E-24	0,56667704	2,1828E-19	5
**RALA**	9,069E-24	0,59911784	2,4393E-19	5
**CORO1B**	9,5163E-24	0,3921618	2,5596E-19	5
**MYCBP2**	1,2164E-23	0,43461826	3,2718E-19	5
**FAM134B**	1,2553E-23	0,71324963	3,3763E-19	5
**FHIT**	1,3878E-23	1,07778797	3,7327E-19	5
**RGS10**	1,7788E-23	0,31396178	4,7845E-19	5
**SPATC1L**	4,7983E-23	1,20250037	1,2906E-18	5
**MBP**	4,8351E-23	0,43341426	1,3005E-18	5
**CD5**	5,028E-23	0,35720298	1,3524E-18	5
**ANK3**	5,7697E-23	0,47144437	1,5519E-18	5
**CD84**	5,9341E-23	1,14601866	1,5961E-18	5
**TSPAN14**	6,4375E-23	0,55729955	1,7315E-18	5
**PNRC1**	6,5739E-23	0,16324148	1,7682E-18	5
**SGTB**	6,8544E-23	0,65238654	1,8436E-18	5
**LMO7**	9,4604E-23	1,36979311	2,5446E-18	5
**PVT1**	1,3591E-22	0,53823981	3,6556E-18	5
**SLC9A9**	1,9084E-22	0,89944922	5,1331E-18	5
**ABLIM1**	2,0411E-22	0,48795666	5,4899E-18	5
**CITED4**	2,6533E-22	0,6257228	7,1365E-18	5
**MBNL1**	2,743E-22	0,26345432	7,3778E-18	5
**ZNF331**	4,0317E-22	0,67781587	1,0844E-17	5
**CDCA7L**	4,1659E-22	1,72317189	1,1205E-17	5
**PDE3B**	6,0217E-22	0,44955993	1,6196E-17	5
**SFI1**	8,2443E-22	0,76630482	2,2175E-17	5
**ATXN7L1**	1,2592E-21	0,9303361	3,3869E-17	5
**CPA5**	1,5834E-21	2,68274601	4,2589E-17	5
**TRIB2**	1,7838E-21	0,78822203	4,7979E-17	5
**ZC2HC1A**	2,2642E-21	1,64574999	6,0901E-17	5
**KALRN**	2,4816E-21	2,03782293	6,6748E-17	5
**ADD3**	3,4135E-21	0,30018864	9,1813E-17	5
**ADPRM**	3,5578E-21	0,75524771	9,5695E-17	5
**FBLN7**	4,8289E-21	0,84971158	1,2988E-16	5
**LIMD2**	4,8341E-21	0,19153849	1,3002E-16	5
**RP11-703M24.5**	4,8735E-21	0,84919333	1,3108E-16	5
**IPCEF1**	5,1361E-21	0,81180638	1,3815E-16	5
**NBEA**	6,6146E-21	2,43715814	1,7791E-16	5
**SYPL1**	6,6492E-21	0,47005813	1,7884E-16	5
**MAGEH1**	6,658E-21	0,86476432	1,7908E-16	5
**TSTD1**	7,027E-21	0,27360535	1,89E-16	5
**BCL7A**	8,4001E-21	1,99461675	2,2594E-16	5

**RGL4**	1,1691E-20	0,6550625	3,1446E-16	5
**CNST**	1,1761E-20	0,53630662	3,1633E-16	5
**DDX24**	1,3294E-20	0,30396033	3,5756E-16	5
**GYPC**	1,4099E-20	0,32227062	3,7922E-16	5
**SNHG7**	1,8082E-20	0,3893676	4,8634E-16	5
**C1orf112**	1,8462E-20	1,37175392	4,9658E-16	5
**ESR1**	2,2716E-20	2,42667911	6,1098E-16	5
**SEMA4D**	2,7392E-20	0,43123243	7,3678E-16	5
**EPHA4**	2,7634E-20	0,79121841	7,4328E-16	5
**PACS1**	3,0982E-20	0,46624888	8,3333E-16	5
**BCAS4**	3,5363E-20	0,92721243	9,5116E-16	5
**PTPRC**	4,3383E-20	0,16476979	1,1669E-15	5
**TMEM156**	4,4607E-20	0,64059736	1,1998E-15	5
**DDX6**	4,522E-20	0,34756101	1,2163E-15	5
**NR3C1**	8,788E-20	0,45207471	2,3637E-15	5
**ZBTB20**	9,6973E-20	0,42296362	2,6083E-15	5
**RIC1**	1,1485E-19	0,63914445	3,089E-15	5
**RP11-61O1.2**	1,2184E-19	3,13111058	3,2772E-15	5
**CYLD**	1,3593E-19	0,34628387	3,656E-15	5
**RP1-167A14.2**	1,3626E-19	1,11388084	3,6649E-15	5
**PCSK1N**	1,6043E-19	0,80053673	4,3151E-15	5
**CCM2**	1,6429E-19	0,38804099	4,419E-15	5
**KANK1**	1,9874E-19	1,23969436	5,3455E-15	5
**BACH1**	2,2622E-19	0,85144874	6,0847E-15	5
**AC104820.2**	2,3786E-19	0,79063117	6,3977E-15	5
**DNPH1**	3,1644E-19	0,47905211	8,5112E-15	5
**ZNF101**	3,3683E-19	0,61133727	9,0598E-15	5
**PABPC1**	3,5145E-19	0,13145199	9,4529E-15	5
**HYKK**	4,5789E-19	1,50319734	1,2316E-14	5
**AC006129.2**	5,5185E-19	0,42518517	1,4843E-14	5
**PLCL1**	5,9852E-19	0,8150558	1,6098E-14	5
**KRT73-AS1**	6,194E-19	2,49188396	1,666E-14	5
**RP11-664D1.1**	7,6729E-19	1,3118792	2,0638E-14	5
**PIK3IP1-AS1**	7,7277E-19	1,39105976	2,0785E-14	5
**PIK3R1**	8,4993E-19	0,3270583	2,2861E-14	5
**RBM11**	8,5167E-19	1,79118153	2,2907E-14	5
**MAP3K14-AS1**	1,2966E-18	1,19784023	3,4874E-14	5
**PSIP1**	1,6538E-18	0,31541693	4,4483E-14	5
**LINC00891**	1,6552E-18	1,40701348	4,4519E-14	5
**UBQLN2**	1,687E-18	0,56589631	4,5376E-14	5
**KCNQ5**	1,6984E-18	1,502126	4,5682E-14	5

**HIVEP2**	2,3611E-18	0,58067074	6,3507E-14	5
**GSTK1**	2,5594E-18	0,17296714	6,8841E-14	5
**ANKRD44**	2,7205E-18	0,29623753	7,3174E-14	5
**STK4**	3,3901E-18	0,27741657	9,1184E-14	5
**PLAC8**	3,4341E-18	0,38390126	9,2367E-14	5
**NPIPB4**	4,465E-18	1,14544531	1,201E-13	5
**YBX3**	6,1228E-18	2,32506885	1,6469E-13	5
**PIGC**	6,3484E-18	0,46207957	1,7075E-13	5
**EPB41**	1,1643E-17	0,30667226	3,1317E-13	5
**CSGALNACT1**	1,4083E-17	0,81072766	3,7879E-13	5
**CHD2**	1,4427E-17	0,44079688	3,8805E-13	5
**C14orf132**	1,6997E-17	2,80706021	4,5717E-13	5
**COX6C**	1,7271E-17	0,18765849	4,6453E-13	5
**LDLRAD4**	2,9931E-17	0,66882861	8,0505E-13	5
**NDUFS5**	3,9569E-17	0,20720892	1,0643E-12	5
**LPAR6**	4,0778E-17	0,79310684	1,0968E-12	5
**KLHL5**	4,3412E-17	0,6497593	1,1677E-12	5
**AKTIP**	4,8158E-17	0,67226128	1,2953E-12	5
**BEX4**	5,732E-17	0,48032315	1,5417E-12	5
**PDE4DIP**	5,8536E-17	0,75565501	1,5745E-12	5
**CD3G**	6,4055E-17	0,23963954	1,7229E-12	5
**TSPAN33**	6,6706E-17	1,90110269	1,7942E-12	5
**TATDN1**	6,8132E-17	0,5576731	1,8325E-12	5
**PLPP1**	7,5228E-17	0,94035789	2,0234E-12	5
**SF1**	8,0346E-17	0,21916594	2,1611E-12	5
**USP6NL**	8,4767E-17	1,60553549	2,28E-12	5
**ITM2A**	9,099E-17	0,32231832	2,4473E-12	5
**BIRC2**	9,5336E-17	0,64186307	2,5642E-12	5
**ZCCHC11**	9,8982E-17	0,42265399	2,6623E-12	5
**TACC3**	9,927E-17	0,47624076	2,6701E-12	5
**RNF216**	1,0342E-16	0,4805249	2,7817E-12	5
**IFNGR2**	1,1606E-16	0,90614217	3,1216E-12	5
**SLC2A3**	1,2028E-16	0,422433	3,2351E-12	5
**LGALS9**	1,2413E-16	0,83965689	3,3389E-12	5
**TBC1D10C**	1,2653E-16	0,2481653	3,4032E-12	5
**PDE7A**	1,6581E-16	0,36235173	4,4598E-12	5
**PLXDC1**	1,7702E-16	1,52722951	4,7612E-12	5
**GCSAM**	1,9242E-16	0,72377029	5,1756E-12	5
**NFKBIZ**	2,1958E-16	0,48733293	5,9059E-12	5
**WHSC1L1**	2,3044E-16	0,26892453	6,1981E-12	5
**LY96**	2,5563E-16	1,09775929	6,8756E-12	5

**NFATC1**	2,7662E-16	0,55748622	7,4403E-12	5
**BNIP3L**	2,9475E-16	0,47522699	7,9279E-12	5
**VNN2**	3,7294E-16	1,3122458	1,0031E-11	5
**GPX4**	4,6359E-16	0,19867863	1,2469E-11	5
**C12orf42**	4,7193E-16	1,31431137	1,2693E-11	5
**FLJ27354**	6,7069E-16	1,09625551	1,8039E-11	5
**TECPR1**	7,6775E-16	0,84071226	2,065E-11	5
**CD3D**	8,1959E-16	0,16282112	2,2044E-11	5
**GPSM3**	8,6889E-16	0,1635859	2,3371E-11	5
**AES**	9,2705E-16	0,12996198	2,4935E-11	5
**IFNG-AS1**	1,0547E-15	0,46018218	2,8368E-11	5
**FAM65B**	1,0791E-15	0,26480107	2,9024E-11	5
**TRAF3IP3**	1,0845E-15	0,21968002	2,9169E-11	5
**NECAP2**	1,2673E-15	0,30591486	3,4087E-11	5
**ZNF467**	1,6863E-15	1,17250156	4,5357E-11	5
**YPEL3**	1,7052E-15	0,21252495	4,5864E-11	5
**STAT3**	1,7627E-15	0,37752969	4,7412E-11	5
**LBH**	2,0936E-15	0,29934159	5,6311E-11	5
**ZWINT**	2,1882E-15	1,9114925	5,8855E-11	5
**AEBP1**	2,3097E-15	2,43859125	6,2125E-11	5
**NCK2**	2,4085E-15	0,27802908	6,4782E-11	5
**SLC12A6**	3,4971E-15	0,81822704	9,4062E-11	5
**YPEL2**	4,1541E-15	0,70065653	1,1173E-10	5
**RASA3**	4,539E-15	0,32472077	1,2208E-10	5
**SCML4**	5,3221E-15	0,33168527	1,4315E-10	5
**HIST1H1D**	6,4723E-15	0,27206328	1,7409E-10	5
**SCGB3A1**	6,6461E-15	1,78943549	1,7876E-10	5
**CRTC3**	7,1091E-15	0,62486184	1,9121E-10	5
**HOOK2**	7,6348E-15	0,55900948	2,0535E-10	5
**MORC3**	7,9321E-15	0,46285547	2,1335E-10	5
**STT3B**	8,5845E-15	0,37180126	2,309E-10	5
**SLC39A10**	8,6385E-15	0,57641103	2,3235E-10	5
**TMPO**	1,1418E-14	0,37252047	3,0712E-10	5
**RCAN3**	1,1546E-14	0,30829895	3,1054E-10	5
**STRADB**	1,2009E-14	0,72102615	3,23E-10	5
**RP11-475O6.1**	1,3596E-14	1,63924901	3,6568E-10	5
**RP11-51J9.5**	1,5464E-14	0,54354147	4,1593E-10	5
**DIP2B**	1,7064E-14	0,81516565	4,5897E-10	5
**LINC00243**	1,8456E-14	0,88056339	4,9641E-10	5
**GPR15**	2,458E-14	1,16159087	6,6114E-10	5
**RILPL2**	2,4791E-14	0,42758927	6,6679E-10	5

**ARHGAP9**	2,5556E-14	0,36333589	6,8738E-10	5
**GRK5**	2,85E-14	0,76092074	7,6656E-10	5
**KIAA1551**	3,7096E-14	0,31263686	9,9778E-10	5
**TUBA1A**	4,0152E-14	0,26619902	1,08E-09	5
**RPS29**	4,2995E-14	0,10972811	1,1564E-09	5
**FUS**	4,3245E-14	0,22160178	1,1632E-09	5
**HMGN1**	4,78E-14	0,20274064	1,2857E-09	5
**CDK5R1**	4,8013E-14	1,29711399	1,2914E-09	5
**CLN5**	4,9702E-14	0,61789317	1,3368E-09	5
**JUND**	5,0912E-14	0,16768355	1,3694E-09	5
**JAK3**	6,5352E-14	0,49214047	1,7578E-09	5
**TLR1**	6,7785E-14	1,19430887	1,8232E-09	5
**CRLF3**	6,9013E-14	0,51835898	1,8562E-09	5
**GGT7**	7,727E-14	0,87386936	2,0783E-09	5
**GAS6**	9,4088E-14	2,16512045	2,5307E-09	5
**RP11-348F1.3**	9,7449E-14	1,64972589	2,6211E-09	5
**PPDPF**	1,0682E-13	0,13269901	2,8732E-09	5
**CTLA4**	1,1666E-13	1,14529056	3,1378E-09	5
**WWP1**	1,3263E-13	0,46234573	3,5673E-09	5
**TCP11L2**	1,3802E-13	0,48211245	3,7123E-09	5
**DGCR6L**	1,4249E-13	0,3179686	3,8327E-09	5
**CTD-3184A7.4**	1,5229E-13	0,37069118	4,0962E-09	5
**RMND5B**	1,562E-13	0,77173248	4,2012E-09	5
**NAP1L3**	1,6072E-13	1,39856726	4,3228E-09	5
**CTC1**	2,1063E-13	0,54487226	5,6654E-09	5
**PKIA**	2,1922E-13	0,73069939	5,8963E-09	5
**MANBA**	2,2514E-13	0,59850944	6,0556E-09	5
**SLC22A23**	2,2559E-13	1,90705165	6,0676E-09	5
**LINS1**	2,4935E-13	0,56569846	6,7069E-09	5
**ASH1L**	2,4956E-13	0,40916496	6,7124E-09	5
**ZFAS1**	2,6355E-13	0,17488516	7,0887E-09	5
**RP3-492J12.2**	2,6644E-13	1,21648575	7,1663E-09	5
**PRKAR1B**	3,047E-13	0,81075213	8,1956E-09	5
**PRKCB**	3,3096E-13	0,35109979	8,9017E-09	5
**APOLD1**	3,332E-13	0,85357229	8,9622E-09	5
**SAMHD1**	3,4019E-13	0,24920493	9,1501E-09	5
**SEPT10**	3,5458E-13	2,3759819	9,5371E-09	5
**P2RY10**	3,7338E-13	0,59638485	1,0043E-08	5
**SIRPG**	3,9672E-13	0,36431606	1,0671E-08	5
**FAM160B1**	4,3054E-13	0,68907574	1,158E-08	5
**ZMYM2**	4,7773E-13	0,47224768	1,285E-08	5

**MAGEF1**	5,002E-13	0,69182366	1,3454E-08	5
**RAC2**	5,2013E-13	0,14172724	1,399E-08	5
**ZDHHC15**	5,3353E-13	2,27594034	1,435E-08	5
**KLF7**	5,3967E-13	1,03402147	1,4516E-08	5
**SECISBP2**	6,1429E-13	0,35722475	1,6523E-08	5
**TRAK2**	6,1898E-13	0,57128197	1,6649E-08	5
**ZNF107**	6,8197E-13	0,52754498	1,8343E-08	5
**ZC3H7A**	6,9734E-13	0,46880142	1,8756E-08	5
**NCOA3**	7,8228E-13	0,55520225	2,1041E-08	5
**HIST1H4C**	8,2661E-13	0,21551845	2,2233E-08	5
**ZC3H12D**	8,7878E-13	0,86049802	2,3637E-08	5
**EPC1**	9,3356E-13	0,22511374	2,511E-08	5
**ELMO1**	9,543E-13	0,41083603	2,5668E-08	5
**SLC8B1**	1,178E-12	0,86983306	3,1686E-08	5
**MAP3K1**	1,245E-12	0,48893203	3,3487E-08	5
**ARHGEF3**	1,4099E-12	0,37060351	3,7923E-08	5
**USP15**	1,4194E-12	0,27559547	3,8179E-08	5
**CPSF6**	1,4357E-12	0,37349127	3,8617E-08	5
**KCNK12**	1,4529E-12	1,13787158	3,9079E-08	5
**DENND1C**	1,6934E-12	0,42107375	4,5547E-08	5
**USP3**	1,7034E-12	0,29431486	4,5817E-08	5
**TIMP2**	1,7932E-12	2,05066246	4,8232E-08	5
**GNAS**	1,8943E-12	0,14148923	5,0951E-08	5
**ZNF609**	2,1432E-12	0,61633748	5,7645E-08	5
**KLF2**	2,2516E-12	0,1265739	6,056E-08	5
**CASK**	2,3612E-12	0,36961823	6,351E-08	5
**SOCS6**	2,4678E-12	2,62450746	6,6375E-08	5
**DDX17**	2,506E-12	0,25417258	6,7404E-08	5
**FBXO32**	2,6305E-12	0,61489275	7,0752E-08	5
**LSR**	2,6693E-12	0,45289668	7,1795E-08	5
**ERCC6L2**	2,7783E-12	0,67242325	7,4727E-08	5
**HIST1H2AG**	2,7952E-12	0,50880079	7,5181E-08	5
**BIRC3**	3,127E-12	0,41569786	8,4107E-08	5
**FAM19A2**	3,2531E-12	1,32396229	8,7499E-08	5
**NLRP1**	3,2564E-12	0,41611315	8,7586E-08	5
**SLC35G1**	3,5919E-12	1,16284132	9,6613E-08	5
**ADSL**	3,6106E-12	0,32483323	9,7115E-08	5
**SLC25A25**	3,8844E-12	0,60473025	1,0448E-07	5
**ELF1**	4,0691E-12	0,25232889	1,0945E-07	5
**ATP1A1**	4,1584E-12	0,37168166	1,1185E-07	5
**CA5B**	4,1646E-12	0,33102615	1,1201E-07	5

**SH3BP5**	4,3232E-12	0,30444501	1,1628E-07	5
**SCAPER**	4,3326E-12	0,50559027	1,1653E-07	5
**CCNI**	4,3481E-12	0,13480443	1,1695E-07	5
**FCHSD2**	4,4879E-12	0,62562348	1,2071E-07	5
**ACP5**	4,7421E-12	0,73915271	1,2755E-07	5
**PRDX6**	5,0421E-12	0,2564489	1,3562E-07	5
**KLF12**	5,1585E-12	0,31554075	1,3875E-07	5
**TRAF1**	5,3395E-12	0,58417019	1,4362E-07	5
**MBD5**	5,765E-12	0,43939684	1,5506E-07	5
**SNX29**	6,1733E-12	0,67018962	1,6604E-07	5
**UPF3A**	6,7071E-12	0,33994283	1,804E-07	5
**KRAS**	7,4894E-12	0,3078636	2,0144E-07	5
**KCNQ1**	8,2808E-12	0,69153906	2,2273E-07	5
**ITGA4**	8,3934E-12	0,2575421	2,2576E-07	5
**PLXNA4**	9,3236E-12	1,85597145	2,5078E-07	5
**CNKSR2**	9,935E-12	1,96413111	2,6722E-07	5
**UBL3**	1,232E-11	0,39157452	3,3136E-07	5
**CIB1**	1,2716E-11	0,20775892	3,4202E-07	5
**ADGRE1**	1,749E-11	1,2129264	4,7043E-07	5
**KIAA2022**	2,2413E-11	2,3620414	6,0283E-07	5
**ANP32B**	2,2689E-11	0,16267552	6,1026E-07	5
**CDR2**	2,5207E-11	0,4236535	6,7798E-07	5
**IGF1R**	2,5261E-11	0,94728077	6,7944E-07	5
**GRINA**	2,5312E-11	0,46135432	6,8083E-07	5
**TENM1**	2,5391E-11	1,3411868	6,8295E-07	5
**TPP2**	2,5609E-11	0,39062327	6,888E-07	5
**NAB1**	2,7049E-11	1,16150657	7,2753E-07	5
**RP11-856F16.2**	2,7109E-11	1,4118052	7,2916E-07	5
**U2AF1L4**	2,8951E-11	0,39013764	7,7869E-07	5
**HIVEP1**	3,074E-11	0,69205033	8,268E-07	5
**PCBP3**	3,1018E-11	2,07084951	8,3428E-07	5
**SDCBP**	3,1419E-11	0,34286587	8,4508E-07	5
**AKT3**	3,6802E-11	0,31778056	9,8986E-07	5
**RP11-243J16.7**	4,0851E-11	1,37954193	1,0988E-06	5
**C2orf40**	4,4077E-11	1,30527998	1,1855E-06	5
**RBBP6**	4,7028E-11	0,25099181	1,2649E-06	5
**HDDC2**	4,7663E-11	0,36598588	1,282E-06	5
**CRIP2**	5,5325E-11	0,43093893	1,4881E-06	5
**COMMD6**	5,5493E-11	0,11135379	1,4926E-06	5
**IL21R**	5,6285E-11	0,76726376	1,5139E-06	5
**UBE2D2**	5,6397E-11	0,15800782	1,5169E-06	5

**SH3KBP1**	5,9971E-11	0,27727784	1,6131E-06	5
**DPEP2**	6,1796E-11	0,48249987	1,6621E-06	5
**ST6GALNAC1**	7,5213E-11	1,02561982	2,023E-06	5
**H3F3B**	7,8892E-11	0,12728817	2,122E-06	5
**LRRN3**	8,1517E-11	0,87250755	2,1926E-06	5
**ZNF442**	8,453E-11	1,88287993	2,2736E-06	5
**GRAP**	8,8773E-11	0,72839616	2,3877E-06	5
**ANKRD12**	9,1296E-11	0,1990007	2,4556E-06	5
**WNT10A**	9,863E-11	1,22023433	2,6528E-06	5
**CDC37L1**	9,8747E-11	0,53572519	2,656E-06	5
**CEP170**	1,0302E-10	0,78763171	2,7709E-06	5
**APBB1**	1,0548E-10	0,50398568	2,837E-06	5
**PHF3**	1,1182E-10	0,30423893	3,0077E-06	5
**NBPF15**	1,2089E-10	0,52354746	3,2516E-06	5
**RP11-342D11.3**	1,3343E-10	0,84643754	3,5888E-06	5
**PLEKHB1**	1,3718E-10	0,49909106	3,6897E-06	5
**SLC40A1**	1,5115E-10	0,46375479	4,0655E-06	5
**SYNGR2**	1,5977E-10	0,27066004	4,2974E-06	5
**N4BP2L2**	1,598E-10	0,23079226	4,2982E-06	5
**CTSL**	1,6416E-10	1,69034521	4,4154E-06	5
**DAZAP2**	1,6635E-10	0,1340202	4,4742E-06	5
**CLK1**	1,9217E-10	0,39151713	5,1687E-06	5
**STMN3**	2,124E-10	0,26509141	5,713E-06	5
**ZFC3H1**	2,2219E-10	0,3829129	5,9762E-06	5
**ARID5A**	2,3486E-10	0,39634696	6,3169E-06	5
**TMEM161B-AS1**	2,8752E-10	0,57563783	7,7335E-06	5
**EVI5**	2,9029E-10	2,01688873	7,8079E-06	5
**DENND2D**	3,2031E-10	0,27702622	8,6154E-06	5
**FAM200B**	3,3117E-10	0,3634629	8,9073E-06	5
**ZNF844**	3,3359E-10	1,11182231	8,9727E-06	5
**ABCC1**	3,4699E-10	0,64499555	9,3331E-06	5
**PPP2R5C**	3,5251E-10	0,25755701	9,4815E-06	5
**TGOLN2**	3,6337E-10	0,28412169	9,7737E-06	5
**PDCD4-AS1**	3,6695E-10	0,50385906	9,8698E-06	5
**ADAMTSL5**	3,8688E-10	1,42193432	1,0406E-05	5
**LCK**	3,9344E-10	0,14669742	1,0582E-05	5
**NUCKS1**	4,1976E-10	0,23751203	1,129E-05	5
**ACVR1**	4,2104E-10	0,67973022	1,1325E-05	5
**PIM2**	4,8461E-10	0,27021835	1,3035E-05	5
**PSD**	5,3293E-10	1,43263808	1,4334E-05	5
**COA1**	5,6643E-10	0,46456152	1,5235E-05	5

**KLHL3**	5,7522E-10	1,56158395	1,5472E-05	5
**HIST1H2BD**	5,851E-10	1,0962883	1,5737E-05	5
**ZNF439**	6,0918E-10	1,08532684	1,6385E-05	5
**NUAK2**	6,138E-10	0,88293201	1,6509E-05	5
**DMXL1**	6,2986E-10	0,45014651	1,6941E-05	5
**PARK2**	6,447E-10	0,78519035	1,7341E-05	5
**SEPW1**	6,9187E-10	0,16592359	1,8609E-05	5
**SUCLG2-AS1**	6,9284E-10	1,2794819	1,8635E-05	5
**ZNF575**	8,7495E-10	0,6970906	2,3534E-05	5
**SUCLG2**	8,7779E-10	0,32393593	2,361E-05	5
**ICA1**	8,9761E-10	1,51979911	2,4143E-05	5
**GSTM3**	9,1088E-10	0,89291301	2,45E-05	5
**JUN**	9,2398E-10	0,2544832	2,4852E-05	5
**NPC2**	9,4149E-10	0,27599635	2,5323E-05	5
**RP11-161M6.2**	9,6201E-10	0,83934456	2,5875E-05	5
**ORMDL3**	9,974E-10	0,44705502	2,6827E-05	5
**ZNF682**	1,0136E-09	0,89807486	2,7262E-05	5
**ZNF862**	1,0322E-09	0,40856568	2,7764E-05	5
**FANCI**	1,0457E-09	0,97768867	2,8126E-05	5
**ZNF518B**	1,0645E-09	0,71633117	2,8631E-05	5
**NIN**	1,1167E-09	0,41289723	3,0035E-05	5
**ZC3H6**	1,1482E-09	0,41596056	3,0882E-05	5
**DZIP3**	1,2344E-09	0,79747798	3,3201E-05	5
**DCXR**	1,2978E-09	0,25912018	3,4906E-05	5
**MDM4**	1,3394E-09	0,42515726	3,6026E-05	5
**RBM39**	1,369E-09	0,18135975	3,6823E-05	5
**EVL**	1,3894E-09	0,12572741	3,7372E-05	5
**CYTH1**	1,4237E-09	0,21447551	3,8294E-05	5
**KLF9**	1,4313E-09	0,35210475	3,8496E-05	5
**MYEF2**	1,4963E-09	1,25774732	4,0247E-05	5
**TRPS1**	1,6049E-09	0,57558602	4,3166E-05	5
**AC103563.8**	1,7476E-09	0,69875641	4,7004E-05	5
**NMRK1**	1,8812E-09	0,34141997	5,0599E-05	5
**CIRBP**	1,8823E-09	0,12836216	5,0628E-05	5
**YTHDC1**	2,1187E-09	0,27160235	5,6988E-05	5
**SOCS3**	2,1859E-09	0,50893216	5,8795E-05	5
**TBL1X**	2,2033E-09	0,51314678	5,9262E-05	5
**F5**	2,2931E-09	1,7264921	6,1677E-05	5
**RERE**	2,3503E-09	0,42506059	6,3216E-05	5
**PNN**	2,5554E-09	0,26399453	6,8733E-05	5
**SORL1**	2,5624E-09	0,31453528	6,892E-05	5

**FOXO1**	2,5692E-09	0,32734185	6,9104E-05	5
**CSDE1**	2,5745E-09	0,19719288	6,9246E-05	5
**JPX**	2,616E-09	0,39802925	7,0362E-05	5
**TXNIP**	2,6595E-09	0,11458027	7,1533E-05	5
**SERPINB6**	2,684E-09	0,37795395	7,2191E-05	5
**TMEM243**	2,7202E-09	0,25586222	7,3166E-05	5
**RICTOR**	2,7677E-09	0,37349154	7,4443E-05	5
**KCNN4**	2,8226E-09	0,71012323	7,5918E-05	5
**NIPAL3**	2,8609E-09	0,46497316	7,6951E-05	5
**EDEM3**	2,9869E-09	0,47380947	8,0338E-05	5
**LRRC7**	3,2297E-09	2,18844594	8,6868E-05	5
**SRSF10**	3,4016E-09	0,21664546	9,1493E-05	5
**ADI1**	3,4447E-09	0,32687141	9,2652E-05	5
**HIP1R**	3,495E-09	0,75067152	9,4005E-05	5
**HMHA1**	3,588E-09	0,19417193	9,6506E-05	5
**VWA5A**	3,9327E-09	1,40277686	0,00010578	5
**HIST1H2AC**	4,0877E-09	0,42661633	0,00010995	5
**PREPL**	4,1171E-09	0,45852608	0,00011074	5
**SULT1A1**	4,147E-09	0,60371021	0,00011154	5
**HNRNPDL**	4,2558E-09	0,11898688	0,00011447	5
**CTB-133G6.1**	4,5656E-09	0,35972376	0,0001228	5
**SRSF11**	5,3635E-09	0,22091374	0,00014426	5
**HMGB2**	5,6729E-09	0,24474492	0,00015258	5
**RPIA**	5,8862E-09	0,36010378	0,00015832	5
**SLC7A6**	6,1795E-09	0,4195118	0,00016621	5
**OPTN**	6,3017E-09	0,24364607	0,0001695	5
**ANKH**	6,3328E-09	0,51703441	0,00017033	5
**ASB16-AS1**	6,836E-09	0,57452676	0,00018387	5
**NKTR**	7,1386E-09	0,26770556	0,00019201	5
**BCL2A1**	7,3756E-09	0,43752204	0,00019838	5
**ZBTB10**	7,5548E-09	0,54822138	0,0002032	5
**NMNAT3**	7,5808E-09	1,60310447	0,0002039	5
**PYHIN1**	7,6202E-09	0,31048403	0,00020496	5
**BLVRA**	8,0698E-09	0,44684776	0,00021705	5
**RGCC**	8,1216E-09	0,19689882	0,00021845	5
**KANSL1**	8,3597E-09	0,350456	0,00022485	5
**TOM1**	8,4053E-09	0,40970236	0,00022608	5
**LAMTOR4**	8,7102E-09	0,14657975	0,00023428	5
**ASAP1**	8,74E-09	0,48771648	0,00023508	5
**SMARCA2**	8,9652E-09	0,30028341	0,00024114	5
**ZNF141**	9,1199E-09	0,55895981	0,0002453	5

**IMPG2**	9,1461E-09	1,31460644	0,000246	5
**SNHG8**	9,2698E-09	0,15803391	0,00024933	5
**AGMAT**	1,0488E-08	1,00800001	0,00028211	5
**CTD-2020K17.1**	1,0523E-08	0,3103384	0,00028303	5
**AGTPBP1**	1,0776E-08	0,35597808	0,00028985	5
**PPM1M**	1,079E-08	0,37203369	0,00029022	5
**GPATCH8**	1,1508E-08	0,34627017	0,00030954	5
**CTD-2015H6.3**	1,1595E-08	1,76577225	0,00031186	5
**CD4**	1,1711E-08	0,24035271	0,000315	5
**LYRM9**	1,174E-08	0,60485464	0,00031577	5
**KIF2A**	1,2775E-08	0,20043058	0,00034361	5
**PTRHD1**	1,2831E-08	0,33989255	0,00034513	5
**DCP2**	1,2872E-08	0,36262303	0,00034621	5
**AHSA2**	1,3437E-08	0,39984297	0,00036142	5
**ACSS2**	1,4518E-08	0,85087553	0,0003905	5
**ZCCHC7**	1,4578E-08	0,3151528	0,00039211	5
**OBSCN**	1,4797E-08	0,81810473	0,000398	5
**MMP28**	1,4839E-08	1,82551768	0,00039913	5
**LRP6**	1,5162E-08	1,67502079	0,00040782	5
**CDKAL1**	1,5464E-08	0,42204694	0,00041594	5
**ZNF483**	1,5479E-08	0,7131631	0,00041634	5
**ARID1B**	1,6945E-08	0,33063876	0,00045578	5
**SEPT1**	1,7292E-08	0,1744263	0,0004651	5
**TRIM73**	1,7315E-08	0,58869001	0,00046571	5
**CD46**	1,749E-08	0,24506388	0,00047043	5
**RNF144A**	1,755E-08	0,5922938	0,00047204	5
**RYK**	1,7766E-08	0,38029276	0,00047785	5
**CYP4V2**	1,7847E-08	0,41566828	0,00048004	5
**ST13**	1,8206E-08	0,16852969	0,00048969	5
**RPL23**	1,8351E-08	0,11938165	0,00049358	5
**SEPN1**	1,9109E-08	0,84525861	0,00051397	5
**CCDC14**	1,9258E-08	0,61009732	0,00051797	5
**BRI3**	1,928E-08	0,25077943	0,00051857	5
**PTPN6**	2,0293E-08	0,20874923	0,00054582	5
**PTPRN2**	2,154E-08	1,04195327	0,00057936	5
**CYFIP2**	2,2013E-08	0,24749016	0,00059207	5
**ZNF292**	2,2128E-08	0,28827027	0,00059517	5
**HAUS3**	2,2216E-08	0,45107703	0,00059755	5
**R3HDM4**	2,3295E-08	0,18983469	0,00062656	5
**RPL13A**	2,3536E-08	0,10503659	0,00063304	5
**HIST1H4F**	2,377E-08	0,54264644	0,00063935	5

**TMEM41B**	2,3967E-08	0,51564871	0,00064465	5
**ZNF580**	2,4021E-08	0,43982679	0,00064609	5
**ETS1**	2,6215E-08	0,14809446	0,00070511	5
**CELF2**	2,8338E-08	0,18624036	0,0007622	5
**NAP1L1**	2,8498E-08	0,1642494	0,0007665	5
**HDAC1**	2,8734E-08	0,25309746	0,00077287	5
**HMGA1**	2,8816E-08	0,31454374	0,00077506	5
**ARHGAP4**	2,8866E-08	0,24716619	0,00077641	5
**RP11-712B9.2**	3,1147E-08	0,72050672	0,00083776	5
**DDX18**	3,16E-08	0,20373149	0,00084995	5
**ZRANB2**	3,1776E-08	0,24881697	0,00085468	5
**HIST1H2BB**	3,3182E-08	0,68881032	0,00089251	5
**TMC8**	3,421E-08	0,21731135	0,00092016	5
**PRKACB**	3,571E-08	0,30949798	0,0009605	5
**TLE2**	3,6872E-08	0,67148303	0,00099175	5
**PIAS1**	3,9935E-08	0,29114472	0,00107413	5
**HECA**	4,0026E-08	0,30273397	0,00107657	5
**CENPC**	4,1349E-08	0,25526284	0,00111217	5
**CHURC1**	4,2524E-08	0,27441571	0,00114376	5
**ZEB1**	4,6597E-08	0,31724875	0,00125333	5
**RBL1**	4,7066E-08	0,48754318	0,00126593	5
**C6orf48**	4,9598E-08	0,32300979	0,00133404	5
**HPCAL4**	5,0105E-08	0,84040007	0,00134767	5
**RP1-153P14.8**	5,1899E-08	0,88789472	0,00139594	5
**YPEL5**	5,2586E-08	0,2445096	0,0014144	5
**PDK1**	5,2757E-08	0,71352638	0,001419	5
**UBE2E1**	5,5045E-08	0,34023246	0,00148053	5
**ATP10A**	5,8005E-08	0,54807546	0,00156015	5
**CD6**	5,9159E-08	0,17207366	0,00159121	5
**RARRES3**	6,0459E-08	0,12685947	0,00162616	5
**HNRNPH3**	6,1512E-08	0,17293245	0,00165448	5
**C11orf31**	6,1581E-08	0,15955083	0,00165635	5
**RNPC3**	6,2385E-08	0,36179833	0,00167797	5
**FTX**	6,3072E-08	0,41382776	0,00169646	5
**RABGAP1**	6,4184E-08	0,51998875	0,00172635	5
**CRYL1**	6,4432E-08	0,46477058	0,00173304	5
**CEP135**	6,626E-08	0,48206745	0,00178218	5
**FAM120B**	7,2228E-08	0,35314962	0,00194272	5
**CFP**	7,2376E-08	0,60355687	0,0019467	5
**ARMCX4**	7,5795E-08	0,95876136	0,00203866	5
**ZNF638**	7,5838E-08	0,30637213	0,00203981	5

**ITFG1**	7,8604E-08	0,30926596	0,00211422	5
**LYPD3**	7,8725E-08	1,11420777	0,00211747	5
**MTUS1**	8,0511E-08	1,43578262	0,0021655	5
**MVP**	8,3098E-08	0,24885243	0,00223509	5
**ZNF10**	8,3188E-08	0,60821346	0,00223751	5
**IKZF1**	8,3631E-08	0,16136775	0,00224942	5
**RAD51B**	8,3928E-08	0,53351694	0,00225741	5
**CTB-4E7.1**	8,6566E-08	0,66945831	0,00232838	5
**ZNF414**	8,7945E-08	0,37578535	0,00236545	5
**ORMDL1**	8,8576E-08	0,16895012	0,00238243	5
**FAM60A**	8,8598E-08	0,37039578	0,00238303	5
**RTN4**	9,1575E-08	0,2785579	0,00246309	5
**CREBZF**	9,2454E-08	0,36639866	0,00248673	5
**RGL1**	9,7405E-08	1,41843044	0,00261989	5
**CASP8AP2**	9,862E-08	0,38507567	0,00265259	5
**HNRNPA2B1**	1,0044E-07	0,10355009	0,00270162	5
**RP11-11N9.4**	1,0155E-07	1,2692447	0,00273131	5
**CELA1**	1,0267E-07	1,07545431	0,00276155	5
**SEC62**	1,1439E-07	0,20239393	0,00307684	5
**ARPC5**	1,1442E-07	0,18362173	0,00307765	5
**CDC42SE2**	1,1587E-07	0,21485168	0,00311646	5
**THOC3**	1,1698E-07	0,27699421	0,00314654	5
**RFX3-AS1**	1,2326E-07	0,69375726	0,00331524	5
**CYTIP**	1,2455E-07	0,15849527	0,00335005	5
**CNOT8**	1,2953E-07	0,32423941	0,00348387	5
**ALKBH7**	1,3043E-07	0,18976272	0,0035081	5
**LUC7L3**	1,3295E-07	0,24375817	0,00357587	5
**MAPK3**	1,3506E-07	0,37153328	0,00363261	5
**HIST1H2BF**	1,3739E-07	0,77413784	0,00369531	5
**RAB37**	1,4454E-07	0,49073176	0,00388756	5
**TP53INP1**	1,4771E-07	0,57124938	0,00397292	5
**CCNT1**	1,5486E-07	0,4227444	0,00416535	5
**ISM1**	1,5821E-07	0,75027747	0,00425526	5
**PKIA-AS1**	1,6501E-07	1,61092808	0,00443821	5
**H1F0**	1,6881E-07	0,82683667	0,00454054	5
**NBPF9**	1,7003E-07	0,87779796	0,00457332	5
**MAD2L1**	1,7165E-07	0,57187752	0,00461678	5
**IFI16**	1,7501E-07	0,25767827	0,00470727	5
**SNX18**	1,8398E-07	0,82617634	0,00494843	5
**GABPB1**	1,8876E-07	0,2756736	0,00507706	5
**GNB5**	1,9751E-07	0,41894744	0,00531232	5

**RP11-902B17.1**	2,0262E-07	0,63753625	0,00544982	5
**ATG9B**	2,0475E-07	0,85209373	0,00550716	5
**THUMPD3-AS1**	2,0938E-07	0,3520217	0,00563163	5
**CSGALNACT2**	2,1012E-07	0,33534796	0,0056515	5
**TECR**	2,1755E-07	0,15642947	0,00585156	5
**LINC01259**	2,1998E-07	0,81540161	0,00591686	5
**XIST**	2,292E-07	0,51260278	0,00616479	5
**NDUFC1**	2,3907E-07	0,18828673	0,00643019	5
**PPP2R3B**	2,3997E-07	0,59110487	0,00645447	5
**TMEM106B**	2,4109E-07	0,28326012	0,0064846	5
**TNFAIP8**	2,4851E-07	0,14702673	0,00668417	5
**PNRC2**	2,4962E-07	0,19951963	0,00671401	5
**HERC3**	2,533E-07	0,52612535	0,00681295	5
**HNRNPA0**	2,553E-07	0,12548158	0,00686669	5
**CDCA7**	2,5962E-07	1,29608708	0,00698291	5
**CTC-343N3.1**	2,7069E-07	0,86914433	0,0072807	5
**NDUFV2-AS1**	2,7283E-07	0,55481641	0,00733837	5
**ZNF506**	2,8325E-07	0,4785677	0,00761859	5
**INF2**	2,8482E-07	0,64729842	0,00766093	5
**MAN1C1**	2,9378E-07	0,9619947	0,00790178	5
**RP1-179N16.6**	3,0003E-07	0,99128393	0,00806995	5
**ZNF431**	3,0371E-07	0,37832912	0,00816889	5
**MTERF4**	3,0431E-07	0,27474627	0,0081849	5
**MIATNB**	3,1029E-07	0,46974512	0,0083459	5
**SENP7**	3,12E-07	0,3151373	0,00839193	5
**CHI3L2**	3,2155E-07	1,03814664	0,00864881	5
**LRRC8B**	3,2156E-07	0,81479098	0,008649	5
**ANAPC16**	3,2183E-07	0,12428905	0,00865619	5
**KIF9-AS1**	3,3539E-07	1,00069917	0,00902111	5
**FAM45A**	3,4663E-07	0,41588424	0,00932319	5
**SCLT1**	3,5465E-07	0,47315549	0,00953899	5
**CHD3**	3,5642E-07	0,31488892	0,00958666	5
**AC093818.1**	3,5911E-07	0,72320906	0,00965887	5
**SPATA13**	3,626E-07	0,59616266	0,00975274	5
**MT1F**	3,6652E-07	0,49262429	0,00985833	5
**RABEP1**	3,7883E-07	0,40809337	0,0101895	5
**TRBV1**	3,8138E-07	1,70410637	0,01025796	5
**DHRS4L2**	3,8174E-07	0,50096051	0,01026779	5
**NOSIP**	3,854E-07	0,12586002	0,01036602	5
**IMMP2L**	3,9917E-07	0,33621044	0,01073659	5
**ZNF563**	4,0159E-07	0,6096355	0,01080146	5

**GNAQ**	4,0209E-07	0,48209606	0,01081488	5
**HMGN3**	4,0416E-07	0,20443471	0,0108708	5
**ISCU**	4,1988E-07	0,14729756	0,01129344	5
**AMMECR1**	4,2668E-07	0,4360269	0,01147643	5
**CNOT7**	4,2756E-07	0,22512303	0,01149998	5
**SVBP**	4,3339E-07	0,29497644	0,0116569	5
**ZNF639**	4,4133E-07	0,26836866	0,01187047	5
**RASAL3**	4,4351E-07	0,22586209	0,01192919	5
**SP3**	4,438E-07	0,28513317	0,01193677	5
**RHOB**	4,5226E-07	0,53247588	0,01216457	5
**TTN**	4,6922E-07	1,22792375	0,01262057	5
**NDUFA4**	4,9354E-07	0,10332354	0,01327472	5
**RFX3**	4,9541E-07	0,40575662	0,01332501	5
**PJA2**	5,0694E-07	0,30360222	0,01363503	5
**NSUN5**	5,1175E-07	0,31560044	0,01376453	5
**USP51**	5,1402E-07	1,50990521	0,01382572	5
**PUM2**	5,1714E-07	0,26672278	0,01390947	5
**PPP2R1A**	5,2097E-07	0,17575654	0,0140124	5
**ALS2CL**	5,2724E-07	1,00131054	0,01418114	5
**PTBP2**	5,3364E-07	0,62048133	0,01435324	5
**SDCCAG8**	5,4459E-07	0,43010342	0,01464778	5
**SHPRH**	5,4564E-07	0,42169502	0,01467605	5
**LMBRD1**	5,5973E-07	0,29581777	0,01505514	5
**PHC3**	5,6155E-07	0,26100921	0,01510404	5
**USP3-AS1**	5,6413E-07	0,55971927	0,01517344	5
**CECR1**	6,1839E-07	0,3832493	0,01663284	5
**AP001055.6**	6,2809E-07	1,32833587	0,01689368	5
**SELM**	6,2864E-07	0,2455529	0,01690859	5
**NDUFB9**	6,395E-07	0,12740772	0,01720052	5
**AKAP13**	6,5507E-07	0,22833332	0,01761943	5
**ATP9B**	6,5786E-07	0,31037197	0,01769456	5
**FABP5**	6,6098E-07	0,73680031	0,01777837	5
**ZYX**	6,7214E-07	0,18694489	0,01807842	5
**CCSER1**	6,8195E-07	1,44183824	0,01834235	5
**PHYKPL**	7,1301E-07	0,29991795	0,01917791	5
**RP13-977J11.2**	7,2045E-07	0,41781973	0,01937793	5
**RP11-66N11.8**	7,3611E-07	0,75175146	0,01979919	5
**ARFRP1**	7,6216E-07	0,29892468	0,02049987	5
**NUP43**	7,7994E-07	0,73224499	0,02097816	5
**ZNF451**	7,8402E-07	0,39653338	0,02108768	5
**FCGRT**	7,9815E-07	0,43499207	0,02146787	5

**CCDC7.1**	8,5554E-07	0,71881012	0,02301151	5
**LENG8**	8,5755E-07	0,27241095	0,02306564	5
**TRADD**	8,6663E-07	0,14955352	0,0233097	5
**DCBLD2**	8,9044E-07	1,65092874	0,02395024	5
**CNN2**	8,9845E-07	0,1184162	0,02416552	5
**CTBS**	9,2622E-07	0,41183238	0,02491259	5
**CTC-523E23.11**	9,2821E-07	0,26218004	0,02496617	5
**TNRC6B**	9,3111E-07	0,18963751	0,02504407	5
**RB1**	9,8971E-07	0,30946893	0,02662014	5
**FBXL3**	1,0089E-06	0,35311709	0,02713606	5
**RASGRP1**	1,0162E-06	0,24285445	0,02733142	5
**KIF22**	1,0417E-06	0,29086348	0,02801741	5
**NUDT14**	1,0419E-06	0,63069792	0,02802454	5
**GCA**	1,0521E-06	0,59907824	0,02829854	5
**TBC1D2B**	1,0803E-06	0,45547357	0,02905587	5
**IL16**	1,0961E-06	0,19784426	0,02948269	5
**C20orf196**	1,1209E-06	0,40335878	0,03014952	5
**PRPSAP2**	1,1218E-06	0,36172067	0,0301733	5
**LRMP**	1,133E-06	0,54737816	0,03047553	5
**TBL1XR1**	1,1405E-06	0,34329306	0,03067602	5
**SDR39U1**	1,1537E-06	0,22247455	0,03103233	5
**TSPYL4**	1,1734E-06	0,43999432	0,03156016	5
**BET1L**	1,1896E-06	0,31026122	0,03199778	5
**RBM43**	1,1938E-06	0,5288904	0,03210831	5
**CLEC11A**	1,2395E-06	1,71284038	0,03333811	5
**PPP6R3**	1,246E-06	0,32025729	0,03351341	5
**VPS13D**	1,2758E-06	0,35579394	0,03431644	5
**WBSCR16**	1,3307E-06	0,46029341	0,03579061	5
**KDM3B**	1,3419E-06	0,40711089	0,03609392	5
**FNBP4**	1,363E-06	0,21437743	0,03665984	5
**MYL6B**	1,388E-06	0,82180263	0,03733405	5
**PCNX**	1,4408E-06	0,25721461	0,03875336	5
**LAX1**	1,4416E-06	0,59483124	0,03877558	5
**PNPLA7**	1,4518E-06	1,20223925	0,03904823	5
**RC3H2**	1,4816E-06	0,34813381	0,03984997	5
**SPG7**	1,4973E-06	0,29216028	0,04027335	5
**BAZ2B**	1,5009E-06	0,50645663	0,04036903	5
**CHRM3**	1,5514E-06	0,8532312	0,04172861	5
**NAA30**	1,5553E-06	0,49752197	0,0418335	5
**KDM6A**	1,556E-06	0,37472169	0,04185263	5
**HPCAL1**	1,5624E-06	0,23276941	0,04202256	5

**ANP32A**	1,5683E-06	0,19164631	0,04218335	5
**GRK6**	1,5908E-06	0,17622854	0,04278885	5
**CHRM3-AS2**	1,6169E-06	0,46550473	0,04348885	5
**CAMKMT**	1,6183E-06	0,31063667	0,04352692	5
**ZNF253**	1,6526E-06	0,61401486	0,04444942	5
**RNF19A**	1,7189E-06	0,31226321	0,04623316	5
**AHI1**	1,7353E-06	0,69427745	0,04667468	5
**CDKN1B**	1,7417E-06	0,16891441	0,04684771	5
**TTC14**	1,7512E-06	0,33537777	0,04710272	5
**CASP6**	1,7735E-06	0,38282477	0,04770105	5
**IKZF3**	1,7741E-06	0,24521639	0,04771736	5
**TLE3**	1,7908E-06	0,76940233	0,04816608	5
**U2SURP**	1,8039E-06	0,25004507	0,04851855	5
**NME3**	1,8174E-06	0,17362704	0,04888156	5
**WNK1**	1,8276E-06	0,27021876	0,04915634	5
**ARGLU1**	1,8511E-06	0,14794614	0,04978989	5
**IFNG-AS1**	3,954E-119	1,80791348	1,064E-114	6
**B2M**	1,513E-115	0,22372067	4,069E-111	6
**TMSB10**	1,047E-106	0,37023817	2,816E-102	6
**KLF2**	1,273E-79	0,52188916	3,4239E-75	6
**CCL5**	8,0415E-78	0,85977204	2,1629E-73	6
**BTG1**	1,1187E-77	0,43216566	3,0089E-73	6
**GZMK**	8,2505E-68	0,86838561	2,2191E-63	6
**ITGB1**	5,1792E-61	0,87867788	1,393E-56	6
**TMSB4X**	2,4973E-59	0,2642328	6,7171E-55	6
**CXCR3**	1,5526E-55	1,03067815	4,176E-51	6
**IL32**	1,3348E-53	0,33592805	3,5901E-49	6
**CD99**	4,6647E-53	0,45620299	1,2547E-48	6
**HLA-C**	4,8706E-53	0,37820213	1,31E-48	6
**ITGB2**	7,1143E-53	0,63454104	1,9135E-48	6
**HCST**	1,9381E-52	0,50280269	5,2128E-48	6
**TXNIP**	1,2846E-49	0,45838691	3,4552E-45	6
**ACTB**	2,8948E-45	0,29587012	7,7863E-41	6
**MS4A1**	7,6319E-45	3,04921621	2,0528E-40	6
**MALAT1**	1,2795E-44	0,30329003	3,4415E-40	6
**HLA-DPB1**	3,8178E-44	1,40407153	1,0269E-39	6
**ZNF683**	2,7318E-43	3,6299198	7,3477E-39	6
**GZMA**	5,3422E-43	0,59312564	1,4369E-38	6
**HLA-E**	1,4019E-42	0,290756	3,7707E-38	6
**RNF214**	2,909E-42	1,39109904	7,8243E-38	6
**XCL1**	1,85E-40	3,2482111	4,976E-36	6

**ITGB2-AS1**	1,7042E-39	1,0744163	4,5837E-35	6
**MT-CYB**	3,9993E-39	0,26854095	1,0757E-34	6
**ITM2A**	1,3673E-38	0,72452505	3,6776E-34	6
**TSC22D3**	2,1748E-38	0,4638404	5,8496E-34	6
**PDCD1**	9,6226E-38	2,03032889	2,5882E-33	6
**ZFP36L2**	2,5849E-36	0,40233318	6,9527E-32	6
**RAC2**	5,9145E-36	0,34976537	1,5908E-31	6
**SH3BGRL3**	1,3123E-34	0,25101757	3,5297E-30	6
**HLA-B**	4,5652E-34	0,16309662	1,2279E-29	6
**CYSLTR1**	1,1991E-31	2,24907324	3,2252E-27	6
**ARID5B**	2,1834E-31	1,00245038	5,8726E-27	6
**COTL1**	3,0753E-31	0,4308456	8,2717E-27	6
**HLA-DPA1**	5,6275E-31	1,12787408	1,5136E-26	6
**ATP1B1**	9,0219E-31	2,81115058	2,4266E-26	6
**DUSP2**	1,8384E-30	0,94093194	4,9447E-26	6
**ALOX5AP**	3,057E-30	0,51463644	8,2224E-26	6
**SMCHD1**	4,2188E-30	0,52766439	1,1347E-25	6
**KLF6**	7,6437E-30	0,51216787	2,0559E-25	6
**MT-CO1**	1,9727E-29	0,24288517	5,3059E-25	6
**FTH1**	1,9738E-29	0,23407647	5,309E-25	6
**PFN1**	2,0457E-29	0,22414988	5,5022E-25	6
**CD69**	3,5008E-29	0,5296693	9,4161E-25	6
**HLA-A**	1,8833E-28	0,22735462	5,0654E-24	6
**MT-ATP6**	2,3335E-28	0,27204042	6,2765E-24	6
**MT-CO3**	4,0895E-28	0,25458633	1,1E-23	6
**IFNG**	4,4912E-28	2,29075323	1,208E-23	6
**JUNB**	5,6832E-28	0,54241707	1,5286E-23	6
**IFITM1**	8,4856E-28	0,24301136	2,2824E-23	6
**CNN2**	8,155E-26	0,38092231	2,1935E-21	6
**C1orf162**	2,1156E-25	1,25271118	5,6903E-21	6
**INPP4B**	5,3795E-25	0,57203795	1,4469E-20	6
**PSMB9**	6,0892E-25	0,43594675	1,6378E-20	6
**CD74**	3,0016E-24	0,41094092	8,0734E-20	6
**DNAJB1**	8,0604E-24	0,45329816	2,168E-19	6
**SARAF**	3,991E-23	0,22029559	1,0735E-18	6
**RGS1**	5,6576E-23	2,03013608	1,5217E-18	6
**MYL6**	6,532E-23	0,25642602	1,7569E-18	6
**SH3BP5**	2,473E-22	0,68319873	6,6515E-18	6
**PIK3R1**	3,0903E-22	0,60356953	8,3121E-18	6
**NR3C1**	9,5226E-22	0,7779566	2,5613E-17	6
**FCRL6**	1,3822E-21	2,64727854	3,7177E-17	6

**S100A4**	4,3986E-21	0,19564728	1,1831E-16	6
**HLA-F**	9,4306E-21	0,39879518	2,5366E-16	6
**UBC**	1,9795E-20	0,23062075	5,3244E-16	6
**TUBA4A**	1,3554E-19	0,60302287	3,6457E-15	6
**PRR5L**	1,6178E-19	1,72359591	4,3515E-15	6
**CST7**	2,5966E-19	0,48064914	6,984E-15	6
**MT-ND3**	3,0142E-19	0,2139056	8,1073E-15	6
**IL10RA**	3,7528E-19	0,5473696	1,0094E-14	6
**CXCR4**	4,3351E-19	0,36338804	1,166E-14	6
**H3F3B**	1,2331E-18	0,2476706	3,3167E-14	6
**KLF10**	3,005E-18	1,43607749	8,0825E-14	6
**LINC00152**	4,1044E-18	0,93535357	1,104E-13	6
**MT-CO2**	4,5642E-18	0,1536934	1,2276E-13	6
**S100A10**	5,2597E-18	0,23287854	1,4147E-13	6
**TSPO**	8,1897E-18	0,34920138	2,2028E-13	6
**RARRES3**	1,4577E-17	0,34036227	3,9208E-13	6
**JUND**	1,6363E-17	0,29093596	4,4012E-13	6
**LEPROTL1**	2,7089E-17	0,31040852	7,2861E-13	6
**SYNE2**	3,12E-17	0,43245977	8,392E-13	6
**CLIC1**	3,1878E-17	0,29616166	8,5743E-13	6
**TPRG1**	3,2964E-17	1,73793484	8,8664E-13	6
**PTGER4**	3,326E-17	0,90332112	8,9459E-13	6
**PARP8**	6,5945E-17	0,41349806	1,7737E-12	6
**MT-ND2**	7,7073E-17	0,24398695	2,073E-12	6
**ANXA6**	1,9943E-16	0,41764711	5,3641E-12	6
**MT-ND4**	3,6572E-16	0,22364444	9,8368E-12	6
**GRK5**	4,9584E-16	1,31654569	1,3337E-11	6
**XCL2**	5,8793E-16	2,91814481	1,5814E-11	6
**PTPRC**	7,9679E-16	0,23007629	2,1431E-11	6
**PPDPF**	1,0209E-15	0,22647807	2,746E-11	6
**SLC9A3R1**	1,1396E-15	0,41585839	3,0651E-11	6
**ANXA2**	2,8615E-15	0,33922999	7,6965E-11	6
**CRIP2**	3,9007E-15	0,80753589	1,0492E-10	6
**SIGIRR**	4,5975E-15	0,42713123	1,2366E-10	6
**AHNAK**	1,1095E-14	0,3592204	2,9843E-10	6
**RP5-1028K7.2**	1,6473E-14	1,83493851	4,4308E-10	6
**ARPC1B**	2,0431E-14	0,34458278	5,4954E-10	6
**FCRL3**	2,4197E-14	2,72063612	6,5082E-10	6
**MT-ND4L**	2,5553E-14	0,14392199	6,8731E-10	6
**HNRNPA2B1**	3,2016E-14	0,22875104	8,6114E-10	6
**CD81**	4,8449E-14	0,28149906	1,3031E-09	6

**S100A11**	5,4079E-14	0,26135569	1,4546E-09	6
**IGFBP4**	6,2531E-14	1,51704702	1,6819E-09	6
**CD3E**	6,5593E-14	0,15829967	1,7643E-09	6
**ELF1**	1,1979E-13	0,45831994	3,2219E-09	6
**CNBP**	3,4862E-13	0,18602993	9,3767E-09	6
**TSTD1**	5,9254E-13	0,36552382	1,5938E-08	6
**MYL12A**	7,5273E-13	0,15620418	2,0246E-08	6
**CCND3**	8,5472E-13	0,24950278	2,2989E-08	6
**YWHAB**	8,568E-13	0,26108939	2,3045E-08	6
**KIAA1551**	1,4084E-12	0,46878635	3,7881E-08	6
**SRGN**	1,4838E-12	0,3012522	3,991E-08	6
**LCK**	1,5991E-12	0,24227098	4,301E-08	6
**RHOH**	1,8424E-12	0,38931919	4,9554E-08	6
**ESM1**	3,3939E-12	1,769122	9,1286E-08	6
**CALM3**	3,746E-12	0,38636573	1,0076E-07	6
**CD2**	3,7998E-12	0,35758177	1,022E-07	6
**LPAR6**	4,8144E-12	1,01681577	1,2949E-07	6
**LGALS1**	5,4908E-12	0,59838776	1,4769E-07	6
**TMEM2**	6,4614E-12	0,92587287	1,7379E-07	6
**PLP2**	8,4748E-12	0,30775723	2,2795E-07	6
**BUB3**	1,1938E-11	0,33929097	3,2111E-07	6
**SAT1**	1,3087E-11	0,45624531	3,52E-07	6
**DAZAP2**	1,3874E-11	0,25182125	3,7316E-07	6
**CD84**	2,1992E-11	1,18276874	5,9151E-07	6
**FOXP1**	2,2489E-11	0,36301991	6,0487E-07	6
**MT-ND1**	2,4135E-11	0,18166404	6,4916E-07	6
**NCK2**	2,5295E-11	0,34775076	6,8037E-07	6
**CALM2**	2,7336E-11	0,24059047	7,3526E-07	6
**EMP3**	3,2374E-11	0,1945871	8,7075E-07	6
**LEF1**	4,3451E-11	0,41494464	1,1687E-06	6
**ALDOA**	4,9731E-11	0,20676808	1,3376E-06	6
**ARL4C**	5,3773E-11	0,30038967	1,4463E-06	6
**BCAP31**	5,406E-11	0,29910994	1,4541E-06	6
**CD3D**	5,561E-11	0,20987236	1,4957E-06	6
**GIMAP4**	5,6611E-11	0,35314985	1,5227E-06	6
**DUSP4**	5,7993E-11	2,46593116	1,5598E-06	6
**TIGIT**	8,6505E-11	1,25313005	2,3267E-06	6
**SPINT2**	9,3683E-11	0,69326216	2,5198E-06	6
**OASL**	1,1299E-10	1,09708082	3,0391E-06	6
**YWHAZ**	1,2592E-10	0,21028929	3,3869E-06	6
**TMBIM6**	1,8456E-10	0,26451505	4,964E-06	6

**HMGB2**	1,8602E-10	0,4099955	5,0035E-06	6
**EMB**	2,0386E-10	0,36498222	5,4833E-06	6
**CHST7**	2,1422E-10	1,32203923	5,7618E-06	6
**PKM**	2,2097E-10	0,26990614	5,9435E-06	6
**FKBP8**	2,6316E-10	0,28639239	7,0782E-06	6
**MARCKSL1**	2,7954E-10	0,70748164	7,5187E-06	6
**CLU**	2,8278E-10	1,86407048	7,606E-06	6
**OPTN**	2,9463E-10	0,43252865	7,9247E-06	6
**TMA7**	3,0299E-10	0,1528471	8,1495E-06	6
**OAZ1**	3,0727E-10	0,19503216	8,2647E-06	6
**STK4**	3,5915E-10	0,30616247	9,6602E-06	6
**HMGB1**	4,1223E-10	0,19221986	1,1088E-05	6
**MYL12B**	5,4536E-10	0,18701278	1,4668E-05	6
**ATP6V0E1**	5,8704E-10	0,23840959	1,579E-05	6
**UQCR10**	5,9227E-10	0,27737049	1,593E-05	6
**ITM2B**	7,2171E-10	0,12772664	1,9412E-05	6
**SAMSN1**	8,116E-10	0,61328229	2,1829E-05	6
**GLIPR2**	8,7767E-10	0,61073287	2,3607E-05	6
**IL2RG**	8,9194E-10	0,20910964	2,399E-05	6
**CFL1**	9,8243E-10	0,14293181	2,6424E-05	6
**TNFSF8**	1,4485E-09	0,60544335	3,8961E-05	6
**IL16**	1,7095E-09	0,4122791	4,5979E-05	6
**WDR1**	1,8412E-09	0,39431968	4,9522E-05	6
**CD82**	1,8841E-09	0,57892919	5,0676E-05	6
**TRAF3IP3**	2,5089E-09	0,27936369	6,7483E-05	6
**NDUFA4**	3,0236E-09	0,20339778	8,1326E-05	6
**RHOA**	3,5936E-09	0,18539985	9,6658E-05	6
**FCGRT**	3,7573E-09	0,8507245	0,00010106	6
**LSP1**	4,1383E-09	0,1836791	0,00011131	6
**CSK**	4,4222E-09	0,34922311	0,00011894	6
**STAT1**	5,0496E-09	0,59502403	0,00013582	6
**CD52**	5,066E-09	0,16099003	0,00013626	6
**CCDC167**	6,1662E-09	0,52287537	0,00016585	6
**SRSF5**	6,3654E-09	0,1737709	0,00017121	6
**MT-ND5**	6,8995E-09	0,19183996	0,00018558	6
**LY6E**	7,388E-09	0,18500557	0,00019871	6
**LCP1**	7,6341E-09	0,2335531	0,00020533	6
**PHLDA2**	8,23E-09	1,81247955	0,00022136	6
**LMNA**	9,7781E-09	0,87413574	0,000263	6
**WIPF1**	1,0594E-08	0,37752362	0,00028495	6
**ANXA1**	1,5045E-08	0,15820055	0,00040467	6

**SAMD3**	1,72E-08	0,61426648	0,00046264	6
**GNAI2**	2,2131E-08	0,2185819	0,00059524	6
**TBC1D10C**	2,3007E-08	0,29465663	0,00061882	6
**ZNF365**	2,3217E-08	1,73484633	0,00062447	6
**SRRM1**	2,5362E-08	0,29425918	0,00068215	6
**SYPL1**	2,5549E-08	0,49847244	0,00068719	6
**LITAF**	2,6112E-08	0,33659955	0,00070234	6
**CSF1**	2,688E-08	1,38726294	0,000723	6
**PSMB10**	2,9341E-08	0,28798767	0,00078919	6
**NELL2**	3,0611E-08	0,46951361	0,00082334	6
**ARPC2**	3,263E-08	0,18402446	0,00087766	6
**H3F3A**	3,3311E-08	0,15124718	0,00089597	6
**SEPW1**	3,6695E-08	0,25171928	0,00098698	6
**HNRNPU**	3,8722E-08	0,3223282	0,0010415	6
**SON**	3,8934E-08	0,23561129	0,0010472	6
**GRK6**	4,1611E-08	0,33817863	0,00111922	6
**APOBEC3G**	4,3281E-08	0,51572248	0,00116412	6
**CYBA**	4,5129E-08	0,1584149	0,00121383	6
**MBP**	4,8959E-08	0,42208903	0,00131684	6
**ATXN1**	5,2533E-08	0,58554461	0,00141299	6
**GZMM**	5,3999E-08	0,29115748	0,00145242	6
**NIN**	6,4398E-08	0,62248375	0,00173212	6
**SRP14**	6,8003E-08	0,16152724	0,00182907	6
**PTGES3**	7,053E-08	0,22775994	0,00189703	6
**RAP1A**	7,5536E-08	0,31398912	0,0020317	6
**LYL1**	8,2796E-08	1,19500039	0,00222696	6
**NCF1**	9,2097E-08	0,51566435	0,00247712	6
**SFXN1**	9,451E-08	0,51728053	0,00254204	6
**EPC1**	9,6432E-08	0,27798856	0,00259372	6
**F2R**	1,1301E-07	1,10295194	0,0030397	6
**ANXA11**	1,3219E-07	0,24106963	0,0035556	6
**AGTRAP**	1,5213E-07	0,71053946	0,00409187	6
**ARAP2**	1,7444E-07	0,55122651	0,00469181	6
**SP100**	1,7604E-07	0,28324717	0,00473497	6
**JUN**	1,8609E-07	0,33238297	0,00500528	6
**SUSD3**	1,8812E-07	0,50333098	0,0050598	6
**FYB**	2,1405E-07	0,1902732	0,00575741	6
**ANKRD12**	2,1469E-07	0,25872322	0,00577453	6
**RAB37**	2,2976E-07	0,70597439	0,00617988	6
**CAP1**	2,4682E-07	0,28536421	0,00663874	6
**ST8SIA1**	2,588E-07	1,29538276	0,0069609	6

**SKP1**	2,7262E-07	0,19370383	0,00733271	6
**CHCHD2**	3,0604E-07	0,16038512	0,00823165	6
**EIF1**	4,0115E-07	0,12301603	0,01078968	6
**UBB**	4,4515E-07	0,16245715	0,01197313	6
**PAM**	4,4904E-07	0,72371035	0,01207789	6
**PRELID2**	5,0857E-07	2,14801634	0,01367905	6
**GSTK1**	5,4625E-07	0,14782909	0,01469253	6
**COX6A1**	5,5472E-07	0,163633	0,01492038	6
**SH3KBP1**	5,6502E-07	0,3674076	0,01519747	6
**RASAL3**	6,5601E-07	0,46811292	0,01764481	6
**PPP3CC**	6,5895E-07	0,42407372	0,01772378	6
**ARPC3**	7,28E-07	0,15581145	0,01958091	6
**PRMT2**	8,5302E-07	0,22951752	0,02294368	6
**WAS**	8,7088E-07	0,27581968	0,02342419	6
**TSPYL2**	8,867E-07	0,58760501	0,02384961	6
**ITK**	1,004E-06	0,38667917	0,0270039	6
**RCSD1**	1,0286E-06	0,31271596	0,02766558	6
**PTGDR**	1,2328E-06	0,77400845	0,0331573	6
**FYN**	1,2409E-06	0,23528123	0,0333762	6
**ANAPC16**	1,447E-06	0,22085197	0,03891968	6
**IQGAP2**	1,4534E-06	0,37690227	0,03909087	6
**PRKCH**	1,4579E-06	0,31102856	0,03921389	6
**SEPT1**	1,4742E-06	0,21102414	0,03965152	6
**MYH9**	1,5293E-06	0,28767584	0,04113455	6
**LIMD2**	1,7206E-06	0,13811451	0,04628014	6
**STT3B**	1,8159E-06	0,46049495	0,04884309	6
**DDX5**	1,8337E-06	0,15783354	0,04932153	6
**IFIT3**	0	6,09765966	0	7
**IFIT1**	2,143E-291	5,35797501	5,764E-292	7
**RSAD2**	1,156E-251	5,06598195	3,11E-247	7
**OAS1**	1,861E-213	3,89648837	5,006E-209	7
**IFI44L**	7,117E-145	3,42675551	1,914E-140	7
**OASL**	2,88E-118	4,26744693	7,747E-114	7
**MX1**	1,996E-113	3,39711469	5,37E-109	7
**CMPK2**	1,818E-112	3,57371503	4,891E-108	7
**EPSTI1**	2,716E-103	2,82904272	7,305E-99	7
**STAT1**	2,7962E-87	2,54885614	7,5209E-83	7
**ISG15**	1,2989E-86	2,92437246	3,4936E-82	7
**OAS3**	4,5534E-83	3,15404727	1,2247E-78	7
**XAF1**	5,4801E-72	2,53055793	1,474E-67	7
**IFI6**	1,3165E-67	3,04221246	3,5411E-63	7

**IFIT2**	1,5374E-67	4,58543409	4,1351E-63	7
**LAMP3**	8,0496E-66	4,48956281	2,1651E-61	7
**GBP1**	1,1741E-61	2,43985204	3,1579E-57	7
**HERC5**	2,7691E-59	2,71917135	7,4479E-55	7
**IFI44**	2,3911E-46	2,14202097	6,4314E-42	7
**IFITM1**	5,2528E-46	0,93596915	1,4128E-41	7
**LY6E**	2,6599E-45	1,25214916	7,1544E-41	7
**IRF7**	4,8271E-42	2,27408663	1,2983E-37	7
**USP18**	1,5423E-39	2,74042489	4,1484E-35	7
**PARP9**	4,2907E-34	2,09122158	1,1541E-29	7
**IFIH1**	1,7743E-33	2,70150756	4,7722E-29	7
**EIF2AK2**	9,1398E-33	1,87816009	2,4583E-28	7
**IFI35**	1,3125E-30	2,00333719	3,5301E-26	7
**OAS2**	8,1151E-26	1,92232273	2,1827E-21	7
**MX2**	9,3601E-26	1,91729826	2,5176E-21	7
**TRIM22**	6,4238E-25	1,36637377	1,7278E-20	7
**ISG20**	9,362E-25	1,04112304	2,5181E-20	7
**BST2**	2,0904E-24	1,35810351	5,6225E-20	7
**PLSCR1**	1,6613E-23	2,11259175	4,4683E-19	7
**ETV7**	1,1516E-22	2,63181598	3,0975E-18	7
**UBE2L6**	1,5627E-22	1,22537879	4,2033E-18	7
**GBP4**	4,0607E-22	1,67324958	1,0922E-17	7
**RP1-71H24.1**	1,2767E-21	3,40797539	3,4339E-17	7
**LGALS9**	2,6039E-20	2,00452155	7,0036E-16	7
**IFI16**	4,2404E-20	1,34055576	1,1405E-15	7
**AP001610.5**	4,3642E-20	3,17434091	1,1738E-15	7
**TYMP**	2,0397E-19	1,70157547	5,4861E-15	7
**GBP5**	2,5992E-19	1,30884092	6,9911E-15	7
**LAP3**	4,5783E-19	1,55993367	1,2314E-14	7
**SAMD9**	4,8026E-19	1,63360735	1,2917E-14	7
**HELZ2**	5,4424E-19	2,64723602	1,4638E-14	7
**HERC6**	7,3241E-19	1,85867494	1,97E-14	7
**TNFSF10**	9,0301E-19	1,45831418	2,4288E-14	7
**PSMB9**	1,1031E-18	0,79675398	2,967E-14	7
**SAMD9L**	9,0371E-18	1,77003834	2,4307E-13	7
**TAP1**	2,0595E-17	1,04399326	5,5396E-13	7
**DDX60**	2,1515E-17	1,94959104	5,7868E-13	7
**PSME2**	1,635E-16	0,88801274	4,3976E-12	7
**SP100**	6,2154E-16	0,94061965	1,6717E-11	7
**BCL2L14**	1,7538E-15	3,44036289	4,7172E-11	7
**GBP2**	3,0733E-15	1,18203061	8,2662E-11	7

**IFI27**	7,3582E-15	3,88157791	1,9791E-10	7
**ODF3B**	6,8619E-14	1,83605928	1,8456E-09	7
**RTP4**	8,3656E-14	2,02229602	2,2501E-09	7
**C19orf66**	9,8519E-14	0,93719393	2,6499E-09	7
**DDX58**	1,5676E-13	1,5670379	4,2165E-09	7
**PARP14**	1,005E-12	1,48366726	2,7031E-08	7
**PHF11**	1,26E-12	0,967293	3,3891E-08	7
**DRAP1**	1,5969E-12	0,74042187	4,2952E-08	7
**MT2A**	1,923E-12	1,08016771	5,1723E-08	7
**IRF1**	2,098E-12	0,86478735	5,6431E-08	7
**IFIT5**	4,5195E-12	1,70065624	1,2156E-07	7
**STAT2**	4,9678E-12	1,56337057	1,3362E-07	7
**HSH2D**	5,1372E-12	1,58834344	1,3817E-07	7
**PMAIP1**	1,0814E-11	3,28257711	2,9085E-07	7
**DTX3L**	2,9935E-11	1,28185196	8,0517E-07	7
**IFITM2**	3,3785E-11	0,53616648	9,0873E-07	7
**RNF213**	5,522E-11	0,77256415	1,4853E-06	7
**PARP10**	6,3899E-11	1,26844628	1,7187E-06	7
**DHX58**	8,7824E-11	1,65990663	2,3622E-06	7
**RBCK1**	2,3422E-10	0,84318888	6,2997E-06	7
**PARP12**	7,1297E-10	1,23492472	1,9177E-05	7
**ASB16**	1,2952E-09	4,22786675	3,4838E-05	7
**PSME1**	1,3688E-09	0,43917551	3,6817E-05	7
**CHMP5**	1,3732E-09	1,08244704	3,6935E-05	7
**B2M**	1,4691E-09	0,1526471	3,9515E-05	7
**ZBP1**	4,8978E-09	1,36416184	0,00013174	7
**CD74**	6,3333E-09	0,49697301	0,00017035	7
**LGALS3BP**	8,607E-09	1,18589538	0,0002315	7
**DDX60L**	1,0821E-08	1,38406706	0,00029105	7
**ADAR**	2,1117E-08	0,75208834	0,00056797	7
**HLA-B**	3,3127E-08	0,19018278	0,00089101	7
**HAPLN3**	3,5195E-08	1,27936858	0,00094665	7
**COLGALT2**	3,6237E-08	3,80230796	0,00097466	7
**TRIM21**	4,7542E-08	1,37075893	0,00127875	7
**ZCCHC2**	5,7042E-08	1,11699002	0,00153426	7
**RP11-388M20.6**	5,8014E-08	3,51489399	0,0015604	7
**SMCHD1**	5,9285E-08	0,59291297	0,00159458	7
**JAK2**	6,7686E-08	1,32572611	0,00182055	7
**NEXN**	7,9617E-08	2,71727181	0,00214146	7
**RAB23**	9,8078E-08	3,4680445	0,00263799	7
**GRAMD1C**	1,2176E-07	2,87987245	0,00327501	7

**ZC3HAV1**	1,3042E-07	1,13426903	0,00350784	7
**BACE1-AS**	2,4173E-07	3,88017875	0,00650172	7
**DNAJB6**	3,5764E-07	0,8376951	0,00961941	7
**N4BP1**	4,2691E-07	1,04859625	0,01148256	7
**SAT1**	5,0382E-07	0,691329	0,01355137	7
**BCL3**	5,1015E-07	0,87646731	0,01372164	7
**PSMB8**	6,0445E-07	0,50312895	0,01625799	7
**BISPR**	6,0546E-07	1,09111826	0,01628509	7
**SDCBP2**	8,4328E-07	3,38539974	0,02268182	7
**SLFN5**	9,4914E-07	0,73820029	0,02552895	7
**TMEM123**	1,0877E-06	0,39858521	0,0292554	7
**STX11**	1,099E-06	1,48082532	0,02955932	7
**PSMB10**	1,1171E-06	0,54928523	0,03004623	7
**PNPT1**	1,4234E-06	1,43313735	0,03828412	7
**CTD-2095E4.5**	1,5762E-06	1,76813858	0,0423942	7
**HDX**	1,7553E-06	2,79578558	0,04721276	7
