# Supplementary material for: Refined single-cell profiling captures a CCR5high CD4+ cytotoxic T-cell precursor in multiple sclerosis
Source: eBioMedicine. 2026 Jun 6;129:106324. doi: 10.1016/j.ebiom.2026.106324 (PMC13264205; doi:10.1016/j.ebiom.2026.106324)
Supplement: Cell-Line-Checklist [file mmc2.pdf]

ICLAC ■ International Cell Line Authentication Committee

Cell Line Checklist for Manuscripts and Grant Applications

This checklist is a resource for scientists who write or review manuscripts and/or for grant applications that use cell lines. Cross-contaminated cell lines could give unreliable results if used for research for various reasons, such as failure to represent the correct species, tissue type or disease state. Such misidentified or false cell lines produce unreliable research data, and we urge reviewers to highlight their use wherever possible.

This checklist will help the author or reviewer to look for obvious cell line quality concerns. The checklist may also be used to communicate any quality concerns to be addressed prior to publication or funding.

General Recommendations:

1) Cell line RRIDs are assigned through a collaboration between Cellosaurus and the Resource Identification Initiative. RRIDs can be found by searching for cell lines at https://www.cellosaurus.org

2) ICLAC recommends that false cell lines (misidentified cell lines with no known authentic stock) should not be used. ICLAC's register of misidentified cell lines can be found at http://iclac.org/databases/cross-contaminations.

3) ICLAC recommends that authentication testing be performed on established cell lines regardless of the application; the test method and results should be included in the Materials and Methods section. Testing should be done at the beginning and end of experimental work, at minimum.

   For human cell lines, short tandem repeat (STR) profiling should be performed and compared to results from donor tissue, or to online databases of human cell line STR reference profiles.
   More information can be found in the published Standard: ANSI/ATCC ASN-0002-2022 Human Cell Line Authentication: Standardization of Short Tandem Repeat (STR) Profiling. ANSI Webstore.

   For non-human cell lines, best practices will vary with the species being tested. At minimum, species should be confirmed using an appropriate method such as karyotyping, isoenzyme analysis, or mitochondrial DNA typing (DNA barcoding). Detailed information can be found regarding DNA barcoding on the published standard: ANSI/ATCC ASN-0003-2015 Species-Level Identification of Animal Cells Through Mitochondrial Cytochrome C Oxidase Subunit 1 (CO1) DNA Barcodes.
   More information on authentication testing can be found at http://iclac.org/references/.

4) It will be helpful for the reader if the authors can include a reference, to provide more information on the cell line's establishment and characterization. However, not all cell lines have this information available in the public domain.

5) Sufficient information should be given to replicate experiments using the cell lines. This includes the growth medium used, including additives; any additional growth requirements, including special substrates and gas mixtures; and the passage number or population doubling level (PDL) used for experimental work.
   Passage number is important when working with early passage or finite cultures, or cell lines where changes in phenotype have been documented with increasing passage. ICLAC recommends that laboratories freeze down stocks when they first receive a cell line and set a limit (e.g., 20 passages) to avoid overpassaging. More information can be found at http://iclac.org/resources/advice-scientists/

Manuscript or Grant Information

Title or Manuscript/Grant ID:	EBIOM-D-25-04605R3
Cell Lines used:	hCMEC/D3
Cell Lines used with Quality Concerns:	-

Indicate "Yes" or "No" for each question below and further comments in the next page. Add new pages, if necessary.

**Cell line designation** □<br>□ **Reporting Requirements**	hCMEC/D3				
The RRID for the cell line is listed?[1]	Yes				
The cell line is known to be problematic? [2]	No				
Authentication testing was performed? [3]	Yes				
Human cell lines: STR profile is available with the manuscript/grant application? [3]	No				
Mycoplasma testing has been performed? [4]	Yes				
The source for the cell line is listed? [5]	Yes				
Sufficient information is given to replicate experiments using the cell line?[6]	Yes				

---

[1] The Resource Identification Initiative (RRID) is meant to help researchers cite the important resources used in scientific papers (See Recommendation #1).
[2] See the ICLAC website for a register of known misidentified cell lines (See Recommendation #2 on Page 1 of this document).
[3] The authentication test method and results should be listed in the manuscript/project (See Recommendation #3 on Page 1 of this document).
[4] The mycoplasma test method and results should be listed in the manuscript/project.
[5] The catalogue number should be included if obtained from a cell line repository. See also Recommendation #4 on Page 1 of this document.
[6] See Recommendation #5 on Page 1 of this document.

Notes or further Comments

RRID: CVCL_U985

STR is performed for each lot of cells to verify unique identity (performed by manufacturer, see validation document).
