# Supplementary material for: Refined single-cell profiling captures a CCR5high CD4+ cytotoxic T-cell precursor in multiple sclerosis
Source: eBioMedicine. 2026 Jun 6;129:106324. doi: 10.1016/j.ebiom.2026.106324 (PMC13264205; doi:10.1016/j.ebiom.2026.106324)
Supplement: Validation document_hCMECD3 [file mmc3.pdf]

Blood-Brain Barrier hCMEC/D3 Cell Line

Immortalized Cell Line

Cat. # SCC066

FOR RESEARCH USE ONLY
NOT FOR USE IN DIAGNOSTIC PROCEDURES
NOT FOR HUMAN OR ANIMAL CONSUMPTION
THIS PRODUCT CONTAINS GENETICALLY MODIFIED ORGANISMS

Pack size: ≥1X10^6 cells/vial

Store at Liquid Nitrogen

Certificate of Analysis

Background

The blood–brain barrier (BBB) is a highly selective permeability barrier that separates the circulating blood from the brain extracellular fluid in the central nervous system. The blood–brain barrier is formed by capillary endothelial cells, which are connected by tight junctions with an extremely high electrical resistivity. The blood–brain barrier allows the passage of water, some gases, and lipid soluble molecules by passive diffusion, as well as the selective transport of molecules such as glucose and amino acids that are crucial to neural function. On the other hand, the blood–brain barrier may prevent the entry of lipophilic, potential neurotoxins by way of an active transport mechanism mediated by P-glycoprotein. Astrocytes are necessary to create the blood–brain barrier. The blood–brain barrier (BBB) prevents entry into the brain of most drugs from the blood. The presence of the BBB makes difficult the development of new treatments of brain diseases, or new radiopharmaceuticals for neuroimaging of brain.

The hCMEC/D3 cell line was derived from human temporal lobe microvessels isolated from tissue excised during surgery for control of epilepsy. The primary isolate was enriched in cerebral endothelial cells (CECs). In the first passage, cells were sequentially immortalized by lentiviral vector transduction with the catalytic subunit of human telomerase (hTERT) and SV40 large T antigen, following which CEC were selectively isolated by limited dilution cloning, and clones were extensively characterized for brain endothelial phenotype. This brain microvascular endothelial cell line represents one such model of the human BBB that can be easily grown and is amenable to cellular and molecular studies on pathological and drug transport mechanisms with relevance to the central nervous system (CNS).

Source

The Blood-Brain Barrier hCMEC/D3 cell line was isolated from a consenting normal human donor following established protocols.

Storage and Handling

Blood-Brain Barrier hCMEC/D3 cells should be stored in liquid nitrogen. The cells can be passage for at least 10 passages without significantly affecting the cell marker expression and functionality.

Quality Control Testing

- Each vial contains ≥ $1X10^6$ viable cells.
- Cells are tested by PCR and are negative for Hepatitis A, B, C, HPV, Herpes and HIV-1 & 2 viruses.
- Cells are negative for mycoplasma contamination.
- Each lot of cells is genotyped by STR analysis to verify the unique identity of the cell line.

References

Couraud PO, et al. (2008) The human brain endothelial cell line hCMEC/D3 as a human blood-brain barrier model for drug transport studies. Fluids Barriers CNS. 2013 Mar 26;10(1):16.

Dauchy S, et al. (2009) Expression and transcriptional regulation of ABC transporters and cytochromes P450 in hCMEC/D3 human cerebral microvascular endothelial cells. Biochem Pharmacol. 2009 Mar 1;77(5):897-909.

Markoutsa E, et al. (2011) Uptake and permeability studies of BBB-targeting immunoliposomes using the hCMEC/D3 cell line. Eur J Pharm Biopharm. 2011 Feb;77(2):265-74.

Couraud PO., et al. (2013) The hCMEC/D3 cell line as a model of the human blood brain barrier. Fluids Barriers CNS. 2013 Mar 26;10(1):16.

Coureuil M, et al. (2009) Meningococcal type IV pili recruit the polarity complex to cross the brain endothelium. Science. 2009 Jul 3;325(5936):83-7.

Schreibelt G, et al. (2007) Reactive oxygen species alter brain endothelial tight junction dynamics via RhoA, PI3 kinase, and PKB signaling. FASEB J. 2007 Nov;21(13):3666-76.

Tai LM, et al. (2009) P-glycoprotein and breast cancer resistance protein restrict apical-to-basolateral permeability of human brain endothelium to amyloid-beta. J Cereb Blood Flow Metab. 2009 Jun;29(6):1079-83.

Coureuil M, et al. (2010) Meningococcus Hijacks a β2-adrenoceptor/β-Arrestin pathway to cross brain microvasculature endothelium. Cell. 2010 Dec 23;143(7):1149-60.

Protocols

hCMEC/D3 Medium Preparation

Cells are cultured in EndoGRO™-MV Complete Media Kit (Cat. No. SCME004) supplemented with 1 ng/mL FGF-2 (Cat. No. GF003).

ECM Coating of Flasks

1. Thaw Collagen Type I, Rat Tail (Cat. No. 08-115) at room temperature.
2. Dilute 1 mL of Collagen Type I with 19 mL 1X PBS (Cat. No. BSS-1005-B). Mix gently. Scale up according to the volumes required.
3. Coat flask with 1:20 diluted Collagen Type I solution. Use 5-10 mL for T75 flasks and 15-25 mL for T225 flasks. Incubate in 37°C incubator for at least one hour before use.

   Note: Flasks may be coated 5-6 days in advance and stored at 2-8°C in the coating solution.
4. Aspirate the coating solution just before plating the cells.

Thawing of Cells

1. **Do not thaw the cells until the recommended medium is on hand.** Cells are thawed in hCMEC/D3 Medium (see hCMEC /D3 Medium Preparation above).
2. Remove the vial of hCMEC/D3 cells from liquid nitrogen and incubate in a 37°C water bath. Closely monitor until the cells are completely thawed. Maximum cell viability is dependent on the rapid and complete thawing of frozen cells.

   IMPORTANT: Do not vortex the cells.
3. As soon as the cells are completely thawed, disinfect the outside of the vial with 70% ethanol. Proceed immediately to the next step.
4. In a laminar flow hood, use a 1 or 2 mL pipette to transfer the cells to a sterile 15 mL conical tube. Be careful not to introduce any bubbles during the transfer process.
5. Using a 10 mL pipette, slowly add dropwise 9ml of hCEMC/D3 Medium (pre-warmed to 37°C) to the 15 mL conical tube.

   IMPORTANT: Do not add the whole volume of media at once to the cells. This may result in decreased cell viability due to osmotic shock.
6. Gently mix the cell suspension by slow pipeting up and down twice. Be careful to not introduce any bubbles.

   IMPORTANT: Do not vortex the cells.
7. Centrifuge the tube at 300 x g for 2-3 minutes to pellet the cells.
8. Decant as much of the supernatant as possible. Steps 5-8 are necessary to remove residual cryopreservative (DMSO).
9. Resuspend the cells in a total volume of 10 -12 mL hCMEC/D3 medium (pre-warmed to 37°C).
10. Plate the cell mixture onto a pre-coated T75 tissue culture flask (See section on ECM Coating of Flasks).
11. Incubate the cells at 37°C in a 5% CO_2 humidified incubator.
12. The next day, exchange the medium with fresh hCMEC/D3 Medium pre-warmed to 37°C. Exchange with fresh medium every two to three days thereafter.
13. When the cells are approximately 80% confluent (3-4 days after plating cells at the density they can be dissociated with Accumax™ (Cat. No. SCR006) or trypsin-EDTA (Cat. No. SM-2003-C) and passaged or alternatively frozen for later use.

Subculturing of Cells

1. Carefully remove the medium from the T75 tissue culture flask containing the confluent layer of hCMEC/D3 cells.
2. Apply 3-5 mL of Accumax™ or trypsin-EDTA solution and incubate in a 37°C incubator for 3-5 minutes.
3. Inspect the plate and ensure the complete detachment of cells by gently tapping the side of the plate with the palm of your hand.
4. Add 8 mL of hCMEC/D3 medium (pre-warmed to 37°C) to the plate.
5. Gently rotate the plate to mix the cell suspension. Transfer the dissociated cells to a 15 mL conical tube.
6. Centrifuge the tube at 300 x g for 3-5 minutes to pellet the cells.
7. Discard the supernatant.
8. Apply 2 mL of hCMEC/D3 media (pre-warmed to 37°C) to the conical tube and resuspend the cells thoroughly.

   IMPORTANT: Do not vortex the cells.
9. Count the number of cells using a hemocytometer.
10. Plate the cells to the desired density (typical split ratio is 1:3 to 1:6).

Cryopreservation of Cells

hCMEC/D3 cells can be frozen in hCMEC/D3 Medium with 10% DMSO using a Nalgene slow freeze Mr. Frosty container.

We Buy 100% Certified Renewable Energy
FOR RESEARCH USE ONLY. Not for use in diagnostic procedures. Not for human or animal consumption. Purchase of this Product does not include any right to resell or transfer, either as a stand-alone product or as a component of another product. Any use of this Product for purposes other than research is strictly prohibited.
Millipore®, the M mark, Upstate®, Chemicon®, Linco® and all other trademarks, unless specifically identified above in the text as belonging to a third party, are owned by Merck KGaA, Darmstadt. Copyright ©2008-2020 Merck KGaA, Darmstadt. All rights reserved.

Representative Lot Data

hCMEC/D3 Cells 48 hrs after thaw

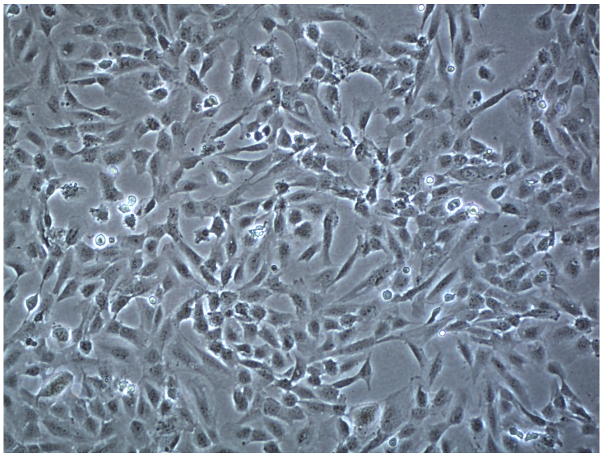

hCMEC/D3 Cells CD144 (VE-Cadherin) Expression

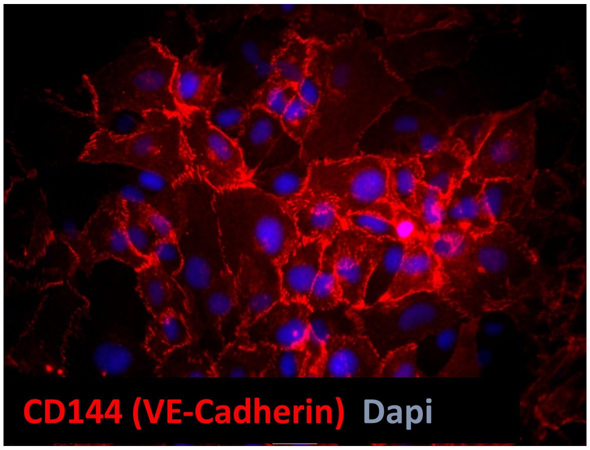

hCMEC/D3 Cells CD31 (PECAM-1) Expression

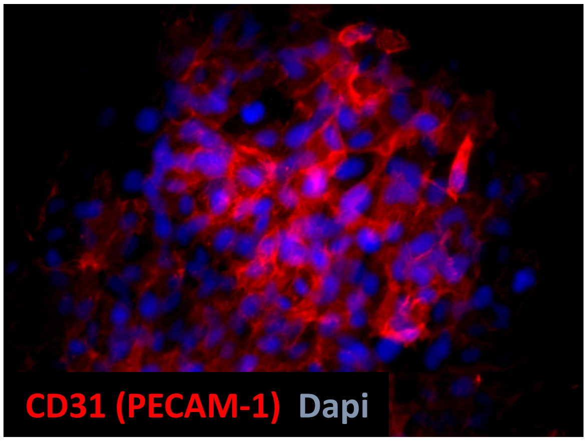

hCMEC/D3 Cells CD105 (Endoglin) Expression

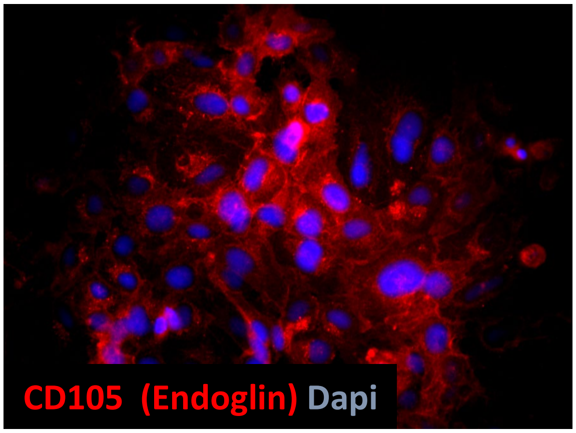

hCMEC/D3 Cells CD36 Expression

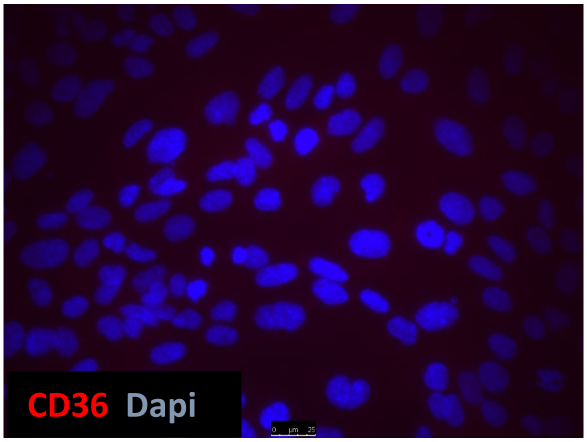

Please visit www.millipore.com for additional product information, test data and references
EMD Millipore Corporation, 28820 Single Oak Drive, Temecula, CA 92590, USA 1-800-437-7500
Technical Support: T: 1-800-MILLIPORE (1-800-645-5476) • F: 1-800-437-7502
We Buy 100% Certified Renewable Energy
Green-e.org
FOR RESEARCH USE ONLY. Not for use in diagnostic procedures. Not for human or animal consumption. Purchase of this Product does not include any right to resell or transfer, either as a stand-alone product or as a component of another product. Any use of this Product for purposes other than research is strictly prohibited.
Millipore®, the M mark, Upstate®, Chemicon®, Linco® and all other trademarks, unless specifically identified above in the text as belonging to a third party, are owned by Merck KGaA, Darmstadt. Copyright ©2008-2020 Merck KGaA, Darmstadt. All rights reserved.

RESTRICTED USE AGREEMENT
(subject to local law)

THIS PRODUCT MAY ONLY BE USED FOR RESEARCH PURPOSES, WHICH IS FURTHER DEFINED BELOW. BY OPENING THIS PRODUCT, YOU ("PURCHASER") HEREBY REPRESENT THAT YOU HAVE THE RIGHT AND AUTHORITY TO LEGALLY BIND YOURSELF AND/OR YOUR EMPLOYER, AS APPLICABLE, AND CONSENT TO BE LEGALLY BOUND BY THE TERMS OF THIS RESTRICTED USE AGREEMENT. IF YOU DO NOT AGREE TO COMPLY WITH THESE TERMS, YOU MAY NOT OPEN OR USE THE PRODUCT AND YOU MUST CALL MILLIPORESIGMA ("SELLER") CUSTOMER SERVICE (1-800-645-5476) TO ARRANGE TO RETURN THE PRODUCT FOR A REFUND.

"Product" means Blood-Brain Barrier hCMEC/D3 Cell Line (SCC066).

"Research Purposes" means any internal *in vitro* research use and specifically excludes the following uses of whatever kind or nature:

- Re-engineering or copying the Product
- Making derivatives, modifications, or functional equivalents of the Product
- Obtaining patents or other intellectual property rights claiming use of the Product
- Using the Product in the development, testing, or manufacture of a Commercial Product
- Using the Product as a component of a Commercial Product
- Reselling or licensing the Product
- Using the Product in clinical or therapeutic applications including producing materials for clinical trials
- Administering the Product to humans
- Using the Product in collaboration with a commercial or non-academic entity

"Commercial Product" means any product intended for: (i) current or future sale; (ii) use in a fee-for-service; or (iii) any diagnostic, clinical, or therapeutic use.

Access to the Product is limited solely to PURCHASER's officers, employees, and students who need to use the Product for Research Purposes. PURCHASER shall comply with all applicable laws in its use and handling of the Product and shall keep it under reasonably safe and secure conditions to prevent unauthorized use or access.

These restrictions will remain in effect for as long as PURCHASER possesses the Product.

PLEASE CONTACT licensing@emdmillipore.com PRIOR TO PURCHASE FOR ANY USE OF THE PRODUCT OUTSIDE OF THIS RESTRICTED USE AGREEMENT.

GMO
This product contains genetically modified organisms.
Este producto contiene organismos genéticamente modificados.
Questo prodotto contiene degli organismi geneticamente modificati.
Dieses Produkt enthält genetisch modifizierte Organismen.
Ce produit contient des organismes génétiquement modifiés.
Dit product bevat genetisch gewijzigde organismen.
Tämä tuote sisältää geneettisesti muutettuja organismeja.
Denna produkt innehåller genetiskt ändrade organismer.

We Buy 100% Certified Renewable Energy
Green-e.org
FOR RESEARCH USE ONLY. Not for use in diagnostic procedures. Not for human or animal consumption. Purchase of this Product does not include any right to resell or transfer, either as a stand-alone product or as a component of another product. Any use of this Product for purposes other than research is strictly prohibited.
Millipore®, the M mark, Upstate®, Chemicon®, Linco® and all other trademarks, unless specifically identified above in the text as belonging to a third party, are owned by Merck KGaA, Darmstadt. Copyright ©2008-2020 Merck KGaA, Darmstadt. All rights reserved.
